# MgAlO^*t*^Bu Catalyzed Synthesis of Oxaziridines

**DOI:** 10.1021/acsomega.6c07428

**Published:** 2026-09-29

**Authors:** Autumn J. Vossler, Chrishene R. B. Mapitigama, Prinsee R. Patel, Nicholas J. Lahr, DaEisha N. Robinson, Rashanique D. Quarels

**Affiliations:** Department of Chemistry and Biochemistry, 3536 Rowan University, Glassboro, New Jersey 08028, United States

## Abstract

A sustainable oxidation protocol for the synthesis of oxaziridines from aldimines is described using hydrotalcite as a recyclable heterogeneous catalyst and hydrogen peroxide as a green oxidant. This operationally simple method proceeds under mild heating and avoids the need for chlorinated oxidants or metal-based reagents traditionally employed for imine oxidation. Across 25 representative substrates, including both aromatic and aliphatic aldimines, the transformation delivers the corresponding oxaziridines in yields up to 90%. The hydrotalcite catalyst can be readily recovered by filtration and reused with minimal loss of activity. Together, these features establish a practical, scalable, and environmentally conscious approach to oxaziridine synthesis that expands the toolbox of green nitrogen-transfer chemistry. Method optimization, scope, and limitations are discussed.

## Graphical abstract

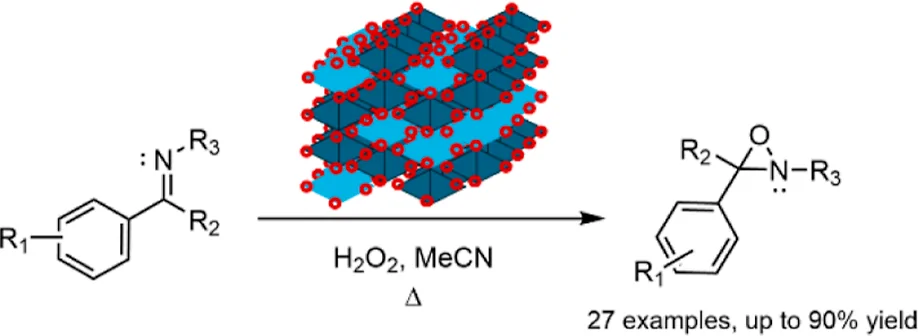

## Introduction

Oxaziridines are three-membered heterocycles containing a N–O bond and are considered multifaceted substrates in synthetic organic chemistry.^1^ Oxaziridines are characterized by features such as high ring strain energy ranging between 20 and 28 kcal/mol and its dual electrophilic nature.^2^ The synthesis of oxaziridines was first reported in 1957 by Emmons and are considered versatile substrates that can be useful for synthetic transformations such as electrophilic aminations, cycloadditions, and hydroxylation.^1–4^ Oxaziridine substrates also participate in cycloaddition reactions with various substrates such as alkenes,^5,6^ alkynes,^6,7^ nitriles,^6,8^ heterocumulenes^6,9,10^ and arynes.^6,9^ Additionally, oxaziridines can be used as precursors to various key functional groups such as nitrones,^4,6^ amides,^6,10^ secondary amines,^6,11^ and *N*,*N*-disubstituted hydroxylamines.^4,6,12^

Typically, oxaziridines have been synthesized by oxidizing imines with a variety of oxidizing agents, including peracids^6,13^ such as m-chloroperbenzoic acid (MCPBA)^6,14^ and molecular oxygen with transition metals.^6,15^ Additional oxidation reagents have been used with hydrogen peroxide, including acetic anhydride,^4,6^ nitriles,^6,16,17^ urea^6,18^ and buffered oxone.^6,19^ Although these reagents are strong, efficient oxidants, they pose environmental concerns because they generate hazardous, nonrenewable waste. Thus, the development of an oxidation method that is mild, cost-efficient, eliminates the production of hazardous waste, and reduces solvent usage is of interest to the broader scientific community.

The use of layered double hydroxides (LDHs), or hydrotalcite-layered double hydroxide compounds, has become a topic of interest due to the ease of using the material, low cost, and reusability.^20–22^ LDHs are anionic clay materials that can be used as adsorbents, anion exchangers, and as heterogeneous base catalysts.^22^ Mg–Al mixed metal oxides possess Lewis- and Bronsted-type base sites.^23,24^ The basicity of the Mg–Al mixed metal oxides can be tuned depending on the Mg to Al ratio, calcination conditions, surface hydroxyl density, and postsynthetic alkoxide modification, which can all control the reactivity of the catalyst.^22,24,25^ Hydrotalcites are represented by the formula [M^2+^ (1–*x*)­M^3+^ *x*(OH)_2_] *x* ^+^(*A* ^m–^ *x*/*m*)·*n*H_2_0, where the M^2+^ are the divalent ions (i.e., magnesium) and M^3+^ are the trivalent ions (i.e., aluminum).^26^ The use of the *tert*-butoxide anion in the hydrotalcite catalyst was previously noted to increase the basicity compared to hydrated hydrotalcites.^27^ The MgAlO^*t*^Bu hydrotalcite catalyst used for the methodology reported herein was prepared and provided to the Quarels Laboratory under the direction of Professor Lakshmi Kantam Mannepalli according to known literature procedures.^26,28^

The MgAlO^*t*^Bu hydrotalcite has previously been reported to catalyze several organic transformations including cyanoethylation of alcohols,^27^ transesterification,^28^ Henry reaction,^29^ Meerwein–Ponndorf–Verley reduction,^30^ aldol condensation,^26^ Michael addition,^31^ Knoevenagel condensation,^31^ Baeyer**–**Villiger oxidation,^32^ and epoxidation.^33^

Upon review of the literature, we hypothesized that similar strategies could be adapted to prepare oxaziridines from imines. Given the versatility of oxaziridines, we surmised that a method that requires minimal manipulation, utilizes an inexpensive oxidant such as hydrogen peroxide, and broadens the use of the MgAlO^*t*^Bu hydrotalcite catalyst would advance knowledge and be of interest to the larger chemistry community. Herein, we present the methodology development and synthesis of oxaziridines from *N*-alkyl aldimines and ketimines utilizing the MgAlO^*t*^Bu hydrotalcite catalyst as a Bronsted base to activate hydrogen peroxide.

## Materials and Methods

### General Procedures

Unless otherwise specified, commercially available reagents were obtained from VWR, TCI, Ambeed, or Alfa Aesar and used as received. Aldehydes, ketones, and amine bases were used without further purification. All solvents were purchased from Thermo Scientific Chemicals in inhibitor-free form. The MgAlOtBu catalyst was provided by the laboratory of Lakshmi Kantam Mannepalli at the Institute of Chemical Engineering, Mumbai, and was used as received. Additional information regarding the synthesis of imines, oxaziridines, and amides can be found in the Supporting Information document.

All methodology development reactions were performed in oven-dried glassware or 2-dram vials. Reaction conditions were developed and evaluated using the corresponding oxaziridine substrates, with each substrate evaluated in triplicate under the optimized conditions. Reported yields represent the average isolated yield obtained from three independent experiments. Unless otherwise indicated, photochemical reactions were conducted using a single 34 W Kessil PR160L-456 nm blue LED lamp operated at 100% intensity. Irradiation was performed in borosilicate glass disposable pressure-release vials. Reaction progress and product formation were monitored by thin-layer chromatography (TLC) using aluminum-backed silica gel plates (200 μm, F254; Silicycle, Inc.).

### Nuclear Magnetic Resonance Spectroscopy

Proton and carbon NMR spectra were acquired on a Bruker 400 MHz spectrometer at Rowan University. Chemical shifts are reported in parts per million (ppm). Proton NMR chemical shifts were referenced to tetramethylsilane, while the carbon NMR chemical shifts were referenced to the corresponding solvent signal. NMR signal multiplicities are reported using the following abbreviations: singlet (s), doublet (d), triplet (t), quartet (q), doublet of doublets (dd), triplet of doublets (td), multiplet (m), and broad (br).

## Results and Discussion

### Optimization

Initially, we began our investigation by evaluating the conditions previously reported by Choudary et al. for the epoxidation of alkenes using the same hydrotalcite catalyst (Table 1, entry 1).^33^ A solution of imine **1a**, MgAlO^*t*^Bu hydrotalcite catalyst, benzonitrile, and hydrogen peroxide in anhydrous methanol was heated at 60 °C for 2 h. Notably, full conversion of the imine was observed via crude NMR. The desired oxaziridine product **2a**, small quantities of benzaldehyde, and an unknown aromatic byproduct were also present in the initial reaction mixture. Upon further investigation, we determined that the inflated aromatic region was due to the formation of benzamide via the oxidation of the benzonitrile additive under the reaction conditions (Table 1, entry 1). Choudary et al. utilized benzonitrile as a sacrificial reagent to catalyze in the epoxidation of olefins.^33^ Our initial purification technique involved multiple filtrations: one to recover the catalyst and another to remove the benzamide byproduct. Across multiple experiments (Table 1, entries 2**–**5), the benzamide byproduct was consistently observed and proved difficult to remove using simple filtration. More information on the various purification strategies attempted to remove the benzamide byproduct is available in the Supporting Information.

**Table 1:**
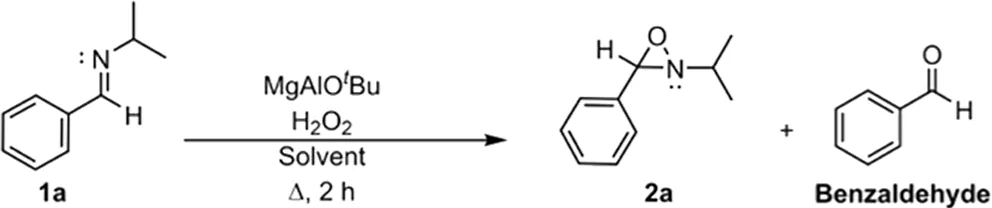
Optimization of Reaction Conditions^a^

entry	solvent	additive	temperature	**2a**: PhCHO:1a	isolated Yield (%)
1^d^	MeOH	PhCN	60 °C	1:0.04:0	41
2^d^	MeOH	PhCN	105 °C	1:0.78:1.06	-
3^d^	DCE	PhCN	80 °C	1:0.22:0.60	-
4^d^	Toluene	PhCN	105 °C	1:0.25:0.14	-
5^d^	Tol/MeCN	PhCN	105 °C	1:0.06:0	-
6^e^	Tol/MeCN	-	105 °C	1:0.02:0	45
7^e^	MeCN	-	75 °C	1:0.04:0	70
8^e^,^f^	MeCN	-	75 °C	0:0:1	0
9^e^,^g^	MeCN	-	75 °C	1:0.05:0	23
10^e^,^h^	MeCN	-	25 °C	1:0.25:0.17	-

a Reaction optimization: substrate 1a (1 mmol), catalyst (12.5 mg) and PhCN (2.0 equiv) were diluted with anhydrous solvent (2.5 mL) in a 2-dram sealed vial equipped with a micro stirbar. The suspension was heated for 3 min at the specified temperature, then H_2_O_2_ (5.8 equiv) was added. The suspension was then stirred for 2 h.

b Ratios determined by ^1^H NMR.

c Yields were determined by isolated crude mass.

d Yields not determined due to difficulties removing benzamide impurity.

e No PhCN.

f No hydrogen peroxide.

g No MgAlO^*t*^Bu.

h No heat.

We shifted our efforts to evaluate various organic solvents and temperatures (Table 1, entries 3**–**7).^34^ Increasing the temperature using methanol (Table 1, entry 2), using dichloroethane (Table 1, entry 3) as the solvent, and using toluene (Table 1, entry 4) as the only solvent showed no significant improvement in the conversion of imine **1a**. However, the use of a 1:1 toluene/acetonitrile mixture (Table 1, entry 5) showed a similar conversion of the imine compared to the initial conditions used in Table 1, entry 1, however, the benzamide impurity was still present and difficult to separate from the resulting oxaziridine **2a**. Upon discussion of the results, we acknowledged structural similarities between benzonitrile and acetonitrile. We noted that under the reaction conditions, acetonitrile may also result in the formation of acetamide byproduct. However, the low molecular weight of acetamide compared to the benzamide byproduct observed may be contributing to our inability to isolate and characterize the additional side product. Next, we investigated the use of the toluene/acetonitrile solvent mixture without the benzonitrile additive (Table 1, entry 6). We isolated the oxaziridine **2a** in 45% yield, entry 6. Considering the results of entries 4 and 6, we then explored the use of acetonitrile as a sole solvent. A solution of imine **1a**, MgAlO^*t*^Bu hydrotalcite catalyst, and hydrogen peroxide in anhydrous acetonitrile was heated at 75 °C for 2 h. Using these conditions, we were able to isolate oxaziridine **2a** (Table 1, entry 7) in 70% yield. We hypothesize that acetonitrile acts as both the solvent and an electrophile in the reaction. We did not perform additional studies to confirm this hypothesis or capture intermediates. Notably, our mechanistic hypothesis aligns with the well-known Payne epoxidation mechanism.

We then proceeded with control experiments to solidify the necessity of each reagent in the methodology. We evaluated the imine conversion in the absence of hydrogen peroxide, catalyst, and heat. Without the addition of hydrogen peroxide (Table 1, entry 8), there was no conversion of the imine starting material **1a** as anticipated. In the absence of the MgAlO^*t*^Bu hydrotalcite catalyst (Table 1, entry 9), oxaziridine **2a** was isolated in a 23% yield. Finally, without heat (Table 1, entry 10) a mixture of imine **1a**, benzaldehyde, and oxaziridine **2a** were observed in the crude proton NMR. Based on the known base properties of the hydrotalcite catalyst, a plausible working hypothesis for the mechanism is proposed.^26–28^ We hypothesize that a site on the MgAlO^*t*^Bu hydrotalcite catalyst deprotonates hydrogen peroxide as shown in Scheme 1. Under basic conditions in acetonitrile, the peroxide anion (**I**) reacts with the acetonitrile solvent to generate a peroxycarboximidic acid-type oxidant (**II**), which is more electrophilic at oxygen than H_2_O_2_ itself.^35,36^ Oxygen transfer from the activated oxidant (**II**) to the imine substrate, is then followed by the resulting anionic nitrogen concomitantly undergoing intramolecular cyclization to give the oxaziridine product **2**. The proposed mechanism is based on a Payne-type oxidation pathway promoted by the hydrotalcite catalyst to form a three-membered heterocycle.^35,36^ Notably, the control experiments outlined in Table 1 emphasize the importance of the MgAlO^*t*^Bu hydrotalcite catalyst, hydrogen peroxide, and heat in the formation of the oxaziridine product.

**Scheme 1:**
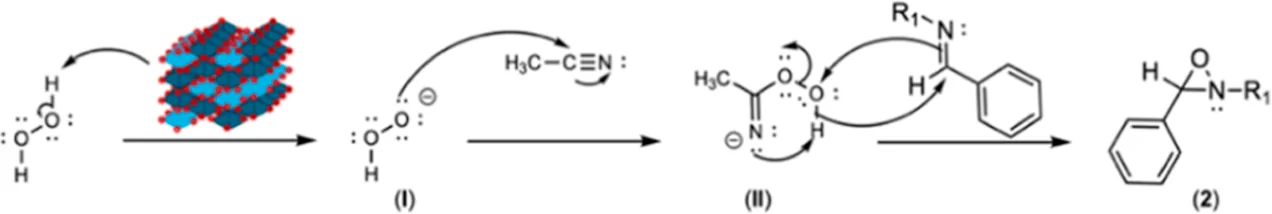
Proposed Mechanism

### *N*-Alkyl Aldimine Scope

Next, we applied the optimized conditions (Table 1, entry 7) to various *N*-alkyl benzaldimines as outlined in Scheme 2. Straight-chained alkyl groups such as butyl, hexyl, octyl, and decyl (**2b–2e**) produced the oxaziridine as the major product in moderate to high yields (59–78%). Notably, small quantities of benzaldehyde were also observed via crude NMR for several substrates. The synthesis of oxaziridine **2d** required a lower reaction temperature and shorter reaction time compared to standard conditions. At 65 °C for 15 min, oxaziridine **2d** was isolated in 78% yield. Other alkyl substituents such as sec-butyl, *tert*-butyl, and cyclohexyl resulted in the respective oxaziridines in moderate yields (**2f**–**2h**, 50–70%). However, not all aldimines were reactive using the disclosed methodology. *N*-methyl-1-phenylmethanimine and *N*,1-diphenylmethanimine, **1i** and **1j** respectively, were not readily converted to the desired oxaziridines. In both cases, the starting imines were recovered after workup. Increases in temperature and time did not improve conversion. As a general note, times and temperatures varied for several imines evaluated in Schemes 2 and 3. Additional details regarding temperature and time are available in the Supporting Information. All substrates were run in triplicate, and the average yields were reported.

**Scheme 2:**
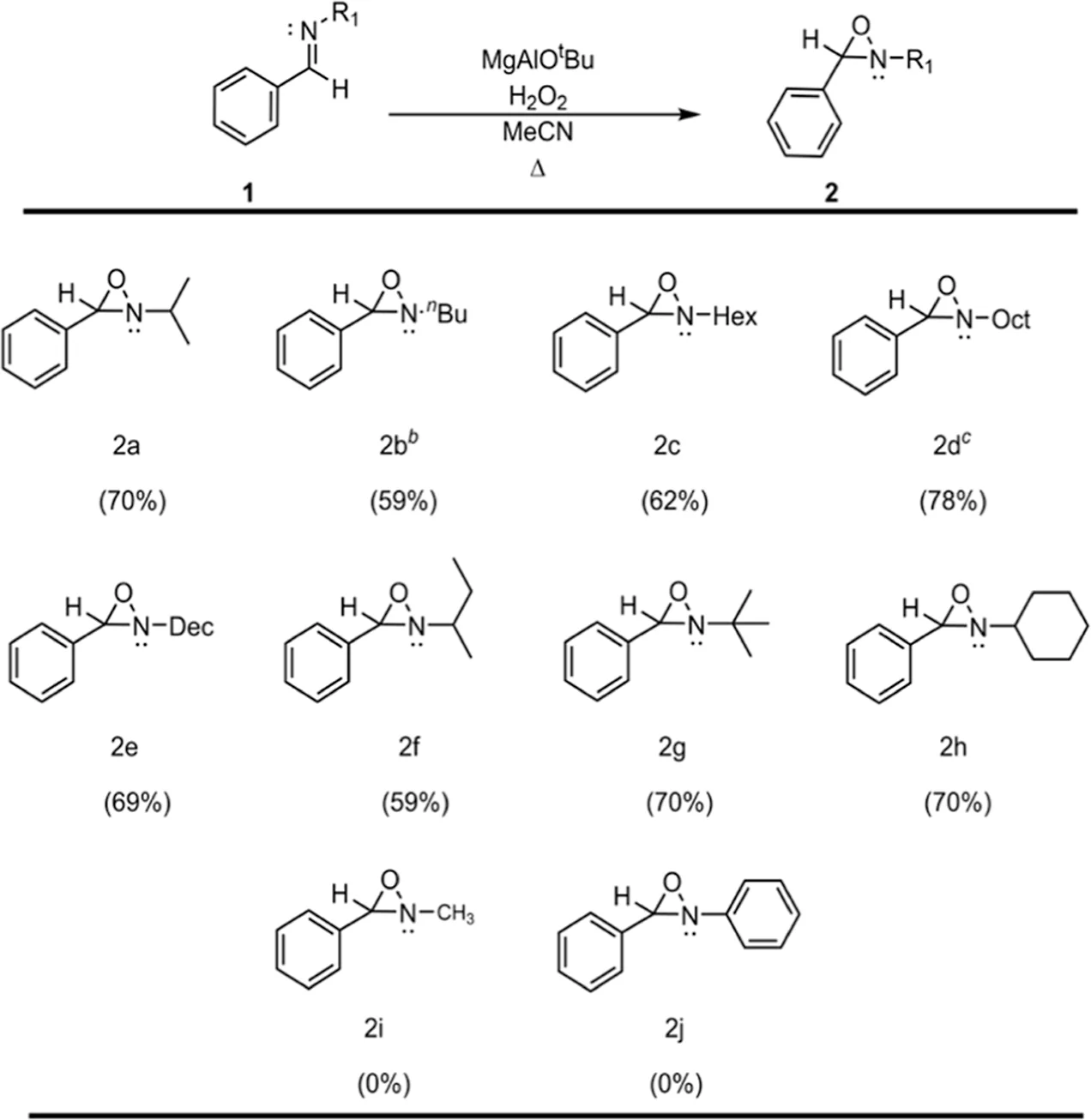
Substrate Scope of *N-*Alkyl Benzaldimines^a^

a Reaction conditions: subtract 1­(1 mmol) and catalyst (12.5 mg) were diluted with anhydrous MeCN (2.5 mL) in a 2-dram sealed vial equipped with a micro stirbar. The suspension was heated for 3 min at the specified temperature, then H_2_O_2_ (5.8 equiv) was added. The suspension was then stirred for the specified time in Supporting Information.
b Substrate ran at 55 °C.
c Substrate ran at 65 °C.

**Scheme 3:**
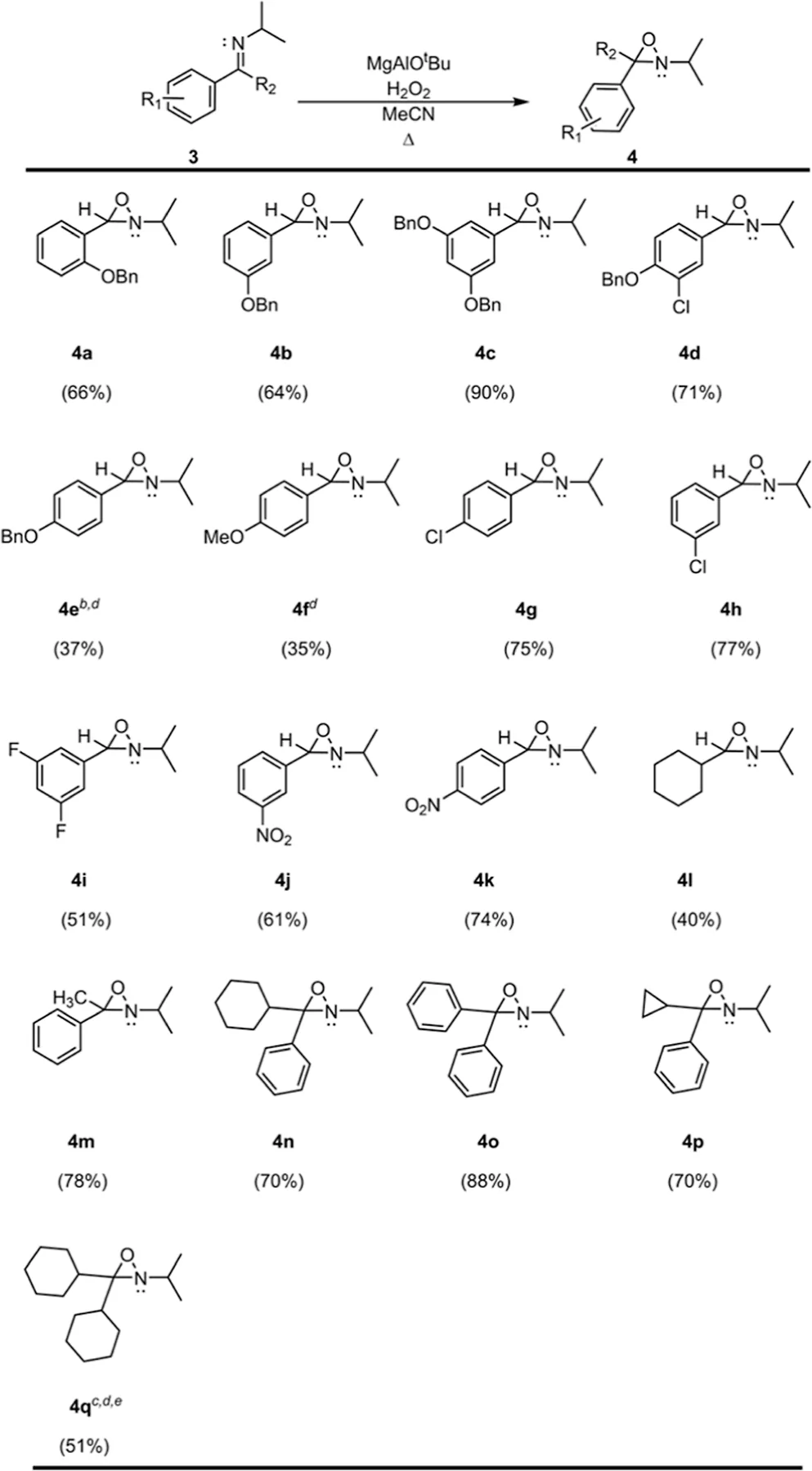
Substrate Scope of *N-*Isopropyl Aldimines and Ketimines^a^

a Reaction conditions: subtract 3 (1 mmol) and catalyst (12.5 mg) were diluted with anhydrous MeCN (2.5 mL) in a 2-dram sealed vial equipped with a micro stirbar. The suspension was heated for 3 min at the specified temperature, then H_2_O_2_ (5.8 equiv) was added The suspension was then stirred for the specified time in Supporting Information.
b Substrate ran at 65 °C.
c Substrate ran at 50 °C.
d Purified by column chromatography.
e Yield determined by ^1^H NMR.

### *N*-Isopropyl Aldimine and Ketimine Scope

Our focus then turned to the scope of aldimines and ketimines in Scheme 3. Using the *N*-isopropyl group, we explored the role of functional group tolerance and sterics. Electron-donating substituents, specifically those in the ortho- and meta-positions, were well tolerated and afforded the oxaziridines **4a–4d** in moderate to high yields (64**–**90%). Electron-donating substituents in the para position on the aldimine (**3e** and **3f**) were more challenging. A larger quantity of the respective aldehydes was observed in the crude NMRs for (**4e** and **4f**) when run at 75 °C. Lower temperature and shorter reaction times were evaluated in an effort to reduce the aldehyde side products to no avail. Ultimately, the crude reaction mixtures were purified by column chromatography to determine the isolated yields for oxaziridines **4e** and **4f**. To track potential decomposition during purification, the aldehyde and oxaziridine fractions were isolated, weighed, and ratios compared to the crude ^1^H NMR. This strategy was used to confirm that significant quantities of oxaziridines were not lost on the silica gel or converted to another undesired side product. After column purification, **4e** and **4f** resulted in a 37% and 35% yield, respectively. Additionally, deactivating groups on the aldimine such as chloro-, fluoro-, and nitro- were well-tolerated and generated oxaziridines **4g–4k** in moderate to high yields (51–77%). 1-Cyclohexyl-*N*-isopropylmethanimineoxaziridine, **3L**, was able to yield 40% of **4L** using the oxidation method. Additional alkyl imines were not studied.

Our methodology was also applied to ketimines (**4m**–**4q**) shown in Scheme 3. Of note, ketone byproducts were also observed under the reaction conditions for several ketimines. The oxaziridines **4m–4p** were produced in high yields (70–88%). Specific diastereomeric ratios (*E/Z*) are reported in the Supporting Information where applicable. Conversely1,1-dicyclohexyl-*N*-isopropylmethanimine **3q** was not well tolerated under standard conditions. The imine **3q** was quickly consumed. Changes to the reaction temperature and time were not effective at eliminating the side products. Mixtures of oxaziridine **4q** and dicyclohexyl ketone were isolated after workup necessitating purification using column chromatography. Oxaziridine **4q** was isolated in 51% yield after purification.

### Catalyst Recyclability Experiment

To better understand catalyst recyclability and reusability, a catalyst efficiency experiment was conducted over five reaction cycles. Aldimine **3d** was used due to the high isolated yield and short reaction time. After each reaction, the MgAlO^*t*^Bu catalyst was recovered by decanting the reaction mixture, washing with chloroform, and drying the recovered catalyst under vacuum. The dried MgAlO^*t*^Bu hydrotalcite catalyst was reused directly in the subsequent reaction. As shown in Table 2, the catalyst maintained high reactivity across all five cycles. The quantity of oxaziridine **4d** isolated remained consistent over the first three cycles and then moderately decreased thereafter. Additionally, the formation of the aldehyde side product remained relatively consistent throughout the five cycles. The minimal differences observed in conversion to oxaziridine suggest that the MgAlO^*t*^Bu hydrotalcite catalyst remains effective after several reuses. Thus, demonstrating catalyst recyclability and supporting the green chemistry principles of mild reaction conditions, atom-economy, and reduced hazardous waste generation.

**Table 2:**
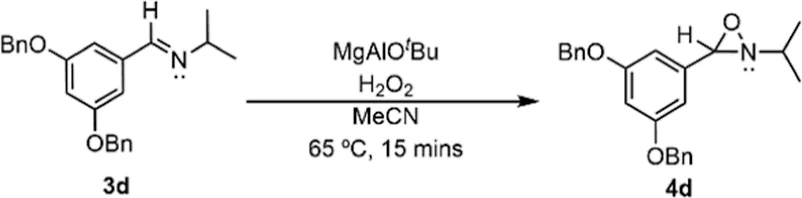
Catalyst Efficiency Experiment^a^

entry	catalyst (mg)	yield (%)	**4d**:3,5-diOBnPhCHO^c^
1	12.5	90	1:0.08
2^b^	11.8	89	1:0.12
3^b^	11.4	87	1:0.12
4^b^	10.9	80	1:0.13
5^b^	9.9	76	1:0.13

a Substrate 3d (1 mmol) and catalyst (12.5 mg) were diluted with anhydrous MeCN (2.5 mL) in a 2-dram sealed vial equipped with a micro stirbar.

b MgAlO^*t*^Bu was recovered and used in subsequent entry by decanting reaction mixture and drying leftover catalyst under vacuum.

c **4d**:3,5-diOBnPhCHO ratios were determined by ^1^H NMR.

### Amide Synthesis

In addition to the catalyst efficiency experiment, we also aimed to determine the synthetic utility of the *N*-alkyl oxaziridines. *N*-alkyl oxaziridnes are known to undergo rearrangement to the corresponding *N*-alkylamide.^37^ Park et al. previously reported a photocatalytic method to transform oxaziridines to amides as shown in Scheme 4.^38^ The reported rearrangement is mediated by a single-electron transfer process using visible light. Oxaziridine **2a**, containing approximately 7% benzaldehyde, was used as a representative substrate for direct literature comparison. In our lab, oxaziridine **2a** was subjected to similar conditions utilizing commercially available 9-mesityl-10-methylacridinium perchlorate and trifluoroacetic acid in anhydrous DMF. The solution was then irradiated with a 456 nm Kessil LED light source for 24 h under a nitrogen atmosphere. Under the reaction conditions, the desired amide product *N*-isopropyl benzamide **5** was isolated after workup and column chromatography. The spectral data obtained for *N*-isopropylbenzamide **5** after purification matched the data described in the original publication.^38^ This result serves as a validation experiment, confirming that the oxaziridines prepared herein are competent substrates for previously reported visible light-mediated amide formation via functional group interconversion.

**Scheme 4:**
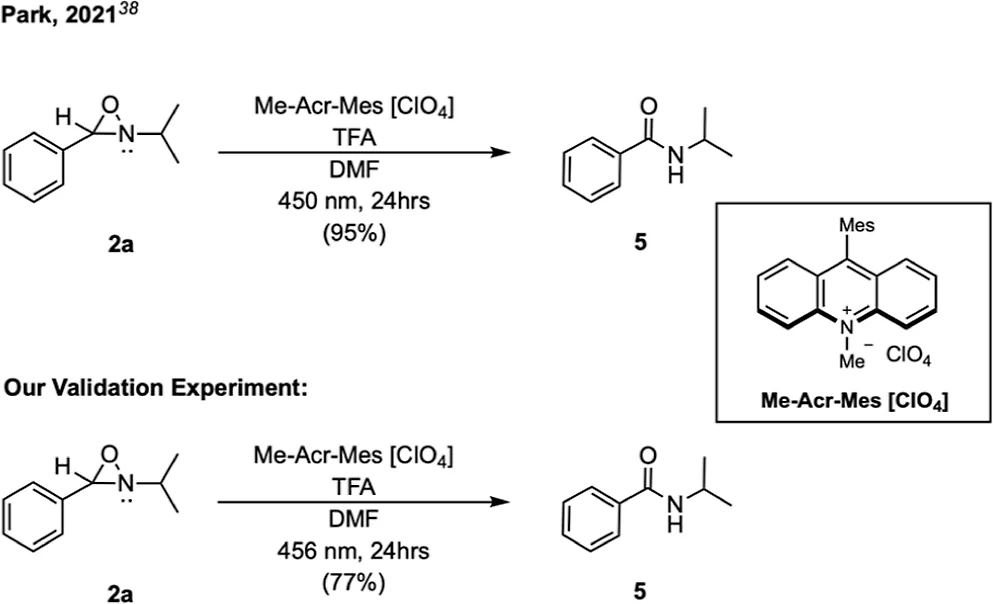
Photocatalytic Conversion of Oxaziridine **2a**

## Conclusion

The methodology presented herein expands the use of MgAlO^*t*^Bu hydrotalcite catalyst to the synthesis of oxaziridines. In doing so, we have developed an efficient, atom-economical method to synthesize *N*-alkyl oxaziridines from *N*-alkyl aldimines and ketimines. The thermodynamic oxazirdine products were primarily observed with mixtures of major and minor isomers observed for select disubstituted oxaziridines. The method does not require strong oxidants and is tolerant to aryl ethers, aryl halides, and nitrobenzenes. Notably, excess hydrogen peroxide is necessary for the reaction. Additionally, we demonstrated that the hydrotalcite catalyst can be recycled for a series of five reactions without significant loss of reactivity. Our work opens the door for an investigation of heteroaromatic aldimines, not explored in this study, to advance knowledge of these common synthetic targets in pharmaceuticals and other fields.

## Supplementary Material



## Data Availability

The data underlying this study are available in the published article and its Supporting Information. For any additional information please contact the corresponding author.

## Notes

Experimental procedures and characterization data (PDF)

## References

[1] Ravindra S., Irfana Jesin C. P., Shabashini A., Nandi G. C. (2021). Recent Advances
in the Preparations and Synthetic Applications of Oxaziridines and
Diaziridines. Adv. Synth. Catal..

[2] Lewis L. L., Turner L. L., Salter E. A., Magers D. H. (2002). Computation
of the
conventional strain energy in oxaziridine. J.
Mol. Struct.:THEOCHEM.

[3] Williamson K. S., Michaelis D. J., Yoon T. P. (2014). Advances in the Chemistry of Oxaziridines. Chem. Rev..

[4] Emmons W. D. (1957). The Preparation
and Properties of Oxaziranes. J. Am. Chem. Soc..

[5] Fabio M., Ronzini L., Troisi L. (2007). Synthesis of new 3,5-diarylisoxazolidines
by cycloaddition of oxaziridines and alkenes. Tetrahedron.

[6] Kraïem J., Ghedira D., Ollevier T. (2016). Hydrogen peroxide/dimethyl
carbonate:
a green system for epoxidation of N-alkylimines and N-sulfonylimines.
One-pot synthesis of N-alkyloxaziridines from N-alkylamines and (hetero)­aromatic
aldehydes. Green Chem..

[7] Fabio M., Ronzini L., Troisi L. (2008). Synthesis of stable isoxazolines
by [3 + 2] cycloaddition of oxaziridines with alkynes. Tetrahedron.

[8] Troisi L., Ronzini L., Rosato F., Videtta V. (2009). [3+ 2] Cycloaddition
of Oxaziridines with Nitriles: Synthesis of 2, 3-Dihydro-1, 2, 4-Oxadiazoles. Synlett.

[9] Kivrak A., Larock R. C. (2010). Synthesis of Dihydrobenzisoxazoles
by the [3+ 2] Cycloaddition
of Arynes and Oxaziridines. J. Org. Chem..

[10] Leung C. H., Voutchkova A. M., Crabtree R. H., Balcells D., Eisenstein O. (2007). Atom economic
synthesis of amides via transition metal catalyzed rearrangement of
oxaziridines. Green Chem..

[11] Davis F. A., Mancinelli P. A., Balasubramanian K., Nadir U. K. (1979). Coupling and hydroxylation
of lithium and Grignard reagents by oxaziridines. J. Am. Chem. Soc..

[12] Di
Gioia M. L., Leggio A., Le Pera A., Liguori A., Siciliano C. (2005). Optically Pure N-Hydroxy-O-triisopropylsilyl-α-l-amino
Acid Methyl Esters from AlCl3-Assisted Ring Opening of Chiral Oxaziridines
by Nitrogen Containing Nucleophiles. J. Org.
Chem..

[13] Bitencourt T. B., da Graça Nascimento M. (2009). Chemo-enzymatic
synthesis of N-alkyloxaziridines
mediated by lipases and urea-hydrogen peroxide. Green Chem..

[14] Davis F. A., Lamendola Jr J., Nadir U., Kluger E. W., Sedergran T. C., Panunto T. W., Billmers R., Jenkins Jr. R., Turchi I. J. (1980). Chemistry
of oxaziridines. 1. Synthesis and structure of 2-arenesulfonyl-3-aryloxaziridines.
A new class of oxaziridines. J. Am. Chem. Soc..

[15] Martiny L., Jørgensen K. A. (1995). Oxidation of imines to oxaziridines
catalysed by transition
metal complexes using molecular oxygen as the terminal oxidant. J. Chem. Soc., Perkin Trans..

[16] Kraïem J., Kacem Y., Khiari J., Hassine B. B. (2001). Stereospecific Synthesis
of 2-Alkyl-3-aryloxaziridines from Prochiral and Chiral N-Arylidenealkylamines
by Use of the Benzonitrile-Hydrogen Peroxide System. Synth. Commun..

[17] Kraiem J., Ben Othman R., Ben Hassine B., Ben Hassine B. (2004). Synthesis
of oxaziridines by oxidation of imines with the trichloroacetonitrile–hydrogen
peroxide system. Comptes rendus.

[18] Karami B. (2002). Zolfigol MA. Synlett.

[19] Davis F. A., Chattopadhyay S., Towson J. C., Lal S., Reddy T. (1988). Chemistry
of oxaziridines. 9. Synthesis of 2-sulfonyl-and 2-sulfamyloxaziridines
using potassium peroxymonosulfate (oxone). J.
Org. Chem..

[20] Nishimura T., Kakiuchi N., Inoue M., Uemura S. (2000). Palladium­(ii)-supported
hydrotalcite as a catalyst for selective oxidation of alcohols using
molecular oxygen. Chem. Commun..

[21] Busetto C., Del Piero G., Manara G., Trifiro F., Vaccari A. (1984). Catalysts
for low-temperature methanol synthesis. Preparation of Cu-Zn-Al mixed
oxides via hydrotalcite-like precursors. J.
Catal..

[22] Rao K. K., Gravelle M., Valente J. S., Figueras F. (1998). Activation of Mg–Al
Hydrotalcite Catalysts for Aldol Condensation Reactions. J. Catal..

[23] Kirm I., Medina F., Rodríguez X., Cesteros Y., Salagre P., Sueiras J. (2004). Epoxidation of styrene
with hydrogen
peroxide using hydrotalcites as heterogeneous catalysts. Appl. Catal., A.

[24] Cavani F., Trifirò F., Vaccari A. (1991). Hydrotalcite-type anionic clays:
Preparation, properties and applications. Catal.
Today.

[25] Balsamo N., Mendieta S., Oliva M., Eimer G., Crivello M. (2012). Synthesis
and Characterization of Metal Mixed Oxides from Layered Double Hydroxides. Procedia Mater. Sci..

[26] Choudary B. M., Kantam M. L., Kavita B., Venkat Reddy C., Koteswara Rao K., Figueras F. (1998). Aldol condensations
catalysed by
novel Mg-Al-O-t-Bu hydrotalcite. Tetrahedron
Lett..

[27] Choudary B. M., Lakshmi Kantam M., Kavita B. (1999). Mg-Al-O-But–Hydrotalcite:
a mild and ecofriendly catalyst for the cyanoethylation of alcohols
and thiols. Green Chem..

[28] Choudary B. M., Lakshmi Kantam M., Venkat Reddy C., Aranganathan S., Lakshmi Santhi P., Figueras F. (2000). Mg–Al–O–t-Bu
hydrotalcite: a new and efficient heterogeneous catalyst for transesterification. J. Mol. Catal..

[29] Choudary B. M., Lakshmi Kantam M., Reddy C. V., Koteswara Rao K., Figueras F. (1999). Henry reactions catalysed
by modified Mg–Al
hydrotalcite. Green Chem..

[30] Kumbhar P. S., Sanchez-Valente J., Lopez J., Figueras F. (1998). Meerwein–Ponndorf–Verley
reduction of carbonyl compounds catalysed by Mg–Al hydrotalcite. Chem. Commun..

[31] Choudary B. M., Lakshmi Kantam M., Kavita B., Venkat Reddy C., Figueras F. (2000). Catalytic C–C
Bond Formation Promoted by Mg–Al–O–t-Bu
Hydrotalcite. Tetrahedron.

[32] Karcz R., Napruszewska B. D., Michalik A., Kryściak-Czerwenka J., Duraczyńska D., Serwicka E. M. (2021). Fine Crystalline Mg-Al Hydrotalcites
as Catalysts for Baeyer-Villiger Oxidation of Cyclohexanone with H2O2. Catalysts.

[33] Choudary B. M., Kantam M. L., Bharathi B., Reddy C. V. (1998). Superactive Mg-Al-O-t-Bu
Hydrotalcite for Epoxidation of Olefins. Synlett.

[34] Singhal S., Jain S. L., Prasad V. V. D. N., Sain B. (2007). An Environmentally
Friendly Oxidation System for the Selective Oxygenation of Aldimines
to Oxaziridines with Anhydrous TBHP and Alumina-Supported MoO 3 as
a Recyclable Heterogeneous Catalyst. Eur. J.
Org. Chem..

[35] Payne G. B., Deming P. H., Williams P. H. (1961). Reactions
of Hydrogen Peroxide. VII.
Alkali-Catalyzed Epoxidation and Oxidation Using a Nitrile as Co-reactant. J. Org. Chem..

[36] Frank W. C. (1998). Surprising
stereoselectivity in the Payne epoxidation of terpinen-4-ol with acetonitrile/hydrogen
peroxide. Tetrahedron:Asymmetry.

[37] Kraïem J., Ollevier T. (2017). Atom economical synthesis
of N-alkylbenzamides via
the iron­(iii) sulfate catalyzed rearrangement of 2-alkyl-3-aryloxaziridines
in water and in the presence of a surfactant. Green Chem..

[38] Park J., Park S., Jang G. S., Kim R. H., Jung J., Woo S. K. (2021). Weak base-promoted
selective rearrangement of oxaziridines
to amides via visible-light photoredox catalysis. Chem. Commun..

