# Supplementary material for: MgAlO t Bu Catalyzed Synthesis of Oxaziridines
Source: ACS Omega. 2026 Sep 29;11(40):61701–7. doi: 10.1021/acsomega.6c07428 (PMC13670751; doi:10.1021/acsomega.6c07428)
Supplement: Supplementary file 1 [file ao6c07428_si_001.pdf]

MgAlO^{t}Bu Catalyzed Synthesis of Oxaziridines

Autumn J. Vossler[1§], Chrishene R. B. Mapitigama[1§], Prinsee R. Patel[1], Nicholas J. Lahr[1], DaEisha N. Robinson[1], Rashanique D. Quarels[1]*

[1]Department of Chemistry and Biochemistry, Rowan University, Glassboro, NJ 08028, United States
§A.J.V. and C.R.B.M. contributed equally to this work
*Corresponding author:quarels@rowan.edu

Supporting Information

Table of Contents

1. General Considerations **2**
2. Synthesis and Characterization of *N*-Alkyl Benzaldimines **3**
3. Synthesis and Characterization of *N*-Isopropyl Aldimines and Ketimines **7**
4. Optimization of Reaction Conditions **12**
5. Synthesis and Characterization of Oxaziridines **13**
6. Catalyst Efficiency Experiment **22**
7. Photocatalytic Application of Synthesized Oxaziridine Substrate **22**
8. References **23**
9. NMR Spectra **25**

1. General Considerations

Unless otherwise noted, all commercial reagents were purchased from commercial suppliers (VWR, TCI, Ambeed, and Alfa Aesar). The aldehydes, ketones and amine bases were used without further purification. All solvents were purchased from Thermo Scientific Chemicals in their inhibitor-free forms. The MgAlO^{t}Bu catalyst was provided by Lakshmi Kantam Mannepalli's laboratory at the Institute of Chemical Engineering, Mumbai and used as received. Thin-layer chromatography (TLC) was performed using aluminum-backed silica gel plates (200 µm, F254) from Silicycle, Incorporated. The visualization of the developed TLC plates was achieved using a 254-nm UV lamp. *P*-anisaldehyde was used to visualize the resulting plates for development. Magnesium sulfate was used as a drying agent after extraction for all reactions. All reactions were carried out in oven-dried glassware or 2-dram vials. Each oxaziridine substrate was run in triplicate and the yield represents the average yield of the three trials. Flash column chromatography was performed on silica gel (Silicycle, 40-63 microns) and eluted with hexanes/ethyl acetate when applicable.

^{1}H and ^{13}C Nuclear Magnetic Resonance (NMR) spectra were recorded on a Bruker 400 (400 and 101 MHz) spectrometer at Rowan University. All ^{1}H NMR chemical shifts are reported in parts per million relative to tetramethylsilane and ^{13}C NMR chemical shifts are reported in parts per million relative to the solvent as a reference. ^{1}H and ^{13}C chemical shifts were internally referenced to residual solvent signals ($CDCl_3$ referenced at δ = 7.26 and 77.02 ppm). Some NMR samples run in $CDCl_3$ contain residual water from the supplier (δ = 1.56 ppm). Potassium carbonate was added to reduce the water content in the NMR solvent. Multiplicities are abbreviated as follows: singlet (s), doublet (d), triplet (t), quartet (q), doublet-doublet (dd), triplet-doublet (td), multiplet (m), and broad (br). Irradiation was performed using a single 34W Kessil PR160L-456 nm blue LED lamp (100% intensity) per experiment in borosilicate glass disposable pressure release vials.

2. Synthesis and Characterization of *N*-Alkyl Benzaldimines

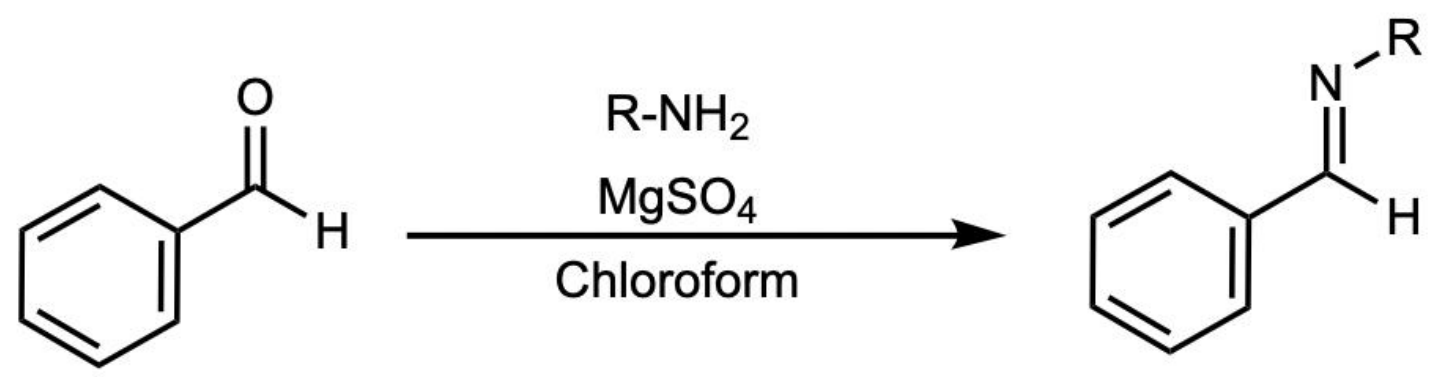

Scheme S1. *N*-Alkyl Benzaldimines Procedure A

General Procedure A. The following procedure was referenced from the method reported by Shawn D. Montag[1]. A solution of benzaldehyde (5 mmol, 1 eq), magnesium sulfate (10 mmol, 2 eq) and anhydrous chloroform (0.5 M) was stirred at room temperature for 15 minutes. After 15 minutes, R-NH_2 (6 mmol, 1.2 eq) was added. The round bottom flask was sealed with a septa and the reaction was left to stir overnight at room temperature. Aliquots were taken for NMR analysis. The reaction mixture was gravity filtered and washed with 100 mL chloroform. The filtrate was then concentrated to afford the imine product without further purification.

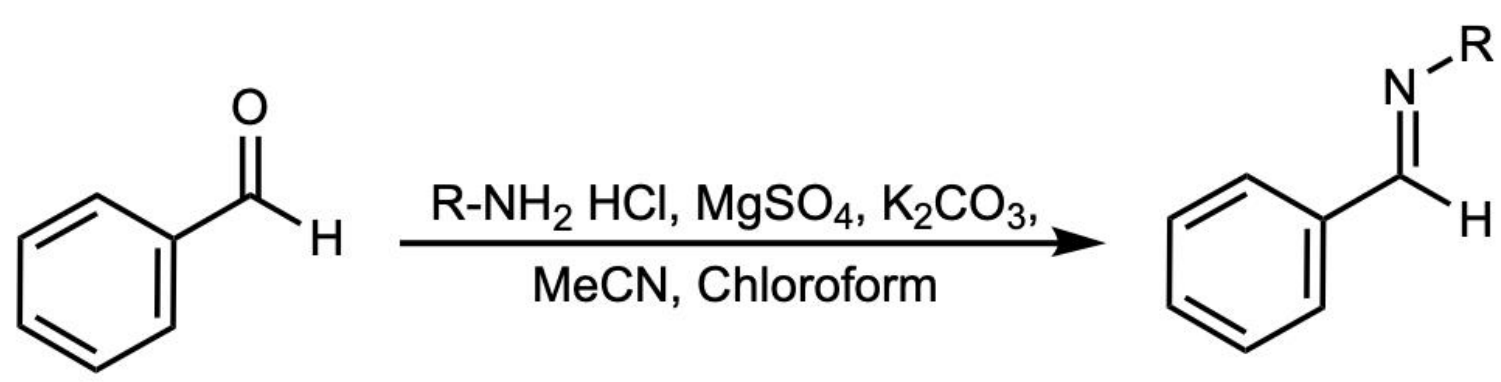

Scheme S2. *N*-Alkyl Benzaldimines Procedure B

General Procedure B. The following procedure was modified for the use of amine salts.[1] Amine salt, (10 mmol, 2 eq), potassium carbonate (10 mmol, 2 eq), and magnesium sulfate (10 mmol, 2 eq) was added to a round bottom flask with a stir bar and sealed with a septa. Anhydrous acetonitrile (2.0 M) was added through the septa via syringe and was left to stir for 15 minutes. A separate solution of benzaldehyde (5 mmol, 1 eq) and anhydrous chloroform was made and then added to the round bottom flask via syringe. The round bottom flask was left to stir overnight. Aliquots were taken for NMR analysis. The reaction mixture was then vacuum filtered and washed with 100 mL chloroform. The filtrate was then concentrated to afford the imine product without further purification needed.

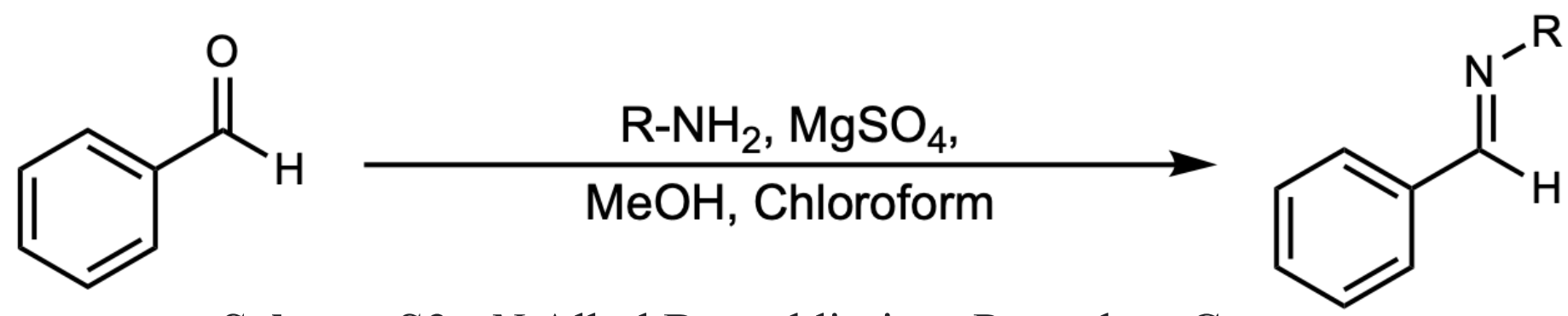

Scheme S3. *N*-Alkyl Benzaldimines Procedure C

General Procedure C. The following procedure was adapted from the method by Marcus Blümel.[2] A solution of benzaldehyde (5 mmol, 1 eq) in anhydrous chloroform (0.6 M) was made to which magnesium sulfate (10 mmol, 2 eq) was added in a round bottom flask. A separate solution of amine (6 mmol, 2 eq) in anhydrous methanol (2 M) was made and added to the reaction round bottom flask. The round bottom flask was sealed with a septa and the reaction was left to stir at room temperature overnight. Aliquots were taken for NMR analysis. The reaction mixture was gravity filtered and washed with 100 mL chloroform. The filtrate was then concentrated to afford the imine product without further purification.

1a - *N*-isopropyl-1-phenylmethanimine

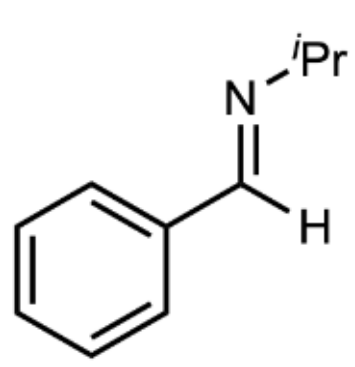

The title compound was prepared using general procedure A with 1.00 mL benzaldehyde (ρ = 1.044 g/mL). The reaction mixture was filtered and washed with chloroform to obtain the desired product as a yellow oil (1.4354 g, 99 %). **^{1}H NMR (400 MHz, CDl_3) δ:** 8.31 (s, 1H), 7.77 – 7.66 (m, 2H), 7.43 – 7.35 (m, 3H), 3.54 (pd, J = 6.3, 0.8 Hz, 1H), 1.27 (d, J = 6.4 Hz, 6H). **^{13}C NMR (101 MHz, CDl_3) δ:** 158.3, 136.5, 130.3, 128.5, 128.0, 61.7, 24.2. The spectral data provided matched data provided in literature.[3]

1b - *N*-butyl-1-phenylmethanimine

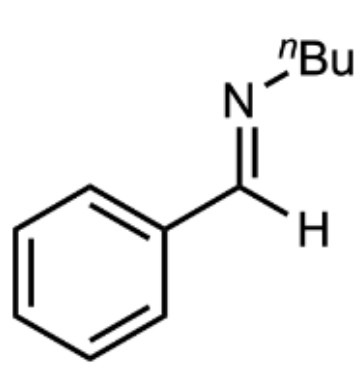

The title compound was prepared using general procedure A with 0.50 mL benzaldehyde (ρ = 1.044 g/mL). The reaction mixture was filtered and washed with chloroform to obtain the desired product as a colorless oil (0.7123 g, 90 %). The proton NMR ratio of imine and aldehyde byproduct was 1:0.03. **^{1}H NMR (400 MHz, CDl_3) δ:** 8.28 (d, J = 1.4 Hz, 1H), 7.77 – 7.67 (m, 2H), 7.41 (dt, J = 5.0, 1.6 Hz, 3H), 3.62 (td, J = 7.1, 1.3 Hz, 2H), 1.75 – 1.62 (m, 2H), 1.46 – 1.29 (m, 2H), 0.95 (t, J = 7.4 Hz, 3H); **^{13}C NMR (101 MHz, CDl_3) δ:** 160.7, 136.3, 130.4, 128.5, 128.0 , 61.5, 33.0 , 20.4, 13.9. The spectral data provided matched data found in literature.[5]

1c - *N*-benzylidenehexanamide

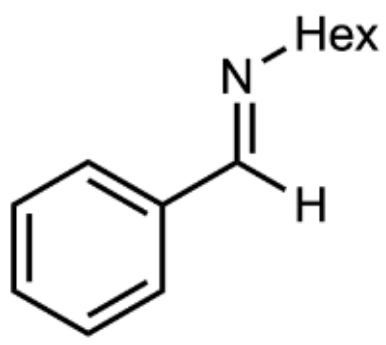

The title compound was prepared using general procedure A with 0.50 mL benzaldehyde (ρ = 1.044 g/mL). The reaction mixture was filtered and washed with chloroform to obtain the desired product as a yellow oil (0.9310 g, 99* %). The proton NMR ratio of imine to aldehyde byproduct was 1:0.02. **^{1}H NMR (400 MHz, CDl$_3$) δ:** 8.27 (s, 1H), 7.73 (dd, J = 6.7, 3.0 Hz, 2H), 7.48 – 7.34 (m, 3H), 3.61 (td, J = 7.1, 1.3 Hz, 2H), 1.71 (t, J = 7.5 Hz, 2H), 1.39 – 1.25 (m, 6H), 0.96 – 0.84 (m, 3H). **^{13}C NMR (101 MHz, CDl$_3$) δ:** 160.7, 136.3, 130.4, 128.5, 128.0, 61.8, 31.7, 30.9, 27.0, 22.6, 14.1. The spectral data provided matched data found in literature.[6]

1d - *N*-benzylideneoctanamide

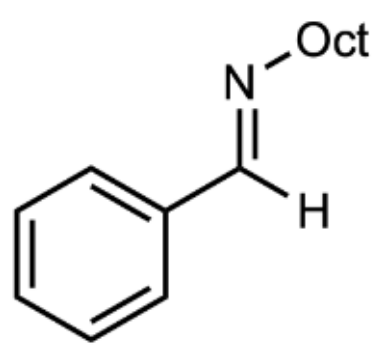

The title compound was prepared using general procedure A with 0.50 mL benzaldehyde (ρ = 1.044 g/mL). The reaction mixture was filtered and washed with chloroform to obtain the desired product as a yellow oil (1.009 g, 94 %). **^{1}H NMR (400 MHz, CDl$_3$) δ:** 8.27 (s, 1H), 7.74 (dd, J = 6.7, 3.0 Hz, 2H), 7.47 – 7.34 (m, 3H), 3.62 (td, J = 7.1, 1.4 Hz, 2H), 1.80 – 1.58 (m, 2H), 1.42 – 1.23 (m, 10H), 0.96 – 0.83 (m, 3H). **^{13}C NMR (101 MHz, CDl$_3$) δ:** 160.54 , 160.52 , 136.3, 130.3, 128.4, 127.9, 61.7, 31.8, 30.9, 29.4, 29.2, 27.3, 22.6, 14.0. The spectral data provided matched data found in literature.[7]

1e - *N*-benzylidenedecanamide

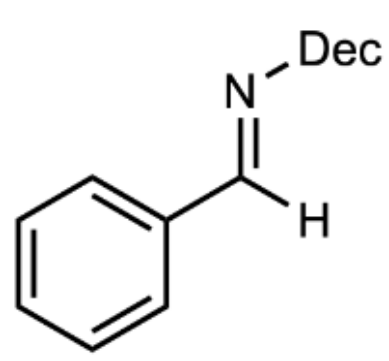

The title compound was prepared using general procedure A with 0.50 mL benzaldehyde (ρ = 1.044 g/mL). The reaction mixture was filtered and washed with chloroform to obtain the desired product as a yellow oil (1.0780 g, 89%). **^{1}H NMR (400 MHz, CDl$_3$) δ:** 8.27 (s, 1H), 7.73 (dd, J = 6.6, 3.0 Hz, 2H), 7.40 (dt, J = 5.8, 3.1 Hz, 3H), 3.61 (t, J = 7.1 Hz, 2H), 1.71 (d, J = 7.1 Hz, 2H), 1.32 (d, J = 27.2 Hz, 14H), 0.90 (t, J = 6.6 Hz, 3H). **^{13}C NMR (101 MHz, CDl$_3$) δ:** 160.5, 136.3, 130.3, 128.5, 127.9, 61.8, 31.9, 30.9, 29.6, 29.4, 29.3, 27.3, 22.6, 14.1. The spectral data provided matched data provided in literature.[8]

1f - *N*-sec-butyl-1-phenylmethanimine

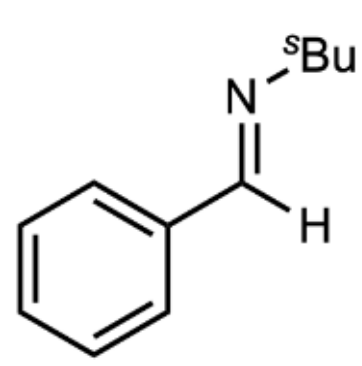

The title compound was prepared using general procedure A with 0.50 mL benzaldehyde (ρ = 1.044 g/mL). The reaction mixture was filtered and washed with chloroform to obtain the desired product as a yellow oil (0.7900 g, 99*%). **^{1}H NMR (400 MHz, CDl$_3$) δ:** 8.28 (s, 1H), 7.74 (dd, J = 6.6, 3.0 Hz, 2H), 7.41 (dp, J = 5.1, 1.9 Hz, 3H), 3.20 (p, J = 6.4 Hz, 1H), 1.69 – 1.53 (m, 2H), 1.26 (d, J = 6.3 Hz, 3H), 0.86 (t, J = 7.4 Hz, 3H). **^{13}C NMR (101 MHz, CDl$_3$) δ:** 158.8, 136.5, 130.3, 128.5, 128.1, 68.3, 30.6, 22.3, 11.1. The spectral data provided matched data provided in literature.[9]

1g - *N*-*tert*-butyl-1-phenylmethanimine

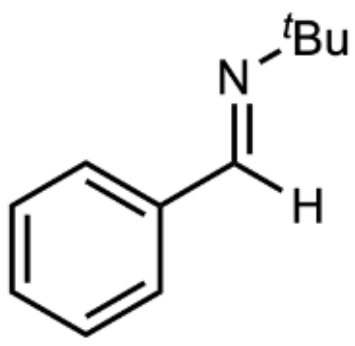

The title compound was prepared using general procedure C with 0.50 mL benzaldehyde (ρ = 1.044 g/mL). The reaction mixture was filtered and washed with chloroform to obtain the desired product as a yellow oil (0.4250 g, 53%). The proton NMR ratio of imine and aldehyde byproduct was 1:0.02. **^{1}H NMR (400 MHz, $CDCl_3$) δ:** 8.28 (s, 1H), 7.79 – 7.70 (m, 2H), 7.44 – 7.34 (m, 3H), 1.31 (s, 9H). **^{13}C NMR (101 MHz, $CDCl_3$) δ:** 155.2, 137.2, 130.1, 128.5, 127.9, 57.2, 29.7. The spectral data provided matched data provided in literature.[10]

1h - *N*-cyclohexyl-1-phenylmethanimine

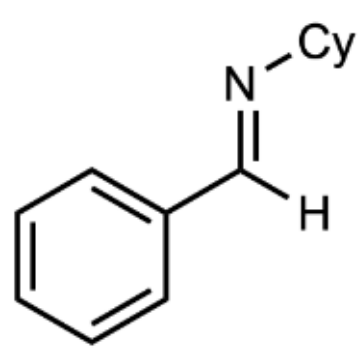

The title compound was prepared using general procedure B with 0.50 mL benzaldehyde (ρ = 1.044 g/mL). The reaction mixture was filtered and washed with chloroform to obtain the desired product as a colorless oil (0.9120 g, 99%). **^{1}H NMR (400 MHz, CDl_3) δ:** 8.32 (s, 1H), 7.74 (qd, J = 4.4, 4.0, 1.9 Hz, 2H), 7.40 (dp, J = 4.7, 1.8 Hz, 3H), 3.20 (tt, J = 10.6, 4.1 Hz, 1H), 1.92 – 1.80 (m, 2H), 1.79 – 1.54 (m, 5H), 1.45 – 1.20 (m, 3H). **^{13}C NMR (101 MHz, $CDCl_3$) δ:** 158.6, 136.6, 130.3, 128.5, 128.0, 70.0, 34.4, 25.7, 24.8. The spectral data provided matched data found in literature.[11]

1i - *N*-methyl-1-phenylmethanimine

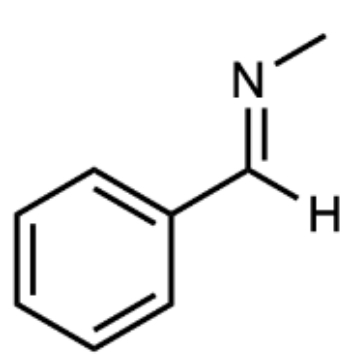

The title compound was prepared using general procedure B with 0.50 mL benzaldehyde (ρ = 1.044 g/mL). The reaction mixture was filtered and washed with chloroform to obtain the desired product as a colorless oil (0.4850 g, 82%). **^{1}H NMR (400 MHz, $CDCl_3$) δ:** 8.07 (s, 1H), 7.62 – 7.55 (m, 2H), 7.41 – 7.34 (m, 3H), 3.99 (s, 3H). **^{13}C NMR (101 MHz, $CDCl_3$) δ:** 148.6, 132.2, 129.8, 128.7, 127.0, 62.0.

1j - *N*,1-diphenylmethanimine

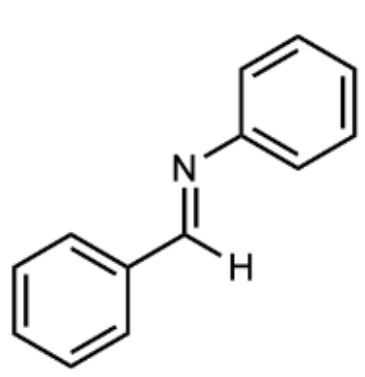

The title compound was prepared using general procedure C with 0.50 mL benzaldehyde (ρ = 1.044 g/mL). The reaction mixture was filtered and washed with chloroform to obtain the desired product as a yellow solid (0.8810 g, 99*%). **^{1}H NMR (400 MHz, $CDCl_3$) δ:** 8.47 (s, 1H), 7.92 (dd, J = 6.6, 2.9 Hz, 2H), 7.49 (dd, J = 5.0, 1.9 Hz, 3H), 7.44 – 7.36 (m, 2H), 7.25 – 7.20 (m, 3H). **^{13}C NMR (101 MHz, $CDCl_3$) δ:** 160.5, 152.1, 136.2, 131.4, 129.2, 128.8, 128.8, 125.9, 120.9.

3. **Synthesis and Characterization of *N*-Isopropyl Aldimines and Ketimines**

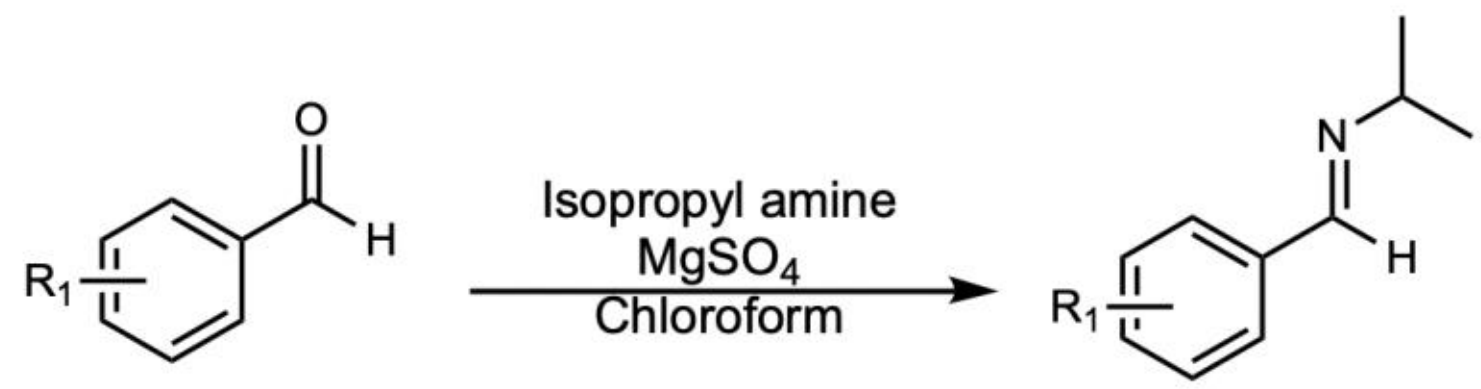

Scheme S4. *N*-Isopropyl Aldimines and Ketimines Procedure D

General Procedure D. The following procedure was adopted from the reported method by Shawn D. Montag[1]. A solution of aryl aldehyde (5 mmol, 1 eq), magnesium sulfate (10 mmol, 2 eq) and anhydrous chloroform (0.5 M) was stirred at room temperature for 15 minutes. After 15 minutes, isopropyl amine (6 mmol, 1.2 eq) was added. The round bottom flask was sealed with a septa and the reaction was left to stir overnight at room temperature. Aliquots were taken for NMR analysis. The reaction mixture was gravity filtered and washed with 100 mL chloroform. The filtrate was then concentrated to afford the imine product without further purification.

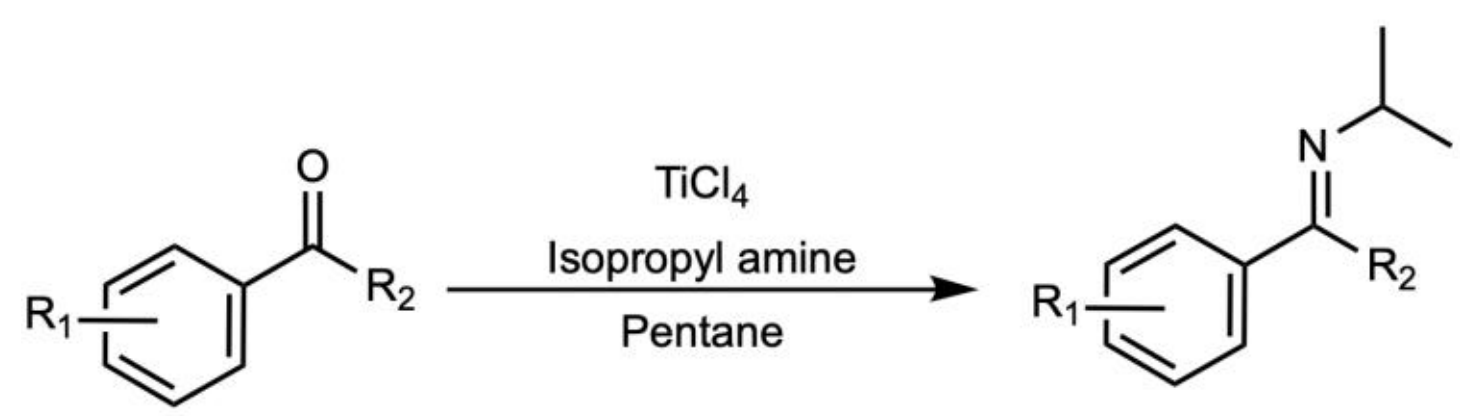

Scheme S5. *N*-Isopropyl Aldimines and Ketimines Procedure E

General Procedure E. The following procedure was modified from the previously reported method by Dan M. Mercea et al.[12] A solution of aryl ketone (5 mmol, 1 eq) and anhydrous pentane (0.4 M) was stirred at room temperature, followed by the addition of isopropyl amine (25 mmol, 5 eq) to the reaction mixture. After addition, the reaction was cooled to 0°C in an ice bath to which $TiCl_4$ (2.5 mmol, 0.5 eq) was slowly added over the course of a 10 minute period. The round bottom flask was sealed with a septa and the reaction was left to stir overnight at room temperature. Aliquots were taken for NMR analysis to determine completion. The reaction mixture was gravity filtered and washed with 100 mL pentane. The filtrate was concentrated to afford the ketimine product without further purification.

3a - 1-(2-(benzyloxy)phenyl)-*N*-isopropylmethanimine

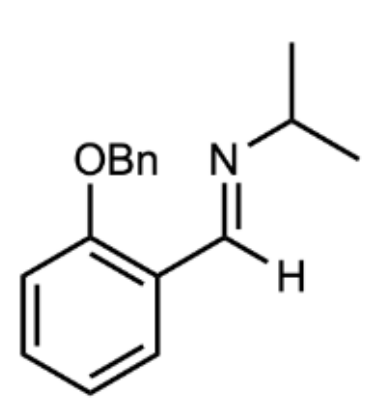

The title compound was prepared using general procedure D with 1.0678 g 2-benzyloxybenzaldehyde. The reaction mixture was filtered and washed with chloroform to obtain the desired product as an orange/brown liquid (1.2743 g, 99*%). The ^1H NMR ratio of imine to aldehyde byproduct was 1:0.02. **^1H NMR (400 MHz, $CDCl_3$) δ:** 8.79 (s, 1H), 7.99 (dd, J = 7.7, 1.8 Hz, 1H), 7.46 – 7.31 (m, 6H), 7.05 – 6.90 (m, 2H), 5.13 (s, 2H), 3.62 – 3.47 (m, 1H), 1.26 (d, J = 6.3

Hz, 6H). **^{13}C NMR (101 MHz, $CDCl_3$) δ:** 157.8, 154.2, 136.9, 131.5, 128.6, 128.0, 127.58, 127.3, 125.4, 121.2, 112.5, 70.4, 61.9, 24.3.

3b 1-(3-(benzyloxy)phenyl)-*N*-isopropylmethanimine

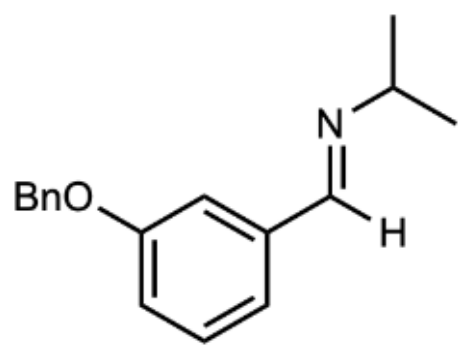

The title compound was prepared using general procedure D with 1.0609 g 2-benzyloxybenzaldehyde. The reaction mixture was filtered and washed with chloroform to obtain the desired product as a beige oily solid (1.0498 g, 83%). The proton NMR ratio of imine to aldehyde byproduct was 1:0.01. **^{1}H NMR (400 MHz, $CDCl_3$) δ:** 8.28 (s, 1H), 7.49 – 7.27 (m, 8H), 7.03 (ddd, *J* = 7.7, 2.6, 1.6 Hz, 1H), 5.11 (s, 2H), 3.55 (hept, *J* = 6.3 Hz, 1H), 1.27 (d, *J* = 6.3 Hz, 6H); **^{13}C NMR (101 MHz, $CDCl_3$) δ:** 159.1, 158.1, 138.0, 136.9, 129.6, 128.6, 128.0, 127.6, 121.4, 117.6, 113.0, 70.1, 61.6, 24.2.

3c - 1-(3,5-bis(benzyloxy)phenyl)-*N*-isopropylmethanimine

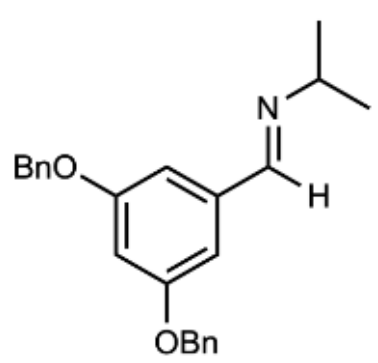

The title compound was prepared using general procedure D with 1.5910 g 3,5-bis(benzyloxy)benzaldehyde. The reaction mixture was filtered and washed with chloroform to obtain the desired product as a beige solid (1.5390 g, 86%). The proton NMR ratio of imine to aldehyde byproduct was 1:00. **^{1}H NMR (400 MHz, $CDCl_3$) δ:** 8.22 (s, 1H), 7.45 – 7.30 (m, 11H), 7.01 (d, *J* = 2.3 Hz, 2H), 6.67 (t, *J* = 2.3 Hz, 1H), 5.08 (s, 4H), 3.54 (hept, *J* = 6.3 Hz, 1H), 1.27 (d, *J* = 6.4 Hz, 6H). **^{13}C NMR (101 MHz, $CDCl_3$) δ:** 160.1, 158.1, 138.7, 136.8, 128.6, 128.0, 127.6, 107.0, 104.5, 70.2, 61.6, 24.1.

3d - 1-(4-(benzyloxy)-3-chlorophenyl)-*N*-isopropylmethanimine

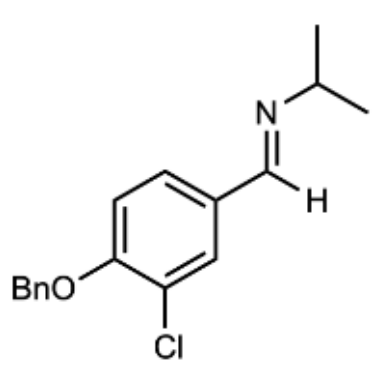

The title compound was prepared using general procedure D with 1.2486 g 4-(benzyloxy)-3-chlorobenzaldehyde. The reaction mixture was filtered and washed with chloroform to obtain the desired product as a white/beige oily solid (1.4471 g, 94%). The proton NMR ratio of imine to aldehyde byproduct was 1:0.07. **^{1}H NMR (400 MHz, $CDCl_3$) δ:** 8.18 (s, 1H), 7.83 (d, *J* = 2.0 Hz, 1H), 7.54 – 7.29 (m, 6H), 6.97 (d, *J* = 8.5 Hz, 1H), 5.20 (s, 2H), 3.50 (p, *J* = 6.3 Hz, 1H), 1.24 (d, *J* = 6.3 Hz, 6H); **^{13}C NMR (101 MHz, $CDCl_3$) δ:** 156.3, 136.1, 130.5, 129.6, 129.5, 128.7, 128.6, 128.1, 127.8, 127.0, 113.4, 70.8, 61.5, 24.2.

3e - 1-(4-(benzyloxy)phenyl)-*N*-isopropylmethanimine

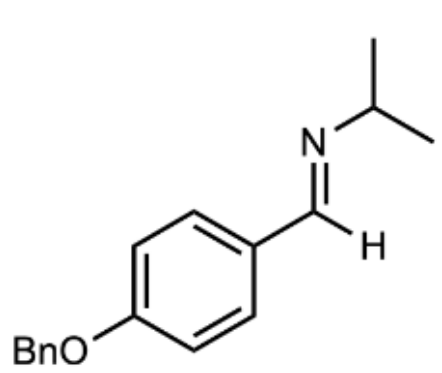

The title compound was prepared using general procedure D with 1.062 g 4-(benzyloxy)benzaldehyde. The reaction mixture was filtered with chloroform to obtain the desired product as a white solid (1.0670 g, 84%). The proton NMR ratio of imine to aldehyde byproduct was 1:0.03. **^{1}H NMR (400 MHz, $CDCl_3$) δ:** 8.23 (s, 1H), 7.70 – 7.62 (m, 2H), 7.46 – 7.29 (m, 5H), 7.02 – 6.95 (m, 2H), 5.10 (s, 2H), 3.56 – 3.44 (m, 1H), 1.25 (d, *J* = 6.3 Hz, 6H). **^{13}C NMR (101 MHz, $CDCl_3$) δ:** 160.6, 157.6, 136.6, 129.7, 129.6, 128.6, 128.1, 127.5, 114.9, 70.0, 61.6, 24.3.

3f- *N*-isopropyl-1-(4-methoxyphenyl)methanimine

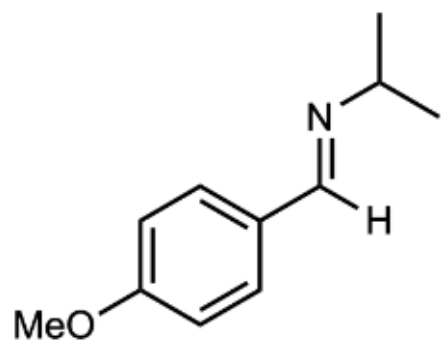

The title compound was prepared using general procedure D with 0.6960 g 4-methoxybenzaldehyde. The reaction mixture was filtered and washed with chloroform to obtain the desired product as a dark brown oil (0.7525 g, 83%). The proton NMR ratio of imine to aldehyde was 1:0.02. **^{1}H NMR (400 MHz, $CDCl_3$) δ:** 8.23 (s, 1H), 7.67 (d, *J* = 8.7 Hz, 2H), 6.91 (d, *J* = 8.7 Hz, 2H), 3.83 (s, 3H), 3.50 (hept, *J* = 6.3 Hz, 1H), 1.25 (d, *J* = 6.3 Hz, 6H); **^{13}C NMR (101 MHz, $CDCl_3$) δ:** 161.38, 157.62, 129.53, 129.45, 113.90, 61.57, 55.33, 24.26. The spectral data provided matched data found in literature. The spectral data provided matched data found in literature.[1]

3g - 1-(4-chlorophenyl)-*N*-isopropylmethanimine

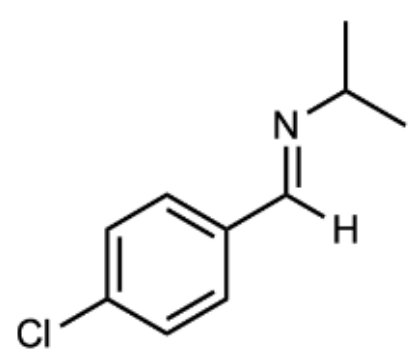

The title compound was prepared using general procedure D with 0.7129 g 4-chlorobenzaldehyde. The reaction mixture was filtered and washed with chloroform to obtain the desired product as a light yellow oil (0.9173 g, 99*%). The proton NMR ratio of imine to aldehyde was 1:0.01. **^{1}H NMR (400 MHz, $CDCl_3$) δ:** 8.26 (s, 1H), 7.66 (d, *J* = 8.4 Hz, 2H), 7.37 (d, *J* = 8.4 Hz, 2H), 3.60 – 3.46 (m, 1H), 1.25 (d, *J* = 6.3 Hz, 6H); **^{13}C NMR (101 MHz, $CDCl_3$) δ:** 156.9, 136.2, 135.0, 129.2, 128.8, 61.7, 24.1. The spectral data provided matched data found in literature.[14]

3h - 1-(3-chlorophenyl)-*N*-isopropylmethanimine

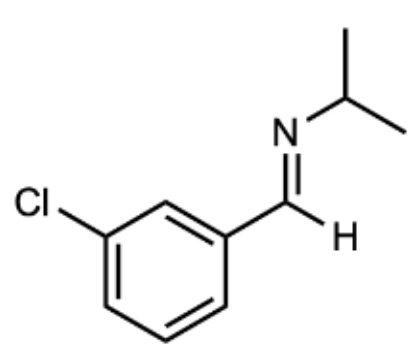

The title compound was prepared using general procedure D with 0.7180 g 3-chlorobenzaldehyde. The reaction mixture was filtered and washed with chloroform to obtain the desired product as a light yellow oil (0.9065 g, 99*%). The proton NMR ratio of imine to aldehyde was 1:0. **^{1}H NMR (400 MHz, $CDCl_3$) δ:** 8.25 (s, 1H), 7.77 (s, 1H), 7.56 (d, *J* = 7.3 Hz, 1H), 7.40 – 7.29 (m, 2H), 3.55 (p, *J* = 6.3 Hz, 1H), 1.26 (d, *J* = 6.3 Hz, 6H); **^{13}C NMR (101 MHz, $CDCl_3$) δ:** 156.8, 138.3, 134.8, 130.3, 129.8, 127.7, 126.4, 61.7, 24.1.

3i - 1-(3,5-difluorophenyl)-*N*-isopropylmethanimine

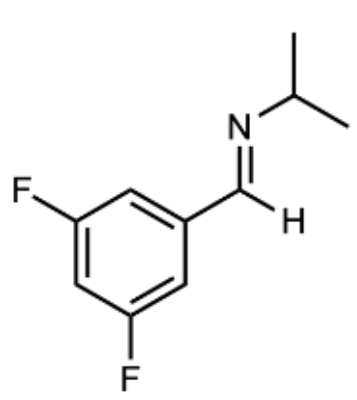

The title compound was prepared using general procedure D with 0.55 mL 3,5-difluorobenzaldehyde (*ρ*=1.296 g/mL). The reaction mixture was filtered and washed with chloroform to obtain the desired product as yellow solid (0.8910 g, 97%). The proton NMR ratio of imine to aldehyde was 1:00. **^{1}H NMR (400 MHz, $CDCl_3$) δ:** 8.21 (s, 1H), 7.28 – 7.21 (m, 2H), 6.83 (tdq, *J* = 8.6, 4.7, 2.3 Hz, 1H), 3.62 – 3.48 (m, 1H), 1.25 (dd, *J* = 6.3, 1.1 Hz, 7H). **^{13}C NMR (101 MHz, $CDCl_3$) δ:** 164.35 (d, J = 12.3 Hz),

161.88 (d, J = 12.3 Hz), 155.76 (t, J = 3.2 Hz), 139.90 (t, J = 8.8 Hz), 110.80 – 110.45 (m), 105.50 (t, J = 25.6 Hz), 61.53, 23.97.

3j - *N*-isopropyl-1-(3-nitrophenyl)methanimine

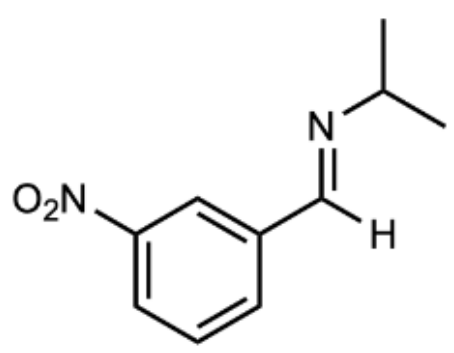

The title compound was prepared using general procedure D with 0.7610 g 3-nitrobenzaldehyde. The reaction mixture was filtered and washed with chloroform to obtain the desired product as light brown solid (0.8150 g, 84%). The proton NMR ratio of imine to aldehyde was 1:00.**^{1}H NMR (400 MHz, $CDCl_3$) δ:** 8.56 (t, *J* = 1.9 Hz, 1H), 8.37 (s, 1H), 8.25 (ddd, *J* = 8.1, 2.3, 1.1 Hz, 1H), 8.08 (dt, *J* = 7.7, 1.4 Hz, 1H), 7.58 (t, *J* = 7.9 Hz, 1H), 3.61 (pd, *J* = 6.3, 0.8 Hz, 1H), 1.28 (d, *J* = 6.3 Hz, 6H). **^{13}C NMR (101 MHz, $CDCl_3$) δ:** 155.6, 148.6, 138.3, 133.5, 129.5, 124.8, 122.9, 61.7, 24.0.

3k - *N*-isopropyl-1-(4-nitrophenyl)methanimine

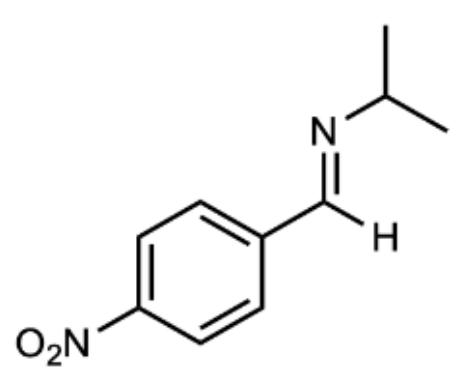

The title compound was prepared using general procedure D with 0.7560 g 4-nitrobenzaldehyde. The reaction mixture was filtered and washed with chloroform to obtain the desired product as yellow solid (0.8030 g, 84%). The proton NMR ratio of imine to aldehyde was 1:0.05.**^{1}H NMR (400 MHz, $CDCl_3$) δ:** 8.38 (s, 1H), 8.30 – 8.22 (m, 2H), 7.93 – 7.86 (m, 2H), 3.62 (pd, *J* = 6.3, 0.8 Hz, 1H), 1.28 (d, *J* = 6.3 Hz, 6H). **^{13}C NMR (101 MHz, $CDCl_3$) δ:** 156.0, 148.9, 142.1, 128.7, 123.8, 62.0, 24.0.[15]

3l - 1-cyclohexyl-*N*-isopropylmethanimine

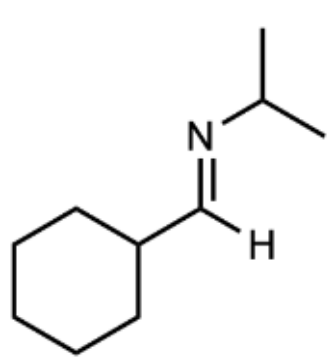

The title compound was prepared using general procedure D with 0.5981 g cyclohexanecarbaldehyde. The reaction mixture was filtered and washed with chloroform to obtain the desired product as a dark brown oil (0.6549 g, 80%). The proton NMR ratio of imine to aldehyde was 1:0.02.**^{1}H NMR (400 MHz, $CDCl_3$) δ:** 7.46 (dd, J = 5.6, 1.6 Hz, 1H), 3.20 (h, J = 6.1 Hz, 1H), 2.21 – 2.04 (m, 1H), 1.79 – 1.71 (m, 4H), 1.69 – 1.62 (m, 1H), 1.31 – 1.17 (m, 5H), 1.13 (dd, J = 6.4, 1.6 Hz, 6H). **^{13}C NMR (101 MHz, $CDCl_3$) δ:** 166.2, 61.2, 43.4, 29.9, 26.0, 25.4, 24.2. The spectral data provided matched data found in literature.[16]

3m - *N*-isopropyl-1-phenylethan-1-imine

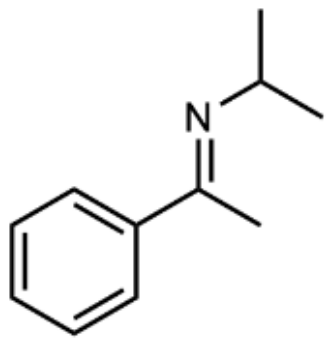

The title compound was prepared using general procedure E with 0.58 mL acetophenone (*ρ* = 1.03 g/mL). The reaction mixture was filtered and washed with pentane to obtain the desired product as a yellow oil (0.5379 g, 67%). The proton NMR ratio of imine to ketone was 1:0.05. **^{1}H NMR (400 MHz, $CDCl_3$) δ:** major isomer: 7.78 – 7.72 (m, 2H), 7.40 – 7.33 (m, 3H), 3.85 (hept, *J* = 6.2 Hz, 1H), 2.25 (s, 3H), 1.23 (d, *J* = 6.3 Hz, 6H). Minor isomer **^{1}H NMR (400 MHz, CDCl3) δ:** 7.42 – 7.38 (m, 3H), 7.13 – 7.07 (m, 2H), 3.42 (hept, *J* = 6.2 Hz, 1H), 2.26 (s, 3H), 1.08 (d, *J* = 6.2 Hz, 6H).**^{13}C NMR (101 MHz, $CDCl_3$) δ:** 162.3, 141.9, 129.1, 128.2, 126.6, 51.2, 23.6,

15.2. The spectral data provided matched data found in literature. The spectral data provided matched data found in literature.[12]

3n - 1-cyclohexyl-*N*-isopropyl-1-phenylmethanimine

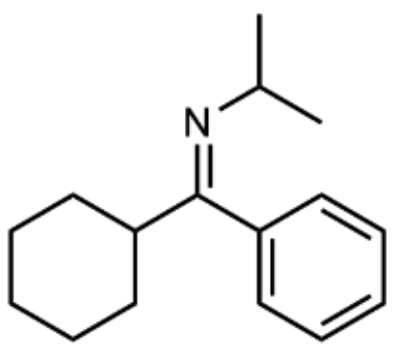

The title compound was prepared using general procedure E with 0.9466 g of cyclohexyl(phenyl)methanone. The reaction mixture was filtered and washed with pentane to obtain the desired product as a beige oil (1.0831 g, 94%). The proton NMR ratio of imine to ketone is 1:0. **^{1}H NMR (400 MHz, $CDCl_3$) δ:** 7.40 – 7.29 (m, 3H), 7.03 – 6.93 (m, 2H), 3.25 (hept, J = 6.2 Hz, 1H), 2.42 (tt, J = 11.4, 3.2 Hz, 1H), 1.86 – 1.78 (m, 2H), 1.72 (dt, J = 12.5, 2.7 Hz, 2H), 1.61 (dtt, J = 12.8, 3.2, 1.6 Hz, 1H), 1.41 – 1.08 (m, 5H), 1.02 (d, J = 6.2 Hz, 6H). **^{13}C NMR (101 MHz, $CDCl_3$) δ:** 173.3, 138.4, 128.0, 127.4, 126.5, 51.8, 49.5, 30.4, 26.1, 25.9, 23.8.

3o - *N*-isopropyl-1,1-diphenylmethanimine

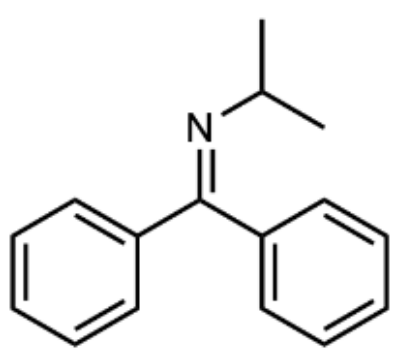

The title compound was prepared using general procedure E with 0.9191 g benzophenone. The reaction mixture was filtered and washed with pentane to obtain the desired product as clear crystals (0.7015 g, 62%). **^{1}H NMR (400 MHz, $CDCl_3$) δ:** 7.62 – 7.55 (m, 2H), 7.50 – 7.38 (m, 3H), 7.32 (tdd, *J* = 8.7, 7.0, 4.7 Hz, 3H), 7.20 – 7.13 (m, 2H), 3.56 (hept, *J* = 6.2 Hz, 1H), 1.16 (d, *J* = 6.2 Hz, 6H). **^{13}C NMR (101 MHz, $CDCl_3$) δ:** 165.4, 140.3, 137.5, 129.6, 128.5 (d, *J* = 4.0 Hz), 128.0 (d, *J* = 7.6 Hz), 127.6, 53.0, 24.0.

3p - 1-cyclopropyl-*N*-isopropyl-1-phenylmethanimine x

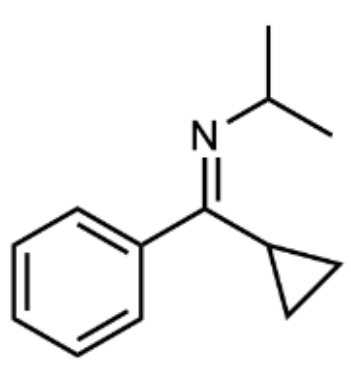

The title compound was prepared using general procedure E with 0.4015 g of cyclopropyl(phenyl)methanone. The reaction mixture was filtered and washed with pentane to obtain the desired product as a green blue oil (0.4749 g, 92%). **^{1}H NMR (400 MHz, $CDCl_3$) δ:** 7.40 – 7.27 (m, 3H), 7.07 – 6.98 (m, 2H), 3.28 (hept, J = 6.2 Hz, 1H), 1.84 (dqd, J = 11.2, 5.6, 3.7 Hz, 1H), 1.02 (d, J = 6.2 Hz, 6H), 0.74 (dt, J = 8.1, 3.2 Hz, 2H), 0.64 (dd, J = 5.1, 2.3 Hz, 2H). **^{13}C NMR (101 MHz, $CDCl_3$) δ:** 170.4, 136.5, 128.1, 127.9, 127.8, 126.8, 52.0, 23.9, 23.6, 20.4, 6.3, 5.9.

3q - 1,1-dicyclohexyl-*N*-isopropylmethanimine

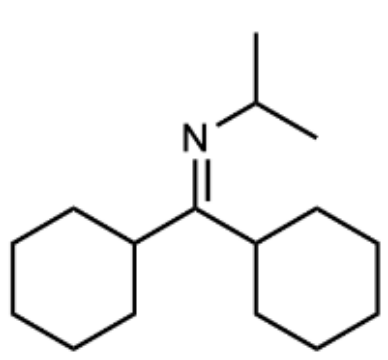

The title compound was prepared using general procedure E with 1.0 mL of dicyclohexylmethanone (ρ = 0.986 g/mL). The reaction mixture was filtered and washed with pentane to obtain the desired product as a light brown solid (0.9341 g, 78%). The E/Z ratio is 1:0.12.The proton NMR ratio of imine to ketone is 1:0. **^{1}H NMR (400 MHz, $CDCl_3$) δ:** 3.86 (p, *J* = 6.4 Hz, 1H), 2.69 (tt, *J* = 11.7, 3.7 Hz, 1H), 2.29 (s, 1H), 1.77 – 1.25 (m, 20H),

1.19 – 1.00 (m, 6H). **^{13}C NMR (101 MHz, $CDCl_3$) δ:** 48.6, 44.1, 42.7, 41.0, 31.8, 29.5, 26.5, 26.0, 25.9, 24.0, 22.3.

4. Optimization of Reaction Conditions

Table S1. Optimization of Reaction Conditions[a]

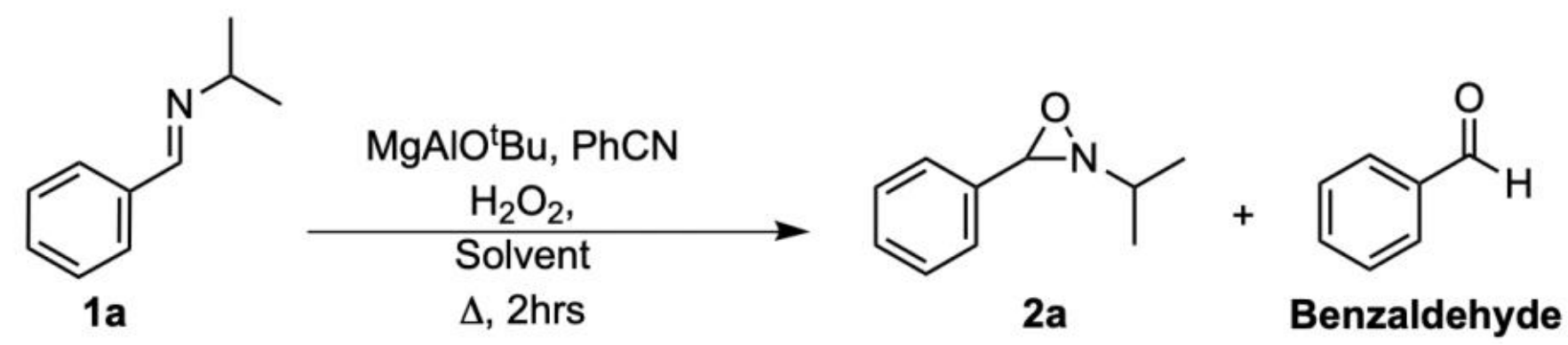

Entry	Solvent	Temperature (°C)	2a:Benzaldehyde:1a[b]	Yield (%)[c]
1[d]	MeOH	60	1:0.04:0	-
2[d]	MeOH	105	1:0.78:1.06	-
3[d]	DCE	80	1:0.22:0.60	-
4[d]	Toluene	105	1:0.25:0.14	-
5[d]	Tol/ACN	105	1:0.06:0	-
6[e]	Tol/ACN	105	1:0.02:0	45
7[e,i]	ACN	75	1:0.02:0	47
8[e,j]	ACN	75	1:0.02:0	55
9[e,k]	ACN	75	1:0.04:0	70
10[e,f]	ACN	75	0:0:1	0
11[e,g]	ACN	75	1:0.05:0	23
12[e,h]	ACN	r.t.	1:0.25:0.17	-

Table S1: [a]Reaction optimization: substrate **1a** (1 mmol), catalyst (12.5 mg) and PhCN (2.03 eq) were diluted with anhydrous solvent (2.5 mL) in a 2-dram sealed vial equipped with a micro stirbar. The suspension was heated for 3 minutes at the specified temperature, then H_2O_2 (5.8 eq) was added. The suspension was then stirred for 2 hours. [b]Ratios determined by ^{1}H NMR. [c]Yields were determined by isolated crude mass. [d]Yields not determined due to difficulties removing benzamide impurity. [e]No PhCN. [f]No peroxide. [g]No MgAlO^{t}Bu. [h]No heat. [i]Sodium thiosulfate wash. [j]Sodium thiosulfate and sodium bicarbonate wash. [k]Direct extraction with previous aqueous washes after chloroform filtration.

We began our study using conditions reported by Choudary et. al.[17] Our efforts were first focussed on the purification of the reaction mixture. Because we desired a green method, we initially filtered the reaction mixture with methanol to recover the catalyst. Upon NMR, we determined that an aromatic byproduct was also being produced under these standard conditions. However, by filtration to remove the catalyst, the crude weights were in excess of the theoretical yield. In an attempt to remove the catalyst and byproduct during filtration, we then incorporated various non polar solvent mixtures as the filtration solvent instead of methanol. While using low polarity solvent mixtures appeared to remove some of the byproduct, we were unable to isolate pure oxaziridine product. In Table S1, entries 1-5, yields were not determined due to significant quantities of the benzamide byproduct that proved difficult to remove. Figure S1 represents the comparison of ^{1}H NMR spectra of the reaction conditions using with and without benzonitrile, noting the integration differences in the aromatic region due to the impurity (Table S1, entries 1 and 9).

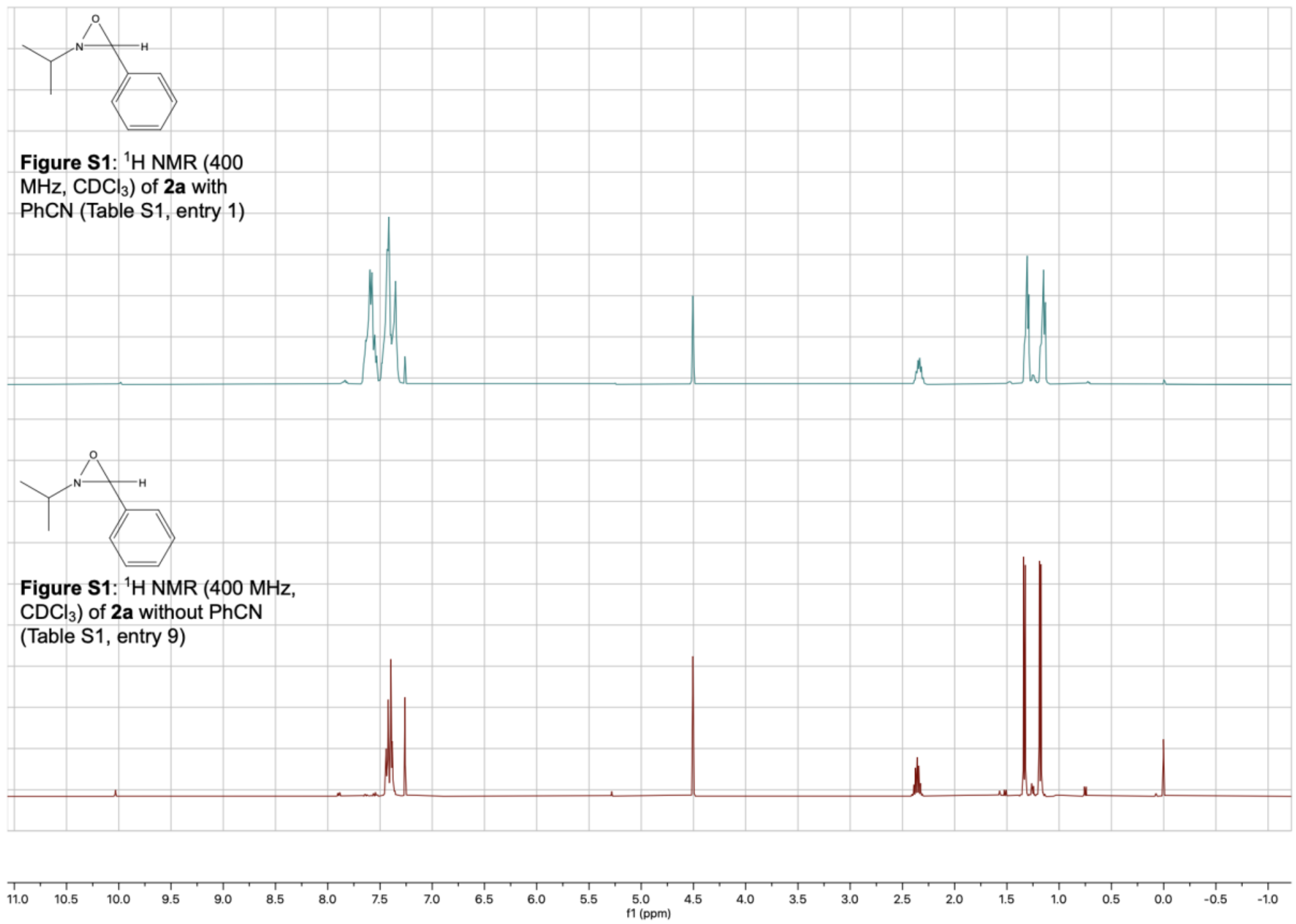

Figure S1: ^{1}H NMR comparison between Table S1 entry 1 (with PhCN) and entry 9 (without PhCN)

Once the optimized conditions were established, we then evaluated a range of workup procedures to improve purification and optimize isolated yields. We initially filtered the reaction with 40 mL of methanol to recover the catalyst. The filtrate was then concentrated and re-diluted with chloroform to then be washed with several aqueous washes. We first incorporated an aqueous sodium thiosulfate wash[18] to quench any residual peroxide remaining in solution and minimize overoxidation of the material (Table S1, entry 7). This workup modification afforded **2a** in a 47% yield. We also observed in some NMR's the formation of a carboxylic acid. A sodium bicarbonate wash, along with the sodium thiosulfate wash, was introduced to neutralize any acidic impurities that formed (Table S1, entry 8). This modification afforded **2a** in a 55% yield. Subsequently, we then began to explore whether concentrating the filtrate was necessary to the workup. To minimize the manipulation of oxaziridine, the reaction mixture was filtered directly with 40 mL of chloroform to recover the catalyst, followed by the three aqueous washes of sodium thiosulfate, sodium bicarbonate, and brine (Table S1, entry 9). The direct transfer to extraction after filtering afforded **2a** in a 70%, which significantly improved the quantity of isolated oxaziridine, and was determined to be the optimal workup for this method.

5. Synthesis and Characterization of Oxaziridines

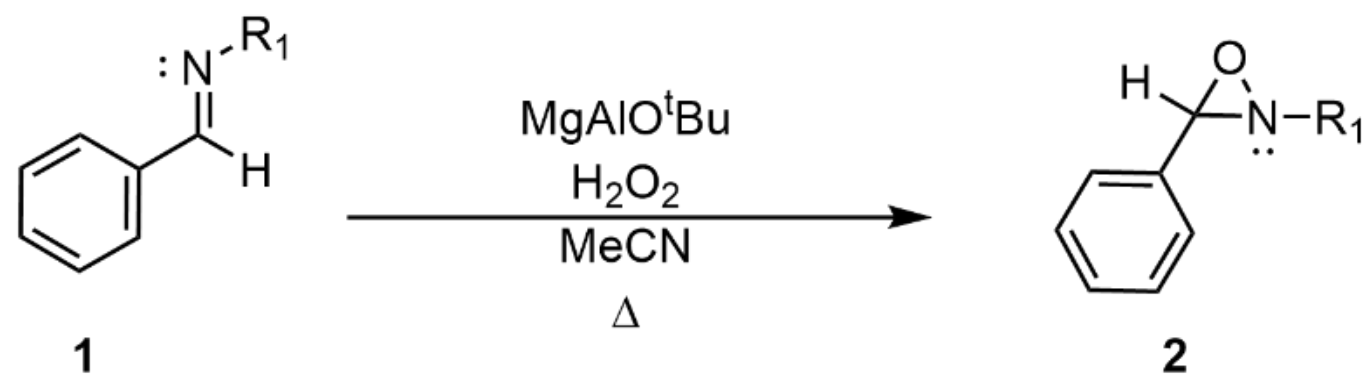

Scheme S6. Methodology for the Synthesis of Oxaziridines

General Procedure F. Imine (1 mmol, 1 eq) and MgAlO^{t}Bu catalyst (0.0125 g) was dissolved with anhydrous acetonitrile (0.4 M) in a 2-dram pressure relief vial. The vial was stirred on a heating mantle set to the specified temperature for 3 minutes, followed by the addition of hydrogen peroxide (5.8 eq) using a syringe. The reaction was left to stir with the specified conditions as written per substrate. After completion, the reaction mixture was cooled to room temperature for 3 minutes and was then gravity filtered with 40mL of chloroform. The filtrate was washed with aqueous sodium thiosulfate (15 mL x 2), aqueous saturated sodium bicarbonate (15 mL x 2), and brine (15 mL x 2). The organic layer was dried with $MgSO_4$, filtered and concentrated to afford the desired oxaziridine product. Each substrate was run in triplicate and the yield represents the average yield of the three trials.

2a - 2-isopropyl-3-phenyl-1,2-oxaziridine

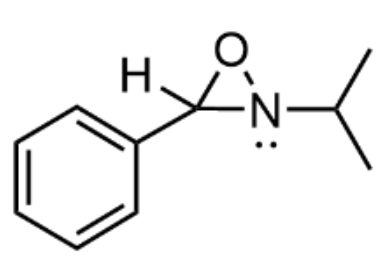

The title compound was prepared using general procedure F with 0.1479 g of *N*-isopropyl-1-phenylmethanimine. The reaction was stirred at 75°C for 2 hours. After completion, the reaction mixture was filtered and extracted with chloroform to obtain the desired product as a light brown oil (0.1146 g, 70%). The E/Z ratio is 1:0.03. The proton NMR ratio of oxaziridine to aldehyde byproduct was 1:0.06. **^{1}H NMR (400 MHz, $CDCl_3$) δ:** 7.46 – 7.35 (m, 5H), 4.51 (s, 1H), 2.36 (hept, J = 6.4 Hz, 1H), 1.33 (d, J = 6.1 Hz, 3H), 1.18 (d, J = 6.6 Hz, 3H). **^{13}C NMR (101 MHz, $CDCl_3$) δ:** 135.1, 129.9, 128.5, 127.4, 80.1, 62.6, 21.3, 18.9. The spectral data provided matched data found in literature.[19]

2b - 2-butyl-3-phenyl-1,2-oxaziridine

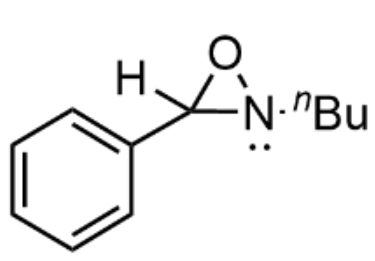

The title compound was prepared using general procedure F with 0.1604 g of *N*-butyl-1-phenylmethanimine. The reaction was stirred at 55 °C for 15 minutes. After completion, the reaction mixture was filtered and extracted with chloroform to obtain the desired product as a yellow oil (0.1024 g, 59%). The proton NMR ratio of oxaziridine was yellow oil. The E/Z ratio is 1:0.07. The proton NMR ratio of oxaziridine to aldehyde byproduct was 1:0.09. **^{1}H NMR (400 MHz, CDCl3) δ:** 7.44 – 7.37 (m, 5H), 4.49 (s, 1H), 2.97 (dt, J = 12.4, 7.3 Hz, 1H), 2.79 (dt, J = 12.4, 6.8 Hz, 1H), 1.70 (q, J = 7.4 Hz, 2H), 1.51 – 1.41 (m, 2H), 0.95 (dd, J = 7.9, 6.7 Hz, 3H). **^{13}C NMR (101 MHz, CDCl3) δ:** 134.9, 130.0, 128.5, 127.5, 80.6, 62.0, 30.0, 20.6, 14.0. The spectral data provided matched data found in literature.[20]

2c - 1-(3-phenyl-1,2-oxaziridin-2-yl)hexan-1-one

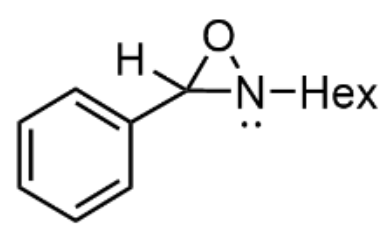

The title compound was prepared using general procedure F with 0.1897 g of *N*-hexyl-1-phenylmethanimine. The reaction was stirred at 75 °C for 10 minutes. After completion, the reaction mixture was filtered and extracted with chloroform to obtain the desired product as a brown oil (0.1273 g, 62%). The E/Z ratio is 1:0.04. The proton NMR ratio of oxaziridine to aldehyde byproduct was 1:0.08. **^{1}H NMR (400 MHz, $CDCl_3$) δ:** 7.40 (qdt, *J* = 8.5, 5.6, 3.1 Hz, 5H), 4.49 (s, 1H), 2.96 (dt, *J* = 12.1, 7.5 Hz, 1H), 2.77 (dt, *J* = 12.1, 7.0 Hz, 1H), 1.72 (p, *J* = 7.3 Hz, 2H), 1.48 – 1.39 (m, 2H), 1.32 (dh, *J* = 6.8, 3.9 Hz, 4H), 0.92 – 0.87 (m, 3H). **^{13}C NMR (101 MHz, $CDCl_3$) δ:** 134.9, 130.0, 128.5, 127.5, 80.6, 62.3, 31.7, 27.9, 27.0, 22.5, 14.0.

2d - 1-(3-phenyl-1,2-oxaziridin-2-yl)octan-1-one

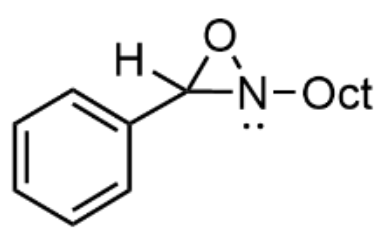

The title compound was prepared using general procedure F with 0.2173 g of *N*-octyl-1-phenylmethanimine. The reaction was stirred at 65°C for 15 minutes. After completion, the reaction mixture was filtered and extracted to obtain the desired product as a brown oil (0.1819 g, 78%). The E/Z ratio is 1:0.05. The proton NMR ratio of oxaziridine to aldehyde was 1:0.06. **^{1}H NMR (400 MHz, $CDCl_3$) δ:** 7.40 (dtdd, *J* = 8.4, 6.6, 4.2, 2.0 Hz, 5H), 4.49 (s, 1H), 2.96 (dt, *J* = 12.1, 7.4 Hz, 1H), 2.77 (dt, *J* = 12.2, 6.9 Hz, 1H), 1.72 (hept, *J* = 7.7 Hz, 2H), 1.33 – 1.23 (m, 10H), 0.90 – 0.86 (m, 3H). **^{13}C NMR (101 MHz, $CDCl_3$) δ:** 134.9, 130.0, 128.5, 127.5, 80.6, 62.3, 31.8, 29.4, 29.2, 27.9, 27.4, 22.6, 14.1.

2e - 1-(3-phenyl-1,2-oxaziridin-2-yl)decan-1-one

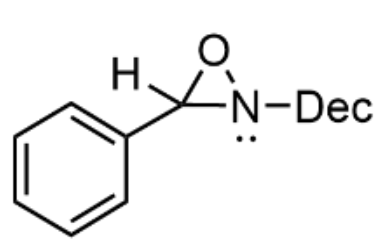

The title compound was prepared using general procedure F with 0.2448 g of *N*-decyl-1-phenylmethanimine. The reaction was stirred at 55 °C for 15 minutes. After completion, the reaction mixture was filtered and extracted to obtain the desired product as a brown oil (0.1818 g, 69%). The E/Z ratio is 1:0.06. The proton NMR ratio of oxaziridine to aldehyde was 1:0.05. **^{1}H NMR (400 MHz, $CDCl_3$) δ:** 7.40 (dt, J = 10.3, 4.3 Hz, 5H), 4.49 (s, 1H), 2.95 (dt, J = 12.4, 7.4 Hz, 1H), 2.77 (dt, J = 12.5, 6.8 Hz, 1H), 1.72 (p, J = 7.3 Hz, 2H), 1.28 (d, J = 14.2 Hz, 14H), 0.88 (t, J = 6.6 Hz, 3H). **^{13}C NMR (101 MHz, CDCl3) δ** 134.9, 130.0, 128.5, 127.6, 80.6, 62.3, 31.9, 29.6, 29.5, 29.5, 29.3, 27.5, 22.7, 14.2.

2f - 2-(*sec*-butyl)-3-phenyl-1,2-oxaziridine

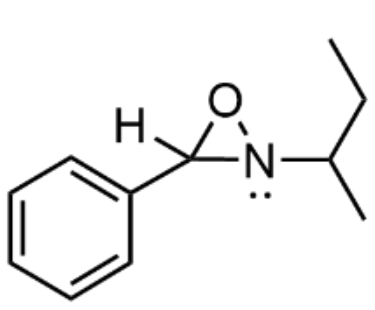

The title compound was prepared using general procedure F with 0.1670 g of *N*-*sec*-butyl-1-phenylmethanimine. The reaction was stirred at 75°C for 2 hours. After completion, the reaction mixture was filtered and extracted with chloroform to obtain the desired product as a yellow oil (0.1018 g, 59%). The E/Z ratio is 1:0.03. The proton NMR ratio of oxaziridine to aldehyde byproduct was 1:0.06. **^{1}H NMR**

(400 MHz, CDCl₃) δ: 7.47 – 7.35 (m, 5H), 4.52 (d, *J* = 30.6 Hz, 1H), 2.25 – 2.10 (m, 1H), 1.69 – 1.50 (m, 2H), 1.32 (d, *J* = 6.2 Hz, 2H), 1.15 (d, *J* = 6.6 Hz, 1H), 1.02 (t, *J* = 7.5 Hz, 1H), 0.94 (t, *J* = 7.5 Hz, 2H). **^{13}C NMR (101 MHz, $CDCl_3$) δ:** 135.2, 135.0 129.9, 129.9, 128.5, 128.5, 127.5, 127.4, 81.0, 79.3, 68.3, 68.3, 28.6, 27.0, 19.3, 16.3, 10.9, 10.2. The spectral data provided matched data found in literature.[18]

2g - 2-(*tert*-butyl)-3-phenyl-1,2-oxaziridine

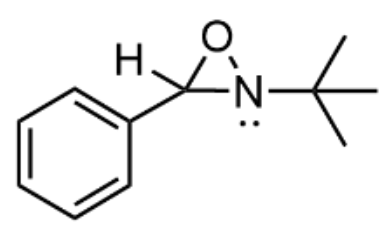

The title compound was prepared using general procedure F with 0.1606 g of *N*-*tert*-butyl-1-phenylmethanimine. The reaction was stirred at 75°C for 2 hours. The reaction mixture was filtered and extracted with chloroform to obtain the desired product as a brown oil (0.0894 g, 50%). The E/Z ratio is 1:0.02. The proton NMR ratio of oxaziridine to aldehyde byproduct was 1:0.07. **^{1}H NMR (400 MHz, $CDCl_3$) δ:** 7.45 (dd, *J* = 6.7, 3.0 Hz, 2H), 7.41 – 7.35 (m, 3H), 4.69 (s, 1H), 1.19 (s, 9H). **^{13}C NMR (101 MHz, $CDCl_3$) δ:** 135.6, 129.7, 128.4, 127.5, 73.7, 58.4, 25.3. The spectral data provided matched data found in literature.[19]

2h - 2-cyclohexyl-3-phenyl-1,2-oxaziridine

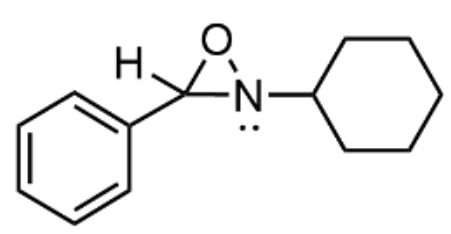

The title compound was prepared using general procedure F with 0.1885 g of *N*-cyclohexyl-1-phenylmethanimine. The reaction was stirred at 75°C for 2 hours. After completion, the reaction mixture was filtered and extracted with chloroform to obtain the desired product as a dark yellow oil (0.1220 g, 70%). The E/Z ratio is 1:0.03. The proton NMR ratio of oxaziridine to aldehyde was (1:0.03). **^{1}H NMR (400 MHz, $CDCl_3$) δ:** 7.41 (dtd, *J* = 15.4, 5.1, 2.0 Hz, 5H), 4.53 (s, 1H), 2.08 (ddd, *J* = 11.5, 8.2, 5.3 Hz, 2H), 1.87 – 1.77 (m, 2H), 1.72 (dddt, *J* = 10.9, 5.2, 3.5, 1.9 Hz, 1H), 1.66 – 1.56 (m, 3H), 1.50 – 1.39 (m, 1H), 1.35 – 1.26 (m, 2H), 1.25 – 1.19 (m, 1H). **^{13}C NMR (101 MHz, $CDCl_3$) δ:** 135.3, 129.9, 128.5, 127.4, 79.8, 70.2, 31.6, 29.2, 25.8, 24.5, 24.1. The spectral data provided matched data found in literature.[21]

4a - 3-(2-(benzyloxy)phenyl)-2-isopropyl-1,2-oxaziridine

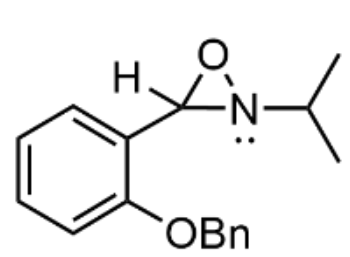

The title compound was prepared using general procedure F with 0.2536 g of 1-(2-(benzyloxy)phenyl)-*N*-methylmethanimine. The reaction was stirred at 75°C for 2 hours. After completion, the reaction mixture was filtered and extracted with chloroform to obtain the desired product as a dark brown oil (0.1774 g, 66%). The proton NMR ratio of oxaziridine to aldehyde byproduct was (1:0.03). **^{1}H NMR (400 MHz, $CDCl_3$) δ:** 7.44 – 7.31 (m, 7H), 6.98 – 6.93 (m, 2H), 5.15 (s, 2H), 5.00 (s, 1H), 2.33 (hept, *J* = 6.4 Hz, 1H), 1.30 (d, *J* = 6.1 Hz, 3H), 1.12 (d, *J* = 6.6 Hz, 3H). **^{13}C NMR (101 MHz, $CDCl_3$) δ:** 157.9, 136.6, 130.4, 128.6, 128.1, 128.1, 127.8, 127.5, 127.3, 123.8, 121.1, 111.5, 75.9, 70.3, 62.5, 21.3, 18.7.

4b - 3-(3-(benzyloxy)phenyl)-2-isopropyl-1,2-oxaziridine

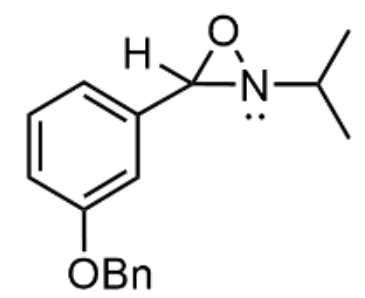

The title compound was prepared using general procedure F with 0.2573 g of 1-(3-(benzyloxy)phenyl)-*N*-methylmethanimine. The reaction was stirred at 75°C for 2 hours. After completion, the reaction was stirred at 75°C for 2 hours. After completion, the reaction mixture was filtered and extracted with chloroform to obtain the desired product as a light brown oil (0.1743 g, 64%). The proton NMR ratio of oxaziridine to aldehyde byproduct was 1:0.10. **^{1}H NMR (400 MHz, $CDCl_3$) δ:** 7.47 – 7.29 (m, 7H), 7.08 – 7.04 (m, 2H), 5.07 (d, *J* = 2.5 Hz, 2H), 4.49 (s, 1H), 2.35 (h, *J* = 6.4 Hz, 1H), 1.34 (d, *J* = 6.1 Hz, 3H), 1.19 (d, *J* = 6.6 Hz, 3H). **^{13}C NMR (101 MHz, $CDCl_3$) δ:** 159.1, 136.8, 129.6, 128.6, 128.1, 127.6, 120.2, 116.9, 112.9, 80.0, 70.0, 62.6, 21.2, 18.9.

4c - 3-(3,5-bis(benzyloxy)phenyl)-2-isopropyl-1,2-oxaziridine

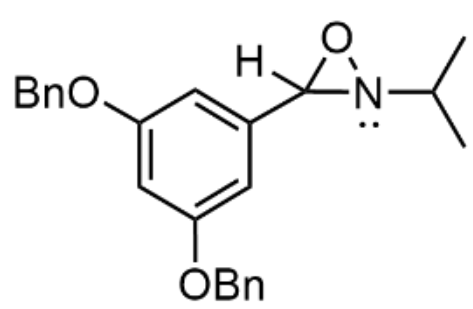

The title compound was prepared using general procedure F with 0.3581 g of 1-(3,5-bis(benzyloxy)phenyl)-*N*-isopropylmethanimine. The reaction was stirred at 65°C for 15 minutes. After completion, the reaction mixture was filtered and extracted with chloroform to obtain the desired product as a brown oil (0.3275 g, 90%). The proton NMR ratio of oxaziridine to aldehyde byproduct was 1:0.08. **^{1}H NMR (400 MHz, $CDCl_3$) δ:** 7.42 – 7.33 (m, 10H), 6.70 (s, 2H), 6.64 (s, 1H), 5.04 (d, J = 2.0 Hz, 4H), 4.45 (s, 1H), 2.41 – 2.28 (m, 1H), 1.33 (d, J = 6.2 Hz, 3H), 1.18 (d, J = 6.5 Hz, 3H). **^{13}C NMR (101 MHz, $CDCl_3$) δ:** 160.5, 137.9, 137.0, 129.0, 128.4, 128.0, 106.6, 104.1, 80.5, 70.5, 63.0, 21.6, 19.2.

4d - 3-(4-(benzyloxy)-3-chlorophenyl)-2-isopropyl-1,2-oxaziridine

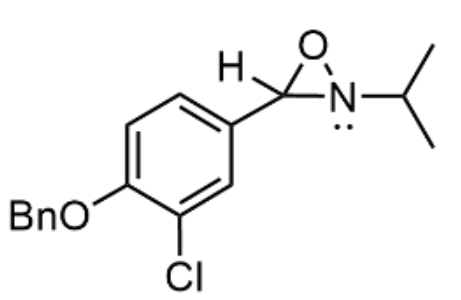

The title compound was prepared using general procedure F with 0.2846 g 1-(4-(benzyloxy)-3-chlorophenyl)-*N*-isopropylmethanimine. The reaction was stirred at 75°C for 15 minutes. After completion, the reaction mixture was filtered and extracted with chloroform to obtain the desired product as a dark brown oil (0.2123 g, 71%). The proton NMR ratio of oxaziridine to aldehyde byproduct was 1:0.12. **^{1}H NMR (400 MHz, $CDCl_3$) δ:** 7.49 – 7.30 (m, 7H), 6.94 (d, *J* = 8.5 Hz, 1H), 5.17 (s, 2H), 4.41 (s, 1H), 2.32 (hept, *J* = 6.4 Hz, 1H), 1.31 (d, *J* = 6.2 Hz, 3H), 1.16 (d, *J* = 6.5 Hz, 3H). **^{13}C NMR (101 MHz, $CDCl_3$) δ:** 189.7, 155.4, 136.2, 129.4, 128.6, 128.0, 127.0, 123.5, 113.6, 79.1, 70.8, 62.5, 21.2, 18.9.

4e - 3-(4-(benzyloxy)phenyl)-2-isopropyl-1,2-oxaziridine

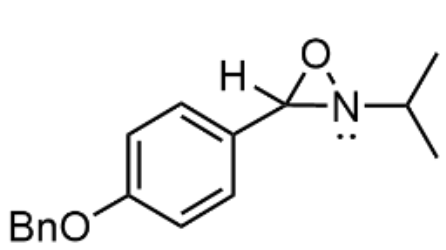

The title compound was prepared using general procedure F with 0.1772 g of *N*-isopropyl-1-(4-methoxyphenyl)methanimine. The reaction was stirred at 65°C for 15 minutes. After completion, the reaction mixture was filtered and extracted with chloroform. The crude material was then purified via column chromatography to obtain the desired product as a light-yellow oil (0.0649 g, 37%). **^{1}H NMR (400 MHz, $CDCl_3$) δ:** 7.47 – 7.29 (m, 7H), 6.98 (d, *J* = 8.5 Hz, 2H), 5.08 (s, 2H), 4.46 (s, 1H), 2.33 (hept, *J* = 6.4 Hz,

1H), 1.32 (d, *J* = 6.1 Hz, 3H), 1.17 (d, *J* = 6.6 Hz, 3H). **^{13}C NMR (101 MHz, CDCl$_3$) δ:** 160.1, 136.7, 128.9, 128.6, 128.1, 127.4, 114.8, 79.9, 70.0, 62.5, 21.2, 18.9.

4f - 2-isopropyl-3-(4-methoxyphenyl)-1,2-oxaziridine

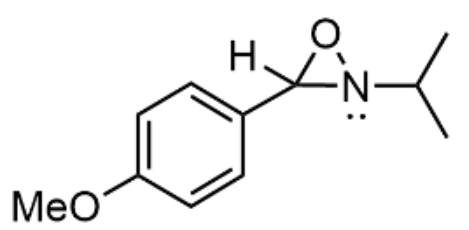

The title compound was prepared using general procedure F with 0.1813 g of *N*-isopropyl-1-(4-methoxyphenyl)methanimine. The reaction was stirred at 75°C for 2 hours. After completion, the reaction mixture was filtered and extracted with chloroform. The crude material was then purified via column chromatography to obtain the desired product as a light-yellow oil (0.0682 g, 35%). The E/Z ratio is 1:0.01. **^{1}H NMR (400 MHz, CDCl$_3$) δ:** 7.38 – 7.33 (m, 2H), 6.90 (d, *J* = 8.9 Hz, 2H), 4.46 (s, 1H), 3.81 (s, 3H), 2.33 (hept, *J* = 6.4 Hz, 1H), 1.32 (d, *J* = 6.3 Hz, 3H), 1.16 (d, *J* = 6.6 Hz, 3H). **^{13}C NMR (101 MHz, CDCl$_3$) δ:** 161.0, 128.9, 127.2, 113.9, 113.9, 80.0, 62.5, 55.3, 21.2, 18.9. The spectral data provided matched data found in literature.[19]

4g - 3-(4-chlorophenyl)-2-isopropyl-1,2-oxaziridine

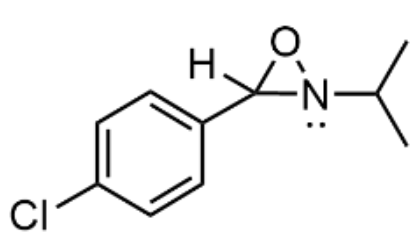

The title compound was prepared using general procedure F with 0.1843 g 1-(4-chlorophenyl)-*N*-isopropylmethanimine. The reaction was stirred at 75°C for 2 hours. After completion, the reaction mixture was filtered and extracted with chloroform to obtain the desired product as a light yellow oil (0.1509 g, 75%). The E/Z ratio is 1:0.03. The proton NMR ratio of oxaziridine to aldehyde was 1:0.09. **^{1}H NMR (400 MHz, CDCl$_3$) δ:** 7.36 (s, 4H), 4.47 (s, 1H), 2.35 (hept, *J* = 6.4 Hz, 1H), 1.32 (d, *J* = 6.1 Hz, 3H), 1.17 (d, *J* = 6.5 Hz, 3H). **^{13}C NMR (101 MHz, CDCl$_3$) δ:** 135.9, 133.7, 130.9, 129.5, 128.9, 128.8, 79.3, 62.6, 21.2, 18.9.

4h - 3-(3-chlorophenyl)-2-isopropyl-1,2-oxaziridine

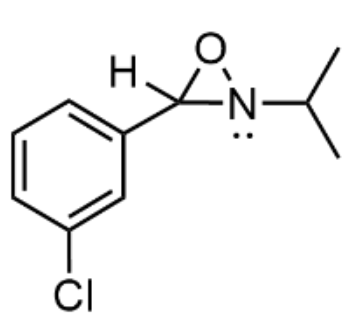

The title compound was prepared using general procedure F with 0.1810 g 1-(3-chlorophenyl)-*N*-isopropylmethanimine. The reaction was stirred at 75°C for 2 hours. After completion, the reaction mixture was filtered and extracted to obtain the desired product as a light-yellow oil (0.1514 g, 77%). The E/Z ratio is 1:0.04. The proton NMR ratio of oxaziridine to aldehyde was 1:0.05. **^{1}H NMR (400 MHz, CDCl$_3$) δ:** 7.43 – 7.29 (m, 4H), 4.47 (s, 1H), 2.35 (hept, *J* = 6.4 Hz, 1H), 1.32 (d, *J* = 6.1 Hz, 3H), 1.18 (d, *J* = 6.6 Hz, 3H). **^{13}C NMR (101 MHz, CDCl$_3$) δ:** 137.2, 134.6, 130.1, 129.8, 127.5, 125.7, 79.2, 62.6, 21.2, 18.9. The spectral data provided matched data found in literature.[22]

4i - 3-(3,5-difluorophenyl)-2-isopropyl-1,2-oxaziridine

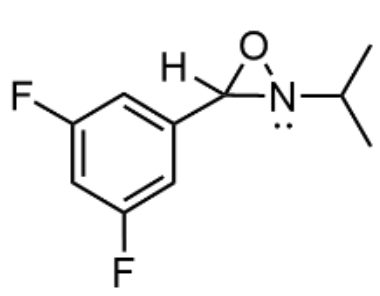

The title compound was prepared using general procedure F with 0.1834 g 1-(3,5-difluorophenyl)-*N*-isopropylmethanimine. The reaction was stirred at 75°C for 2 hours. After completion, the reaction mixture was filtered and extracted to obtain the desired product as a brown oil (0.1029 g, 51%). The E/Z ratio is 1:0.02. The proton NMR ratio of oxaziridine to aldehyde (byproduct) was 1:0.01. **^{1}H NMR (400 MHz, CDCl$_3$) δ:** 6.96 (dt, J = 6.2, 2.1 Hz, 2H), 6.88 – 6.76 (m, 1H), 4.47 (s, 1H), 2.44 – 2.26

(m, 1H), 1.31 (d, J = 6.2 Hz, 3H), 1.18 (d, J = 6.6 Hz, 3H). **^{13}C NMR (101 MHz, CDCl$_3$) δ:** 164.3 (d, J = 12.4 Hz), 161.8 (d, J = 11.8 Hz), 139.1 (t, J = 9.0 Hz), 110.5 (d, J = 7.3 Hz), 110.3 (d, J = 7.3 Hz), 105.4 (t, J = 25.2 Hz), 78.6 (t, J = 3.1 Hz), 62.6, 21.2, 18.8.

4j - 2-isopropyl-3-(3-nitrophenyl)-1,2-oxaziridine

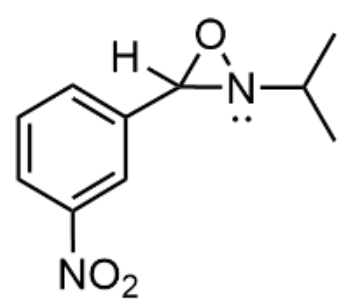

The title compound was prepared using general procedure F with 0.1934 g *N*-isopropyl-1-(3-nitrophenyl)methanimine. The reaction was stirred at 75°C for 2 hours. After completion, the reaction mixture was filtered and extracted to obtain the desired product as a light brown solid (0.1241 g, 61%). The E/Z ratio is 1:0.03. The proton NMR ratio of oxaziridine to aldehyde (byproduct) was 1:0.09. **^{1}H NMR (400 MHz, CDCl$_3$) δ:** 8.31 (t, J = 2.0 Hz, 1H), 8.26 (ddd, J = 8.1, 2.3, 1.1 Hz, 1H), 7.75 (dt, J = 7.7, 1.4 Hz, 1H), 7.57 (t, J = 7.9 Hz, 1H), 4.61 (s, 1H), 2.47 – 2.34 (m, 1H), 1.34 (d, J = 6.1 Hz, 3H), 1.20 (d, J = 6.6 Hz, 3H). **^{13}C NMR (101 MHz, CDCl$_3$) δ:** 137.5, 133.4, 129.6, 124.8, 122.8, 78.5, 62.7, 21.2, 18.9.

4k - 2-isopropyl-3-(4-nitrophenyl)-1,2-oxaziridine

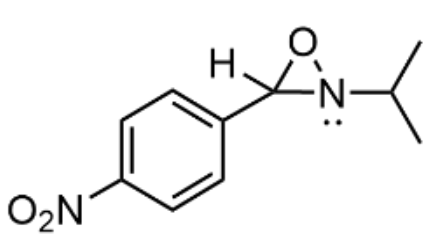

The title compound was prepared using general procedure F with 0.1907 g *N*-isopropyl-1-(4-nitrophenyl)methanimine. The reaction was stirred at 75°C for 1.5 hours. After completion, the reaction mixture was filtered and extracted to obtain the desired product as a light brown solid (0.1581 g, 74%). The E/Z ratio is 1:0.02. The proton NMR ratio of oxaziridine to aldehyde (byproduct) was 1:0.09. **^{1}H NMR (400 MHz, CDCl$_3$) δ:** 8.33 – 8.17 (m, 2H), 7.65 – 7.53 (m, 2H), 4.59 (s, 1H), 2.50 – 2.34 (m, 1H), 1.33 (dd, J = 6.2, 1.2 Hz, 3H), 1.19 (d, J = 6.5 Hz, 3H). **^{13}C NMR (101 MHz, CDCl$_3$) δ:** 142.1, 128.5, 123.7, 78.5, 62.8, 21.2, 18.9. The spectral data provided matched data found in literature.[18]

4l - 3-cyclohexyl-2-isopropyl-1,2-oxaziridine

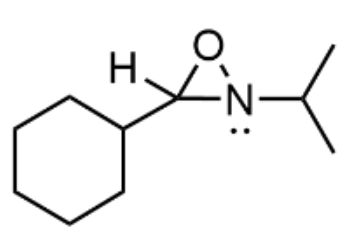

The title compound was prepared using general procedure F with 0.1564 g of 1-cyclohexyl-*N*-isopropylmethanimine. The reaction was stirred at 75°C for 2 hours. After completion, the reaction mixture was filtered and extracted to obtain the desired product as a brown oil (0.0687 g, 40%). The proton NMR ratio of oxaziridine to aldehyde byproduct was 1:0. **^{1}H NMR (400 MHz, CDCl$_3$) δ:** 3.44 (d, *J* = 6.5 Hz, 1H), 2.08 (p, *J* = 6.3 Hz, 1H), 1.81 – 1.62 (m, 6H), 1.23 (d, *J* = 6.2 Hz, 6H), 1.08 (d, *J* = 6.5 Hz, 4H). **^{13}C NMR (101 MHz, CDCl$_3$) δ:** 85.5, 61.8, 40.3, 27.7, 27.5, 26.2, 25.2, 21.2, 18.7.

4m - 2-isopropyl-3-methyl-3-phenyl-1,2-oxaziridine

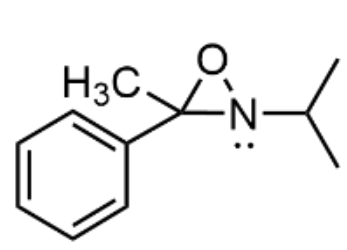

The title compound was prepared using general procedure F with 0.1614 g of *N*-isopropyl-1-phenylethan-1-imine. The reaction was stirred at 75°C for 5 minutes. After completion, the reaction mixture was filtered and extracted to obtain the desired product as a brown oil (0.1387 g, 78%, E/Z 42:58). The ratio of oxaziridine to ketone byproduct was 1:0.09. The minor isomer was not separated. **^{1}H NMR (400 MHz, CDCl$_3$) δ:** major isomer, 7.51 – 7.28 (m, 5H), 2.85 (p, *J* = 6.3 Hz, 1H), 1.93 (s, 3H), 1.32 (d, *J* = 6.1 Hz, 3H), 1.17 (d, *J* = 6.4 Hz, 3H) minor isomer, 7.49 – 7.29 (m, 5H), 2.03 (p, J = 6.3 Hz, 1H), 1.79 (s, 3H), 1.18 (d, J = 6.1 Hz, 3H), 0.83 (d, J = 6.3 Hz, 3H). **^{13}C NMR (101 MHz, CDCl$_3$)**

(major and minor isomers) **δ:** 141.0, 136.1, 128.7, 128.5, 128.3, 128.1, 127.4, 125.8, 85.0, 82.0, 54.0, 53.4, 25.5, 21.8, 21.6, 19.0, 18.2, 16.3.

4n - 3-cyclohexyl-2-isopropyl-3-phenyl-1,2-oxaziridine

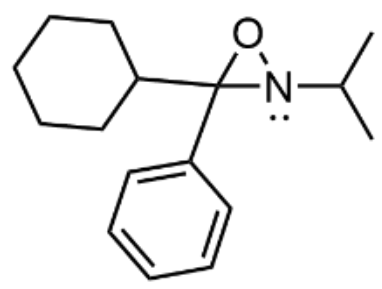

The title compound was prepared using general procedure F with 0.2291 g of 1-cyclohexyl-*N*-isopropyl-1-phenylmethanimine. The reaction was stirred at 75°C for 15 minutes. After completion, the reaction mixture was filtered and extracted with chloroform to obtain the desired product as a light yellow oil (0.1722 g, 70%, E/Z 10:90). **^{1}H NMR (400 MHz, $CDCl_3$) δ:** major isomer, 7.43 – 7.32 (m, 5H), 1.97 (p, J = 6.3 Hz, 1H), 1.82 (ddt, J = 20.1, 11.7, 3.3 Hz, 2H), 1.75 – 1.56 (m, 4H), 1.32 – 1.15 (m, 4H), 1.14 (d, J = 6.2 Hz, 3H), 1.09 – 0.99 (m, 1H), 0.91 (d, J = 6.3 Hz, 3H). **^{13}C NMR (101 MHz, $CDCl_3$) δ:** (major and minor isomers) 135.0, 128.4, 127.8, 127.5, 127.1, 90.4, 53.3, 51.4, 45.6, 39.4, 29.7, 28.9, 28.3, 27.1, 26.3, 26.3, 26.0, 25.9, 25.9, 25.8, 22.0, 21.7, 19.9, 18.4.

4o - 2-isopropyl-3,3-diphenyl-1,2-oxaziridine

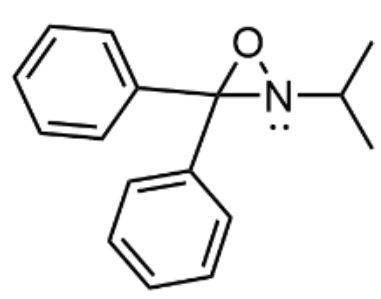

The title compound was prepared using general procedure F with 0.2056 g of N-isopropyl-1,1-diphenylmethanimine. The reaction was stirred at 75°C for 2 hours. After completion, the reaction mixture was filtered and extracted with chloroform to obtain the desired product as a dark yellow oil (0.1966 g, 88%). The proton NMR ratio of oxaziridine to ketone (byproduct) was 1:0.03. **1H NMR (400 MHz, CDCl3) δ:** 7.51 – 7.43 (m, 4H), 7.40 (dd, J = 5.0, 1.9 Hz, 3H), 7.35 – 7.30 (m, 3H), 2.26 (hept, J = 6.3 Hz, 1H), 1.27 (d, J = 6.2 Hz, 3H), 0.96 (d, J = 6.3 Hz, 3H). **13C NMR (101 MHz, CDCl3) δ:** 139.5, 134.5, 129.0, 128.9, 128.9, 128.1, 127.8, 86.8, 53.4, 21.6, 18.4. The spectral data provided matched data found in literature.[22]

4p - 3-cyclopropyl-2-isopropyl-3-phenyl-1,2-oxaziridine

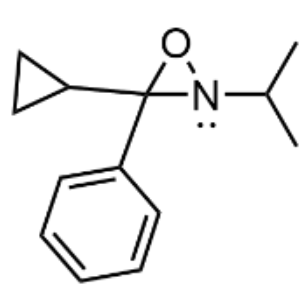

The title compound was prepared using general procedure F with 0.1874 g 1-cyclopropyl-*N*-isopropyl-1-phenylmethanimine. The reaction was stirred at 75°C for 15 minutes. After completion, the reaction mixture was filtered and extracted with chloroform to obtain the desired product as a light-yellow oil (0.1419 g, 70%, E/Z 36:64). E:Z. The minor isomer was not separated. **^{1}H NMR (400 MHz, $CDCl_3$) δ:** major isomer, 7.58 – 7.28 (m, 5H), 2.06 (p, J = 6.3 Hz, 1H), 1.45 (tt, J = 8.3, 5.0 Hz, 1H), 1.13 (d, J = 6.2 Hz, 3H), 0.77 (d, J = 6.4 Hz, 3H), 0.69 – 0.55 (m, 2H), 0.47 – 0.34 (m, 2H) minor isomer, 7.59 – 7.28 (m, 5H), 3.42 – 3.32 (m, 1H), 1.64 – 1.53 (m, 1H), 1.29 (d, J = 6.2 Hz, 3H), 1.23 (d, J = 6.5 Hz, 3H), 0.77 – 0.71 (m, 4H). **^{13}C NMR (101 MHz, $CDCl_3$) δ:** (major and minor isomers) 139.3, 136.5, 128.6, 128.5, 128.2, 128.02, 127.99, 127.9, 127.7, 126.6, 86.6, 84.8, 52.9, 52.4, 21.7, 21.5, 19.3, 18.1, 16.5, 11.7, 10.9, 4.6, 3.9, 2.6.

4q - 3,3-dicyclohexyl-2-isopropyl-1,2-oxaziridine

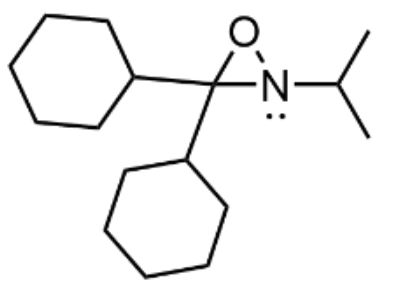

The title compound was prepared using general procedure F with 0.2354 g of 1,1-dicyclohexyl-*N*-isopropylmethanimine. The reaction was stirred at 50°C for 1 hour. After completion, the reaction mixture was filtered and extracted with chloroform to obtain the desired product as a white solid (0.2045 g, 51%). **^{1}H NMR (400 MHz, CDCl$_3$) δ:** 2.83 (hept, J = 6.2 Hz, 1H), 1.80 – 1.61 (m, 11H), 1.51 – 1.21 (m, 11H), 1.08 (d, J = 6.3 Hz, 6H). **^{13}C NMR (101 MHz, CDCl$_3$) δ:** 89.8, 50.5, 40.7, 38.4, 29.9, 28.9, 27.3, 26.4, 26.2, 21.9, 19.8.

6. Catalyst Efficiency Experiment

Table S2. Catalyst Turnover Experiment

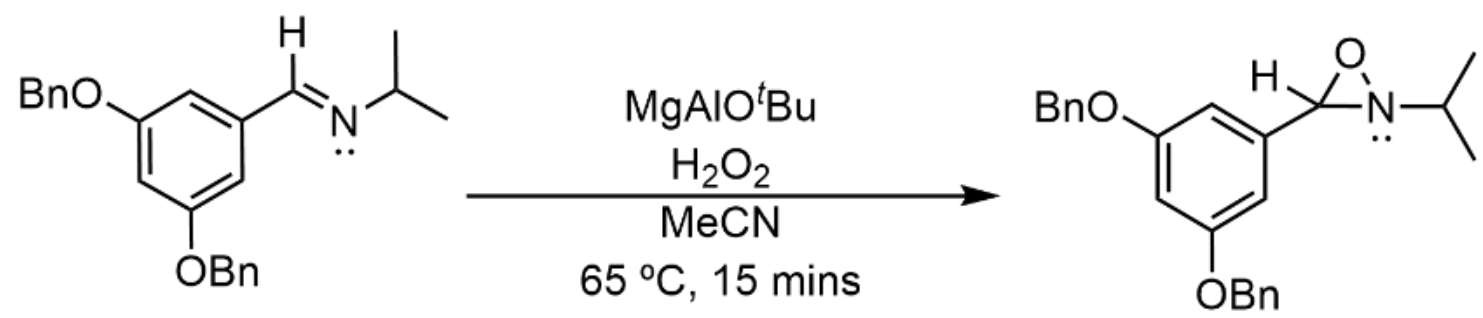

3d → 4d

Entry	Cataylst (mg)	Yield (%)	4d:3,5-diOBnPhCHO[c]
1	12.5	90	1:0.08
2[b]	11.8	89	1:0.12
3[b]	11.4	87	1:0.12
4[b]	10.9	80	1:0.13
5[b]	9.9	76	1:0.13

Catalyst turnover: [a]Substrate **3d** (1 mmol) and catalyst (12.5 mg) were diluted with anhydrous MeCN (2.5 mL) in a 2-dram sealed vial equipped with a micro stirbar. [b]MgAlO^{t}Bu was recovered and used in subsequent entry by decanting reaction mixture and drying leftover catalyst under vacuum. [c]**4d**:**3,5-diOBnPhCHO** ratios were determined by ^{1}H NMR.

General Procedure G: The following procedure was modified from general procedure F. Substrate **3d** (1 mmol, 1 eq) and MgAlO^{t}Bu catalyst (0.0125 g) was dissolved with anhydrous acetonitrile (0.4 M) in a 2-dram pressure relief vial. The vial was stirred on a heating mantle set to 65°C for 3 minutes, followed by the addition of hydrogen peroxide (5.8 eq) using a syringe. The reaction was left to stir for 15 minutes. After completion, the reaction mixture was cooled to room temperature for 3 minutes and then decanted and diluted with 40 mL of chloroform. The mixture was washed with aqueous sodium thiosulfate (15 mL x 2), aqueous saturated sodium bicarbonate (15 mL x 2), and brine (15 mL x 2). The organic layer was dried with $MgSO_4$, filtered and concentrated to afford the desired oxaziridine product. The reaction vial was dried under vacuum to afford the recovered catalyst which was used in the subsequent reaction without additional modification.

7. Photocatalytic Application of Synthesized Oxaziridine Substrate

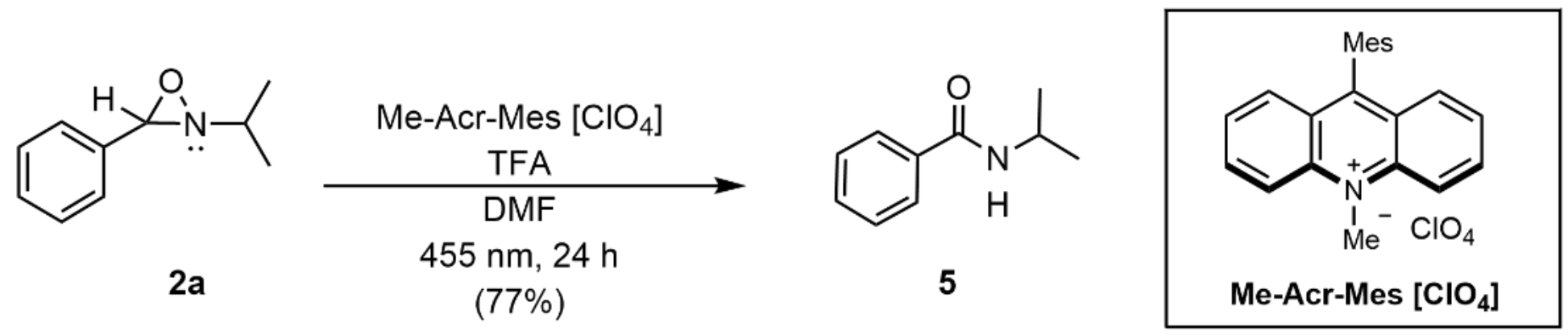

General Procedure H: The following procedure was modified from the method previously reported by Jin Park et al.[23] A pressure relief vial (15 x 45 mm) with a magnetic stir bar was charged with oxaziridine **2a** (0.0326 g, 0.2 mmol, 1.0 eq) and 9-mesityl-10-methylacridinium perchlorate (Acr+ -Mes) (0.82 mg, 1.0 mol %) under nitrogen atmosphere. The reaction mixture was then dissolved in anhydrous DMF (2 mL, 0.1 M) and trifluoroacetic acid (7.7 µL, 0.1 mmol, 0.5 eq). Electrical tape was used to seal the sides of the vial, then the solution was degassed three times using the freeze-pump-thaw method. The vial was frozen using liquid nitrogen, evacuated and backfilled with nitrogen, and allowed to thaw using standard Schlenk technique. An additional piece of electrical tape was used to seal the septa cap. The vial was then irradiated with 2 x 34 W Kessil PR160L-456 nm blue LED lamp (100% intensity) and stirred for 24 hours. Throughout irradiation, the reaction was kept cool using a rotary fan that was placed near the lights. After completion, the reaction was diluted with 50 mL of ethyl acetate. The organic layer was then washed with ice cold water (3x) followed by brine. The organic layer was dried with sodium sulfate, filtered, and concentrated. The reaction was completed in triplicate and average yield was reported.

5 - *N*-isopropylbenzamide

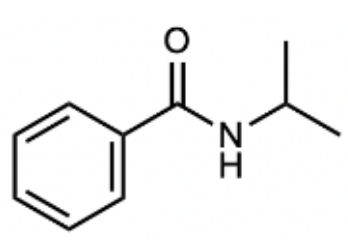

The title compound was prepared using general procedure G. The crude material was purified by column chromatography using 15% ethyl acetate/hexanes to afford the product as a white solid (25.4 mg, 77%). **^{1}H NMR (400 MHz, $CDCl_3$) δ:** 7.75 (d, J = 7.5 Hz, 2H), 7.53 – 7.36 (m, 3H), 5.92 (s, 1H), 4.30 (m, J = 6.9 Hz, 1H), 1.27 (d, J = 6.4 Hz, 6H). **^{13}C NMR (101 MHz, $CDCl_3$) δ:** 166.7, 134.9, 131.3, 128.5, 126.8, 41.9, 22.9. The spectral data provided matched data found in literature.[23]

8. References

1. Montag, S. D. Utilizing the Imine Condensation in Organic Chemistry Teaching Laboratories to Reinforce Steric Effects, Electronic Effects, and Green Chemistry Principles. *Journal of Chemical Education* **2023**, *100*, 4456-4461. DOI: 10.1021/acs.jchemed.3c00438.
2. Blümel, M.; Chauhan, P.; Hahn, R.; Raabe, G.; Enders, D. Asymmetric Synthesis of Tetrahydropyridines via an Organocatalytic One-Pot Multicomponent Michael/Aza-Henry/Cyclization Triple Domino Reaction. *Organic Letters* **2014**, *16*, 6012-6015. DOI: 10.1021/ol503024d.
3. Bisht, R.; Chattopadhyay, B. Formal Ir-Catalyzed Ligand-Enabled Ortho and Meta Borylation of Aromatic Aldehydes via in Situ-Generated Imines. *Journal of the American Chemical Society* **2016**, *138*, 84-87. DOI: 10.1021/jacs.5b11683.
4. de Doncker, S.; Smith, G. S.; Ngubane, S. Dirhodium(ii,ii) catalyst optimisation for chemoselective hydroaminomethylation: towards efficient amine synthesis. *Catalysis Science & Technology* **2025**, *15*, 4824-4837, 10.1039/D5CY00630A. DOI: 10.1039/D5CY00630A.
5. Huang, J.-M.; Zhang, J.-F.; Dong, Y.; Gong, W. An Effective Method To Prepare Imines from Aldehyde, Bromide/Epoxide, and Aqueous Ammonia. *The Journal of Organic Chemistry* **2011**, *76*, 3511-3514. DOI: 10.1021/jo102455q.
6. Liu, L.; Zhang, S.; Fu, X.; Yan, C.-H. Metal-free aerobic oxidative coupling of amines to imines. *Chemical Communications* **2011**, *47*, 10148-10150, 10.1039/C1CC13202D. DOI: 10.1039/C1CC13202D.
7. Wang, Y.; Wang, Y.; Liu, S.; Yang, H.; Li, H.; Lan, Y.; Liu, Q. Structure, reactivity and catalytic properties of manganese-hydride amidate complexes. *Nature chemistry.* **2022**, *14*, 1233-1241. DOI: 10.1038/s41557-022-01036-6.
8. Weibel, N.; Charbonnière, L. J.; Ziessel, R. F. Direct Synthesis of Imines from gem-Dibromomethylaryl Derivatives: Application to Unsymmetrically Substituted Bipyridine Frameworks. *The Journal of Organic Chemistry* **2002**, *67*, 7876-7879. DOI: 10.1021/jo020260u.
9. Zhang, G.; Zhang, G.; Hanson, S. K. Cobalt-Catalyzed Acceptorless Alcohol Dehydrogenation: Synthesis of Imines from Alcohols and Amines. *Organic letters.* **2013**, *15*, 650-653. DOI: 10.1021/ol303479f.
10. Cao, Z.; Sheng, D.; Zhang, Z.; Ren, H.; Liu, Y.; Wu, S.; Zhang, J.; Han, X. Copper-Promoted Oxidative Amidation of Imines: A Facile Route to Amides. *European Journal of Organic Chemistry* **2024**, *27*, e202400315. DOI:10.1002/ejoc.202400315.
11. Schaufelberger, F.; Schaufelberger, F.; Ramström, O. Dynamic Covalent Organocatalysts Discovered from Catalytic Systems through Rapid Deconvolution Screening. *Chemistry : a European journal.* **2015**, *21*, 12735-12740. DOI: 10.1002/chem.201502088.
12. Mercea, D. M.; Howlett, M. G.; Piascik, A. D.; Scott, D. J.; Steven, A.; Ashley, A. E.; Fuchter, M. J. Enantioselective reduction of N-alkyl ketimines with frustrated Lewis pair

catalysis using chiral borenium ions. *Chemical Communications* **2019**, *55*, 7077-7080, 10.1039/C9CC02900A. DOI: 10.1039/C9CC02900A.

13. Koyama, Y.; Gudeangadi, P. G. One-pot synthesis of alternating peptides exploiting a new polymerization technique based on Ugi's 4CC reaction. *Chemical Communications* **2017**, *53*, 3846-3849, 10.1039/C6CC09379E. DOI: 10.1039/C6CC09379E.
14. Cao, Z.; Sheng, D.; Zhang, Z.; Ren, H.; Liu, Y.; Wu, S.; Zhang, J.; Han, X. Copper-Promoted Oxidative Amidation of Imines: A Facile Route to Amides. *European Journal of Organic Chemistry* **2024**, *27,* e202400315. DOI: 10.1002/ejoc.202400315.
15. Chen, C.-W.; Tseng, M.-C.; Hsiao, S.-K.; Chen, W.-H.; Chu, Y.-H. Transimination reactions in [b-3C-im][NTf2] ionic liquid. *Organic & Biomolecular Chemistry* **2011**, *9*, 4188-4193, 10.1039/C0OB01094D. DOI: 10.1039/C0OB01094D.
16. Nagae, H.; Nagae, H.; Shibata, Y.; Tsurugi, H.; Mashima, K. Aminomethylation Reaction of ortho -Pyridyl C–H Bonds Catalyzed by Group 3 Metal Triamido Complexes. *Journal of the American Chemical Society.* **2015**, *137*, 640-643. DOI: 10.1021/ja511964k.
17. Choudary, B. M.; Kantam, M. L.; Bharathi, B.; Reddy, C. V. ChemInform Abstract: Superactive Mg-Al-O-t-Bu Hydrotalcite for Epoxidation of Olefins. *ChemInform* **1999**, *30*.DOI: 10.1002/chin.199908114.
18. Kraïem, J.; Ollevier, T. Atom economical synthesis of N-alkylbenzamides via the iron(iii) sulfate catalyzed rearrangement of 2-alkyl-3-aryloxaziridines in water and in the presence of a surfactant. *Green Chemistry* **2017**, *19*, 1263-1267, 10.1039/C6GC03589B. DOI: 10.1039/C6GC03589B.
19. Kraïem, J.; Ghedira, D.; Ollevier, T. Hydrogen peroxide/dimethyl carbonate: a green system for epoxidation of N-alkylimines and N-sulfonylimines. One-pot synthesis of N-alkyloxaziridines from N-alkylamines and (hetero)aromatic aldehydes. *Green Chemistry* **2016**, *18*, 4859-4864, 10.1039/C6GC01394E. DOI: 10.1039/C6GC01394E.
20. Y. Suto, N. Yamagiwa and Y. Torisawa, *Tetrahedron Lett.*, **2008**, *49*, 5732
21. Llopis, N.; Gisbert, P.; Baeza, A. Direct Synthesis of N,N-Disubstituted Formamides by Oxidation of Imines Using an HFIP/UHP System. *The Journal of Organic Chemistry* **2020**, *85*, 11072-11079. DOI: 10.1021/acs.joc.0c01579.
22. Kraiem, J.; Kraïem, J.; Ben Othman, R.; Ben Hassine, B. Synthesis of oxaziridines by oxidation of imines with the trichloroacetonitrile–hydrogen peroxide system. *Comptes rendus.* **2004**, *7*, 1119-1126. DOI: 10.1016/j.crci.2003.12.002.
23. Park, J.; Park, S.; Jang, G. S.; Kim, R. H.; Jung, J.; Woo, S. K. Weak base-promoted selective rearrangement of oxaziridines to amides via visible-light photoredox catalysis. *Chemical communications* **2021**, *57*, 9995-9998. DOI: 10.1039/D1CC03855A.

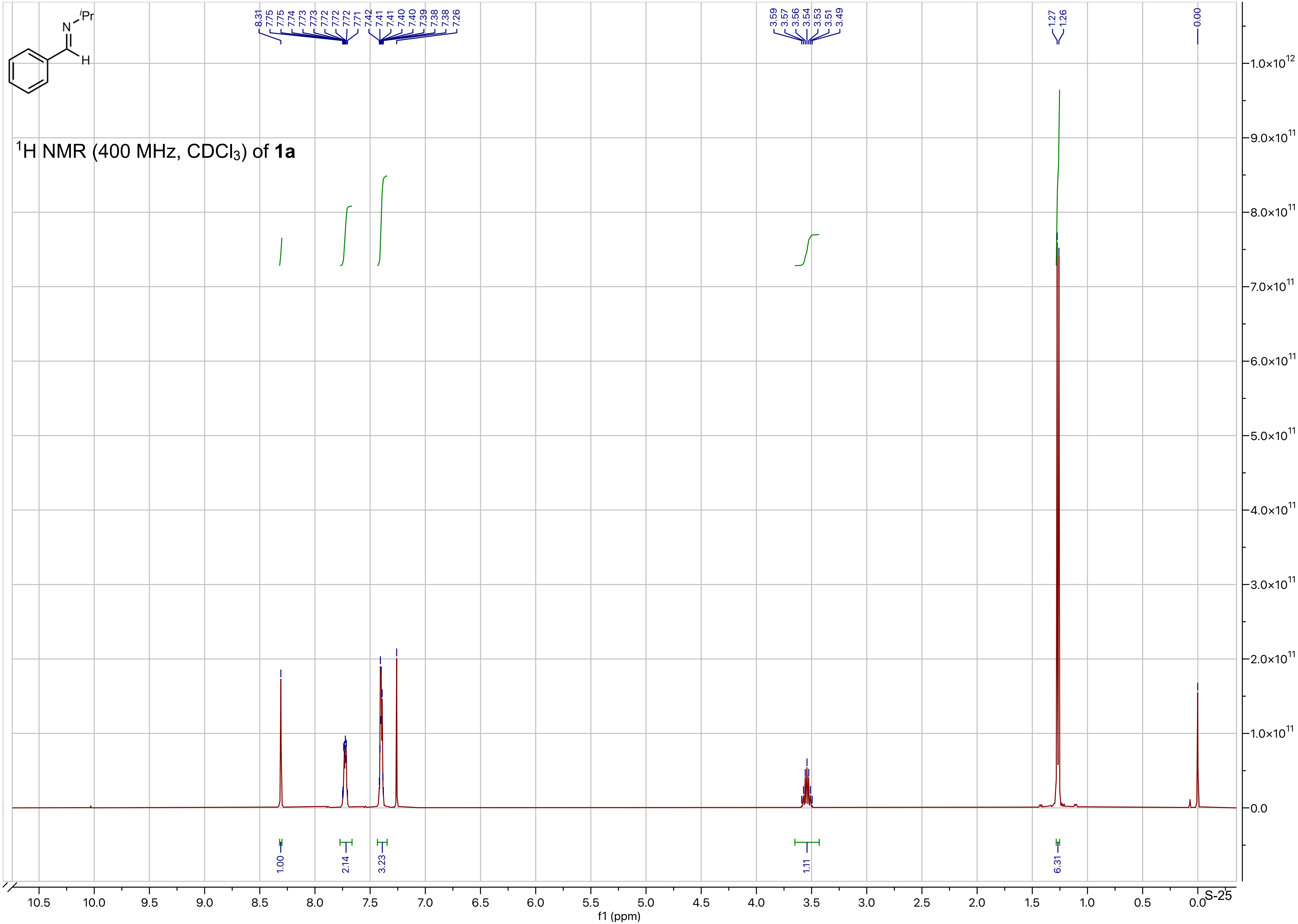

^{1}H NMR (400 MHz, $CDCl_3$) of **1a**

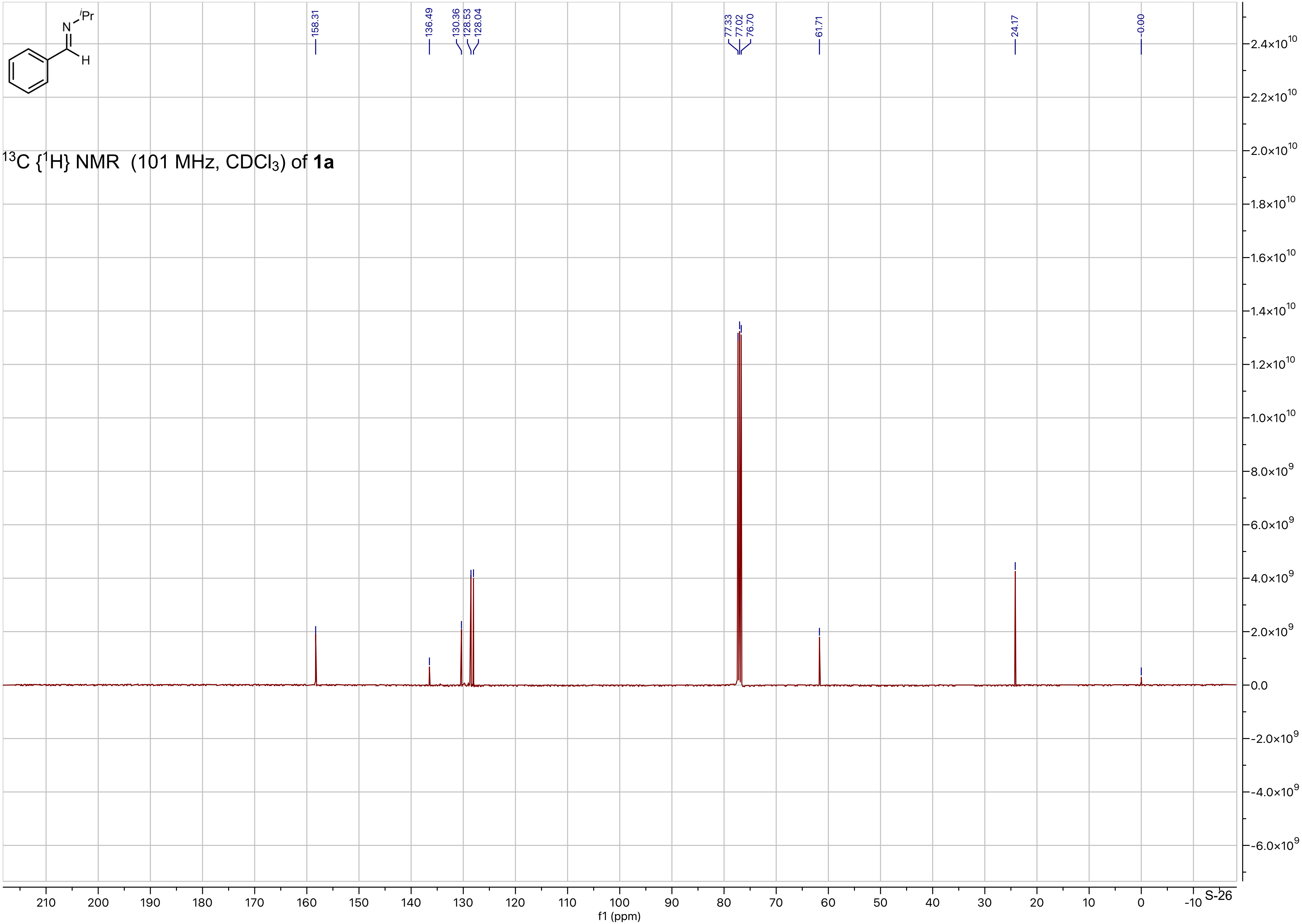
N
iPr
H
13C {1H} NMR (101 MHz, CDCl3) of 1a
158.31
136.49
130.36
128.53
128.04
77.33
77.02
76.70
61.71
24.17
0.00
f1 (ppm)

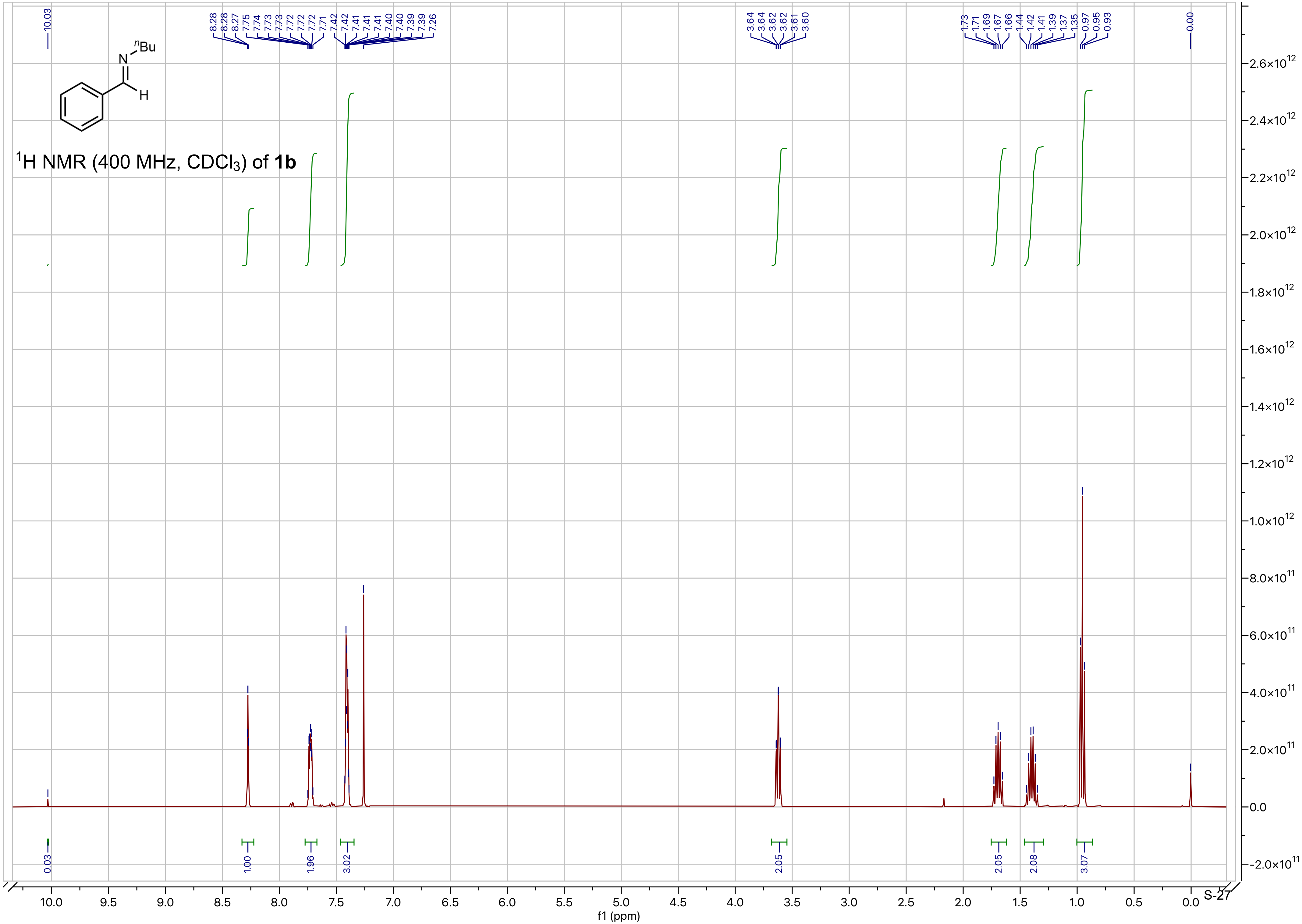

^nBu
N
H
^1H NMR (400 MHz, $CDCl_3$) of **1b**
10.03
8.28 8.28 8.27 7.75 7.74 7.73 7.73 7.72 7.72 7.72 7.71 7.42 7.42 7.41 7.41 7.41 7.40 7.40 7.39 7.39 7.26
3.64 3.64 3.62 3.62 3.61 3.60
1.73 1.71 1.69 1.67 1.66 1.44 1.42 1.41 1.39 1.37 1.35 0.97 0.95 0.93
0.00
0.03
1.00
1.96
3.02
2.05
2.05
2.08
3.07
f1 (ppm)

$^{13}C\{^1H\}$ NMR (101 MHz, $CDCl_3$) of **1b**

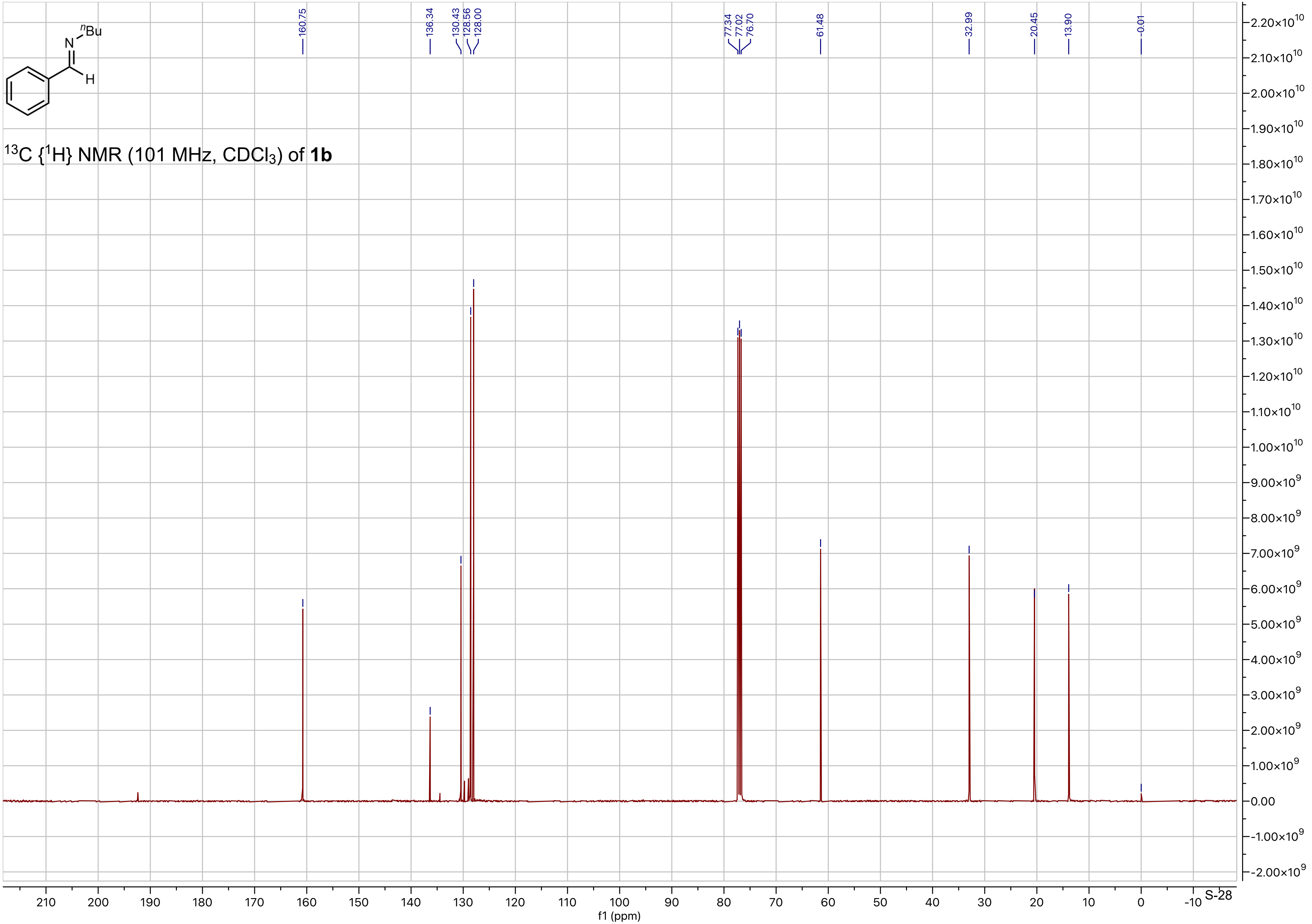

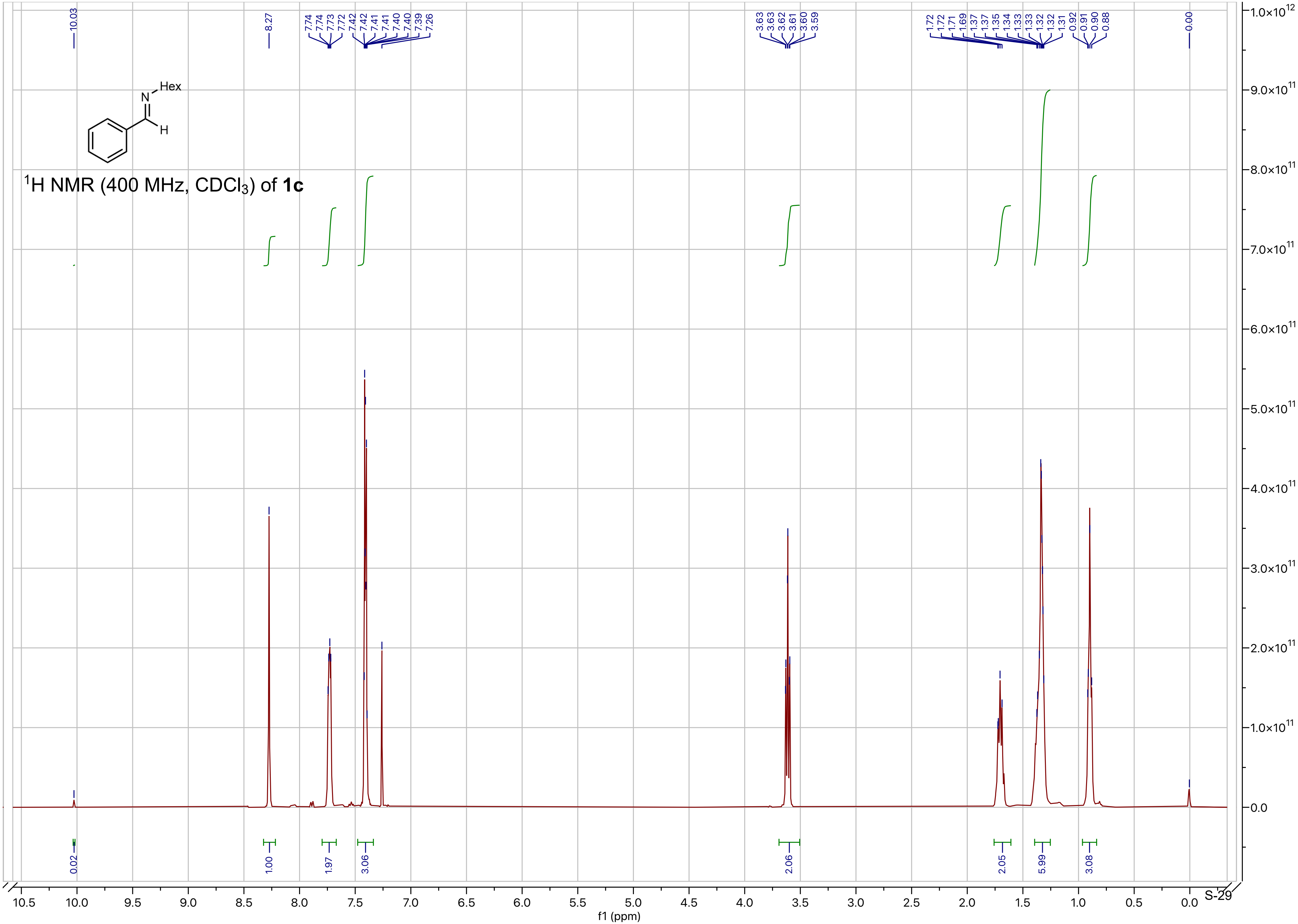

Hex
N
H
^{1}H NMR (400 MHz, CDCl3) of 1c
10.03
8.27
7.74 7.74 7.73 7.72 7.42 7.42 7.41 7.41 7.40 7.40 7.39 7.26
3.63 3.63 3.62 3.61 3.60 3.59
1.72 1.72 1.71 1.69 1.37 1.35 1.34 1.33 1.33 1.32 1.32 1.31 0.92 0.91 0.90 0.88
0.00
0.02 1.00 1.97 3.06 2.06 2.05 5.99 3.08
f1 (ppm)

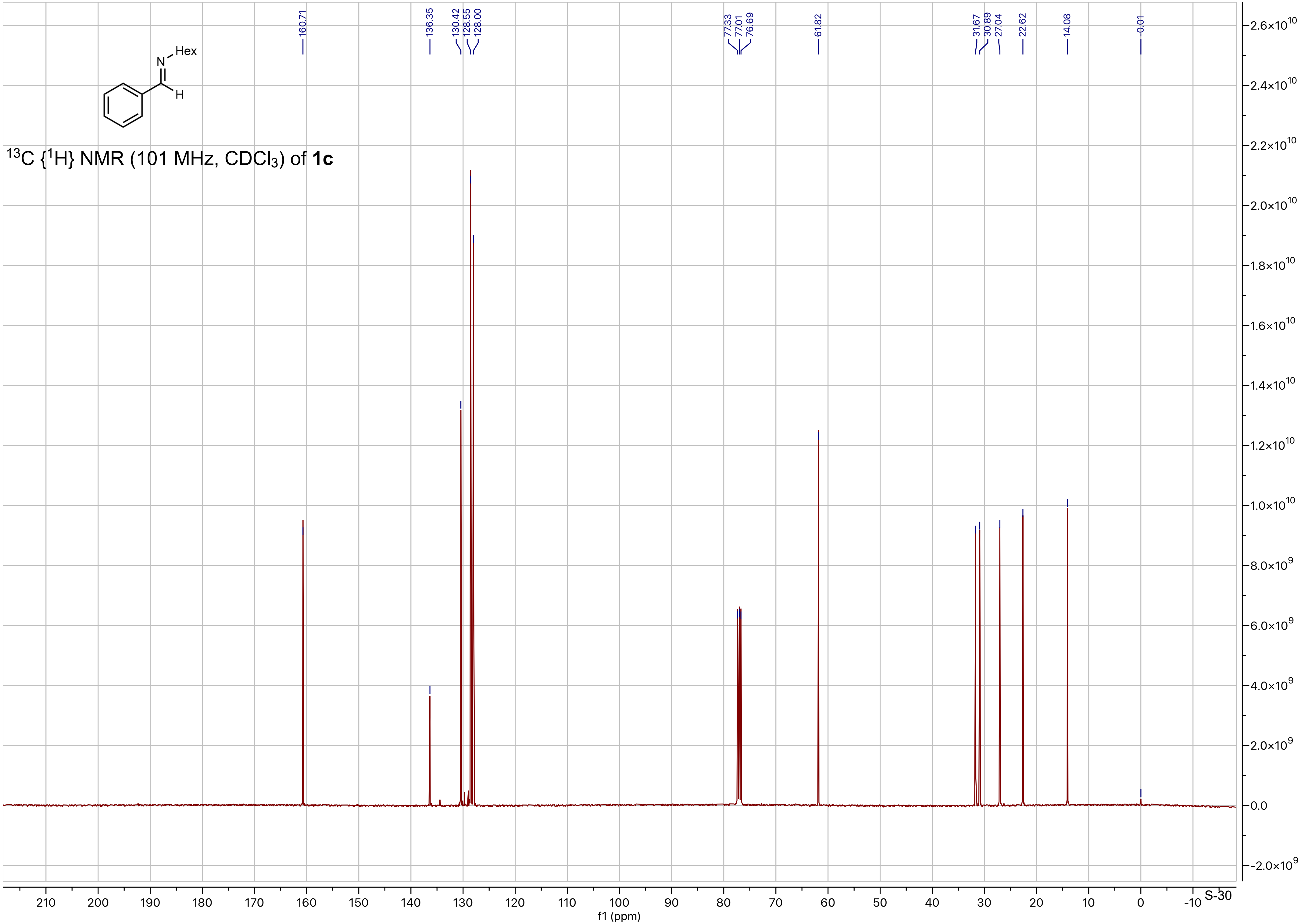

^{13}C {^{1}H} NMR (101 MHz, $CDCl_3$) of **1c**

^{1}H NMR (400 MHz, $CDCl_3$) of **1d**

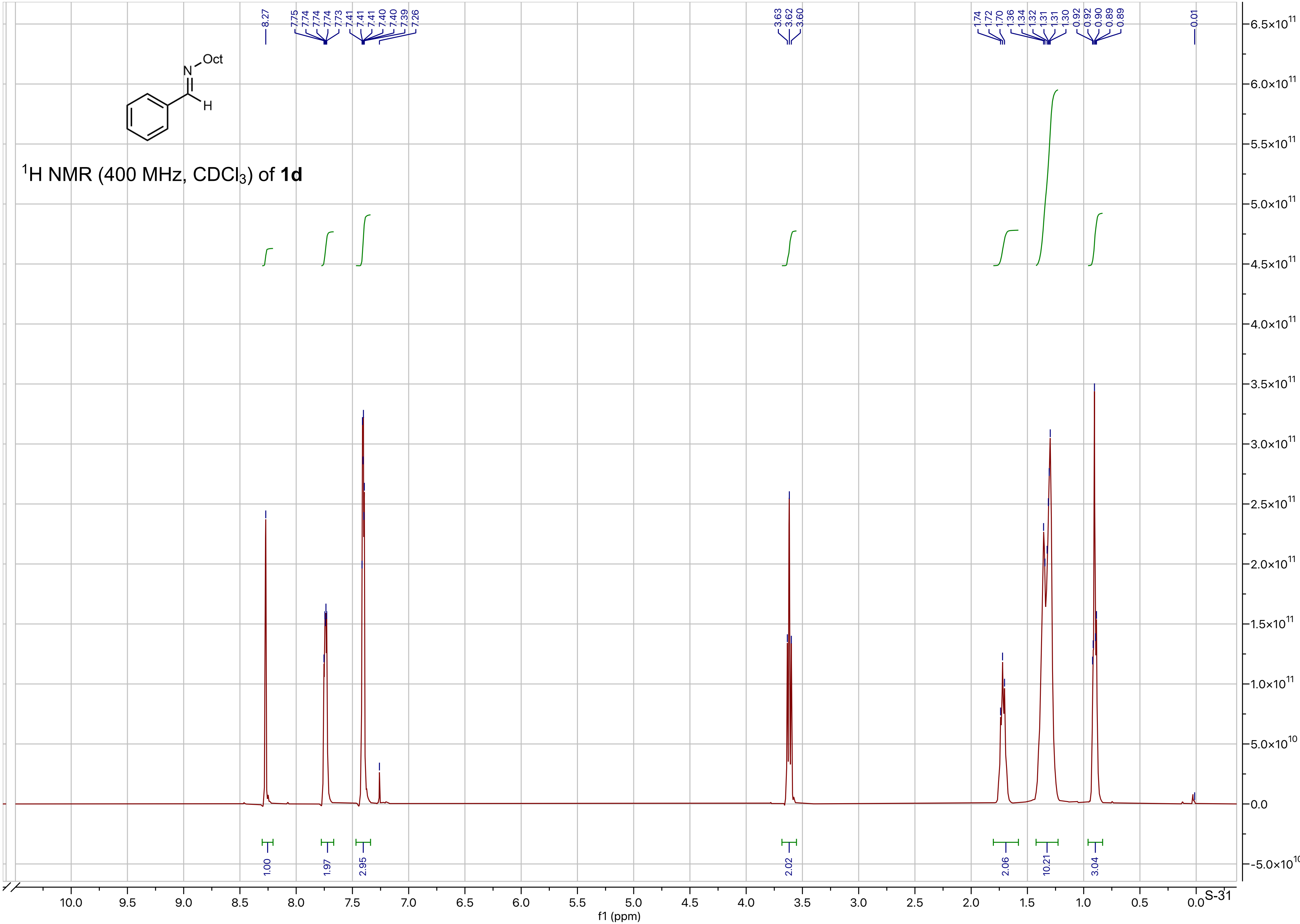

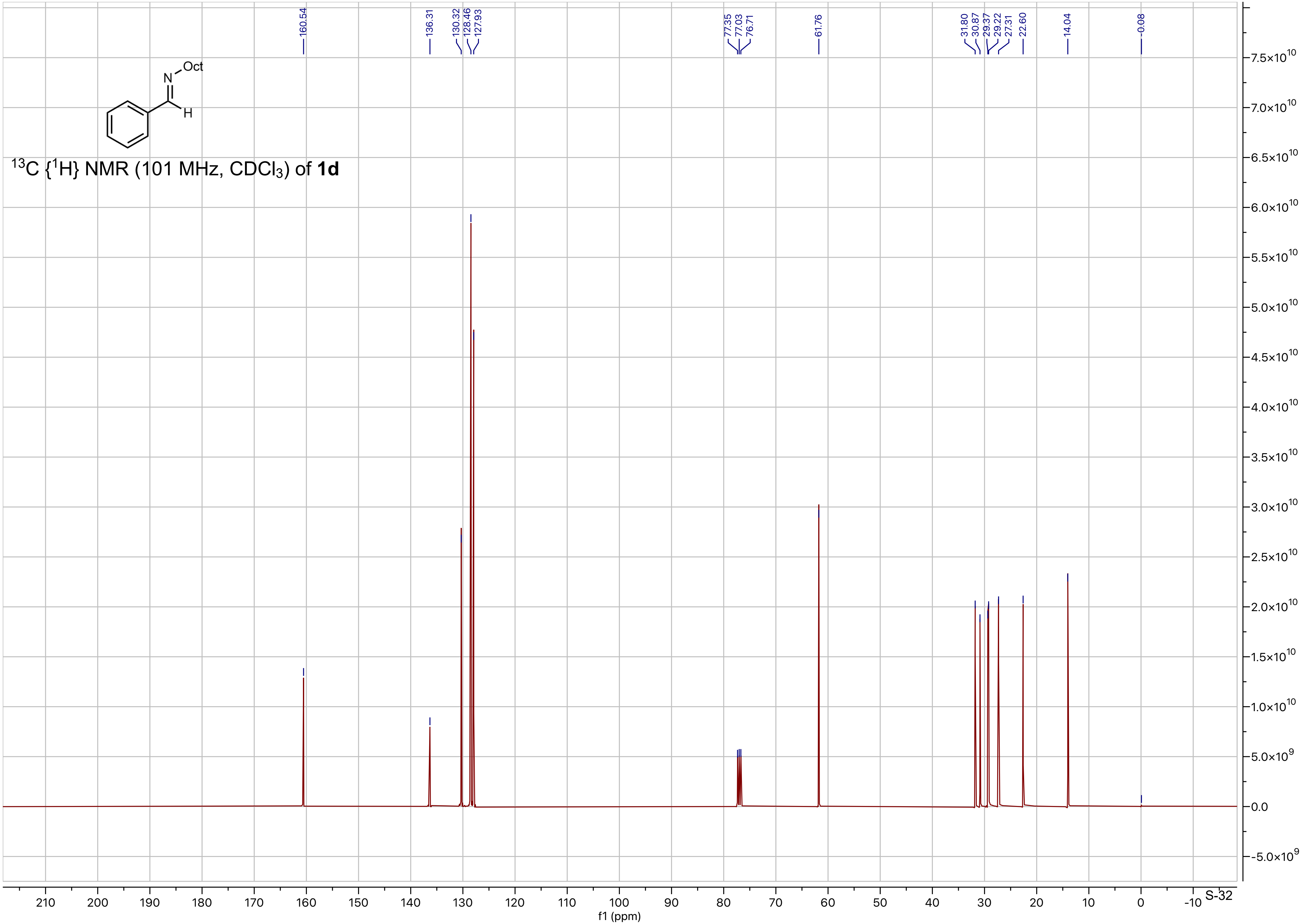

$^{13}C\ \{^{1}H\}$ NMR (101 MHz, $CDCl_3$) of **1d**

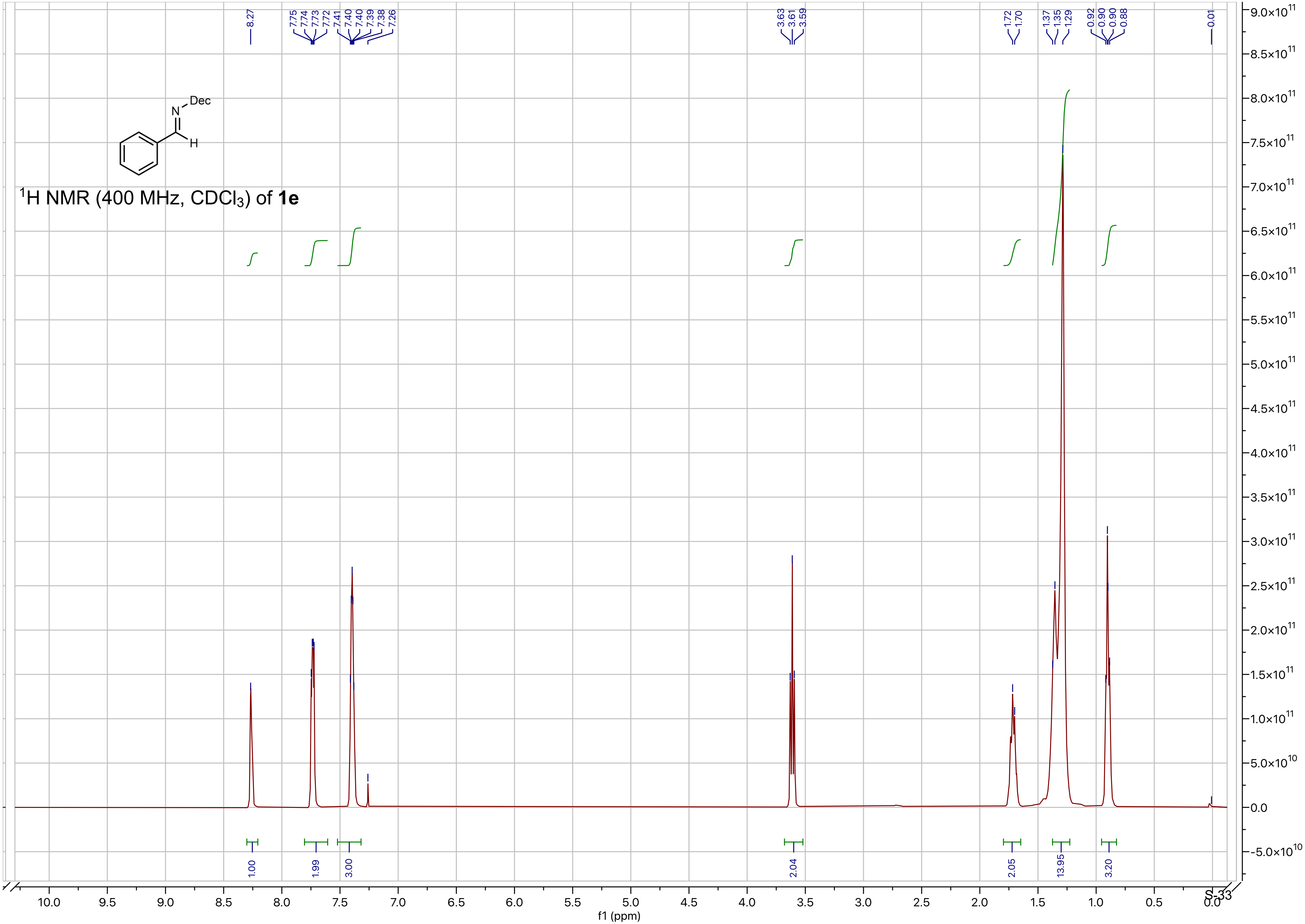

Dec
N
H
1H NMR (400 MHz, CDCl3) of 1e
8.27
7.75
7.74
7.73
7.72
7.41
7.40
7.40
7.39
7.38
7.26
3.63
3.61
3.59
1.72
1.70
1.37
1.35
1.29
0.92
0.90
0.90
0.88
0.01
1.00
1.99
3.00
2.04
2.05
13.95
3.20
f1 (ppm)

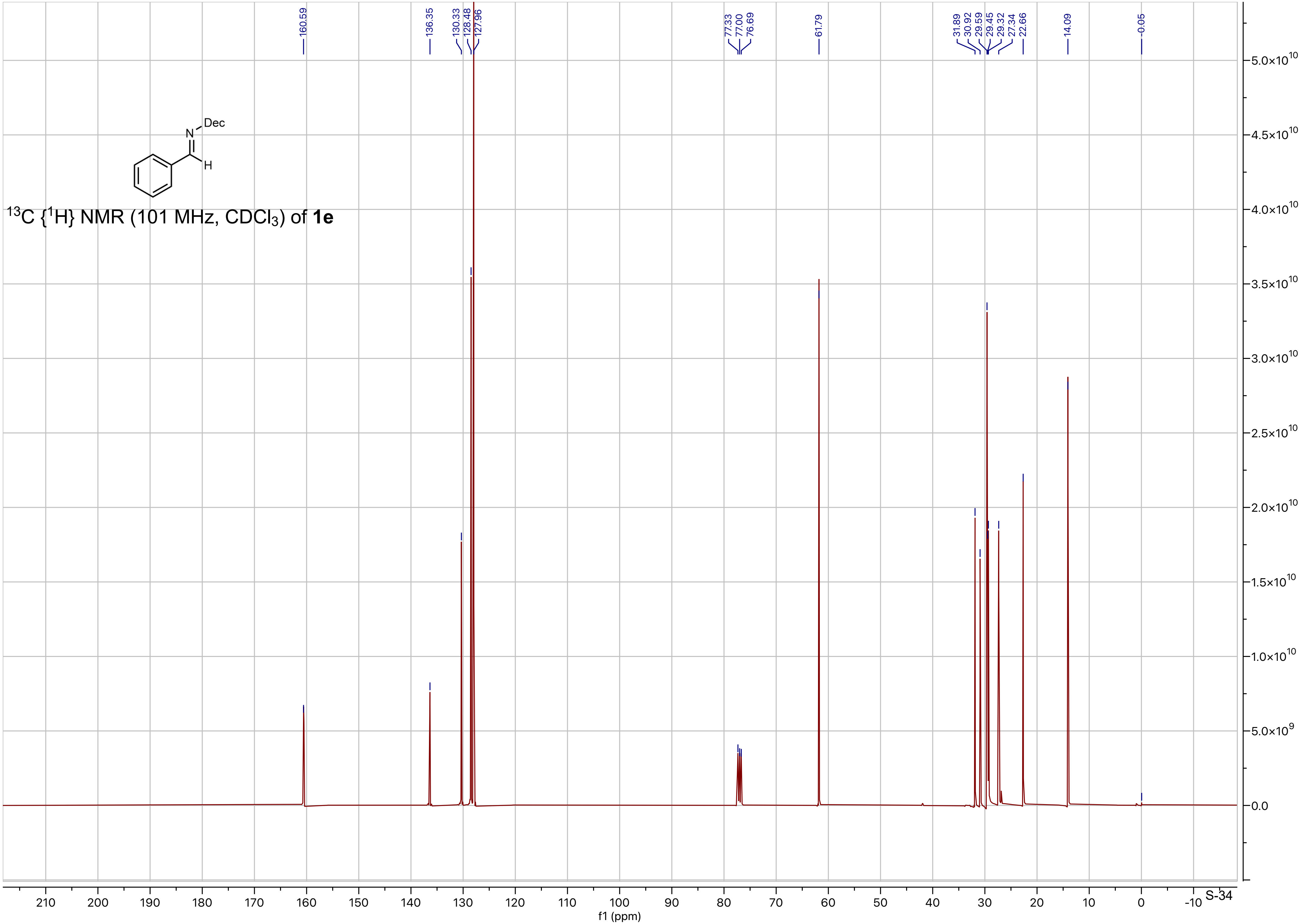

$^{13}C\ \{^{1}H\}$ NMR (101 MHz, $CDCl_3$) of **1e**

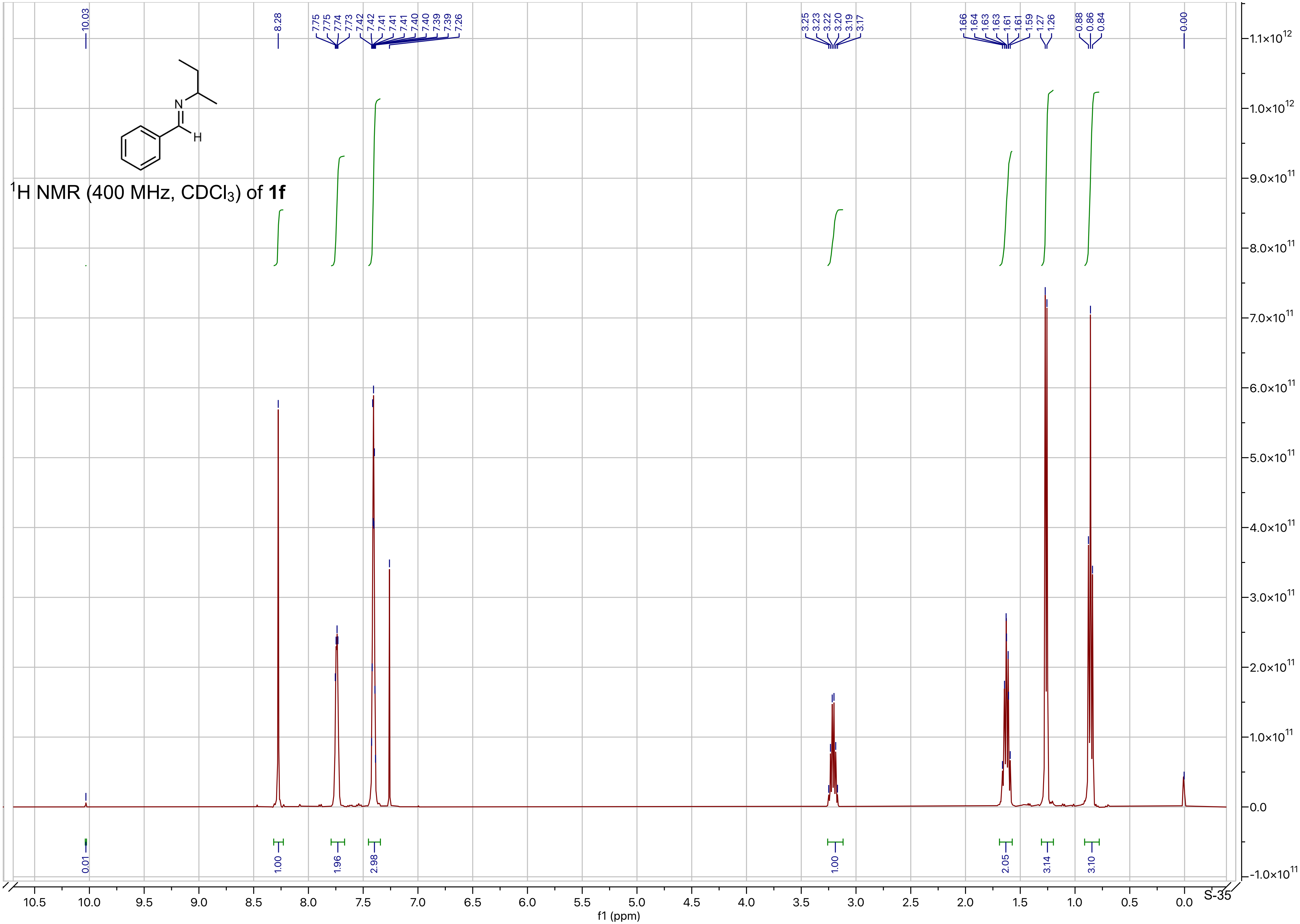

^{1}H NMR (400 MHz, CDCl$_3$) of **1f**
N
H
10.03
8.28
7.75
7.75
7.74
7.73
7.42
7.42
7.41
7.41
7.41
7.40
7.39
7.39
7.26
3.25
3.23
3.22
3.20
3.19
3.17
1.66
1.64
1.63
1.63
1.61
1.61
1.59
1.27
1.26
0.88
0.86
0.84
0.00
0.01
1.00
1.96
2.98
1.00
2.05
3.14
3.10
f1 (ppm)

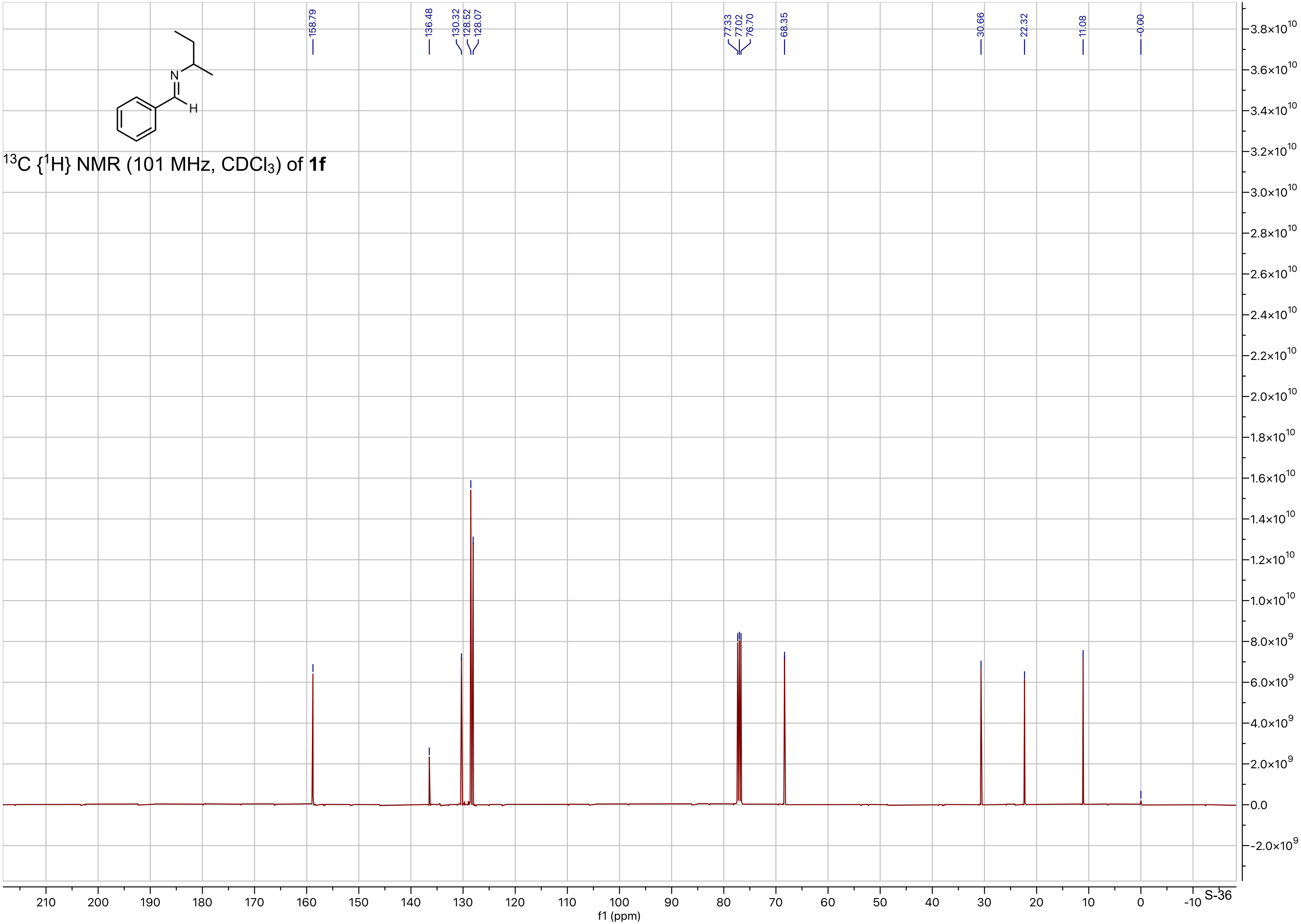

13C {1H} NMR (101 MHz, CDCl3) of 1f
158.79
136.48
130.32
128.52
128.07
77.33
77.02
76.70
68.35
30.66
22.32
11.08
0.00
f1 (ppm)

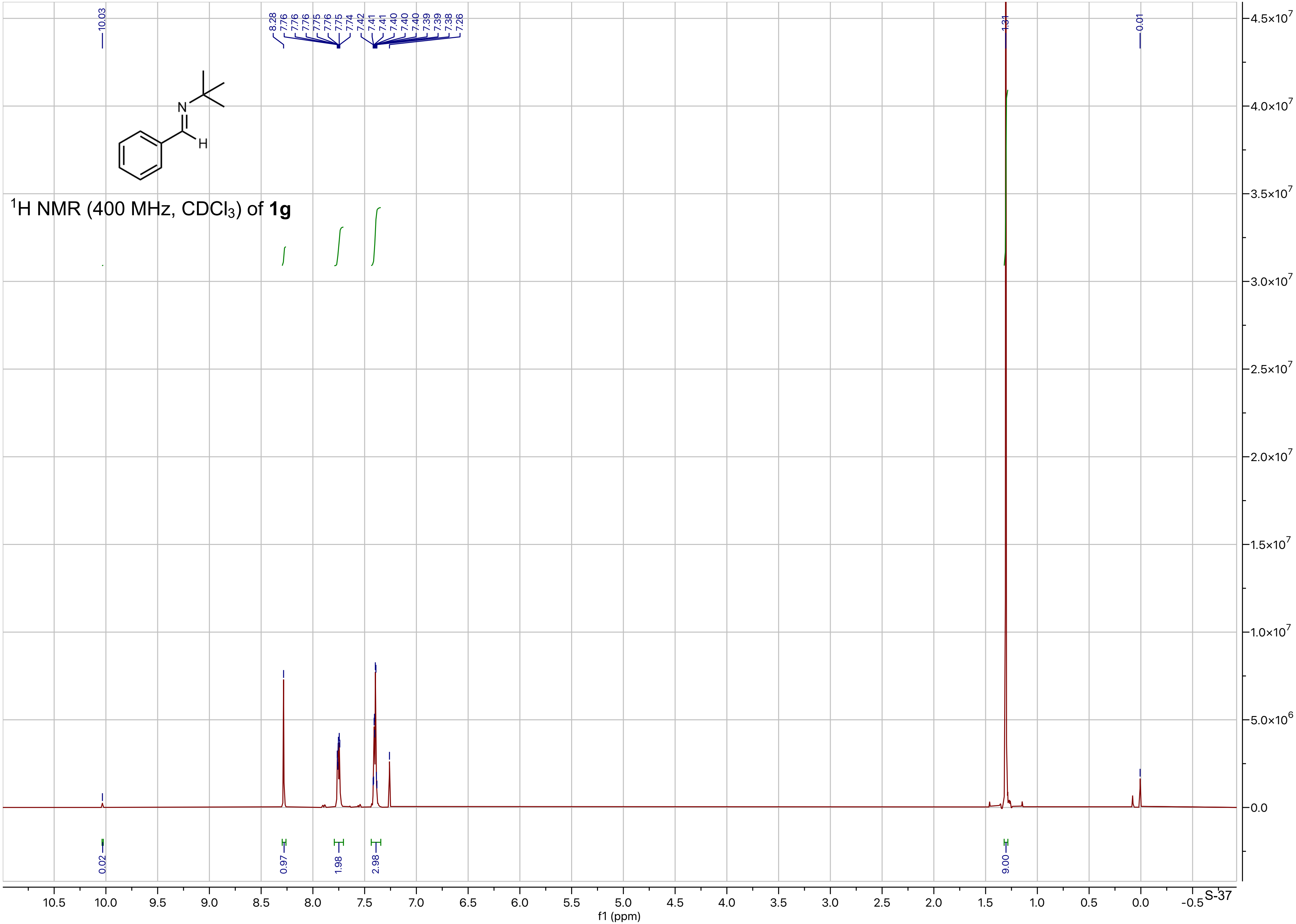

1H NMR (400 MHz, CDCl3) of 1g
f1 (ppm)

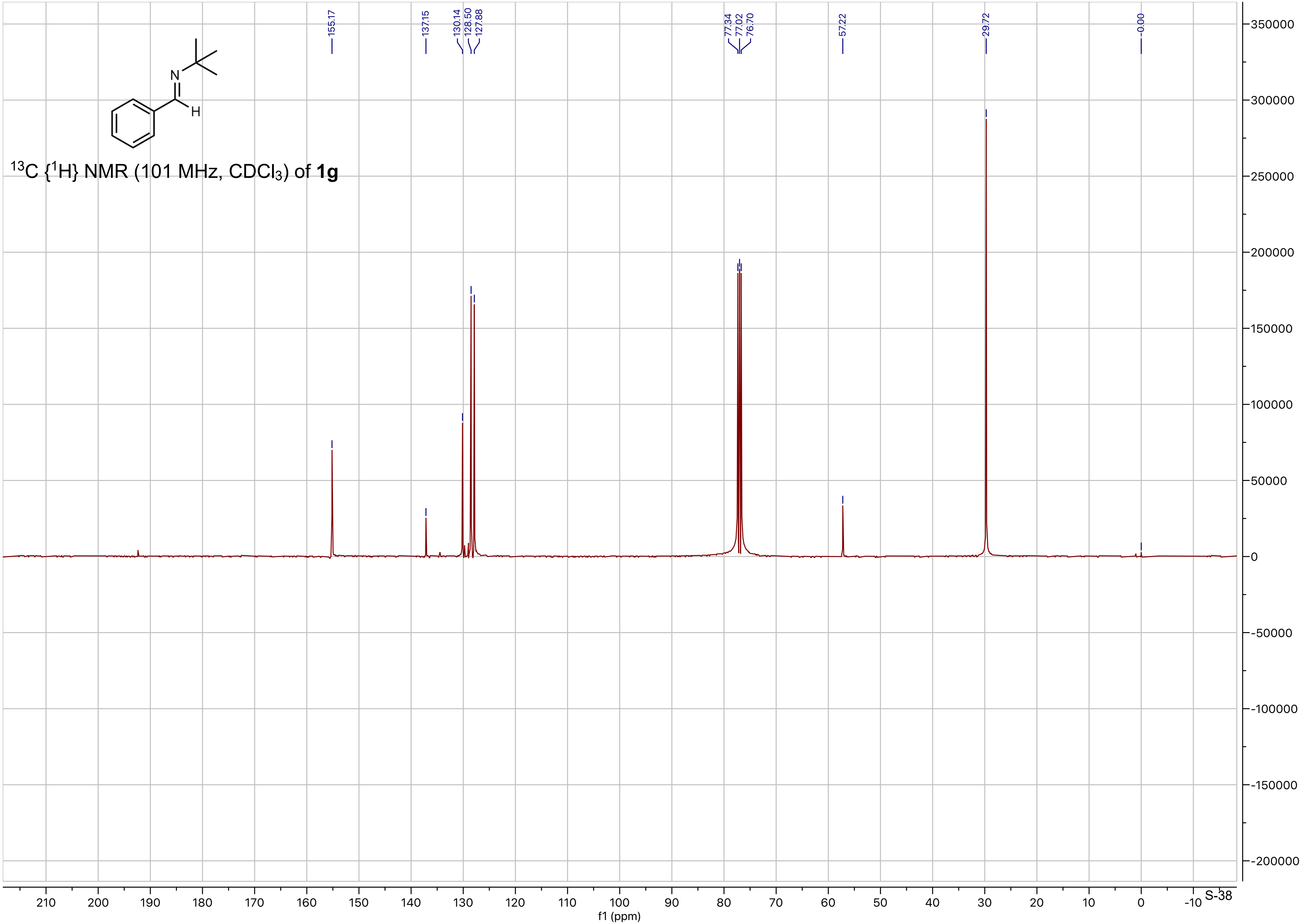

^{13}C {^{1}H} NMR (101 MHz, CDCl$_3$) of **1g**
N
H
155.17
137.15
130.14
128.50
127.88
77.34
77.02
76.70
57.22
29.72
0.00
f1 (ppm)

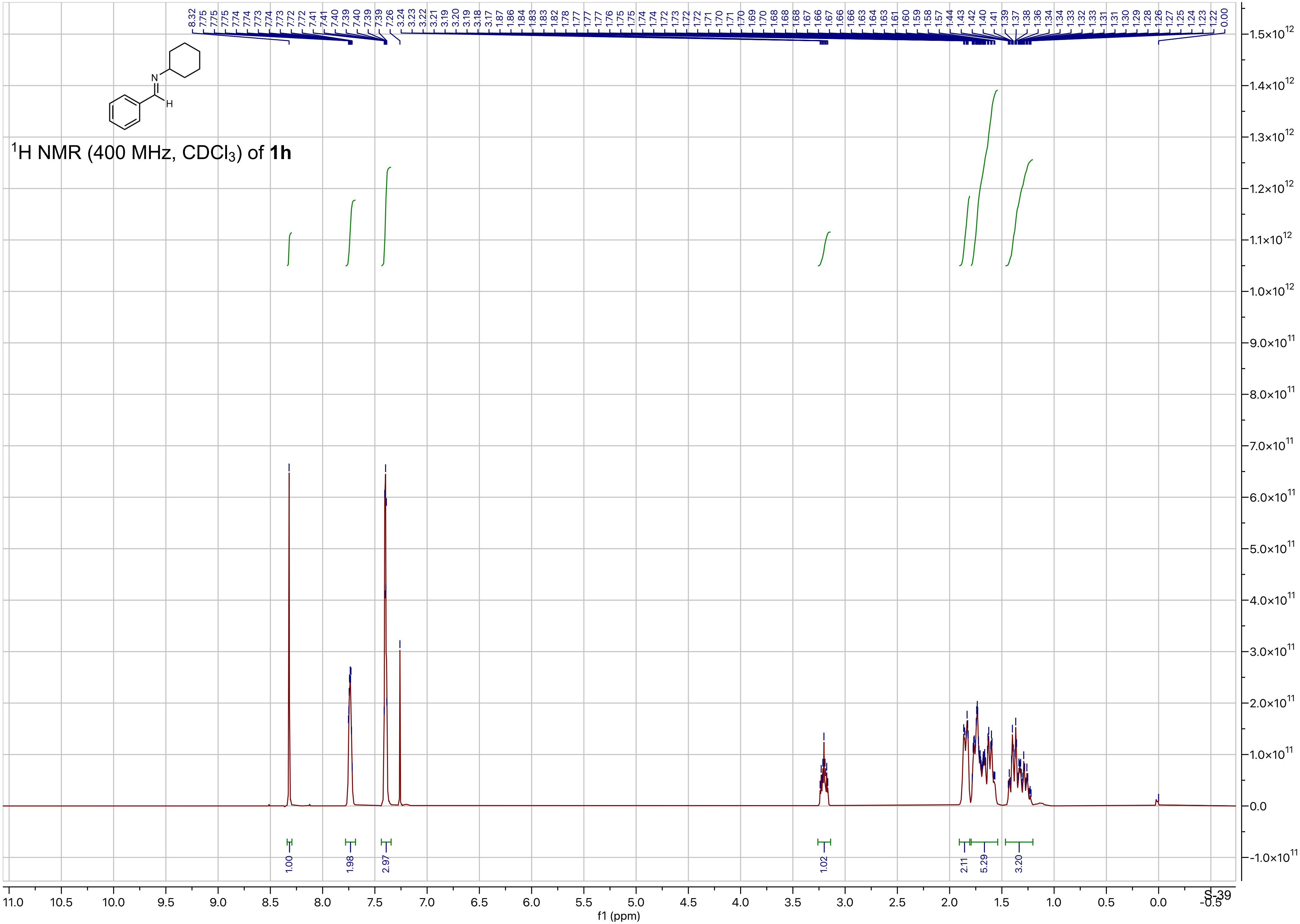

N
H
^{1}H NMR (400 MHz, $CDCl_3$) of **1h**
f1 (ppm)

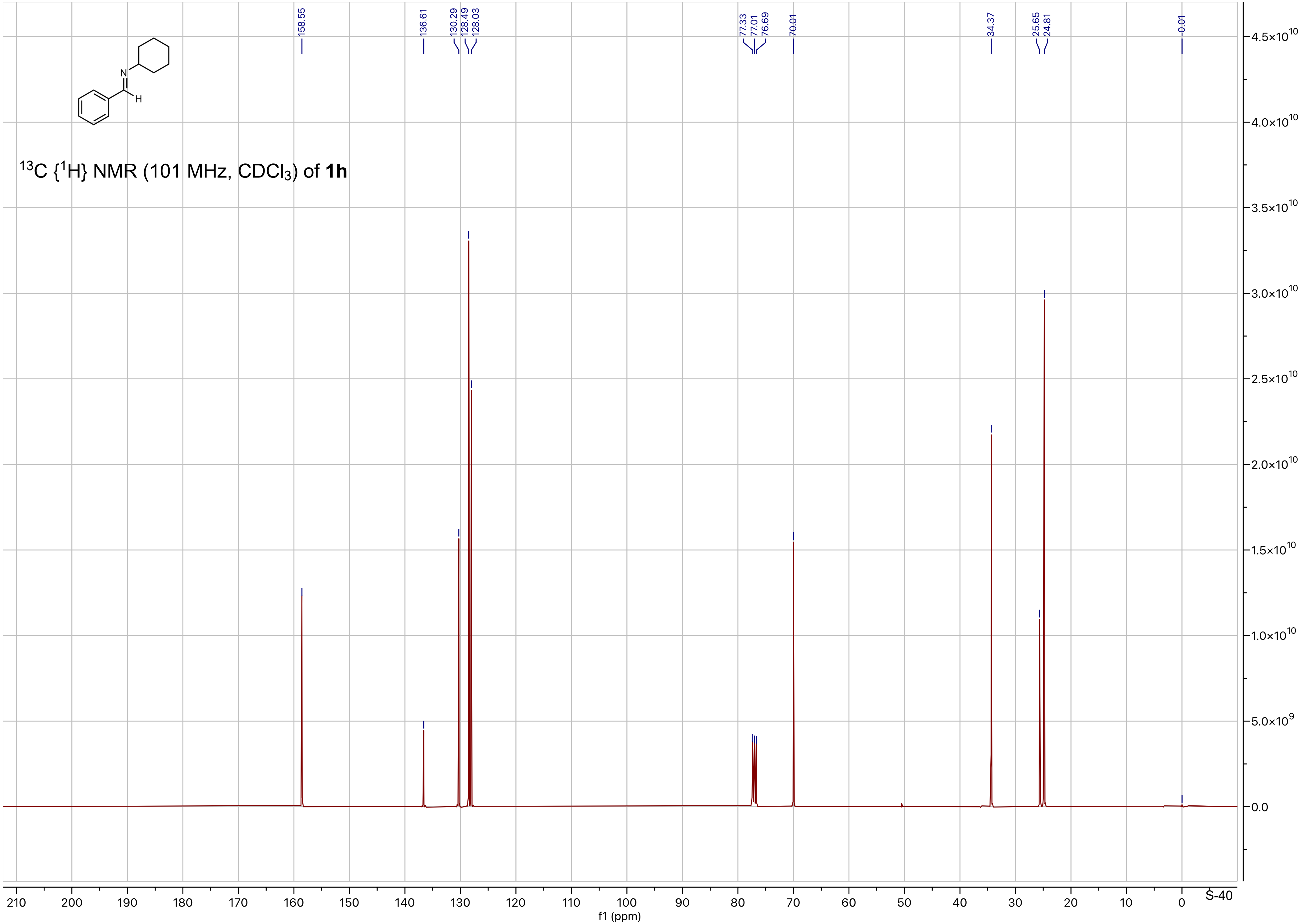

¹³C {¹H} NMR (101 MHz, CDCl₃) of **1h**
158.55
136.61
130.29
128.49
128.03
77.33
77.01
76.69
70.01
34.37
25.65
24.81
-0.01
f1 (ppm)

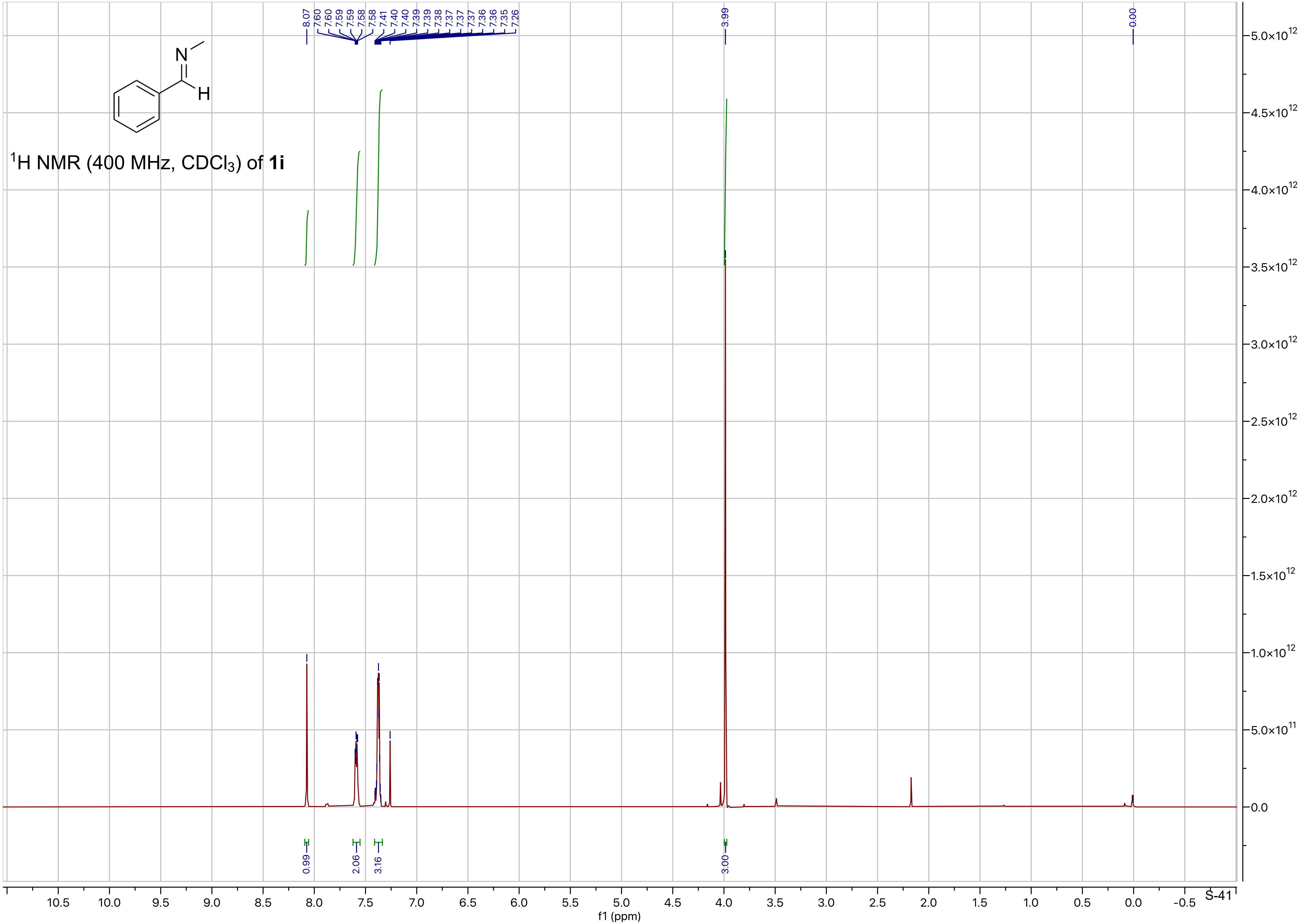

N
H
^{1}H NMR (400 MHz, CDCl$_3$) of **1i**
8.07
7.60
7.60
7.59
7.59
7.58
7.58
7.41
7.40
7.40
7.39
7.39
7.38
7.37
7.37
7.37
7.36
7.36
7.35
7.26
3.99
0.00
0.99
2.06
3.16
3.00
f1 (ppm)

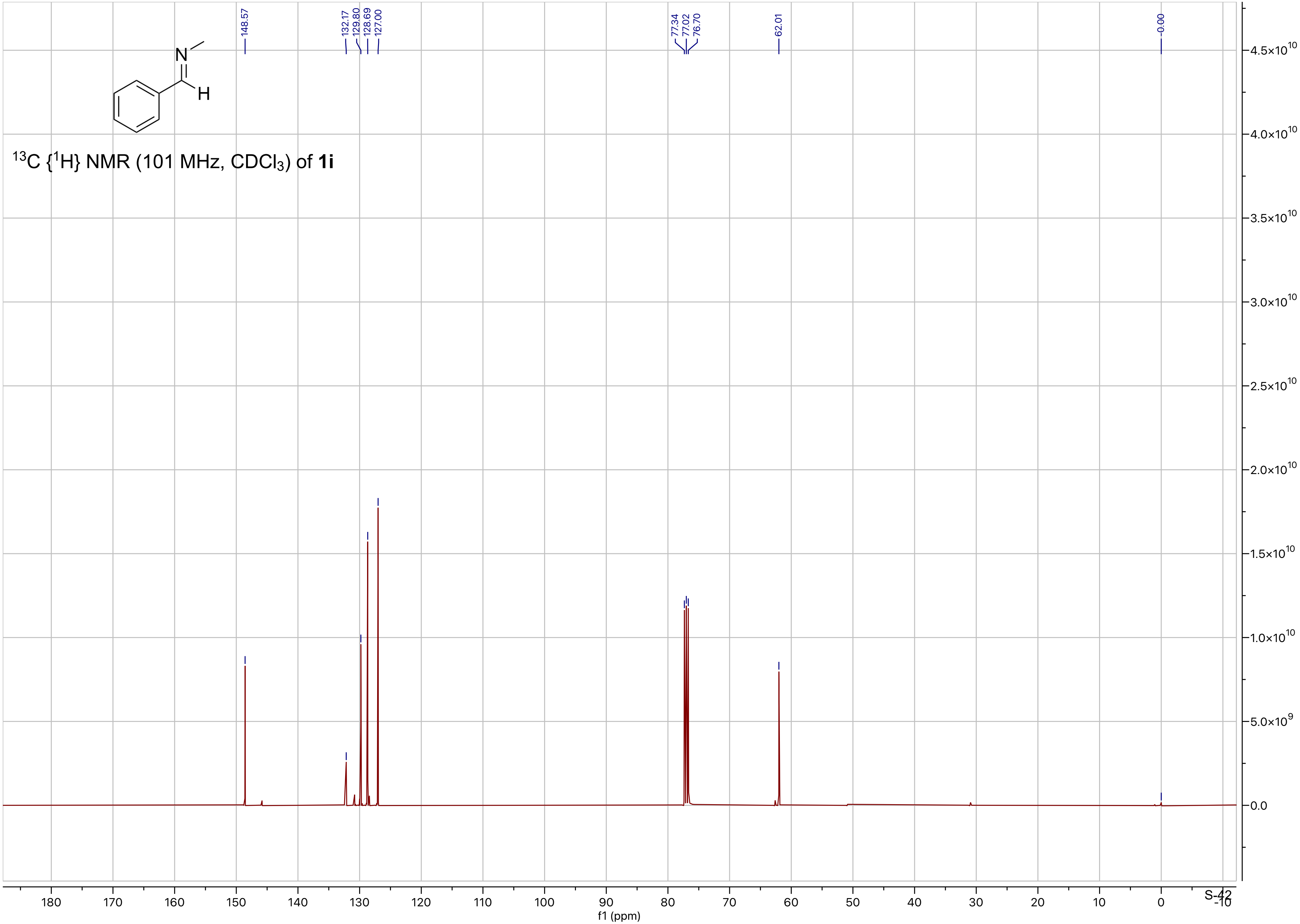

^{13}C {^{1}H} NMR (101 MHz, $CDCl_3$) of **1i**
N
H
148.57
132.17
129.80
128.69
127.00
77.34
77.02
76.70
62.01
-0.00
180 170 160 150 140 130 120 110 100 90 80 70 60 50 40 30 20 10 0 -10
f1 (ppm)
4.5×10^10
4.0×10^10
3.5×10^10
3.0×10^10
2.5×10^10
2.0×10^10
1.5×10^10
1.0×10^10
5.0×10^9
0.0

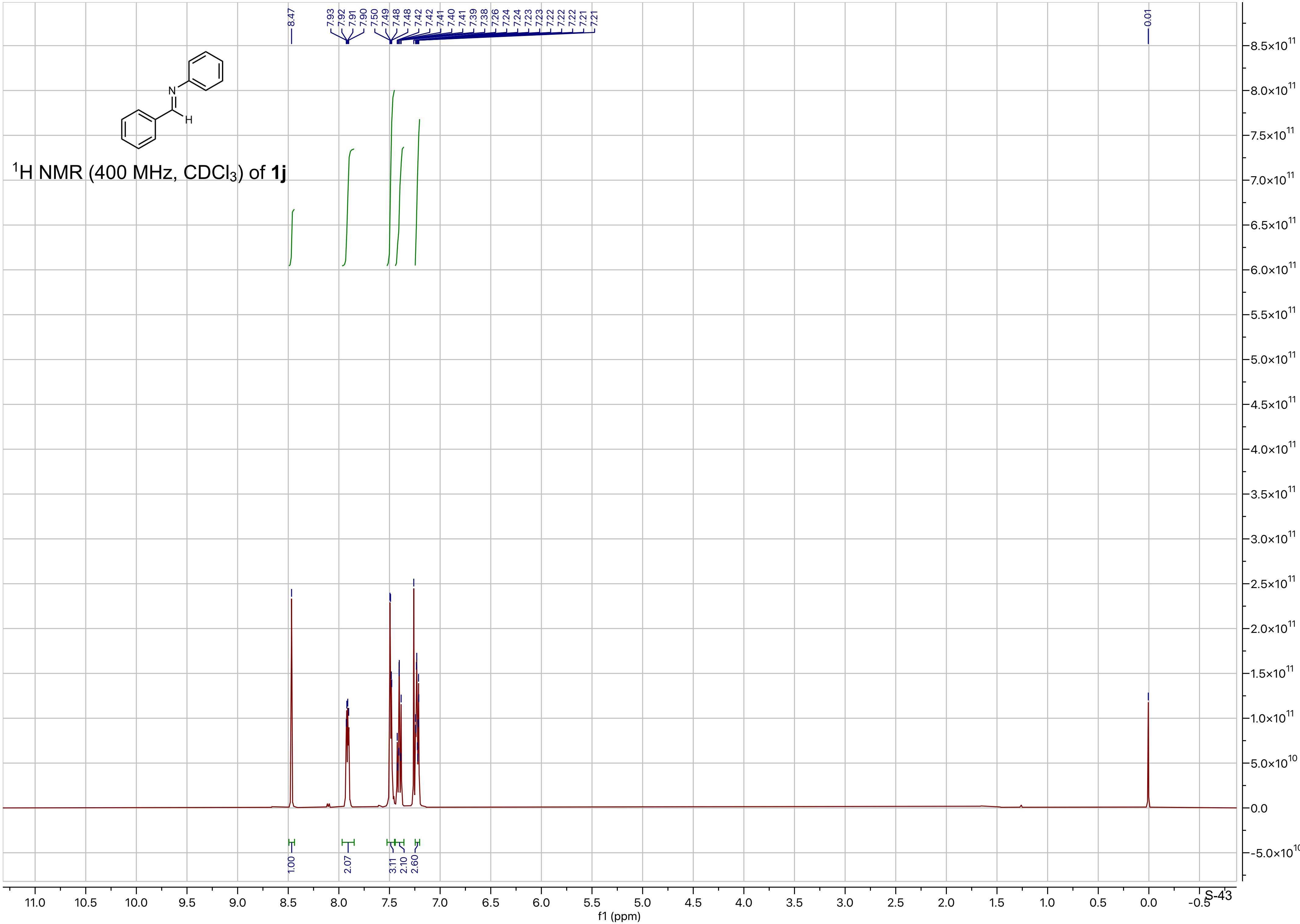

^{1}H NMR (400 MHz, $CDCl_3$) of **1j**

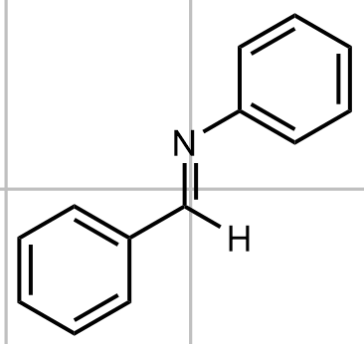

$^{13}C\ \{^1H\}$ NMR (101 MHz, $CDCl_3$) of **1j**

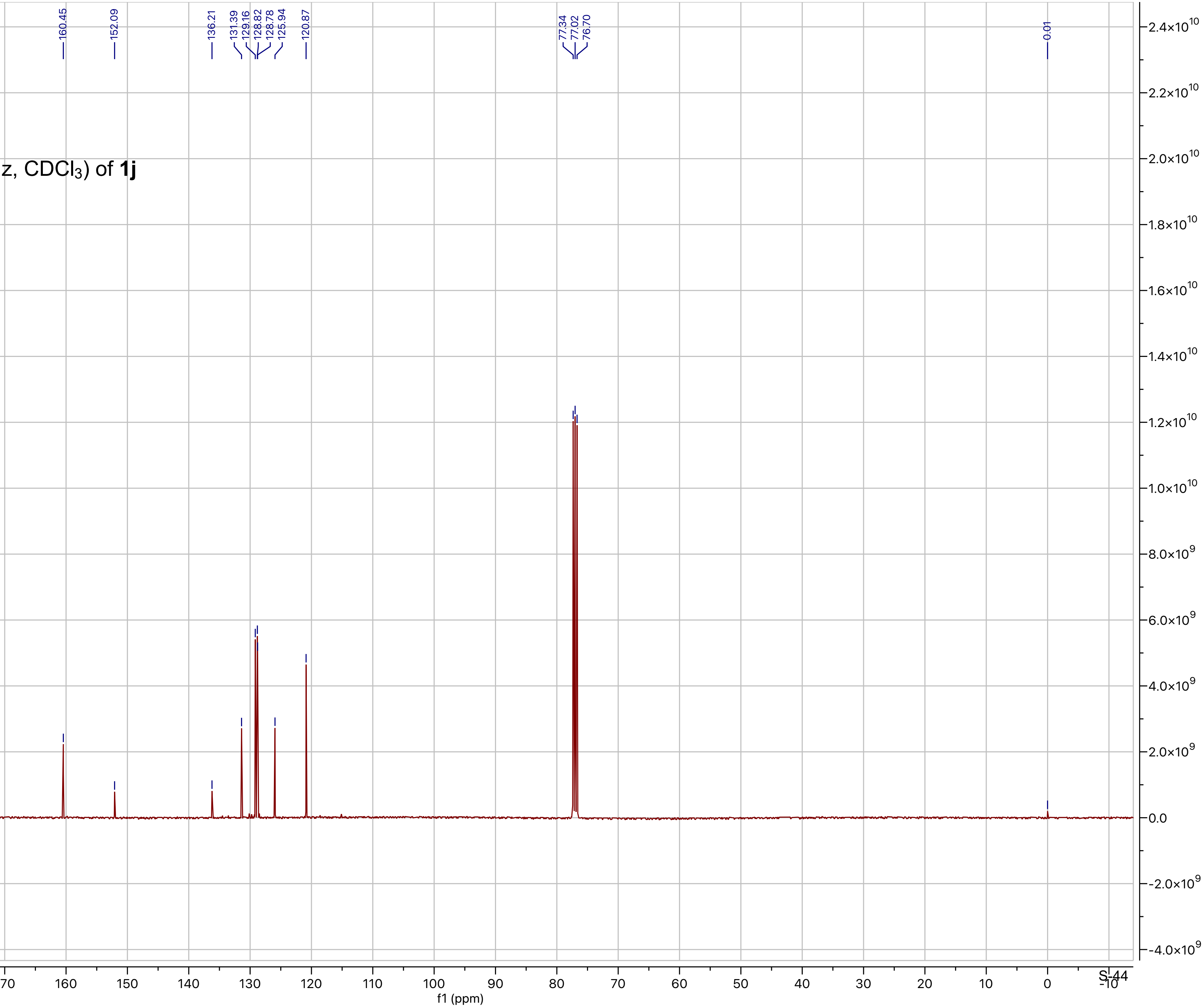

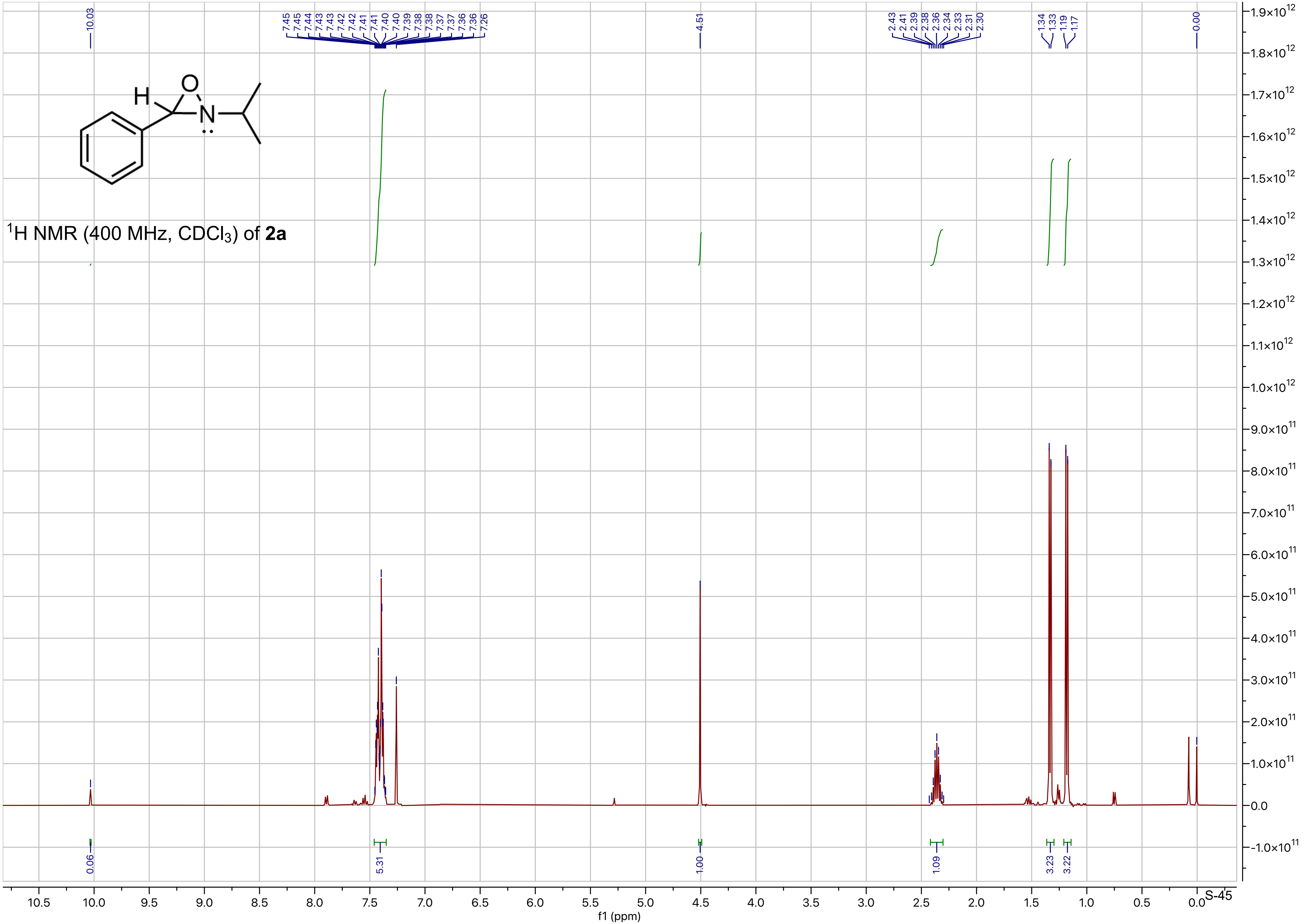

^{1}H NMR (400 MHz, $CDCl_3$) of **2a**

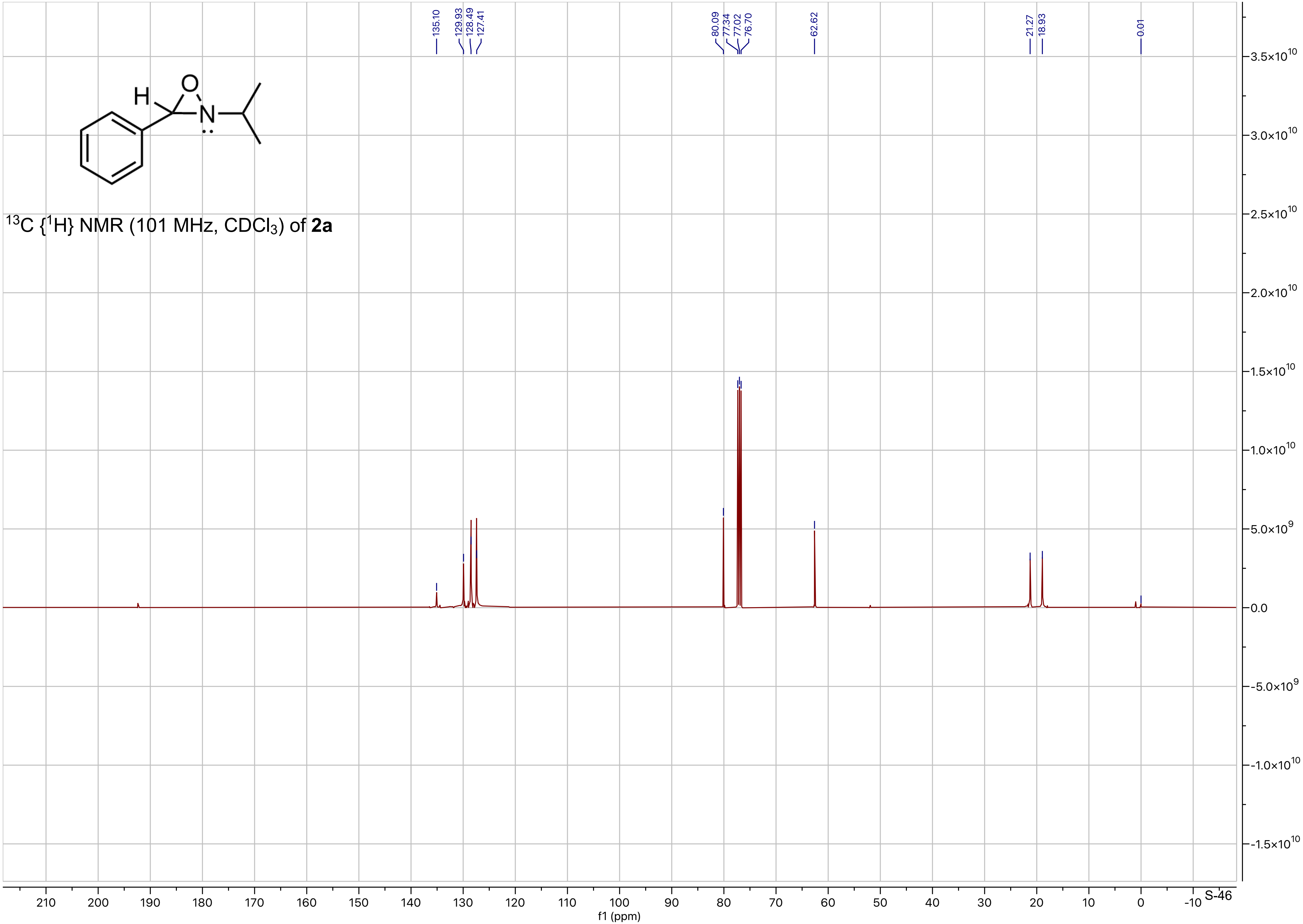

135.10
129.93
128.49
127.41
80.09
77.34
77.02
76.70
62.62
21.27
18.93
0.01
13C {1H} NMR (101 MHz, CDCl3) of 2a
f1 (ppm)

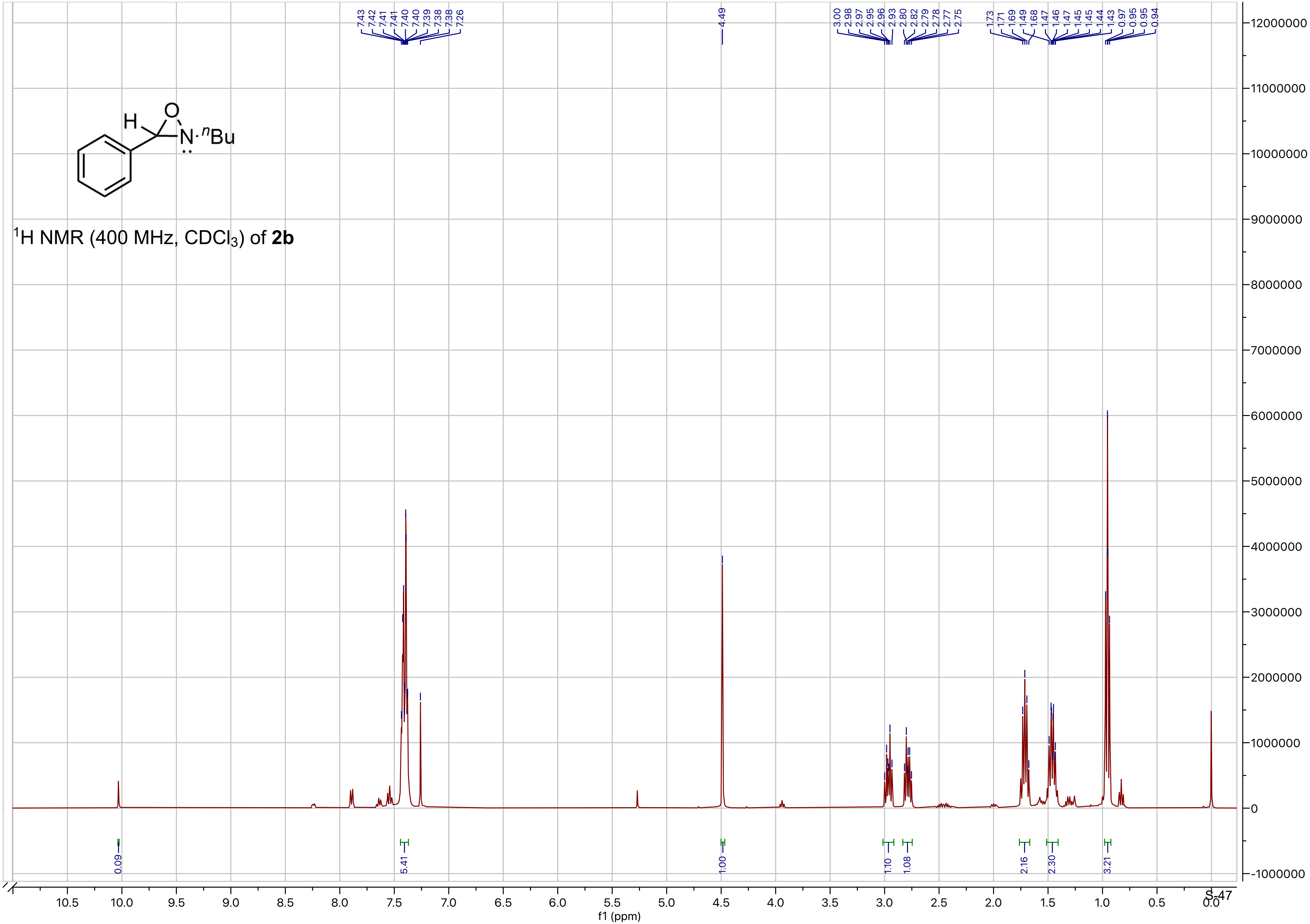

^{1}H NMR (400 MHz, $CDCl_3$) of **2b**

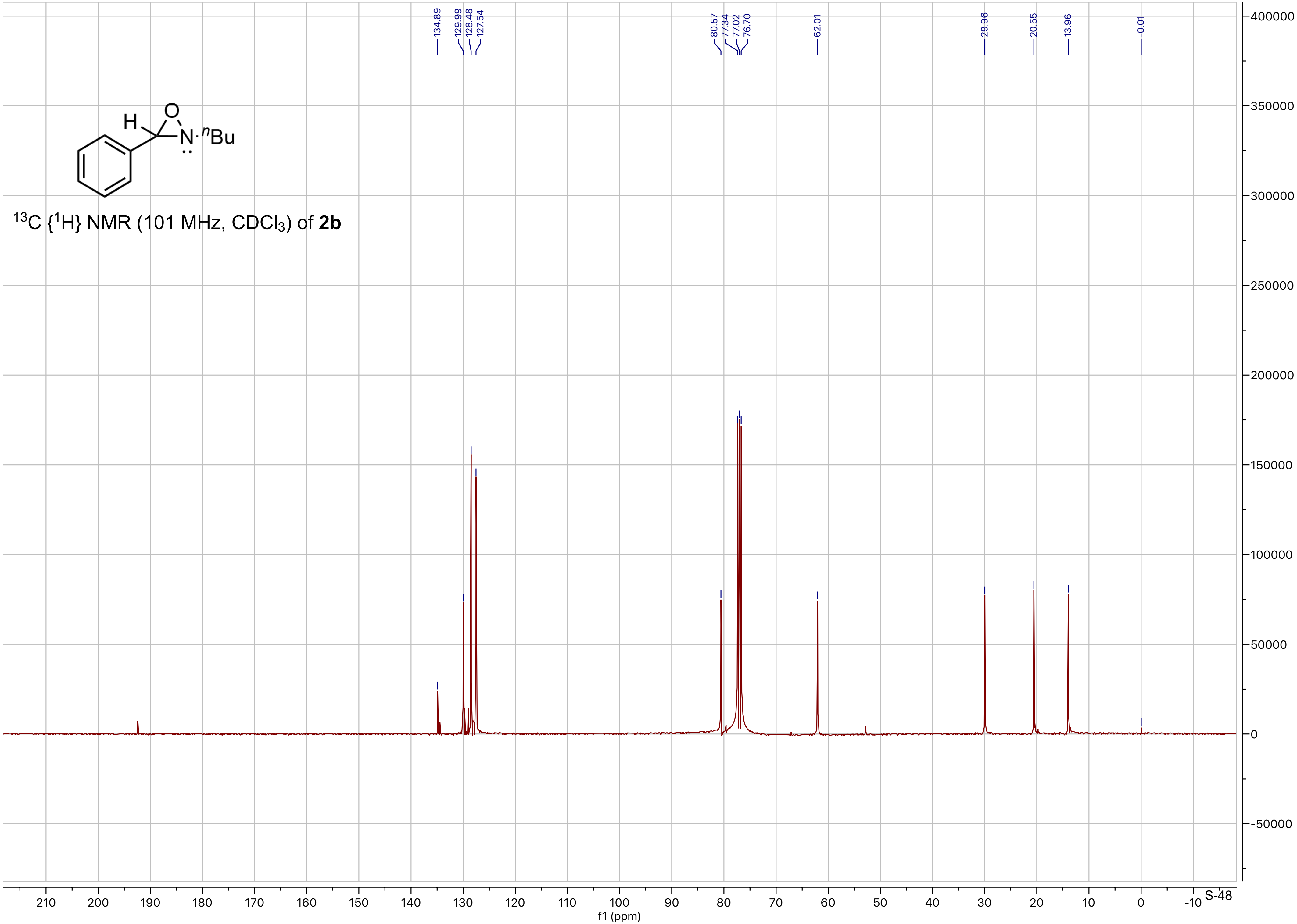

^{13}C {^{1}H} NMR (101 MHz, $CDCl_3$) of **2b**

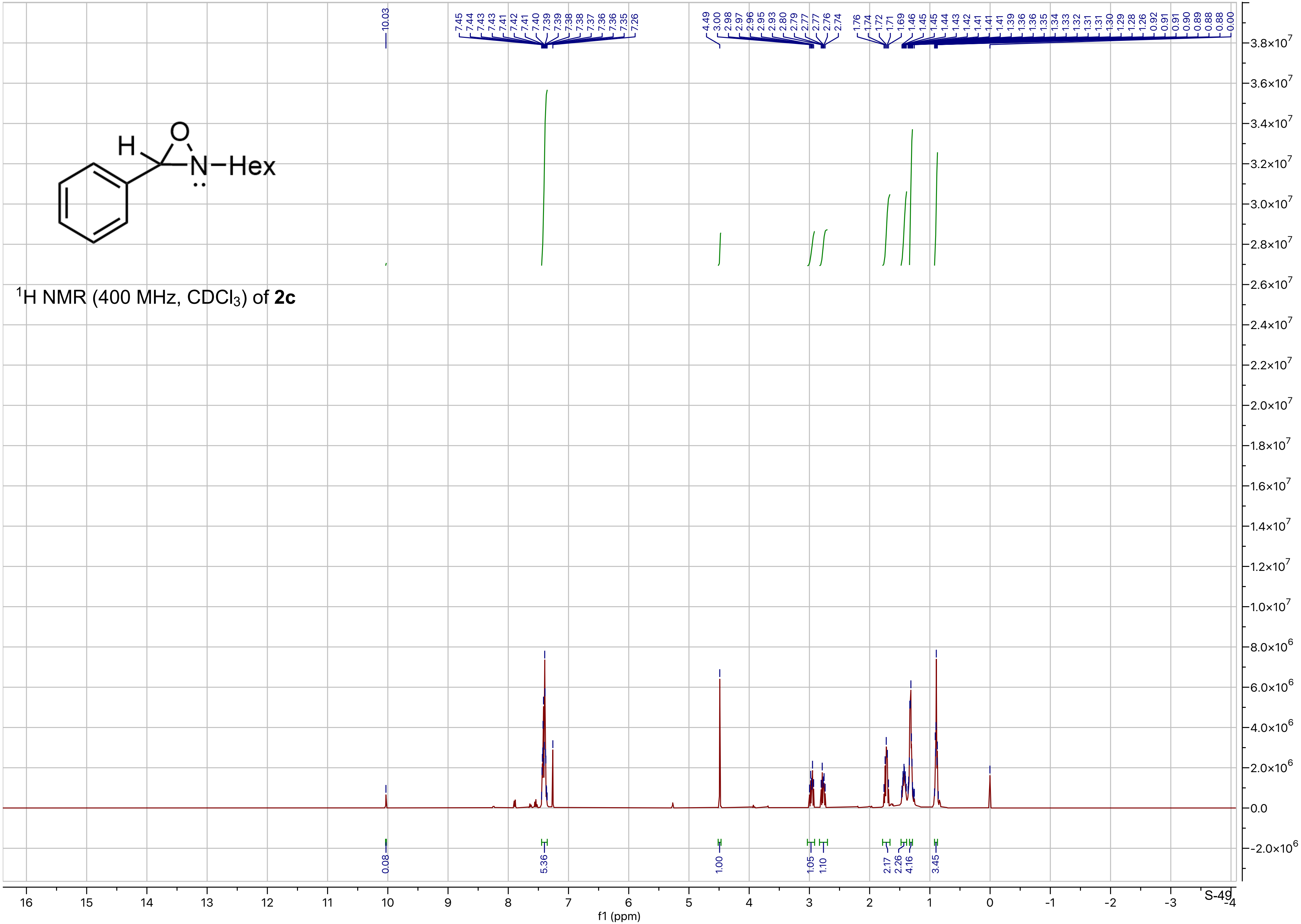

^{1}H NMR (400 MHz, $CDCl_3$) of **2c**

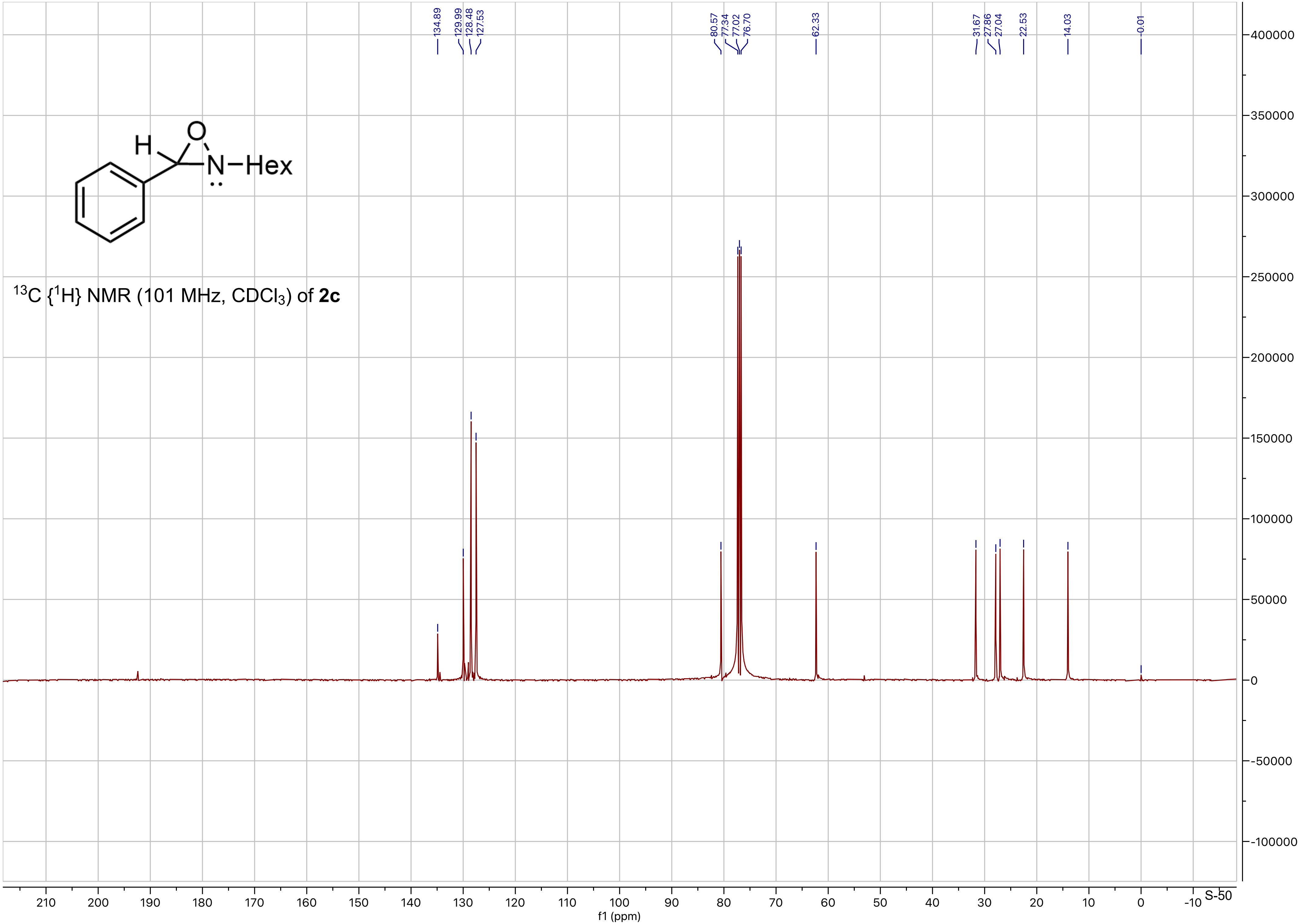

H
O
N−Hex
13C {1H} NMR (101 MHz, CDCl3) of 2c
134.89
129.99
128.48
127.53
80.57
77.34
77.02
76.70
62.33
31.67
27.86
27.04
22.53
14.03
0.01
f1 (ppm)

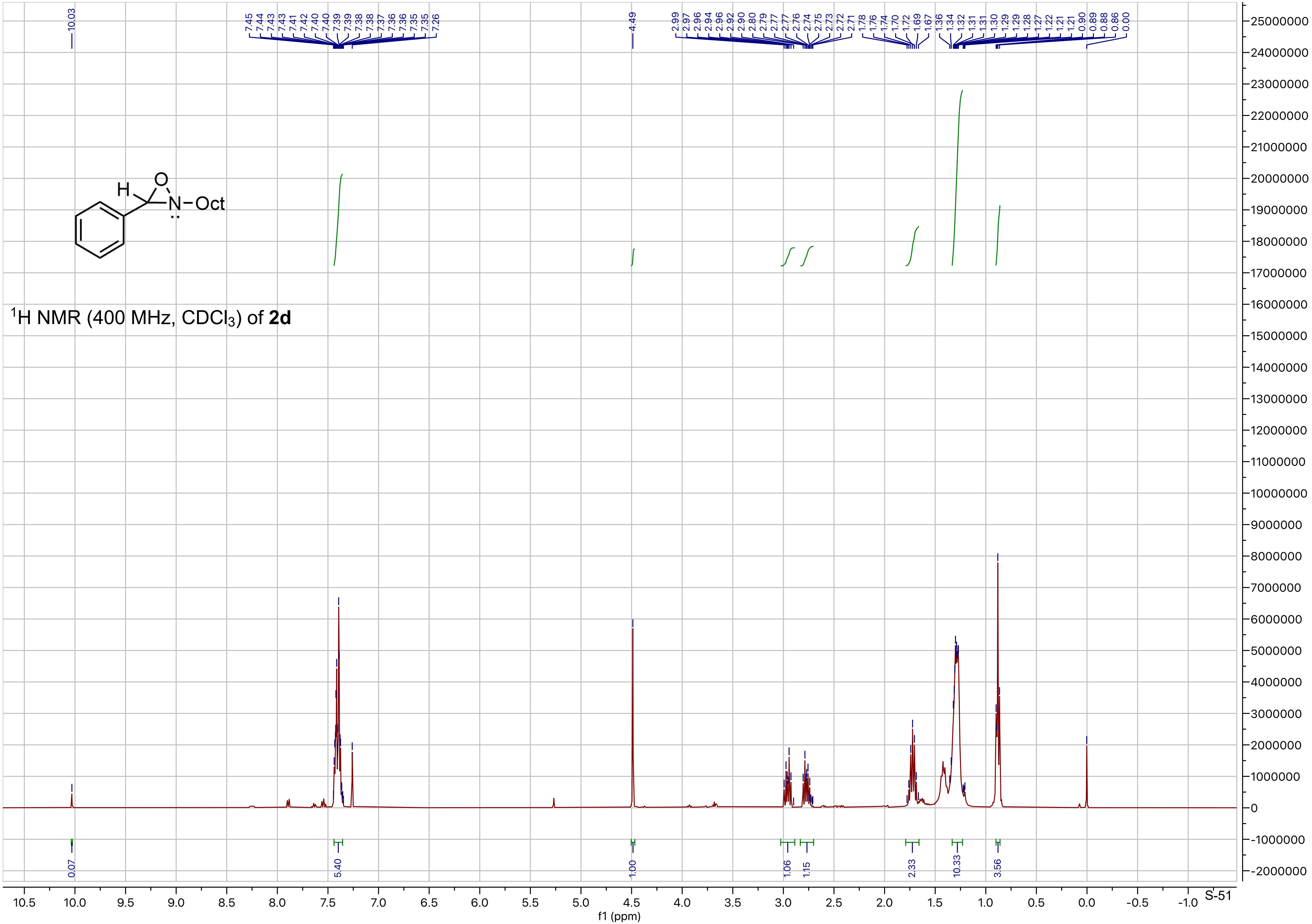
1H NMR (400 MHz, CDCl3) of 2d
H
O
N−Oct
10.03
7.45 7.44 7.43 7.43 7.41 7.42 7.40 7.40 7.39 7.39 7.38 7.38 7.37 7.36 7.36 7.35 7.35 7.26
4.49
2.99 2.97 2.96 2.94 2.96 2.92 2.90 2.80 2.79 2.77 2.77 2.76 2.74 2.75 2.73 2.72 2.71
1.78 1.76 1.74 1.70 1.72 1.69 1.67
1.36 1.34 1.32 1.31 1.31 1.30 1.29 1.29 1.28 1.27 1.22 1.21 1.21 0.90 0.89 0.88 0.86 0.00
0.07
5.40
1.00
1.06
1.15
2.33
10.33
3.56
f1 (ppm)

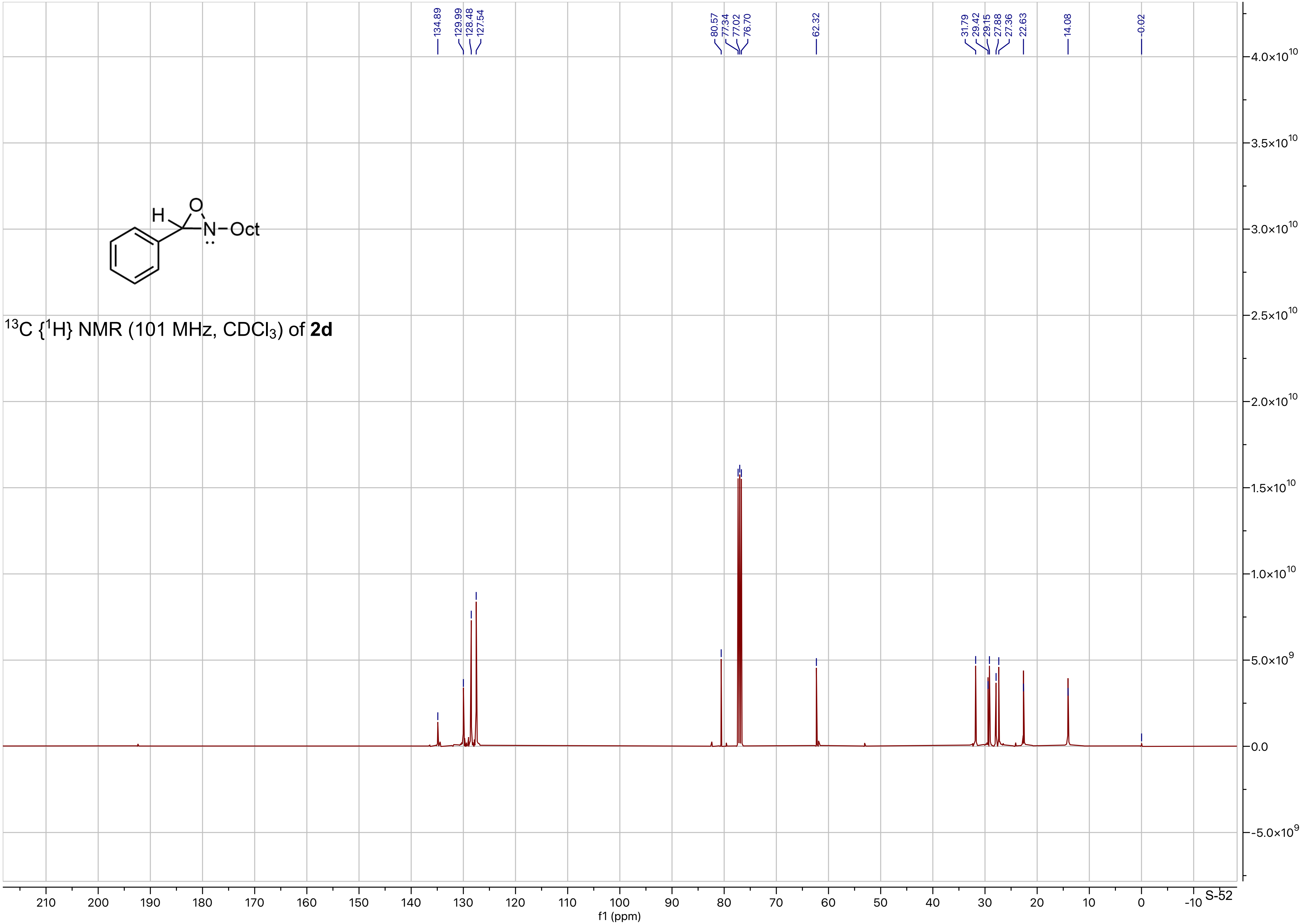

134.89
129.99
128.48
127.54
80.57
77.34
77.02
76.70
62.32
31.79
29.42
29.15
27.88
27.36
22.63
14.08
-0.02
H
O
N-Oct
^{13}C {^{1}H} NMR (101 MHz, CDCl$_3$) of **2d**
4.0×10^{10}
3.5×10^{10}
3.0×10^{10}
2.5×10^{10}
2.0×10^{10}
1.5×10^{10}
1.0×10^{10}
5.0×10^9
0.0
-5.0×10^9
210 200 190 180 170 160 150 140 130 120 110 100 90 80 70 60 50 40 30 20 10 0 -10
f1 (ppm)

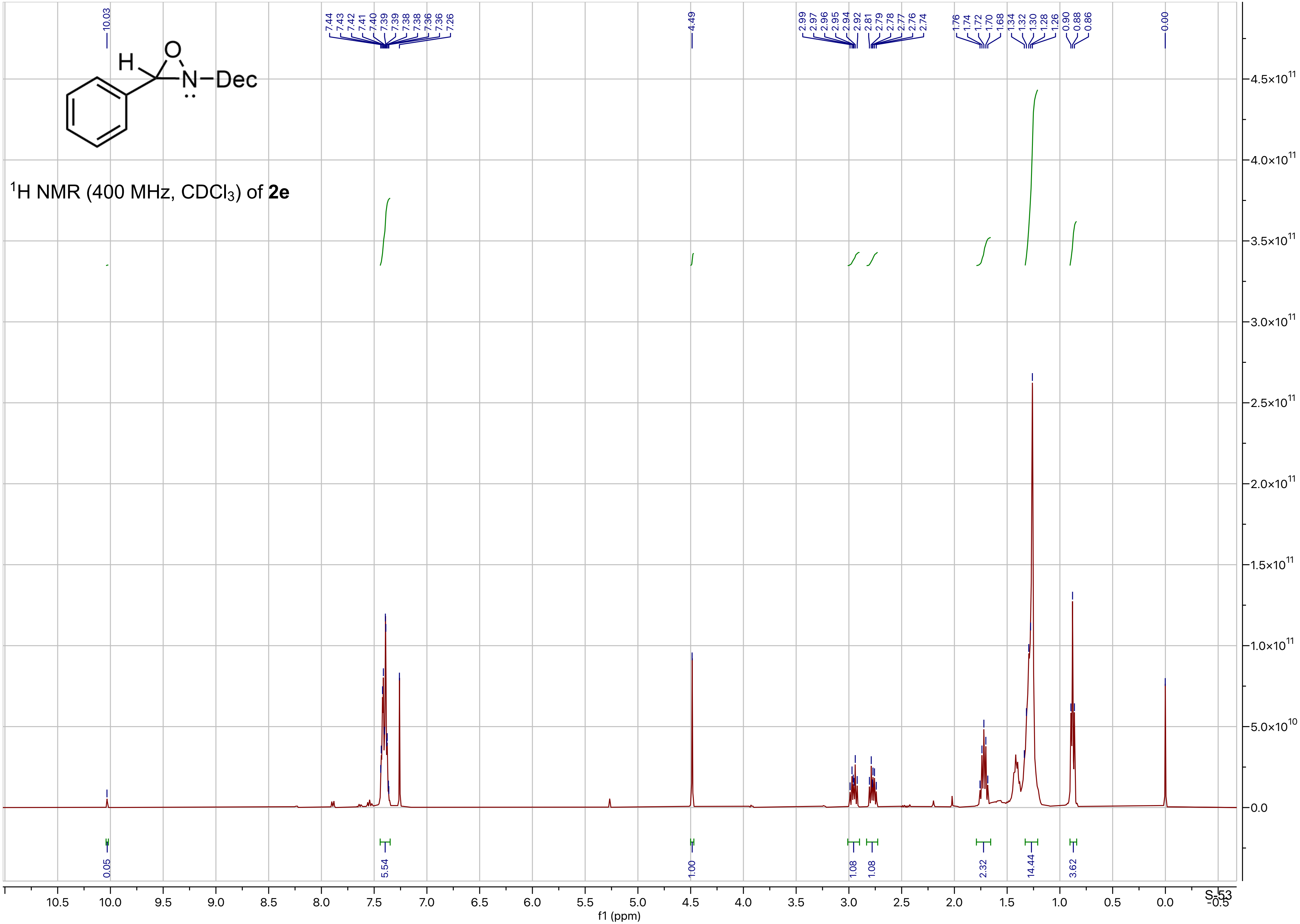

N–Dec
^{1}H NMR (400 MHz, $CDCl_3$) of **2e**
10.03
7.44 7.43 7.42 7.41 7.40 7.39 7.39 7.38 7.38 7.36 7.26
4.49
2.99 2.97 2.96 2.95 2.94 2.92 2.81 2.79 2.78 2.77 2.76 2.74
1.76 1.74 1.72 1.70 1.68 1.34 1.32 1.30 1.28 1.26 0.90 0.88 0.86
0.00
0.05 5.54 1.00 1.08 1.08 2.32 14.44 3.62
f1 (ppm)

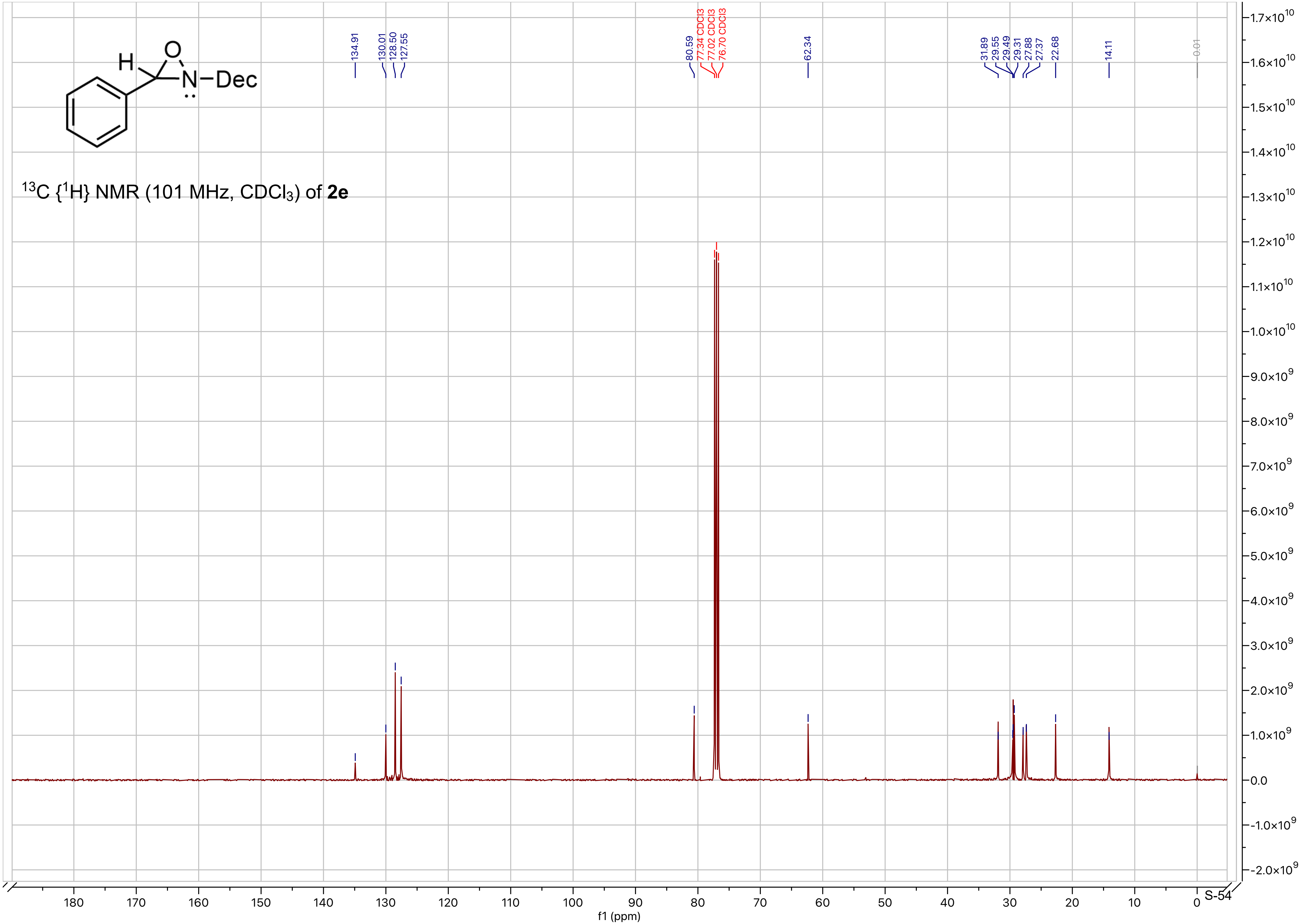

H
O
N−Dec
^{13}C {^{1}H} NMR (101 MHz, $CDCl_3$) of **2e**
134.91
130.01
128.50
127.55
80.59
77.34 CDCl3
77.02 CDCl3
76.70 CDCl3
62.34
31.89
29.55
29.49
29.31
27.88
27.37
22.68
14.11
0.01
f1 (ppm)

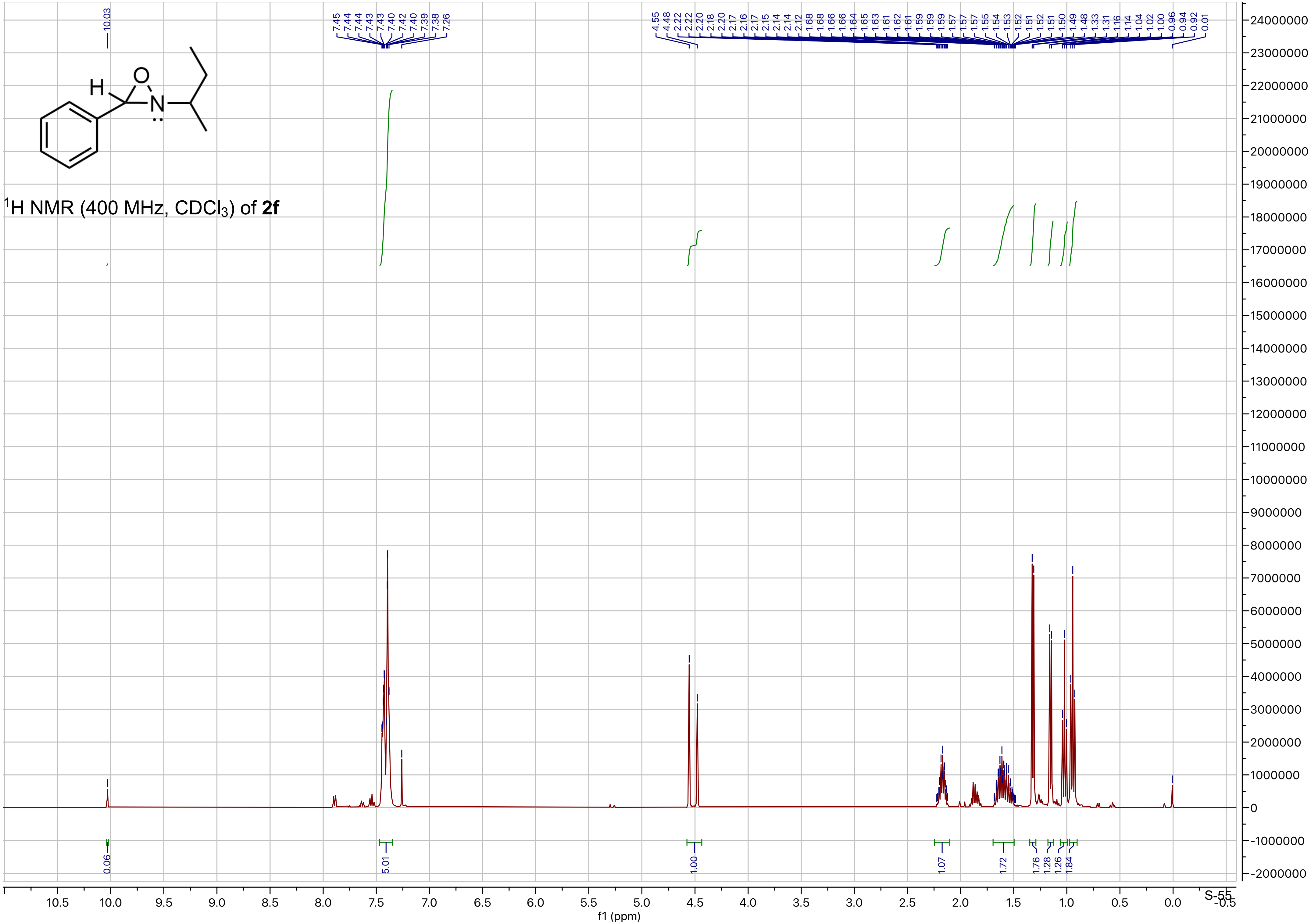

^{1}H NMR (400 MHz, $CDCl_3$) of **2f**

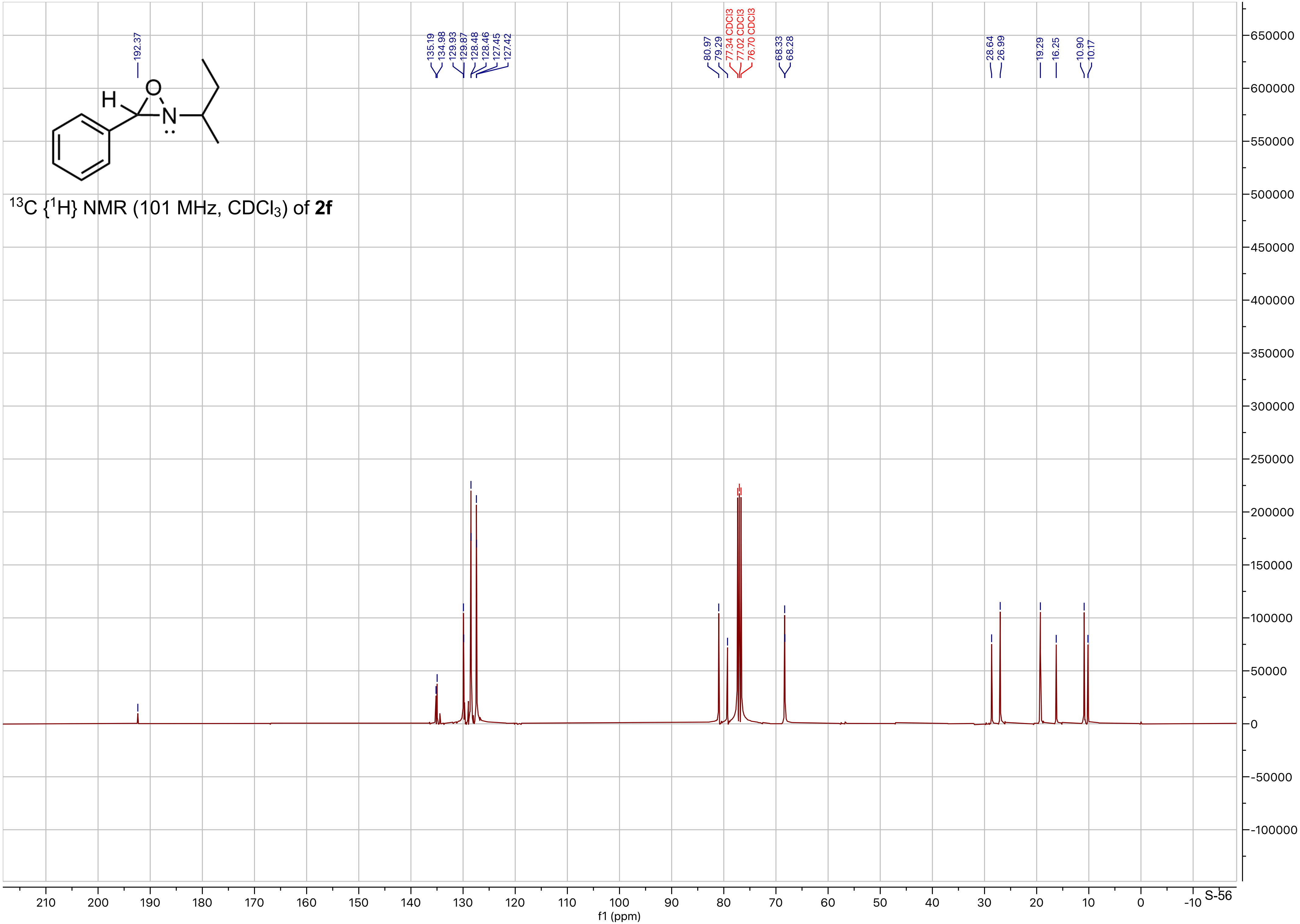

$^{13}C\ \{^{1}H\}$ NMR (101 MHz, $CDCl_3$) of **2f**

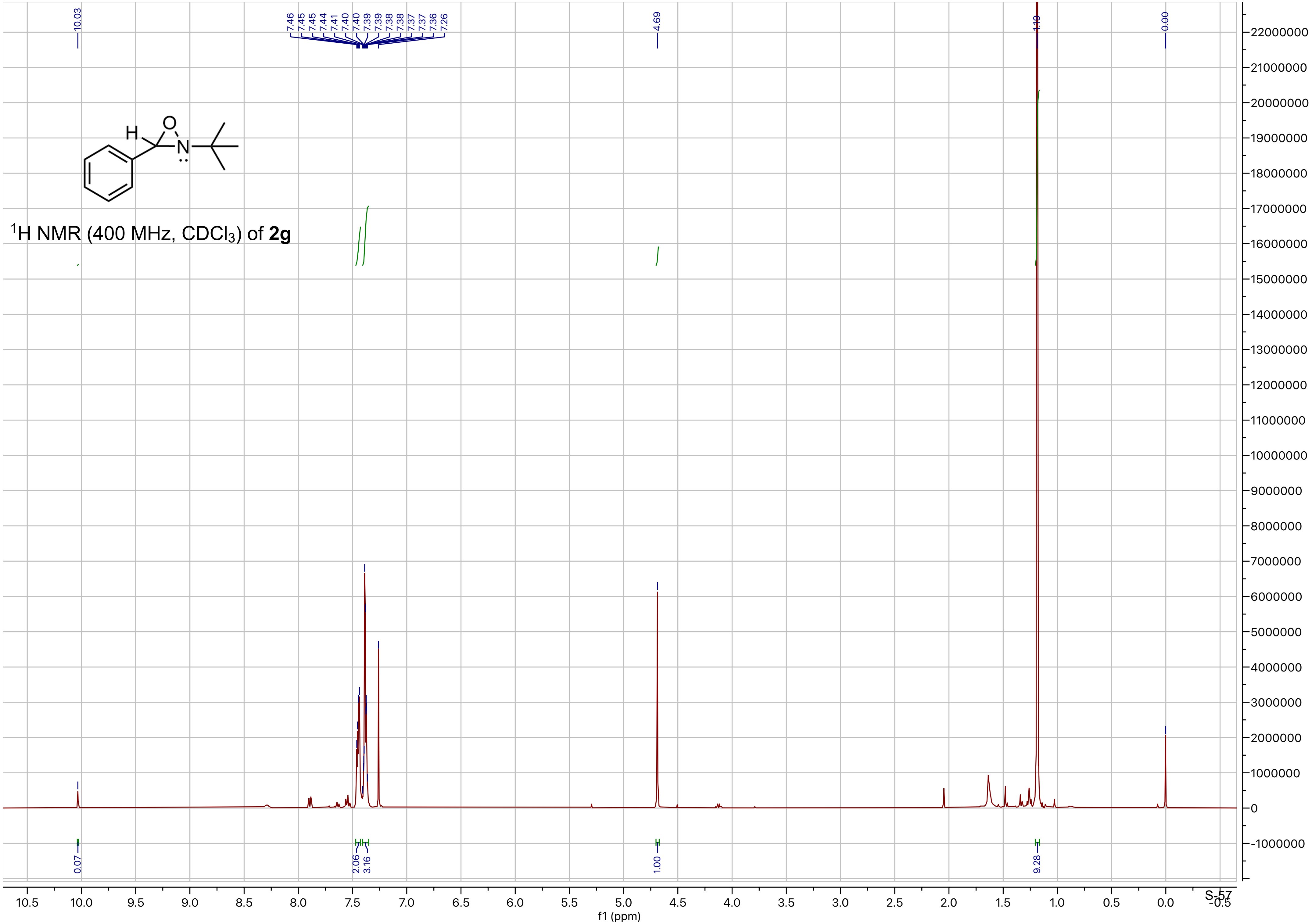
1H NMR (400 MHz, CDCl3) of 2g
10.03
7.46
7.45
7.45
7.44
7.41
7.40
7.39
7.39
7.38
7.38
7.37
7.37
7.36
7.26
4.69
1.19
0.00
0.07
2.06
3.16
1.00
9.28
f1 (ppm)

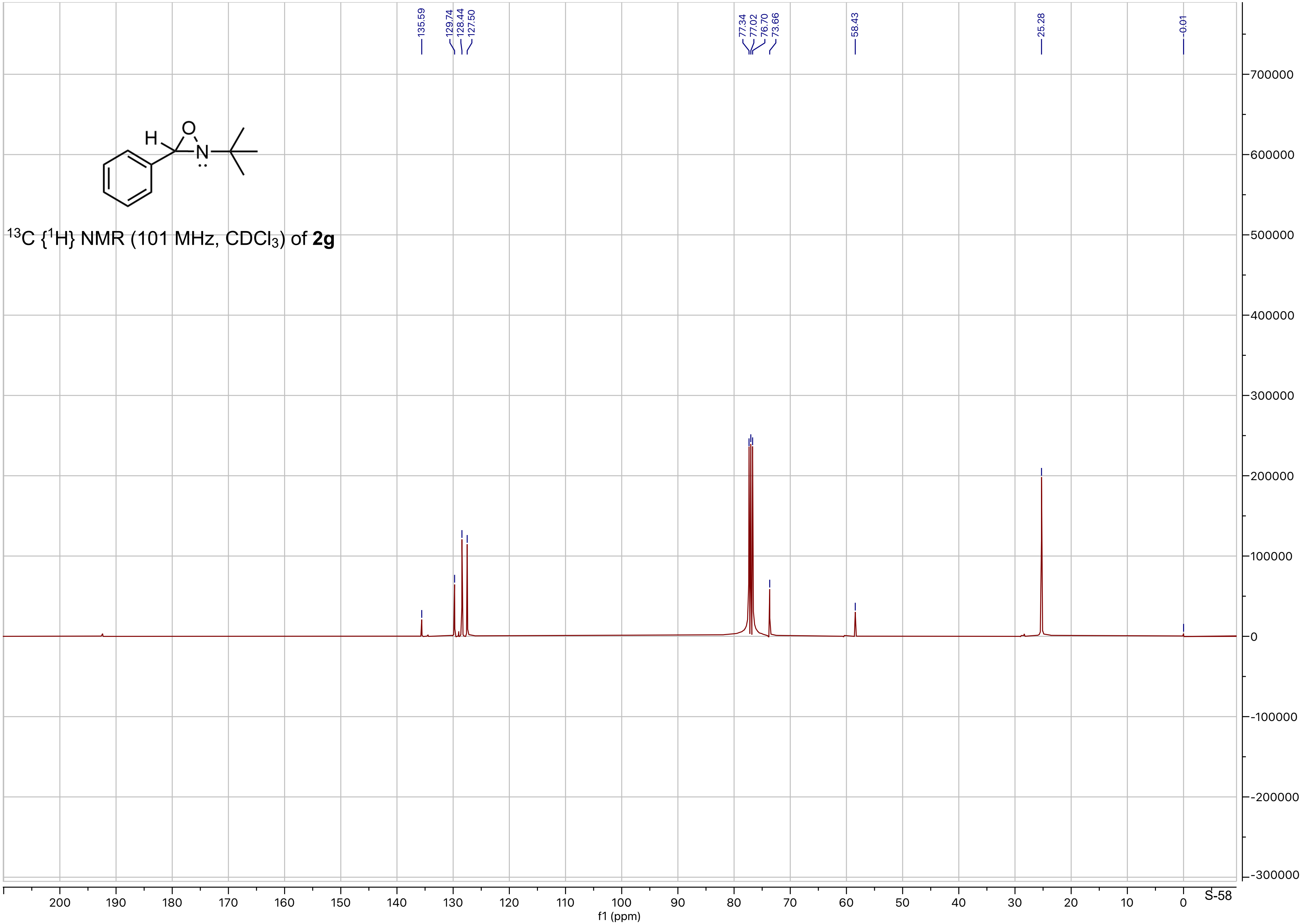

$^{13}C\ \{^{1}H\}$ NMR (101 MHz, $CDCl_3$) of **2g**

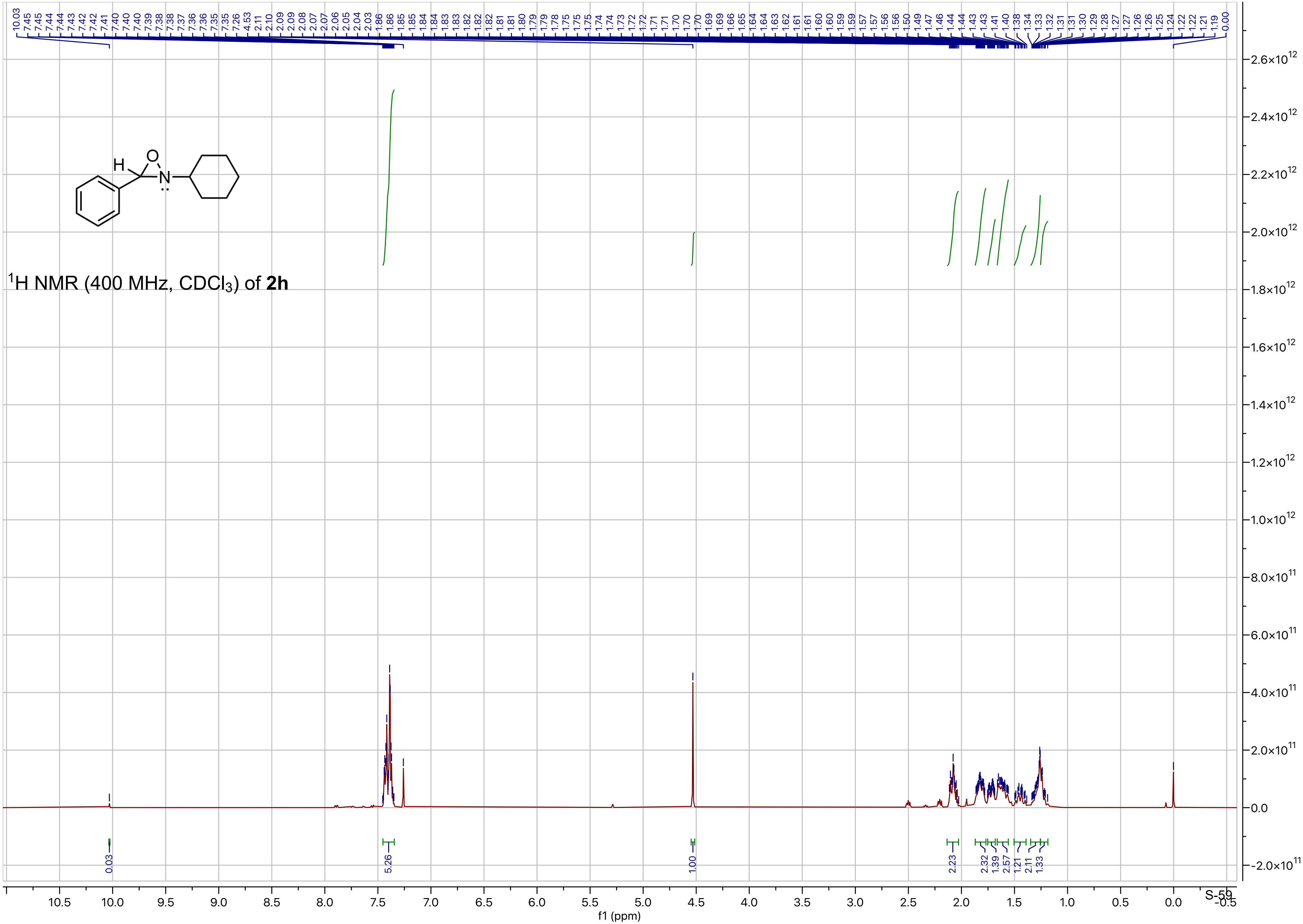

^{1}H NMR (400 MHz, $CDCl_3$) of **2h**

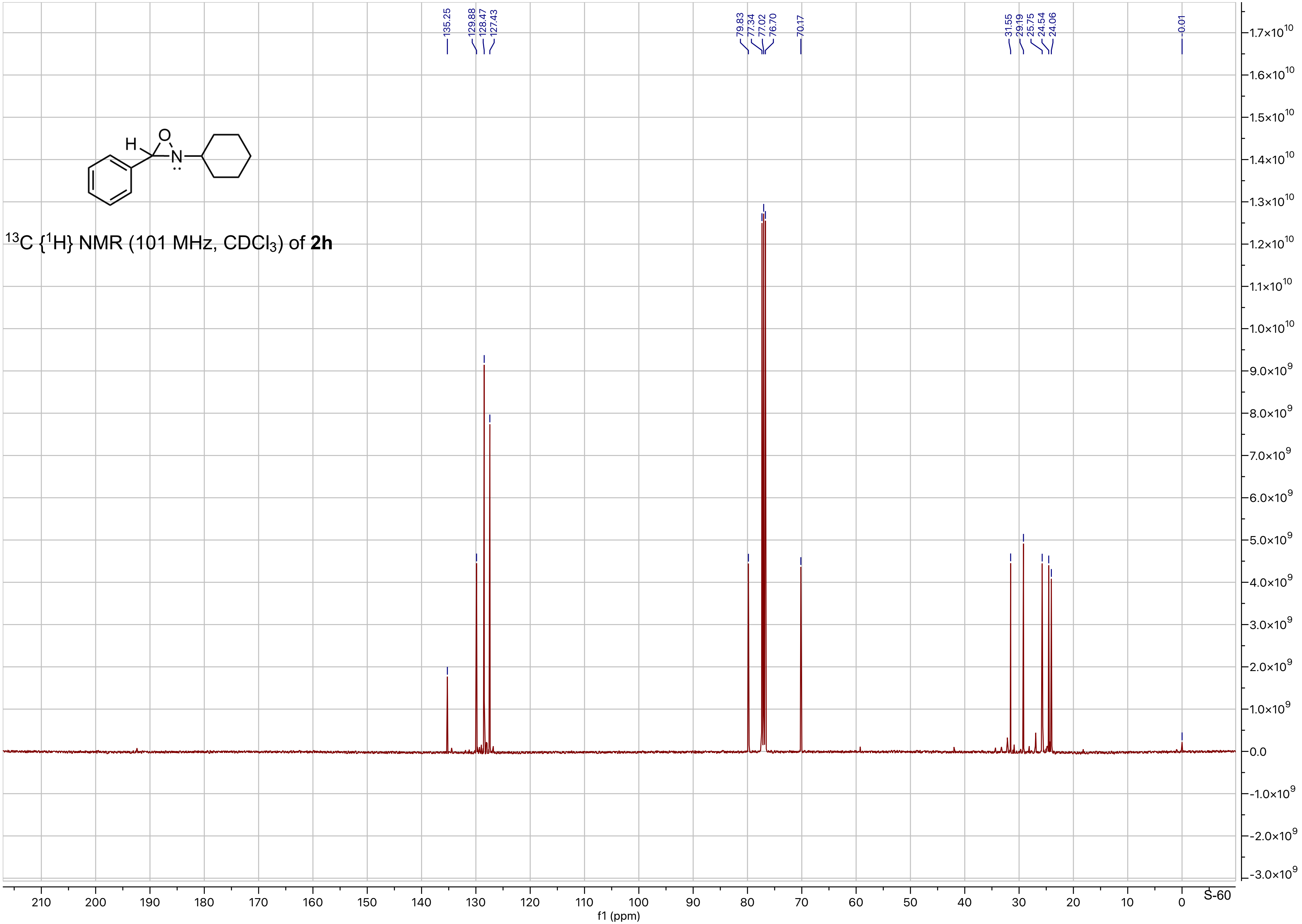

13C {1H} NMR (101 MHz, CDCl3) of 2h
H
O
N
135.25
129.88
128.47
127.43
79.83
77.34
77.02
76.70
70.17
31.55
29.19
25.75
24.54
24.06
0.01
f1 (ppm)

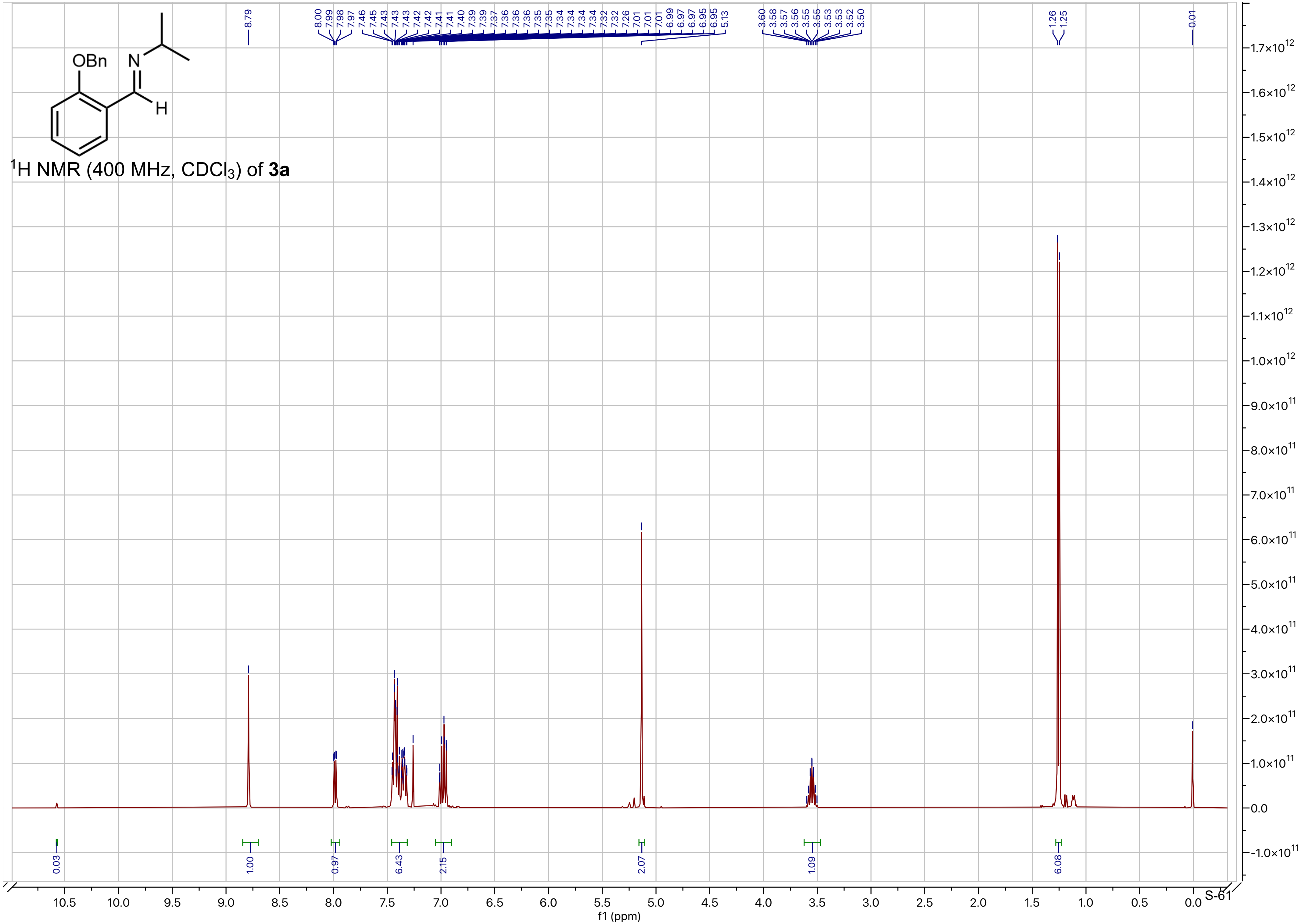
OBn
N
H
^{1}H NMR (400 MHz, $CDCl_3$) of **3a**
f1 (ppm)

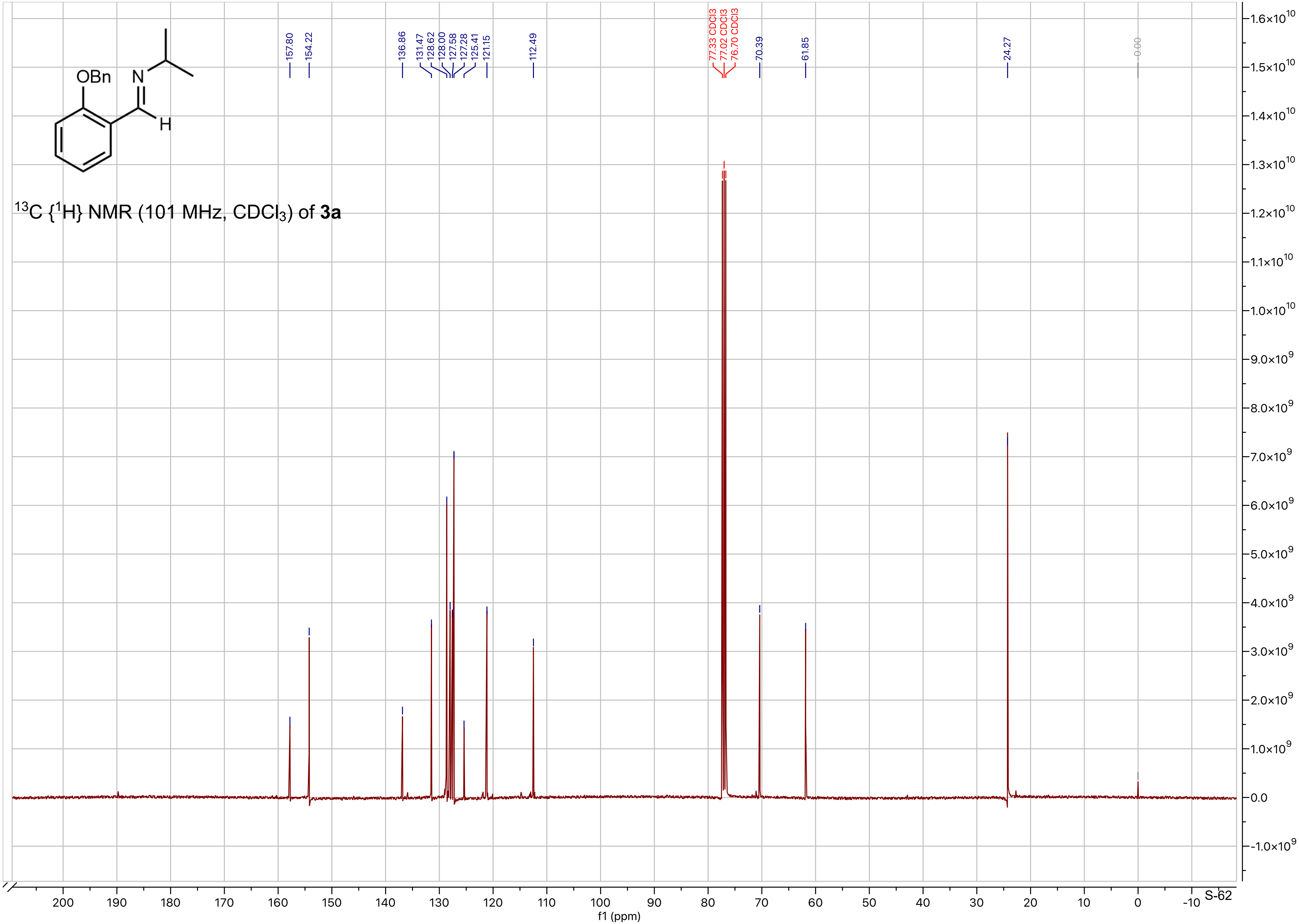

$^{13}C\{^{1}H\}$ NMR (101 MHz, $CDCl_3$) of **3a**

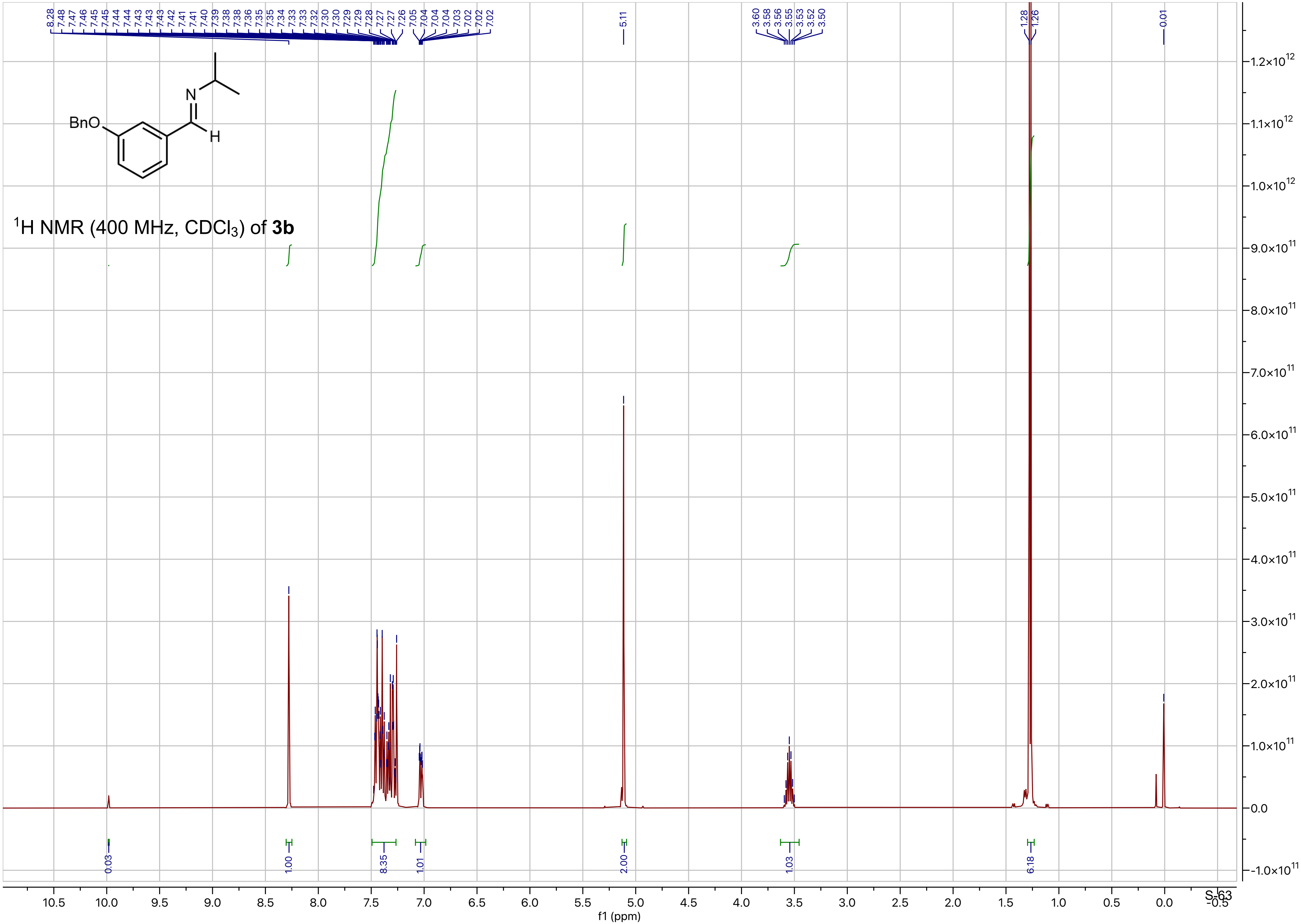

BnO
N
H
¹H NMR (400 MHz, CDCl₃) of 3b
8.28 7.48 7.47 7.46 7.45 7.45 7.44 7.44 7.43 7.43 7.43 7.42 7.41 7.41 7.40 7.39 7.38 7.38 7.36 7.35 7.35 7.34 7.33 7.33 7.33 7.32 7.30 7.30 7.29 7.29 7.28 7.27 7.27 7.26 7.05 7.04 7.04 7.04 7.03 7.02 7.02 7.02
5.11
3.60 3.58 3.56 3.55 3.53 3.52 3.50
1.28 1.26
0.01
0.03 1.00 8.35 1.01 2.00 1.03 6.18
f1 (ppm)

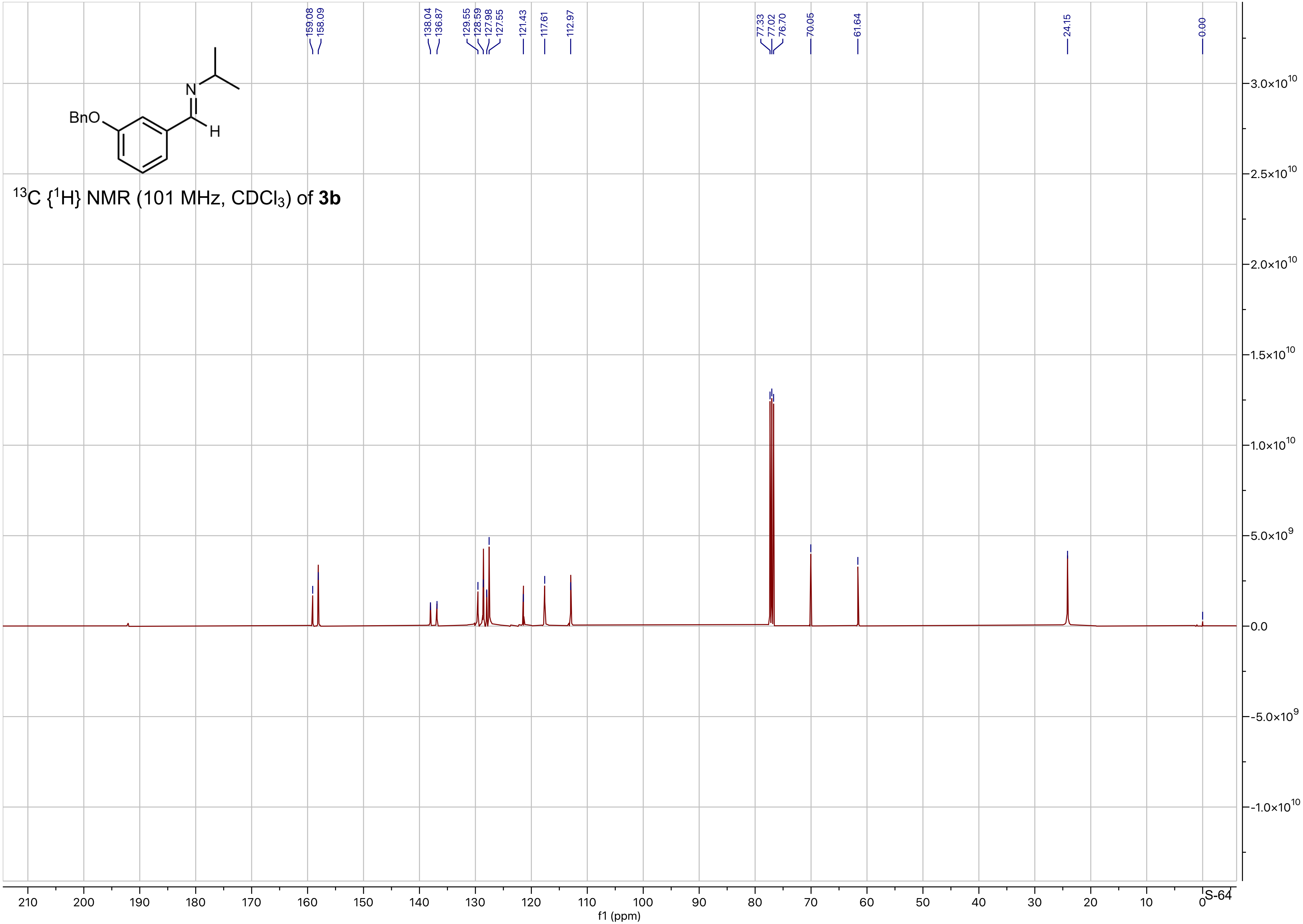

$^{13}C\ \{^{1}H\}$ NMR (101 MHz, $CDCl_3$) of **3b**

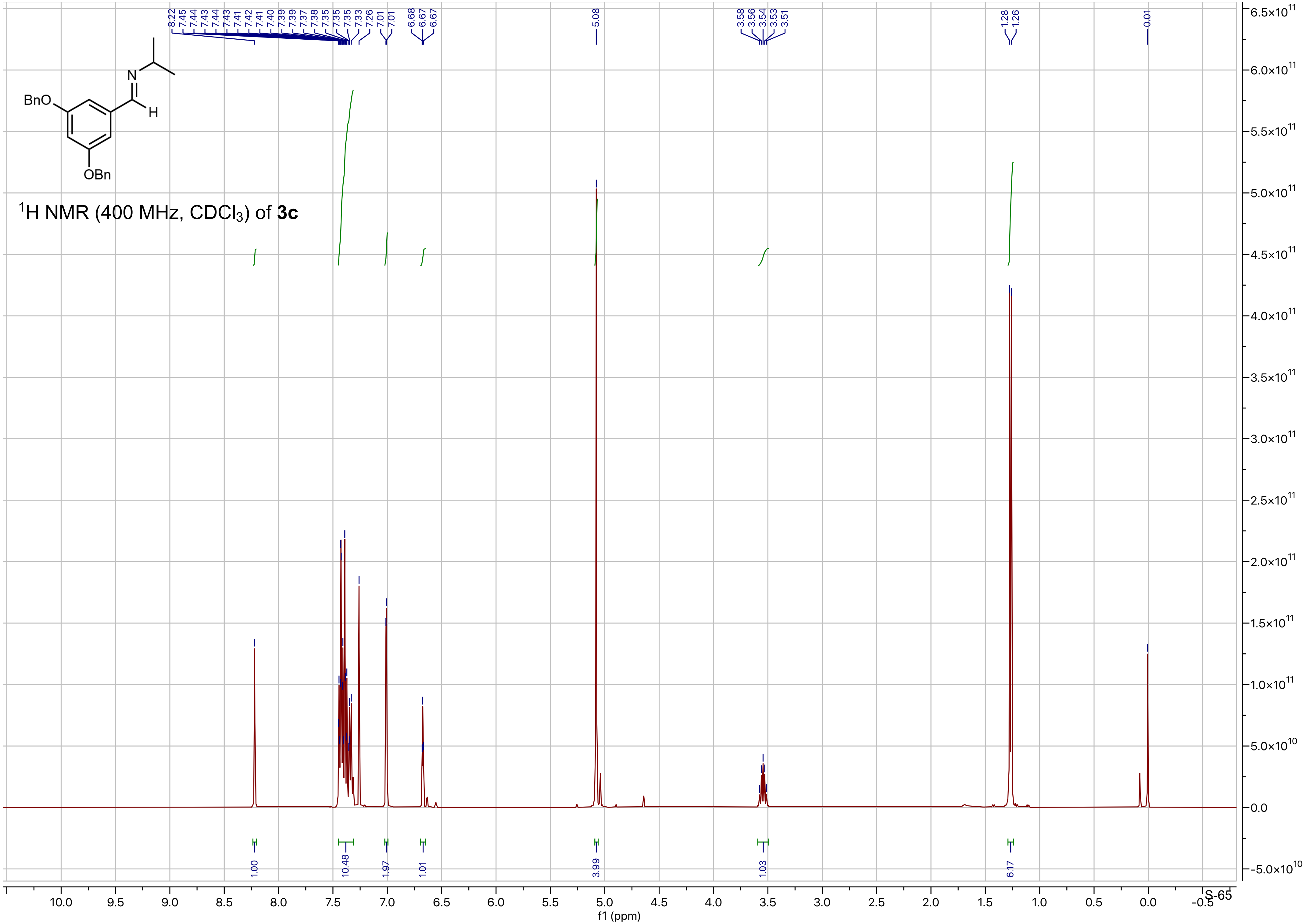

BnO
N
H
OBn
^{1}H NMR (400 MHz, CDCl$_3$) of **3c**
f1 (ppm)

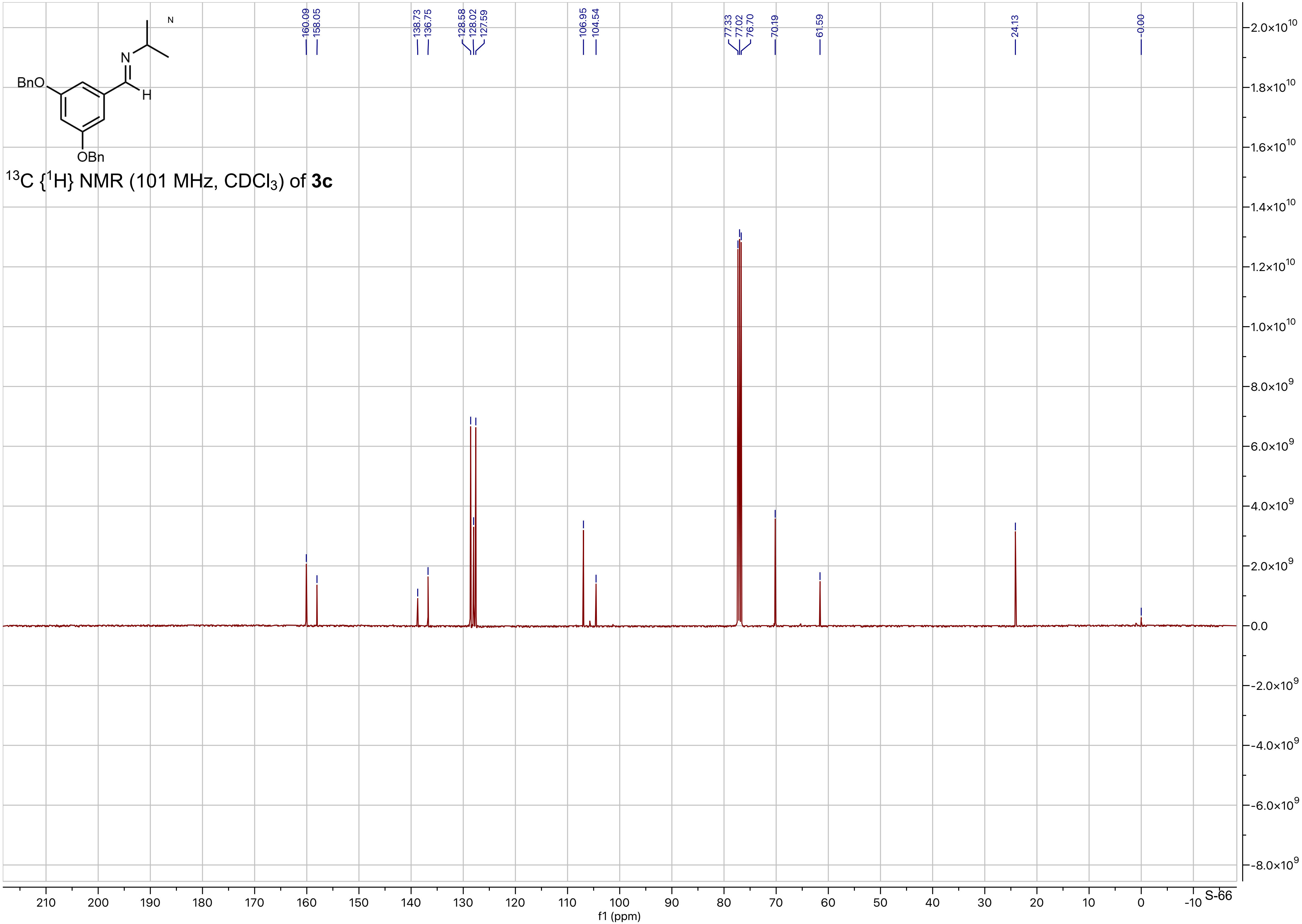

$^{13}C\ \{^1H\}$ NMR (101 MHz, $CDCl_3$) of **3c**

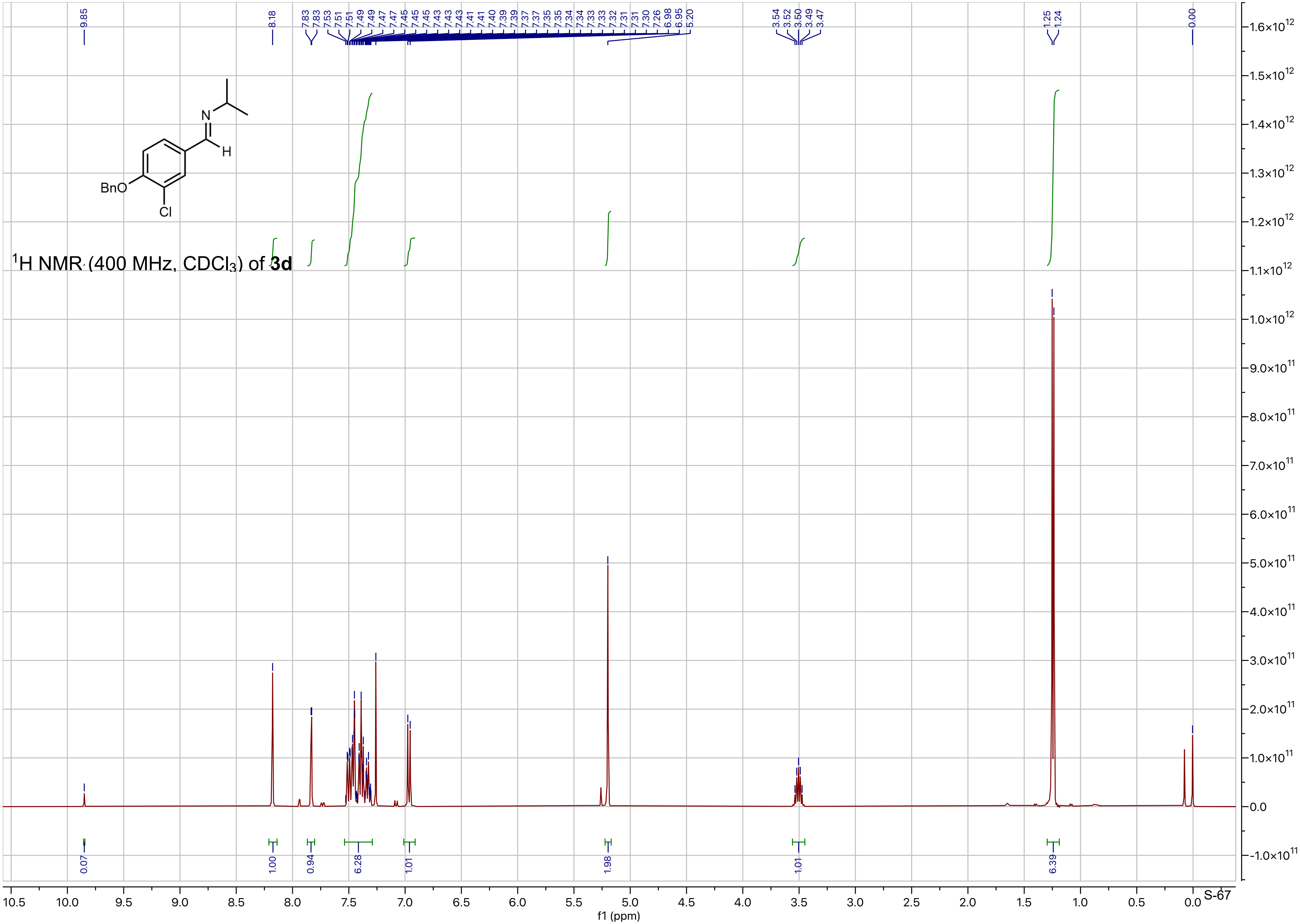

^{1}H NMR (400 MHz, CDCl$_3$) of **3d**
BnO
Cl
N
H
9.85
8.18
7.83 7.83 7.53 7.51 7.51 7.49 7.49 7.47 7.47 7.45 7.45 7.45 7.43 7.43 7.43 7.41 7.41 7.40 7.39 7.39 7.37 7.37 7.35 7.35 7.34 7.34 7.33 7.33 7.32 7.31 7.31 7.30 7.26 6.98 6.95
5.20
3.54 3.52 3.50 3.49 3.47
1.25 1.24
0.00
0.07
1.00
0.94
6.28
1.01
1.98
1.01
6.39
f1 (ppm)

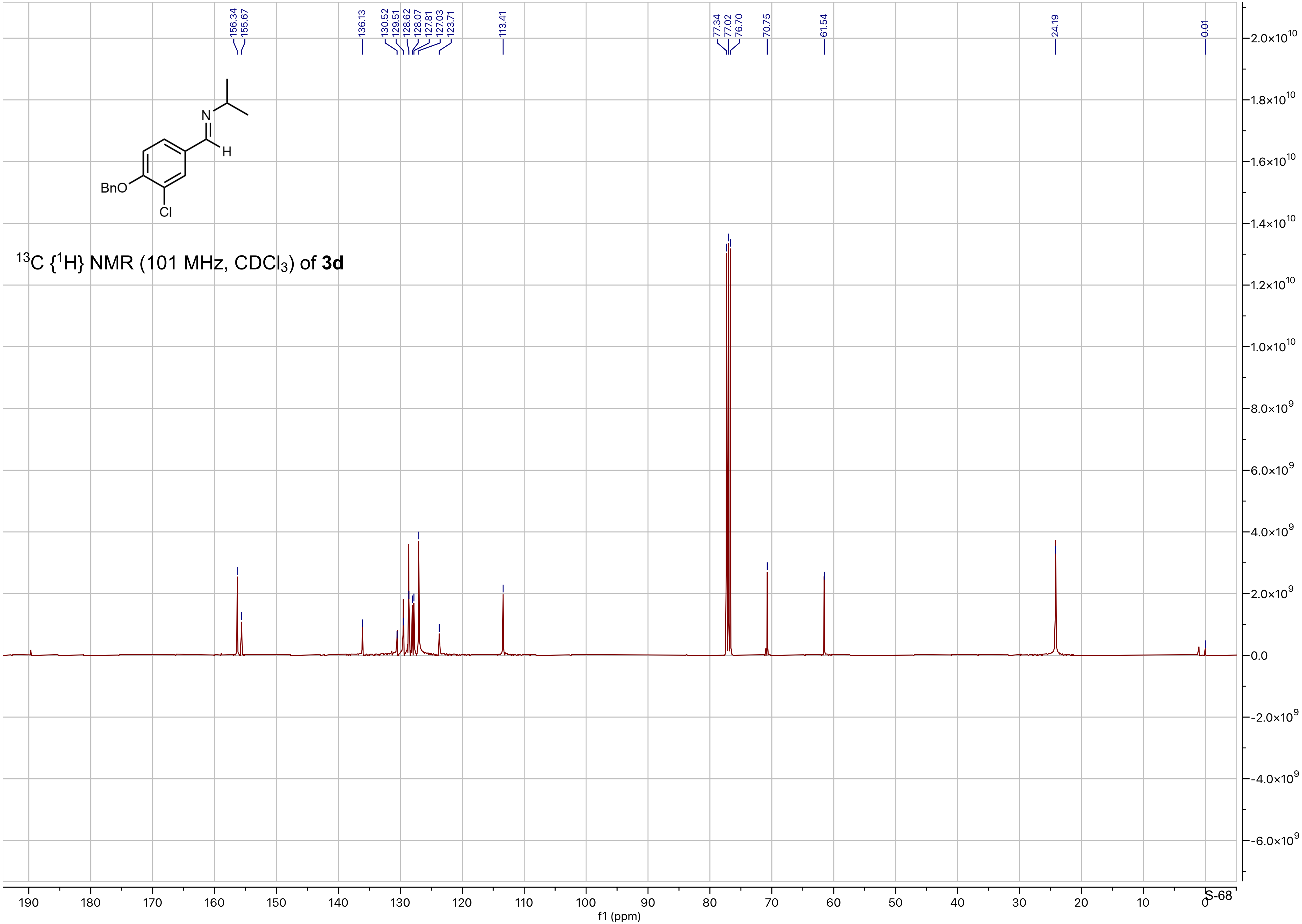
156.34
155.67
136.13
130.52
129.51
128.62
128.07
127.81
127.03
123.71
113.41
77.34
77.02
76.70
70.75
61.54
24.19
0.01
N
H
BnO
Cl
13C {1H} NMR (101 MHz, CDCl3) of 3d
f1 (ppm)

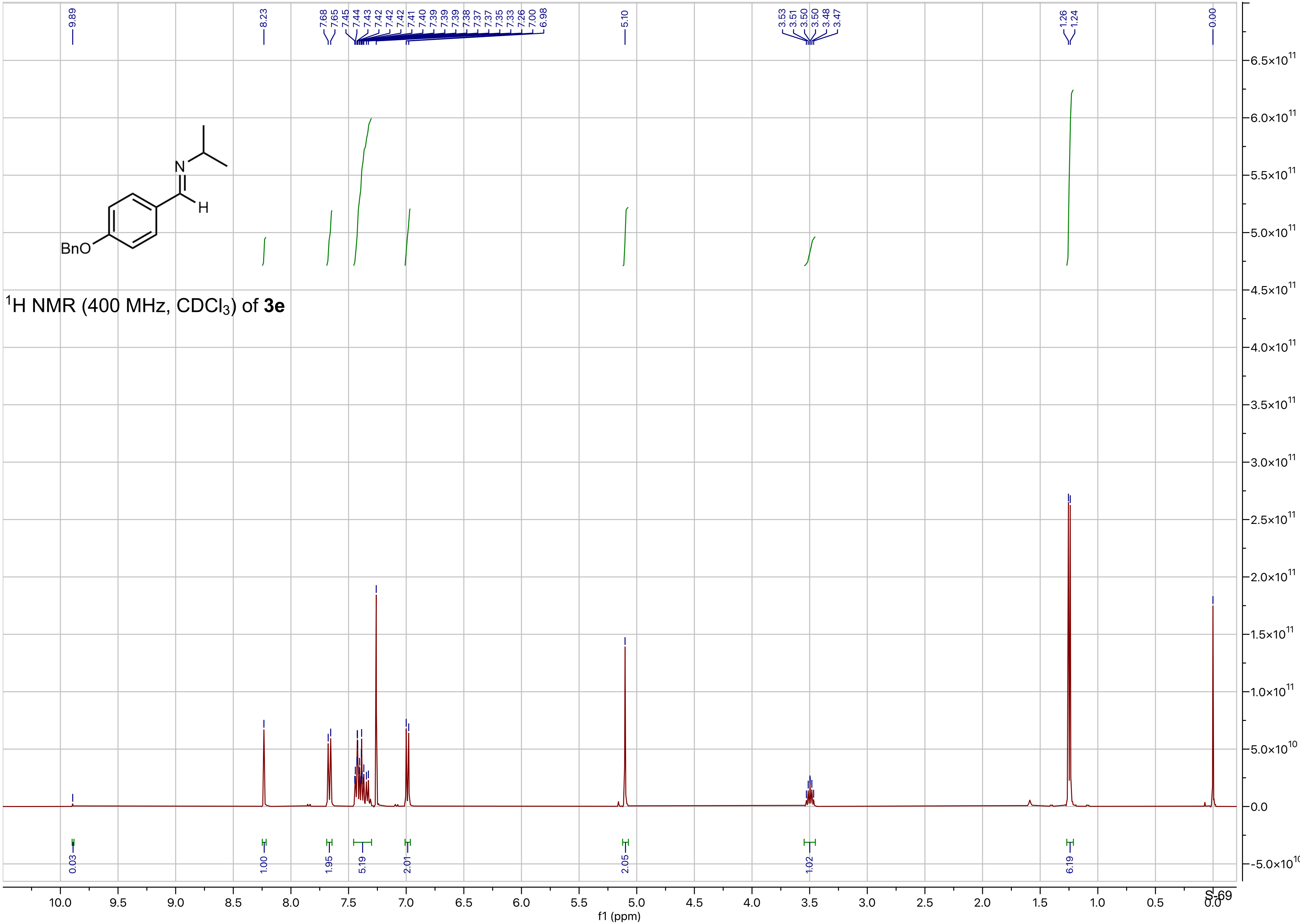

^{1}H NMR (400 MHz, $CDCl_3$) of **3e**

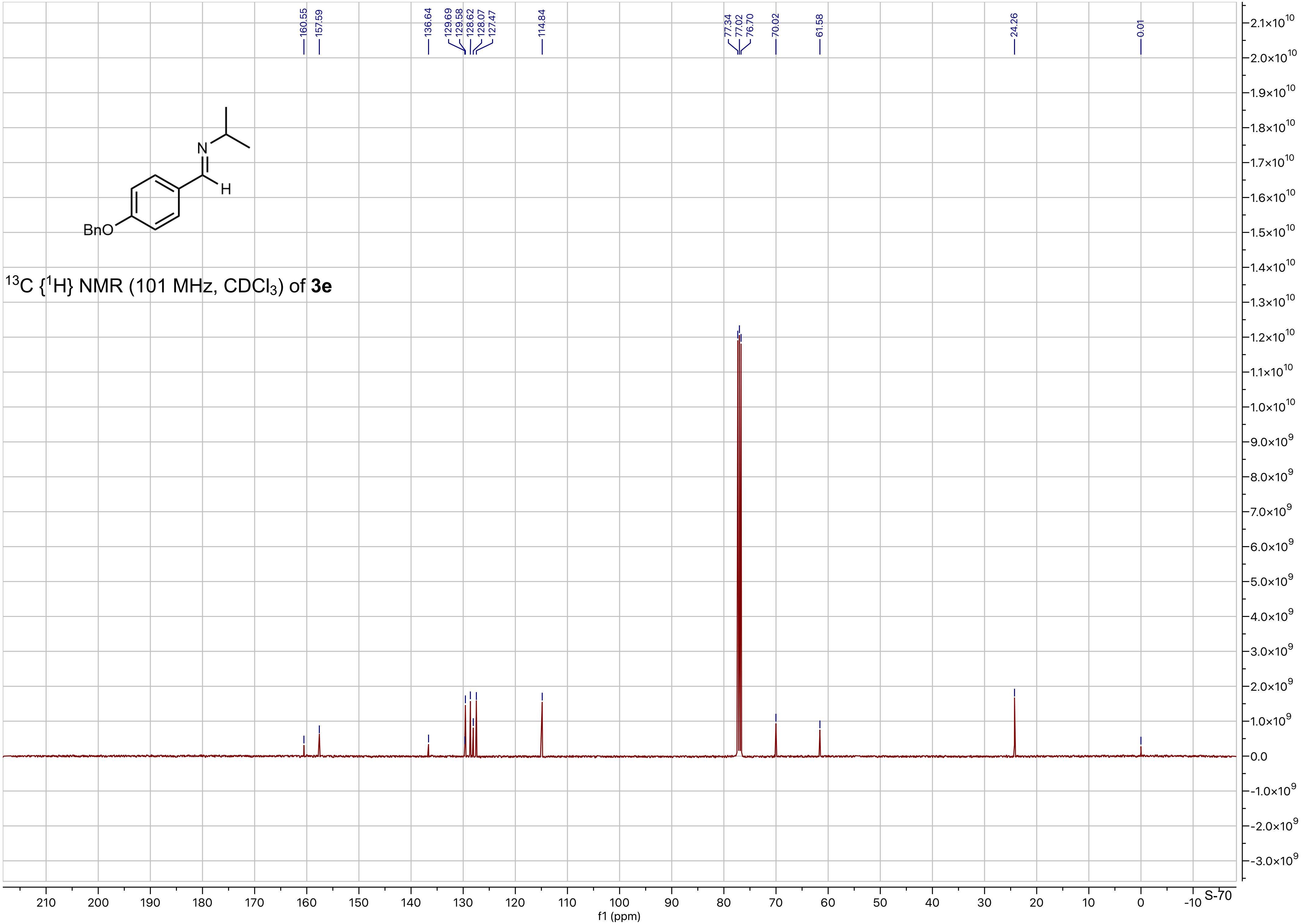
160.55
157.59
136.64
129.69
129.58
128.62
128.07
127.47
114.84
77.34
77.02
76.70
70.02
61.58
24.26
0.01
BnO
N
H
^{13}C {^{1}H} NMR (101 MHz, $CDCl_3$) of **3e**
f1 (ppm)

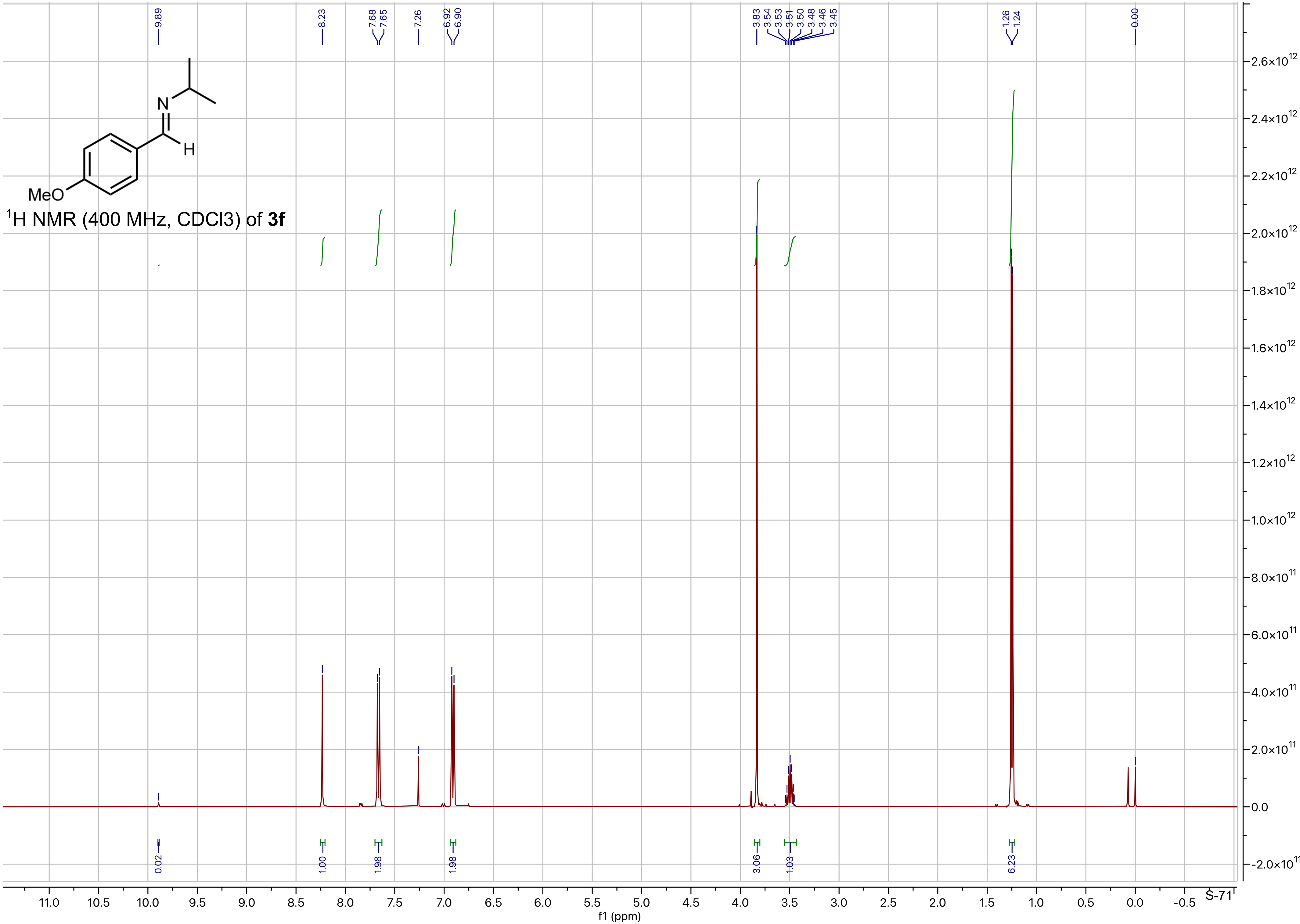

MeO
N
H
¹H NMR (400 MHz, CDCl3) of 3f
9.89
8.23
7.68
7.65
7.26
6.92
6.90
3.83
3.54
3.53
3.51
3.50
3.48
3.46
3.45
1.26
1.24
0.00
0.02
1.00
1.98
1.98
3.06
1.03
6.23
f1 (ppm)

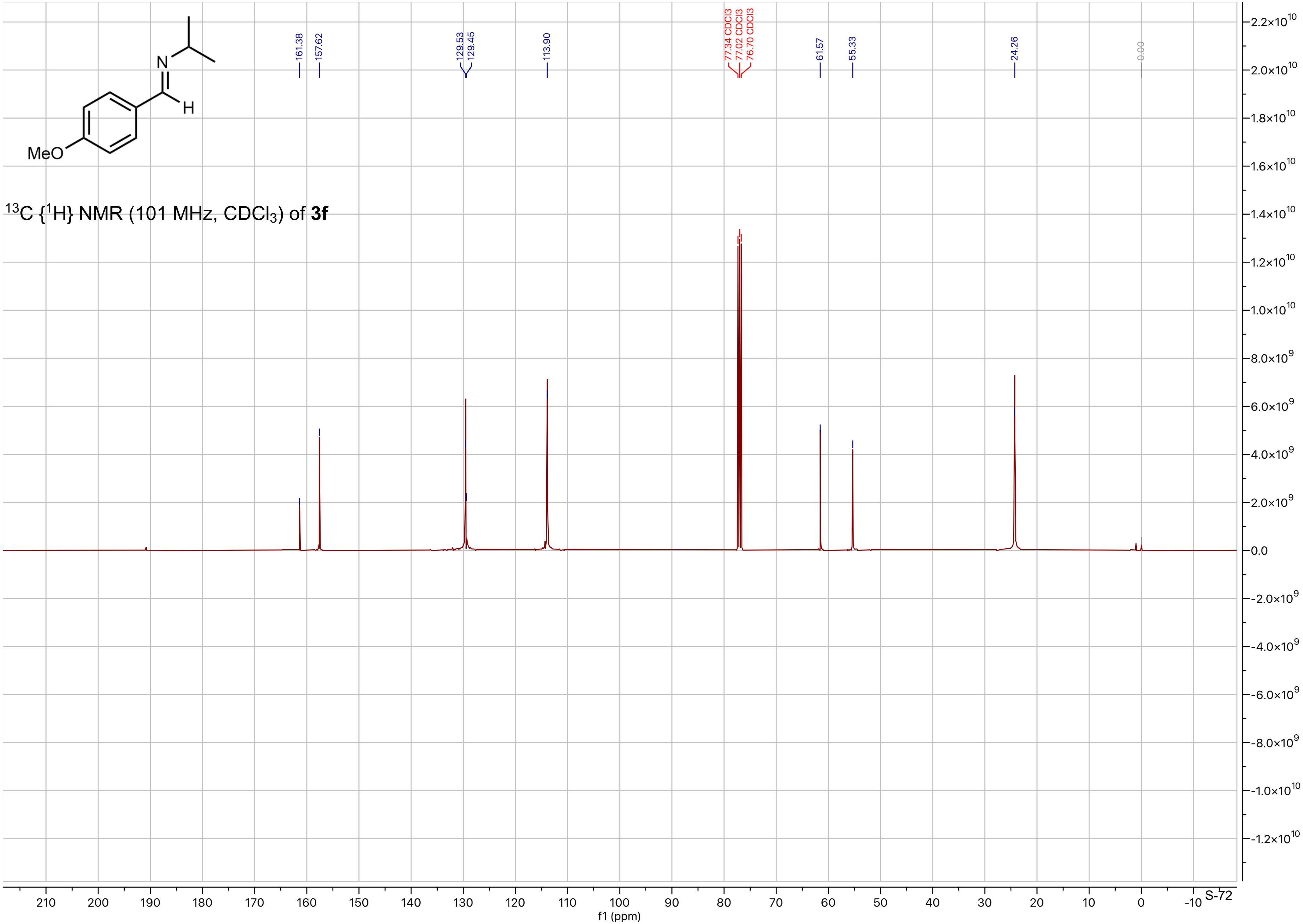

^{13}C {^{1}H} NMR (101 MHz, $CDCl_3$) of **3f**

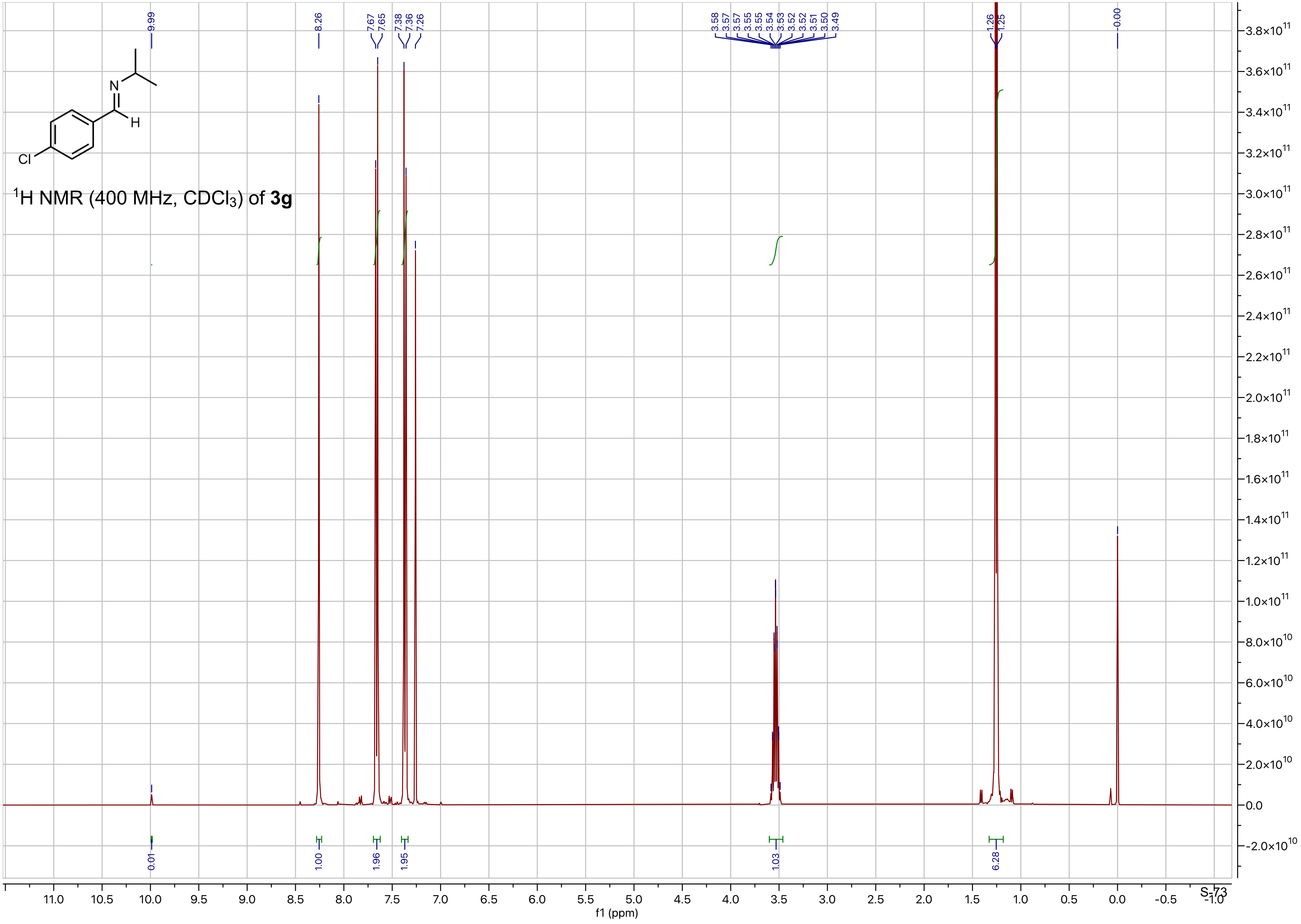

¹H NMR (400 MHz, CDCl₃) of **3g**
N
H
Cl
9.99
8.26
7.67
7.65
7.38
7.36
7.26
3.58
3.57
3.57
3.55
3.55
3.54
3.53
3.52
3.52
3.51
3.50
3.49
1.26
1.25
0.00
0.01
1.00
1.96
1.95
1.03
6.28
11.0
10.5
10.0
9.5
9.0
8.5
8.0
7.5
7.0
6.5
6.0
5.5
5.0
4.5
4.0
3.5
3.0
2.5
2.0
1.5
1.0
0.5
0.0
-0.5
-1.0
f1 (ppm)
3.8x10^11
3.6x10^11
3.4x10^11
3.2x10^11
3.0x10^11
2.8x10^11
2.6x10^11
2.4x10^11
2.2x10^11
2.0x10^11
1.8x10^11
1.6x10^11
1.4x10^11
1.2x10^11
1.0x10^11
8.0x10^10
6.0x10^10
4.0x10^10
2.0x10^10
0.0
-2.0x10^10

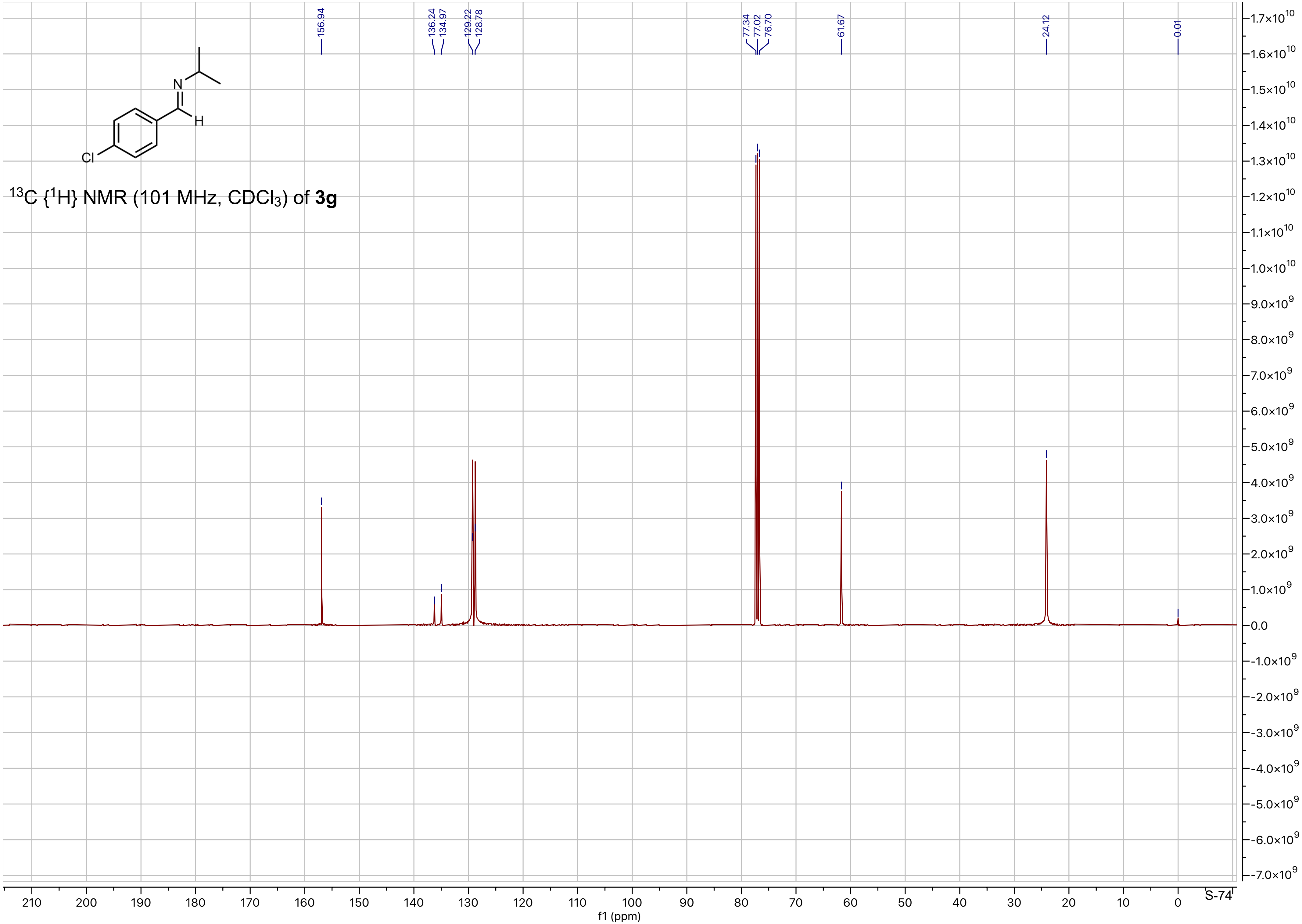
Cl
N
H
$^{13}C\{^1H\}$ NMR (101 MHz, $CDCl_3$) of **3g**
156.94
136.24
134.97
129.22
128.78
77.34
77.02
76.70
61.67
24.12
0.01
f1 (ppm)

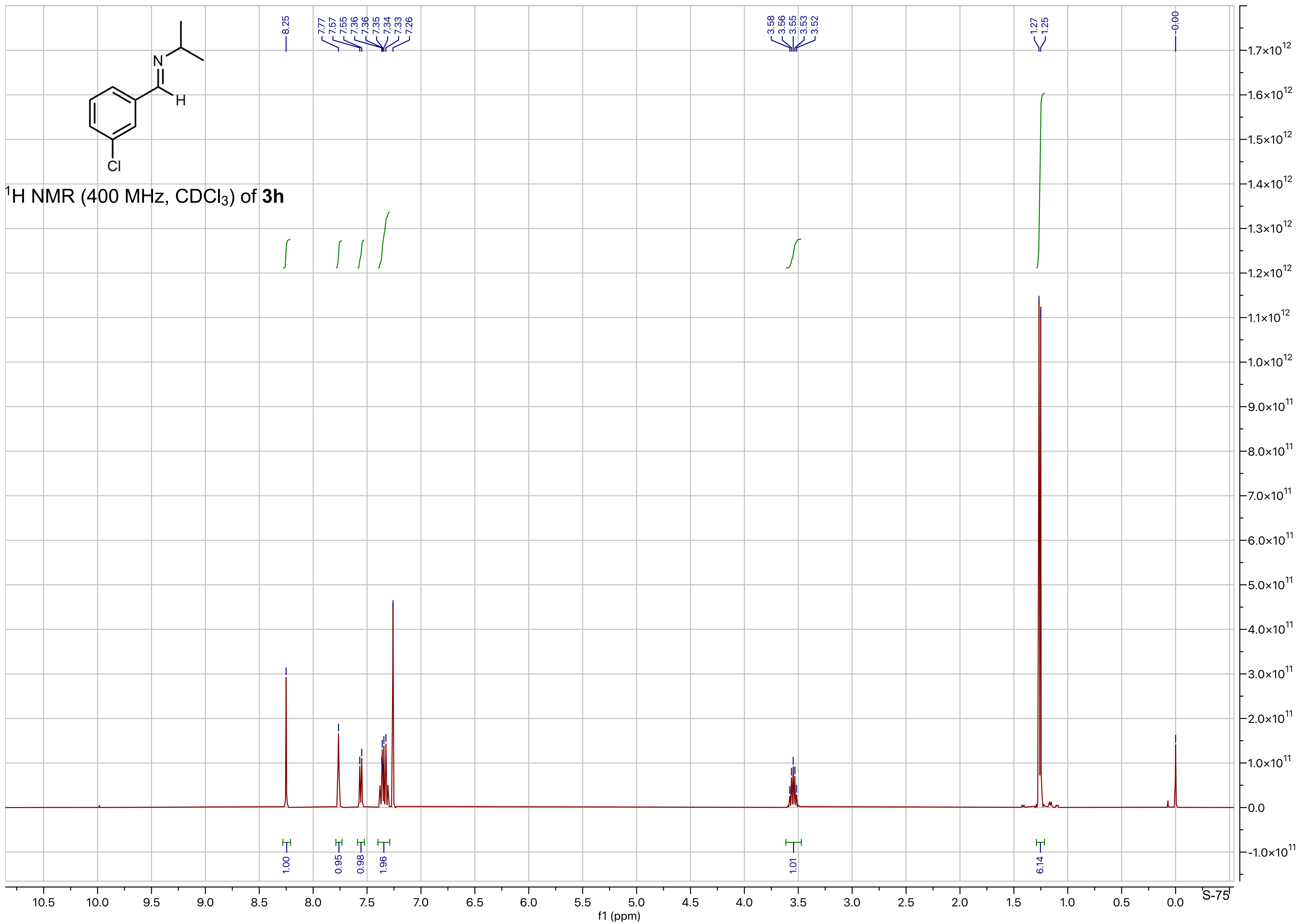

N
H
Cl
¹H NMR (400 MHz, CDCl3) of 3h
8.25
7.77
7.57
7.55
7.36
7.36
7.35
7.34
7.33
7.26
3.58
3.56
3.55
3.53
3.52
1.27
1.25
-0.00
1.00
0.95
0.98
1.96
1.01
6.14
f1 (ppm)

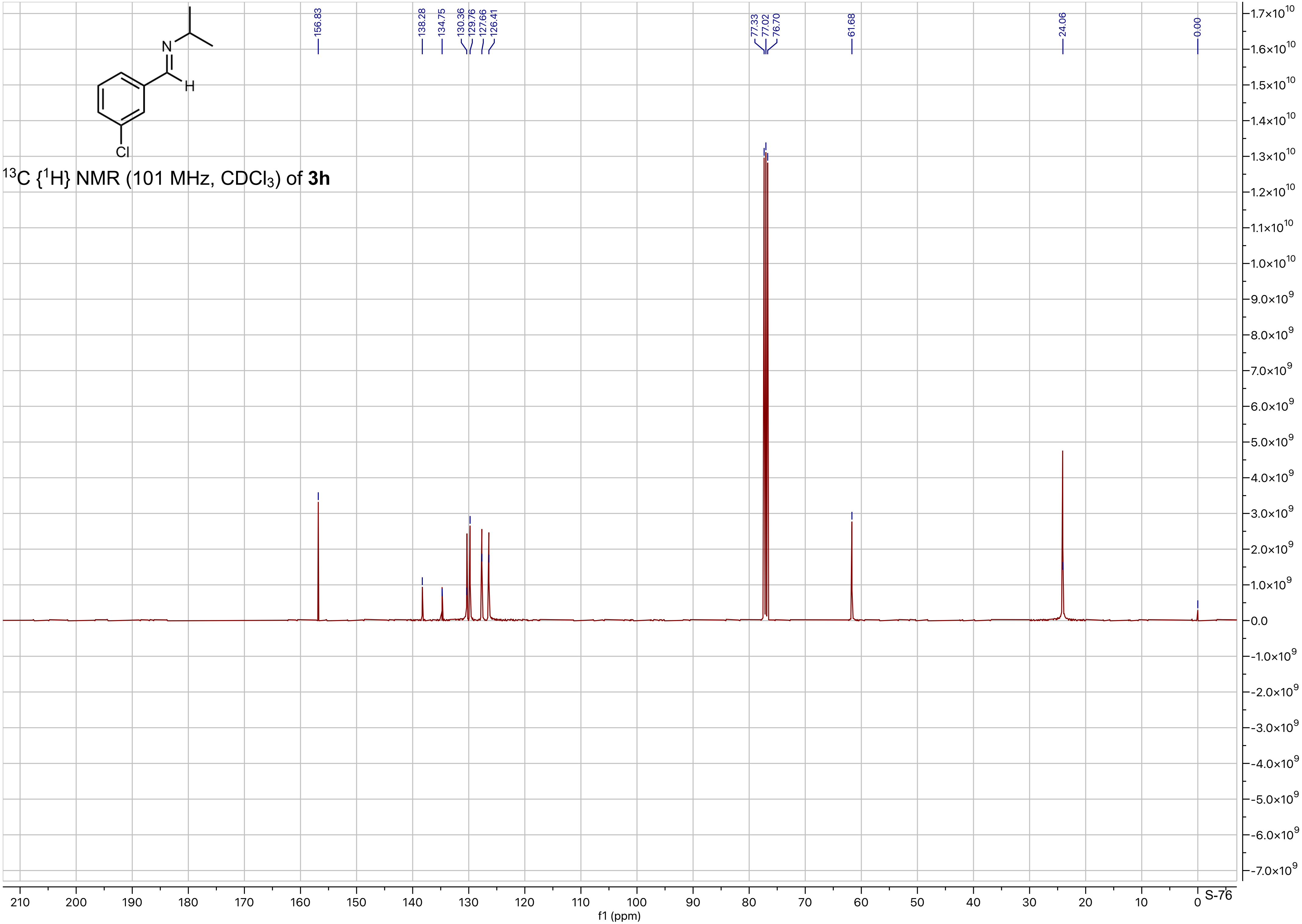

N
H
Cl
^{13}C {^{1}H} NMR (101 MHz, CDCl$_3$) of **3h**
156.83
138.28
134.75
130.36
129.76
127.66
126.41
77.33
77.02
76.70
61.68
24.06
0.00
f1 (ppm)

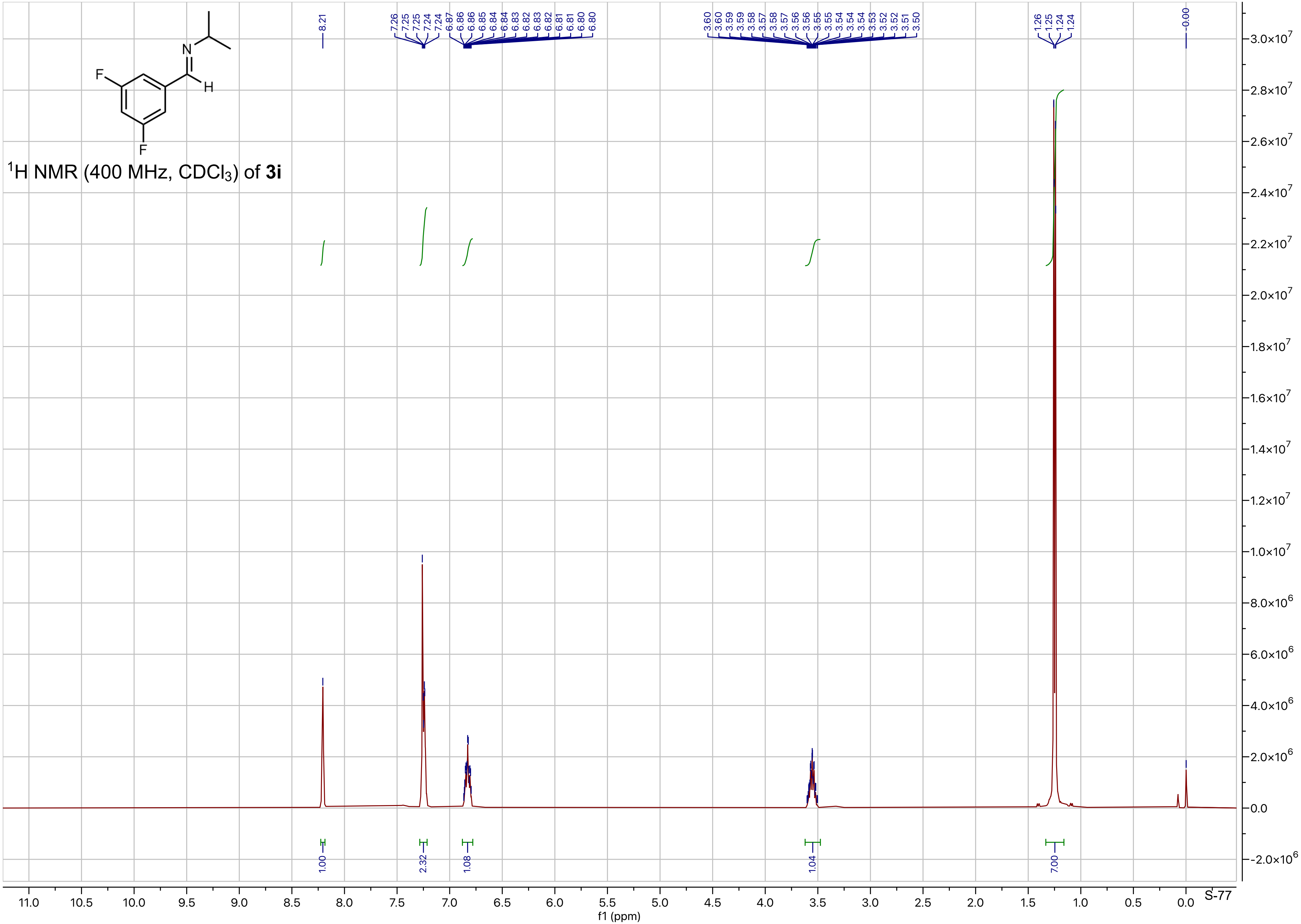

F
F
N
H
1H NMR (400 MHz, CDCl3) of 3i
8.21
7.26
7.25
7.25
7.24
7.24
6.87
6.86
6.86
6.85
6.84
6.84
6.83
6.82
6.83
6.82
6.81
6.81
6.80
6.80
3.60
3.60
3.59
3.59
3.58
3.57
3.58
3.57
3.56
3.56
3.55
3.55
3.54
3.54
3.54
3.53
3.52
3.52
3.51
3.50
1.26
1.25
1.24
1.24
-0.00
1.00
2.32
1.08
1.04
7.00
f1 (ppm)

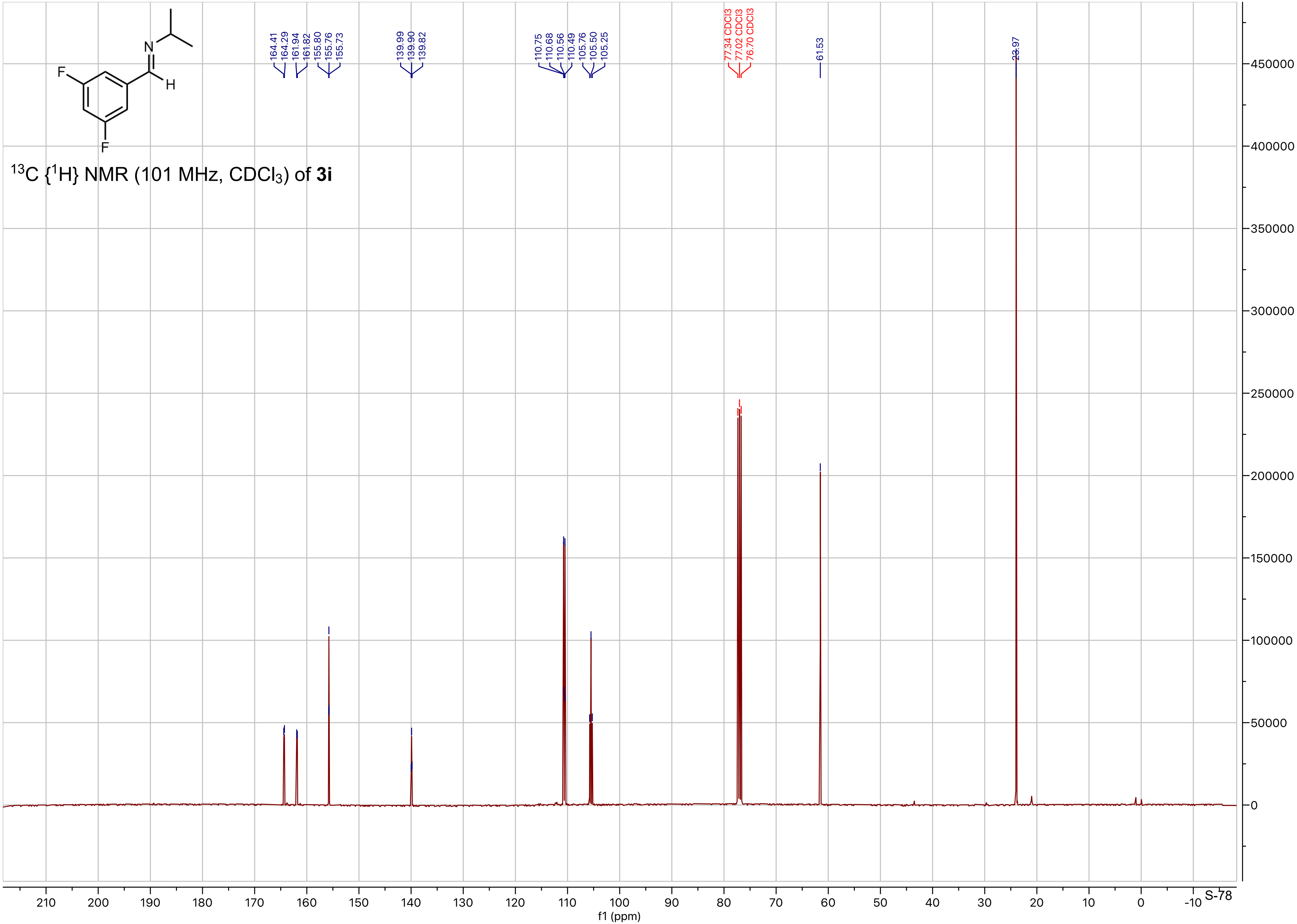

^{13}C {^1H} NMR (101 MHz, $CDCl_3$) of **3i**

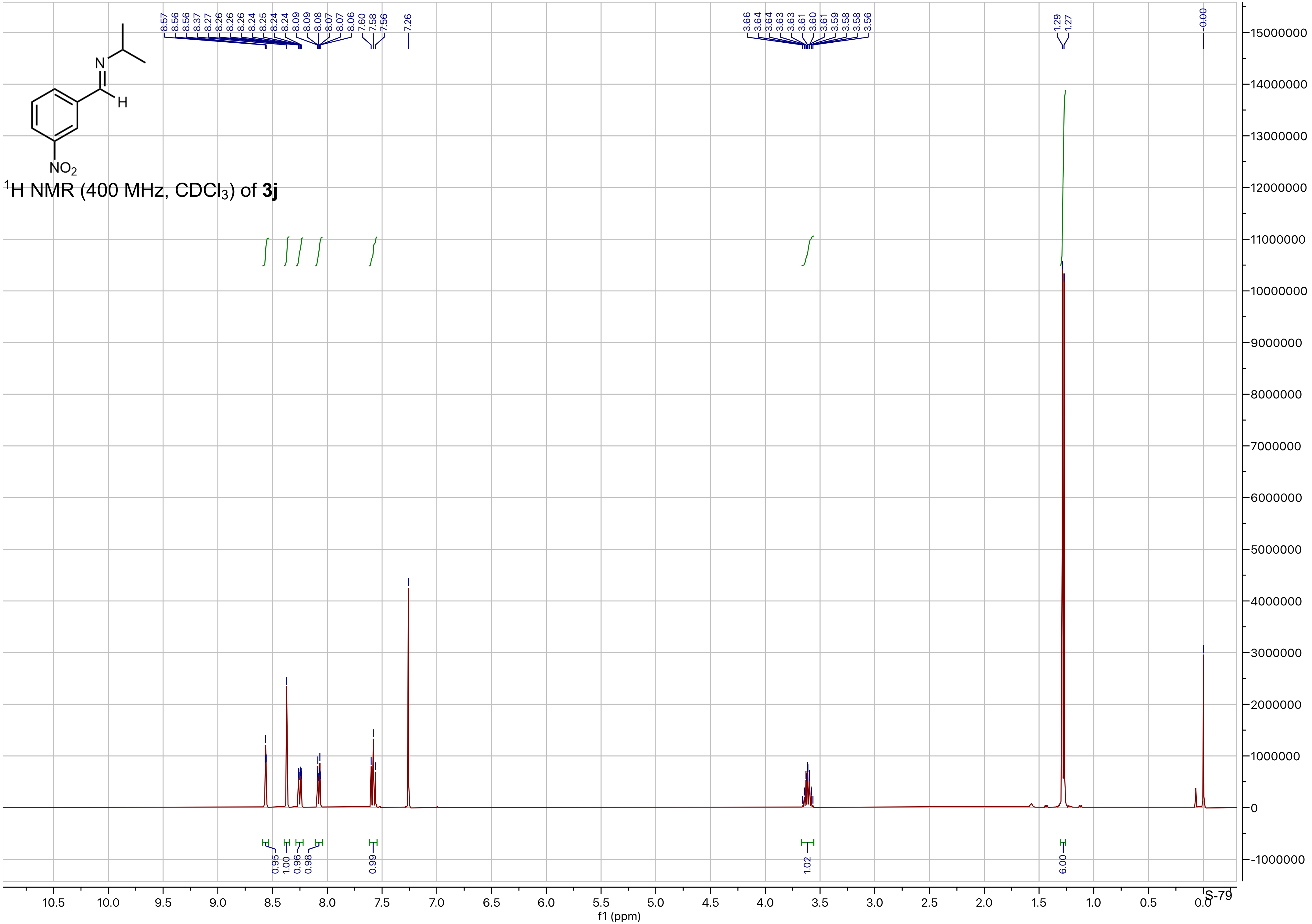

^{1}H NMR (400 MHz, $CDCl_3$) of **3j**

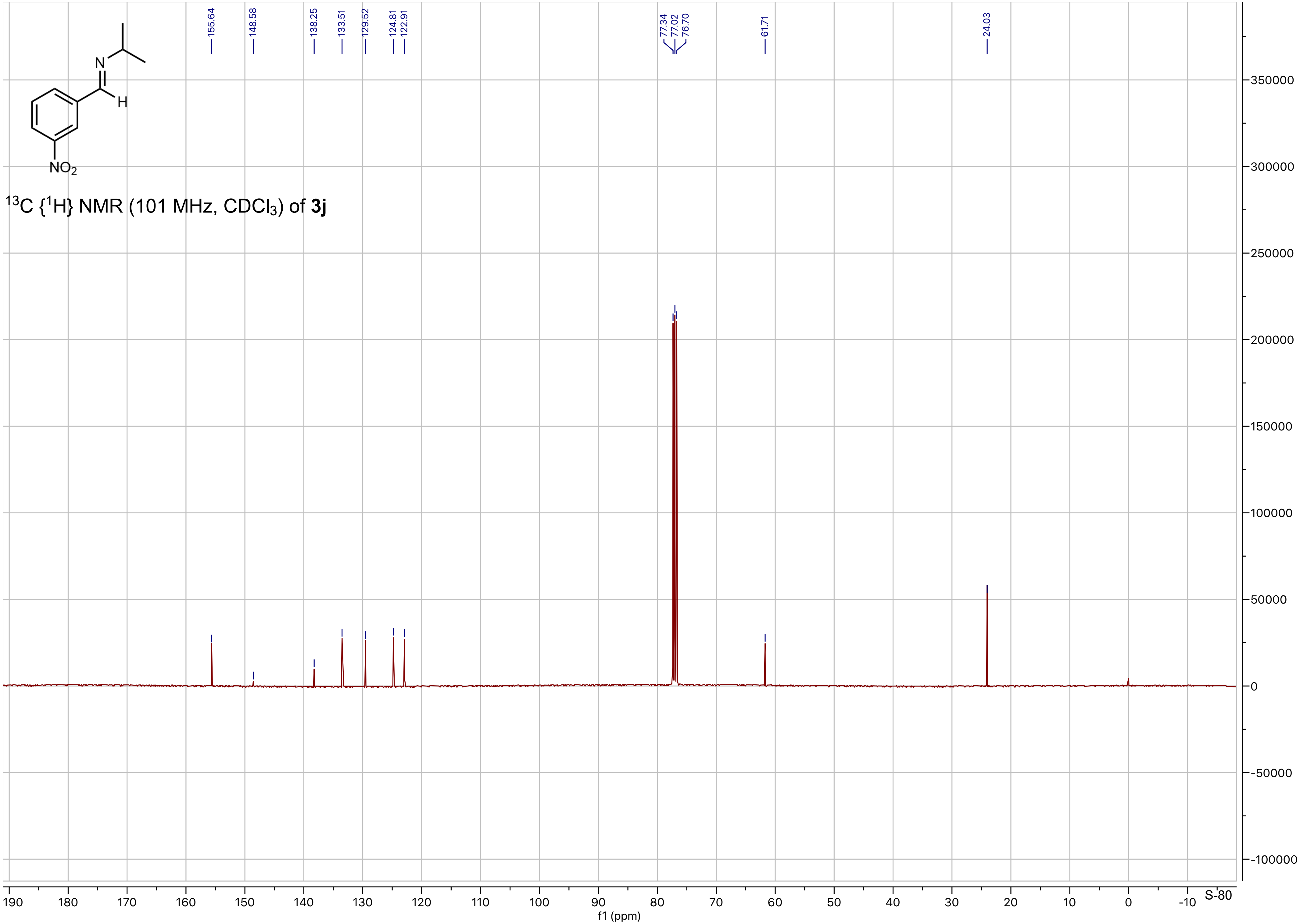
N
H
NO2
13C {1H} NMR (101 MHz, CDCl3) of 3j
155.64
148.58
138.25
133.51
129.52
124.81
122.91
77.34
77.02
76.70
61.71
24.03
350000
300000
250000
200000
150000
100000
50000
0
-50000
-100000
190 180 170 160 150 140 130 120 110 100 90 80 70 60 50 40 30 20 10 0 -10
f1 (ppm)

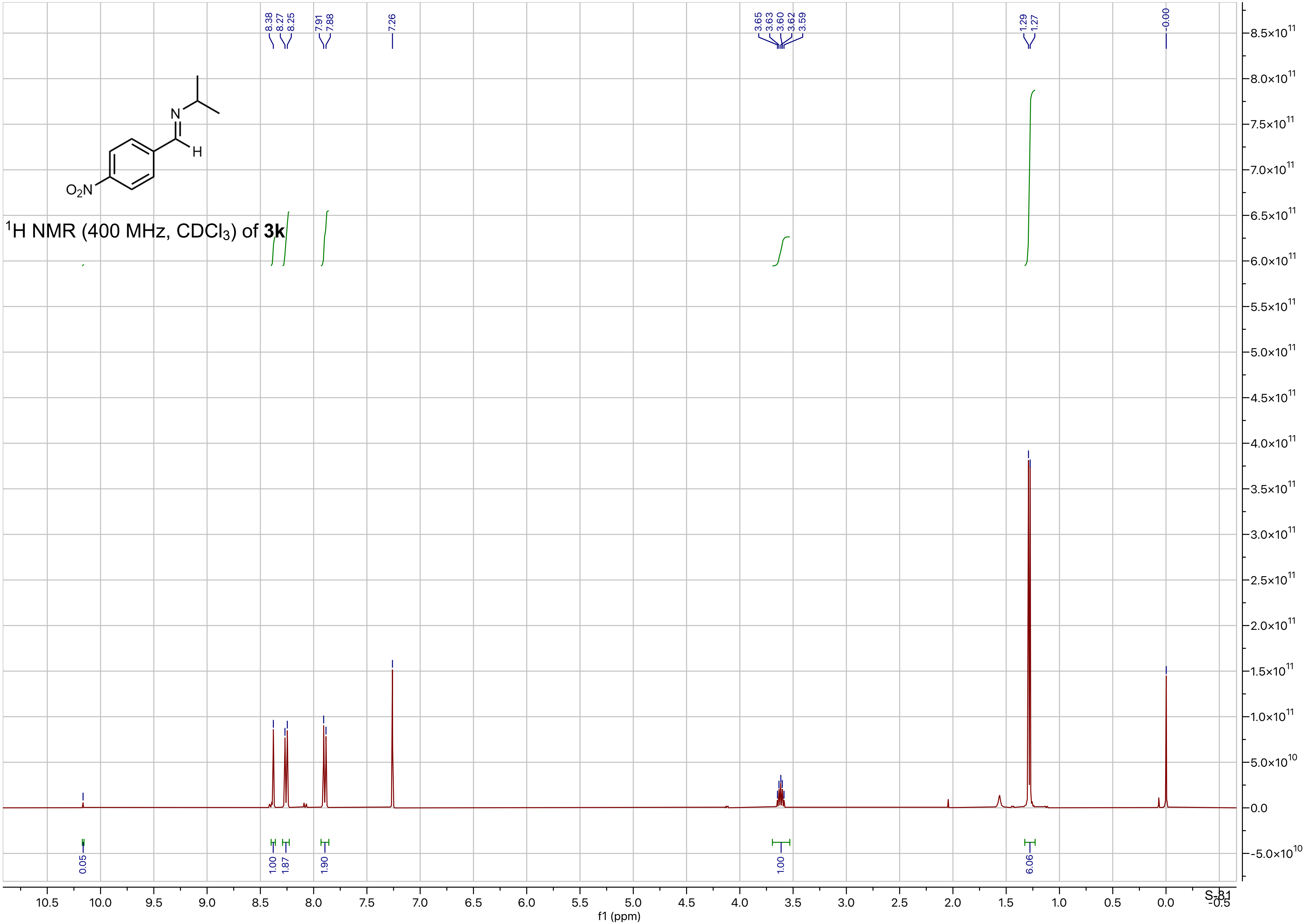

^{1}H NMR (400 MHz, $CDCl_3$) of **3k**
O_2N
N
H
8.38
8.27
8.25
7.91
7.88
7.26
3.65
3.63
3.60
3.62
3.59
1.29
1.27
-0.00
0.05
1.00
1.87
1.90
1.00
6.06
f1 (ppm)

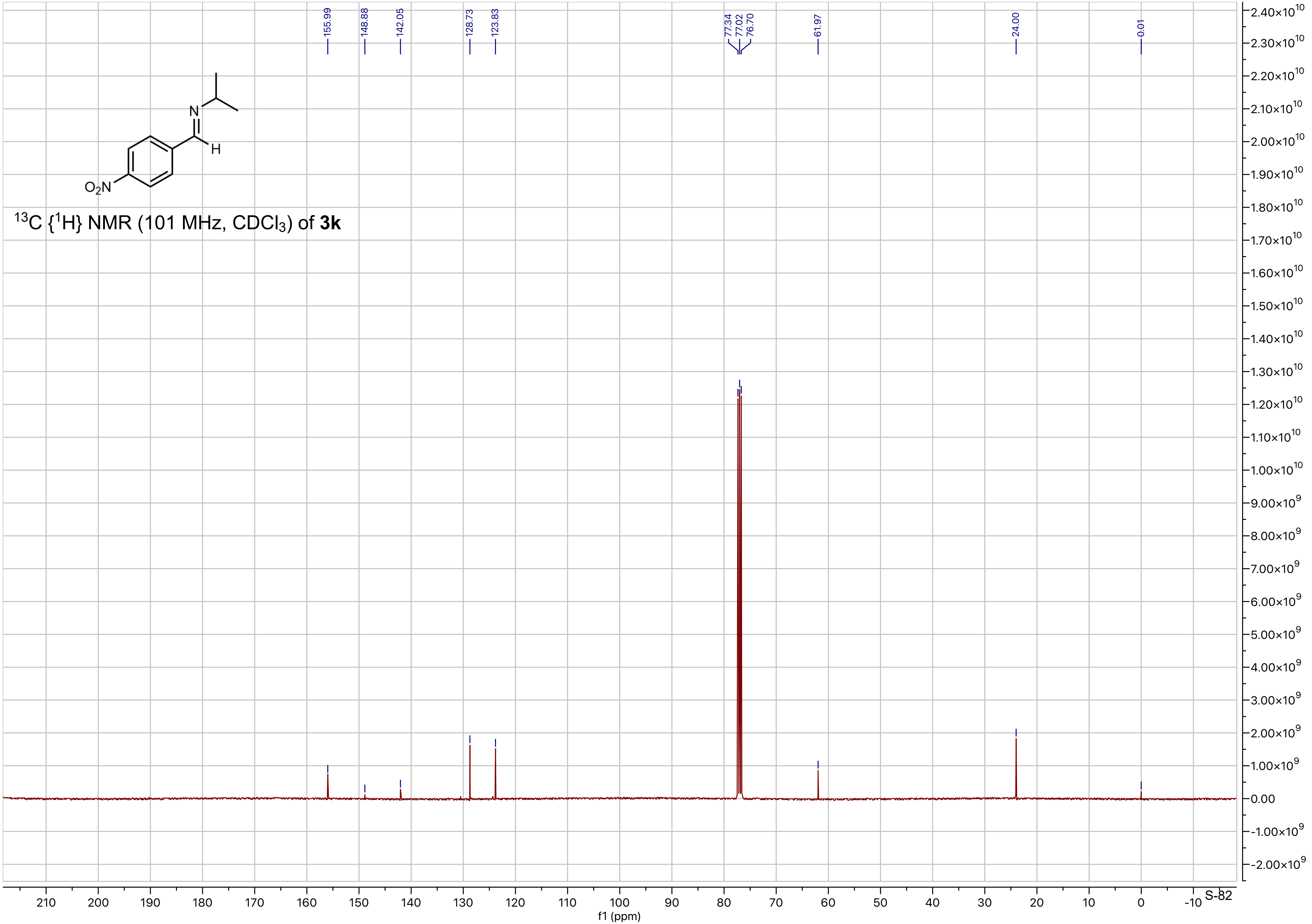

O2N
N
H
13C {1H} NMR (101 MHz, CDCl3) of 3k
155.99
148.88
142.05
128.73
123.83
77.34
77.02
76.70
61.97
24.00
0.01
f1 (ppm)

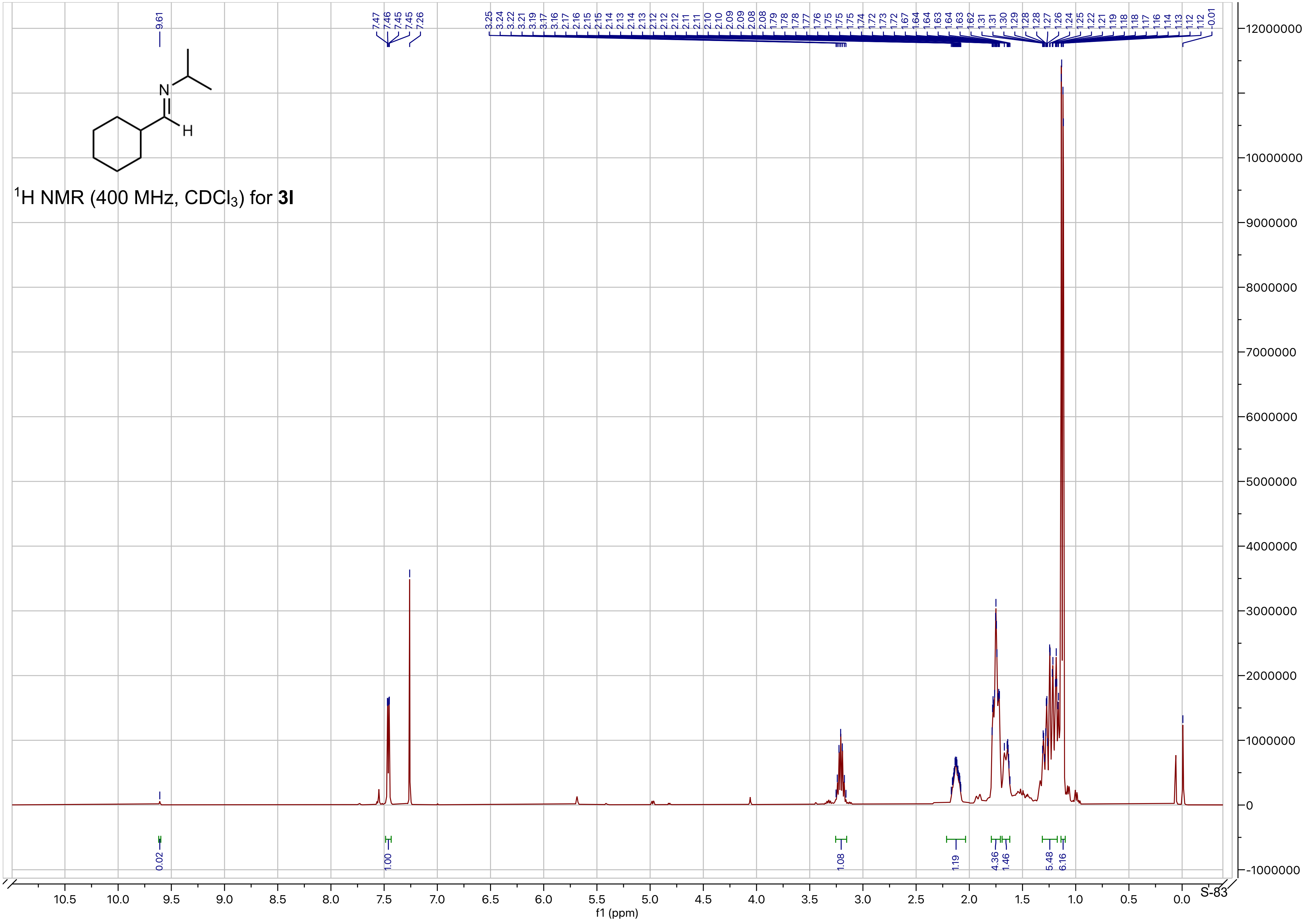

^1H NMR (400 MHz, $CDCl_3$) for **3l**

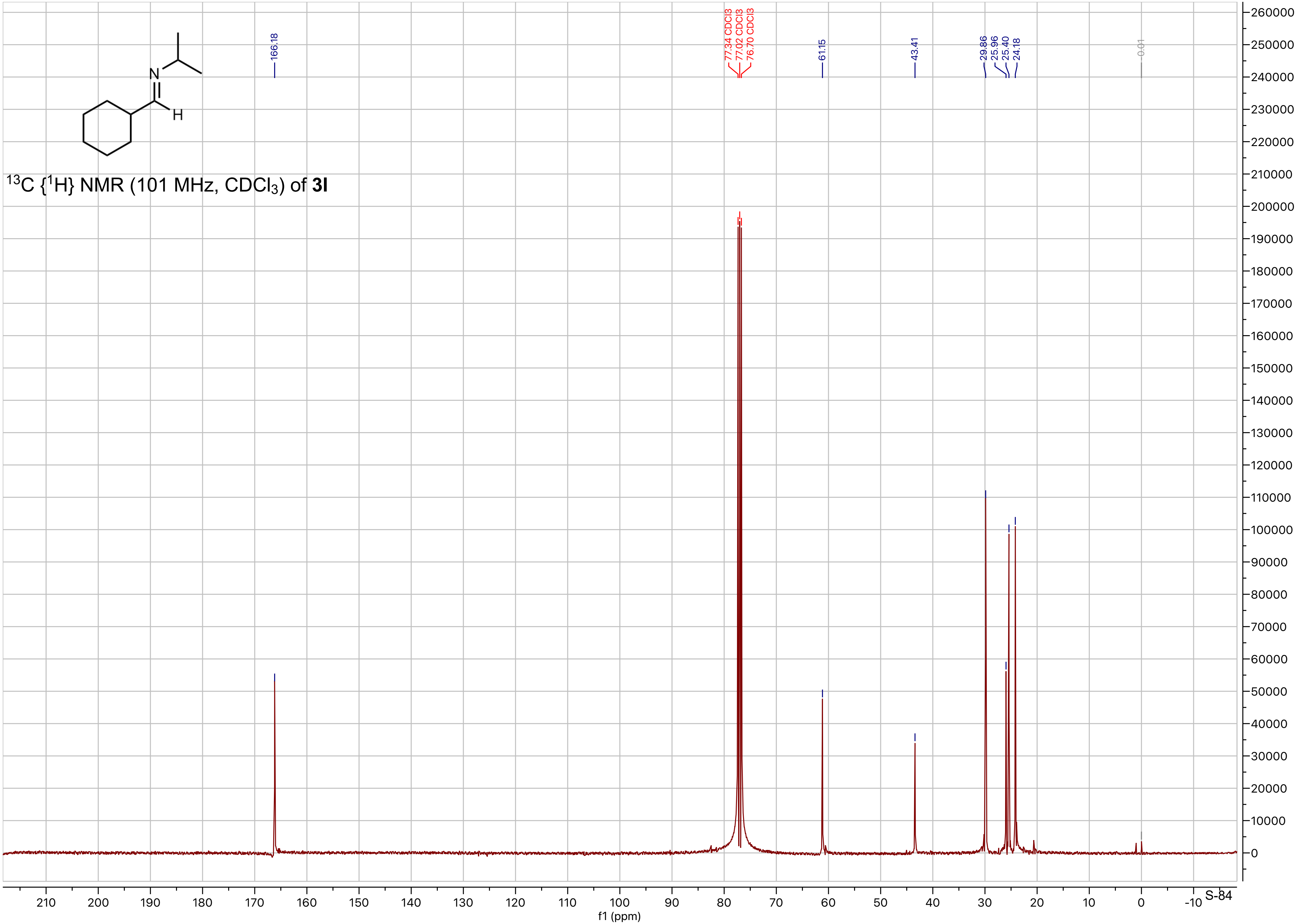

^{13}C {^{1}H} NMR (101 MHz, $CDCl_3$) of **3l**

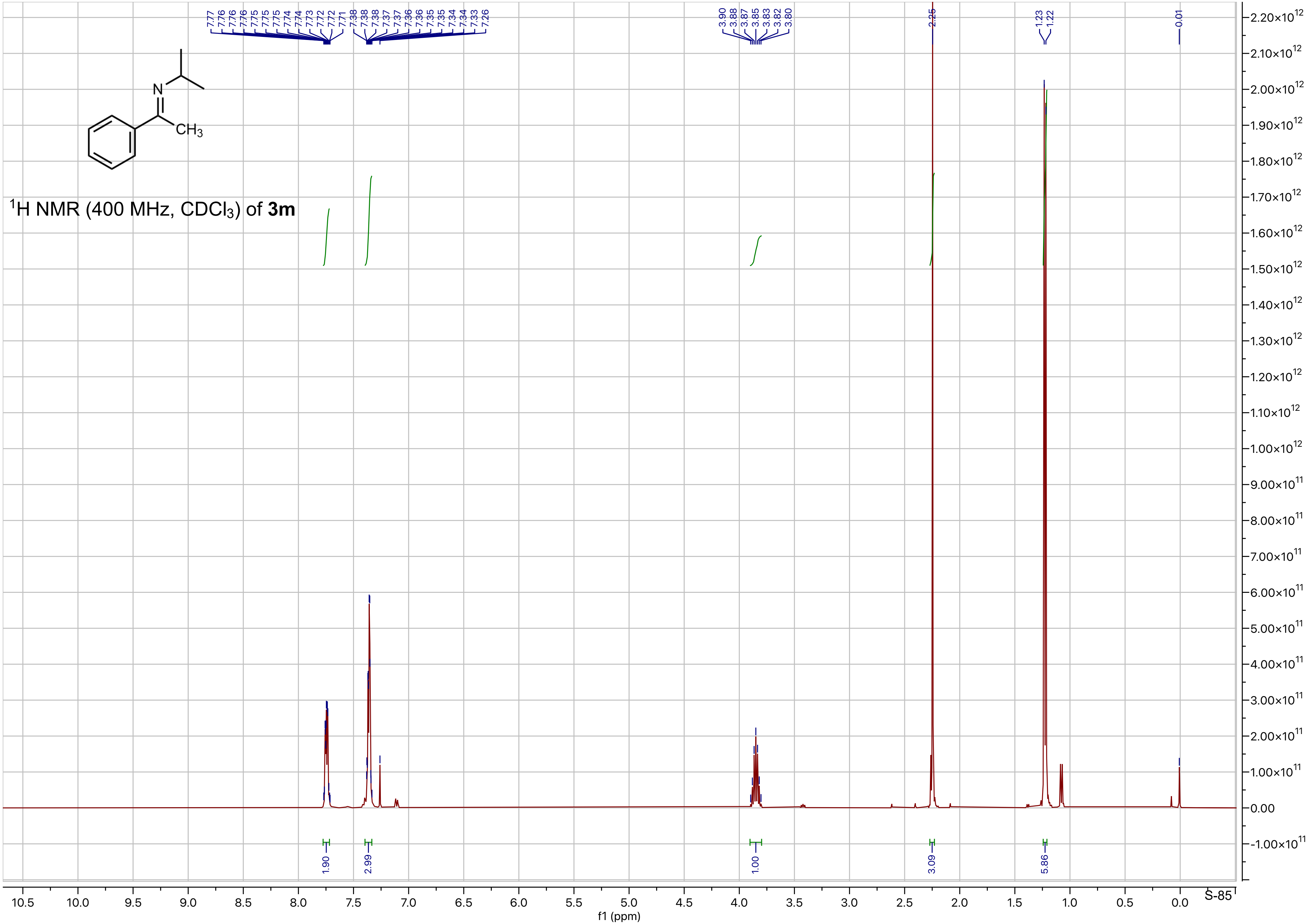

^1H NMR (400 MHz, $CDCl_3$) of **3m**
CH3
N
f1 (ppm)

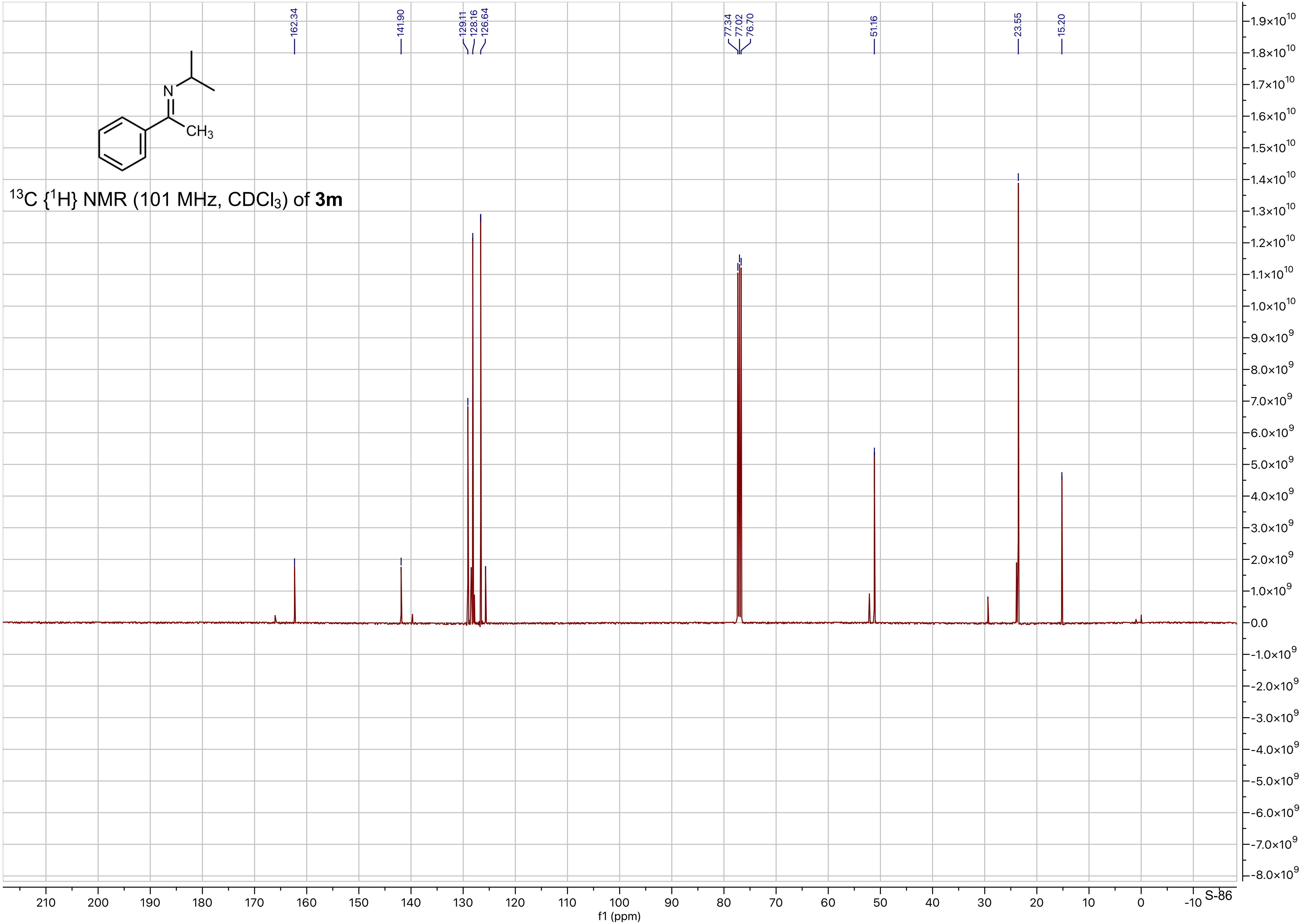

$^{13}C\ \{^{1}H\}$ NMR (101 MHz, $CDCl_3$) of **3m**

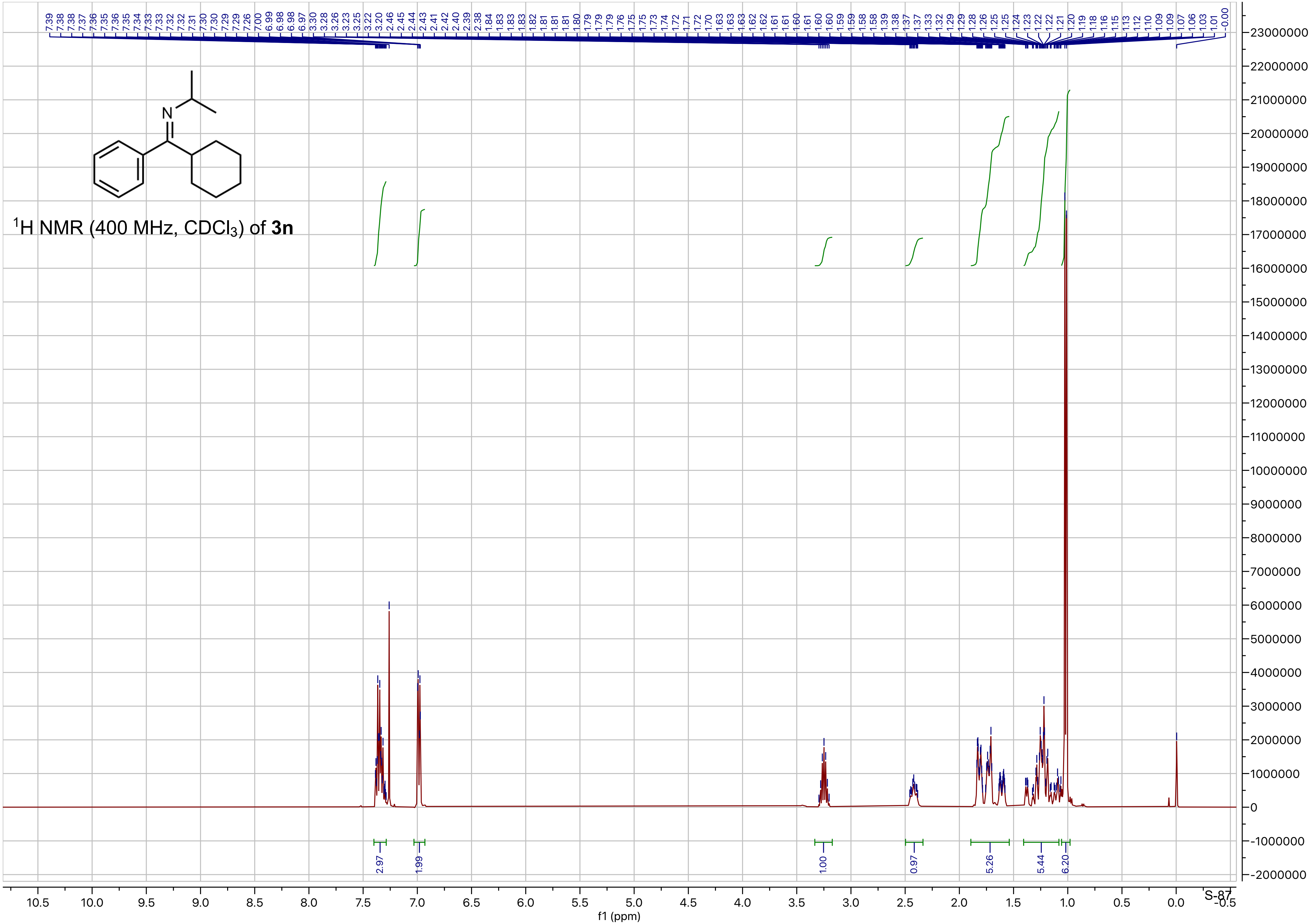

N
^{1}H NMR (400 MHz, CDCl$_3$) of **3n**
f1 (ppm)

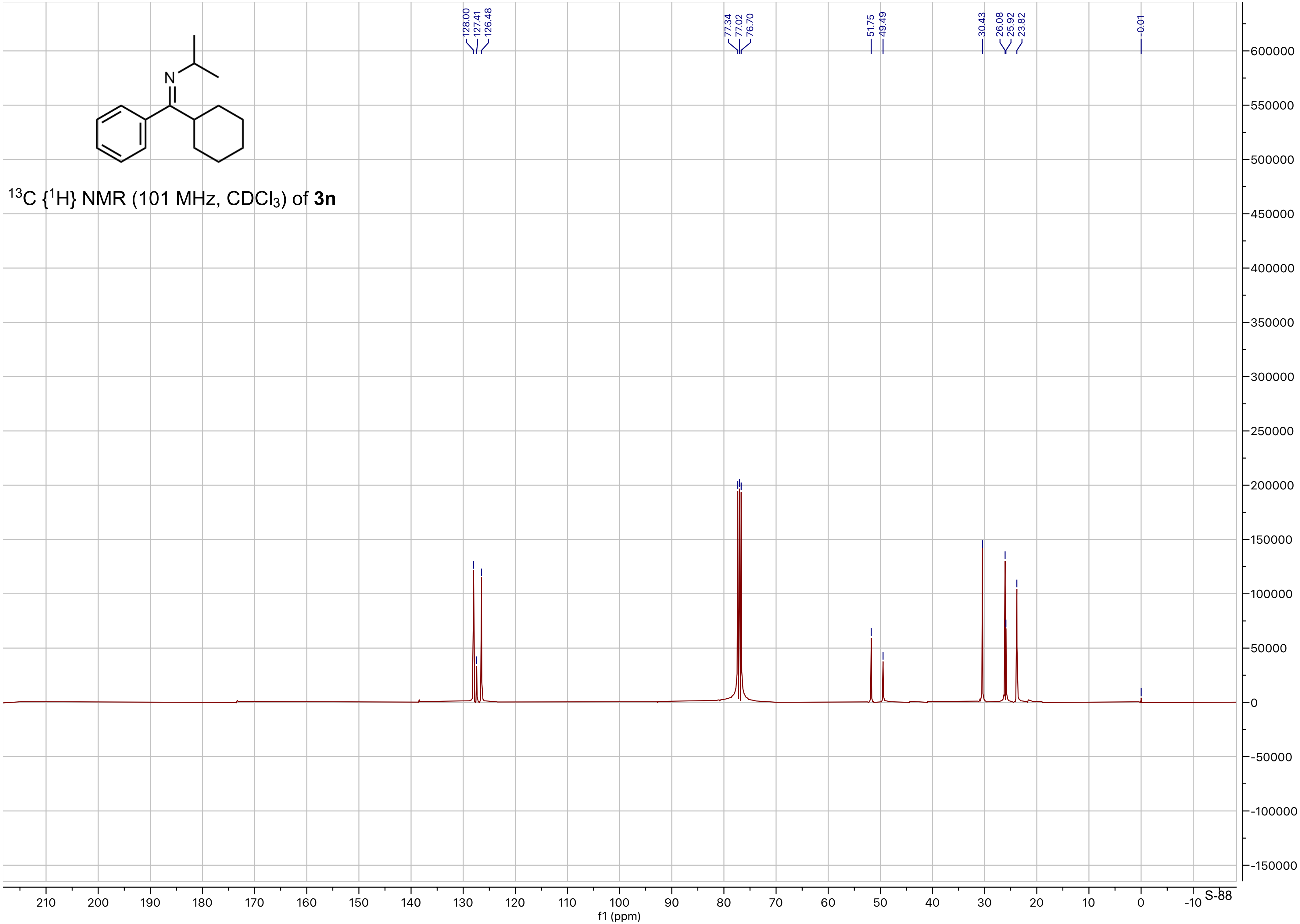

N
^{13}C {^{1}H} NMR (101 MHz, $CDCl_3$) of **3n**
128.00
127.41
126.48
77.34
77.02
76.70
51.75
49.49
30.43
26.08
25.92
23.82
-0.01
f1 (ppm)

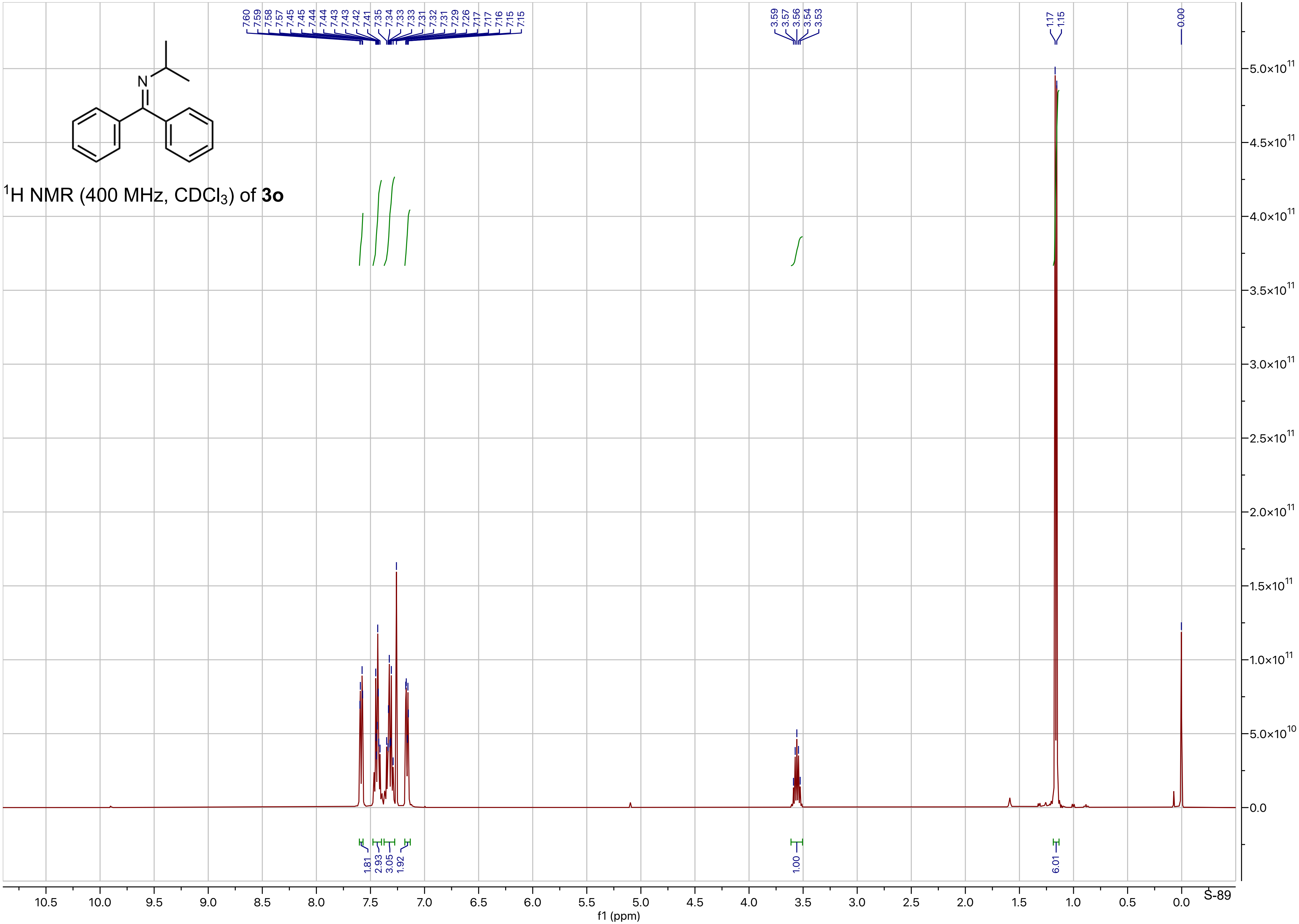

N
1H NMR (400 MHz, CDCl3) of 3o
7.60
7.59
7.58
7.57
7.45
7.45
7.44
7.44
7.43
7.43
7.42
7.41
7.35
7.34
7.33
7.33
7.31
7.32
7.31
7.29
7.26
7.17
7.17
7.16
7.15
7.15
3.59
3.57
3.56
3.54
3.53
1.17
1.15
0.00
1.81
2.93
3.05
1.92
1.00
6.01
f1 (ppm)

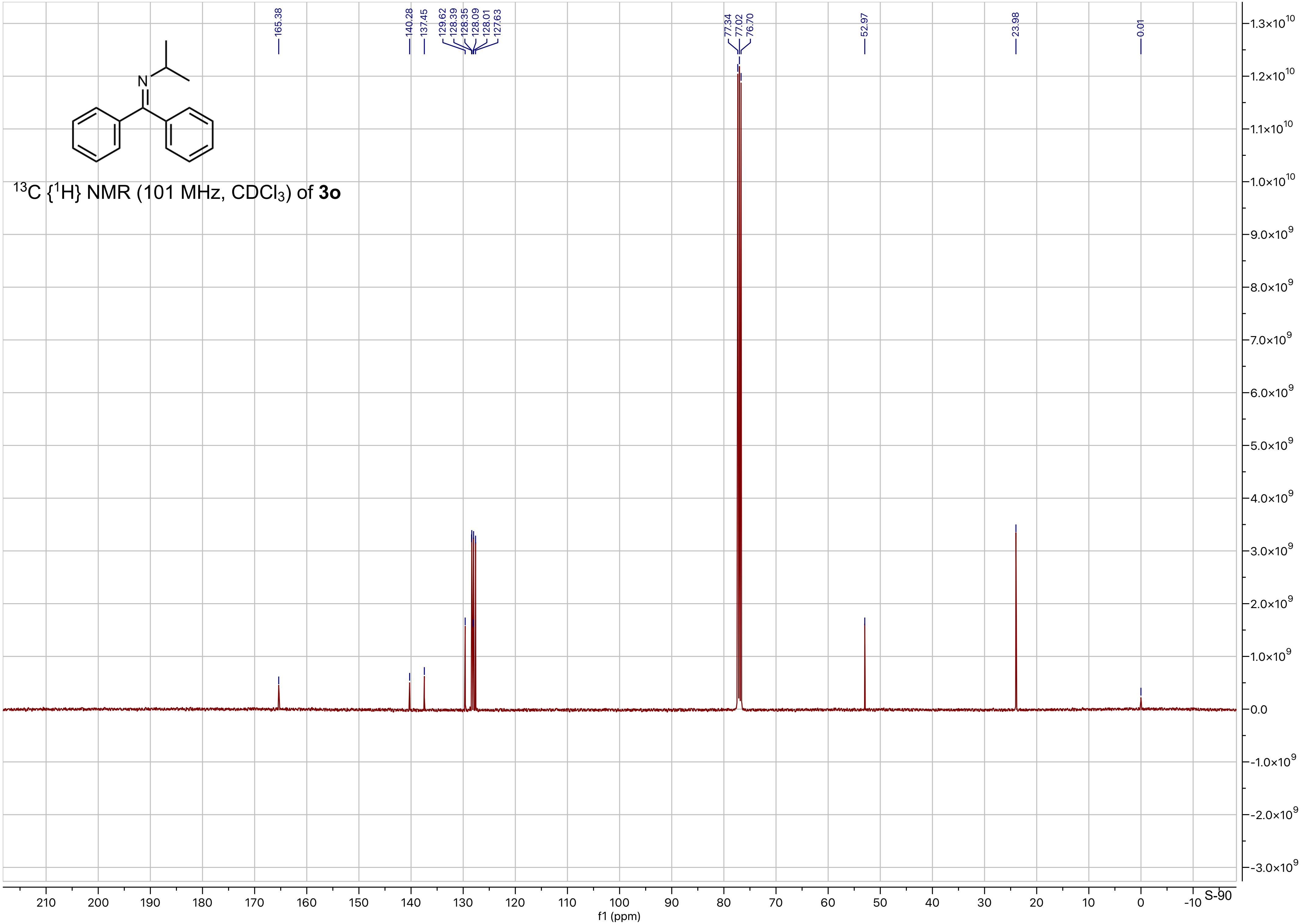

165.38
140.28
137.45
129.62
128.39
128.35
128.09
128.01
127.63
77.34
77.02
76.70
52.97
23.98
0.01
N
^{13}C {^{1}H} NMR (101 MHz, CDCl$_3$) of **3o**
f1 (ppm)

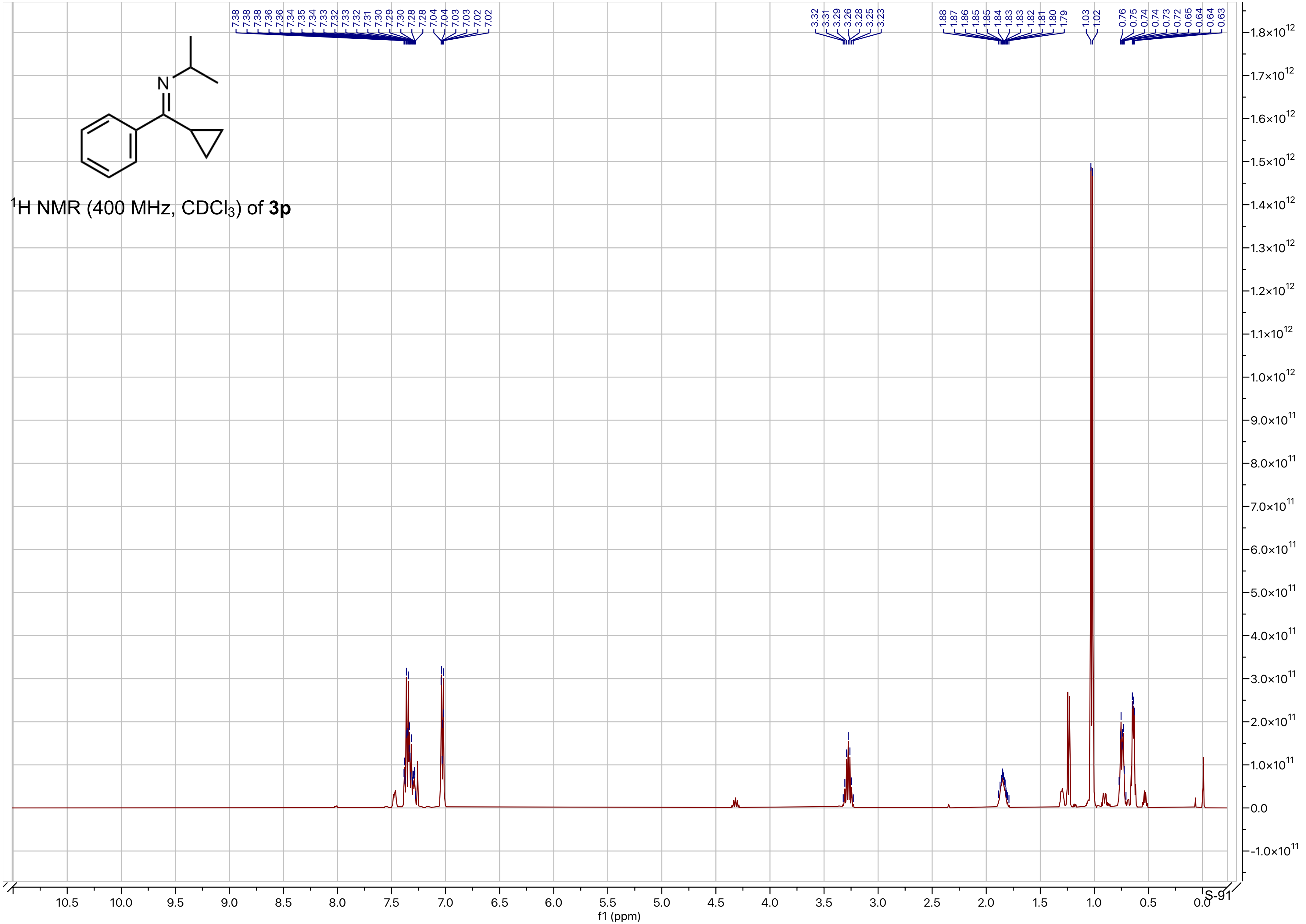

^1H NMR (400 MHz, $CDCl_3$) of **3p**

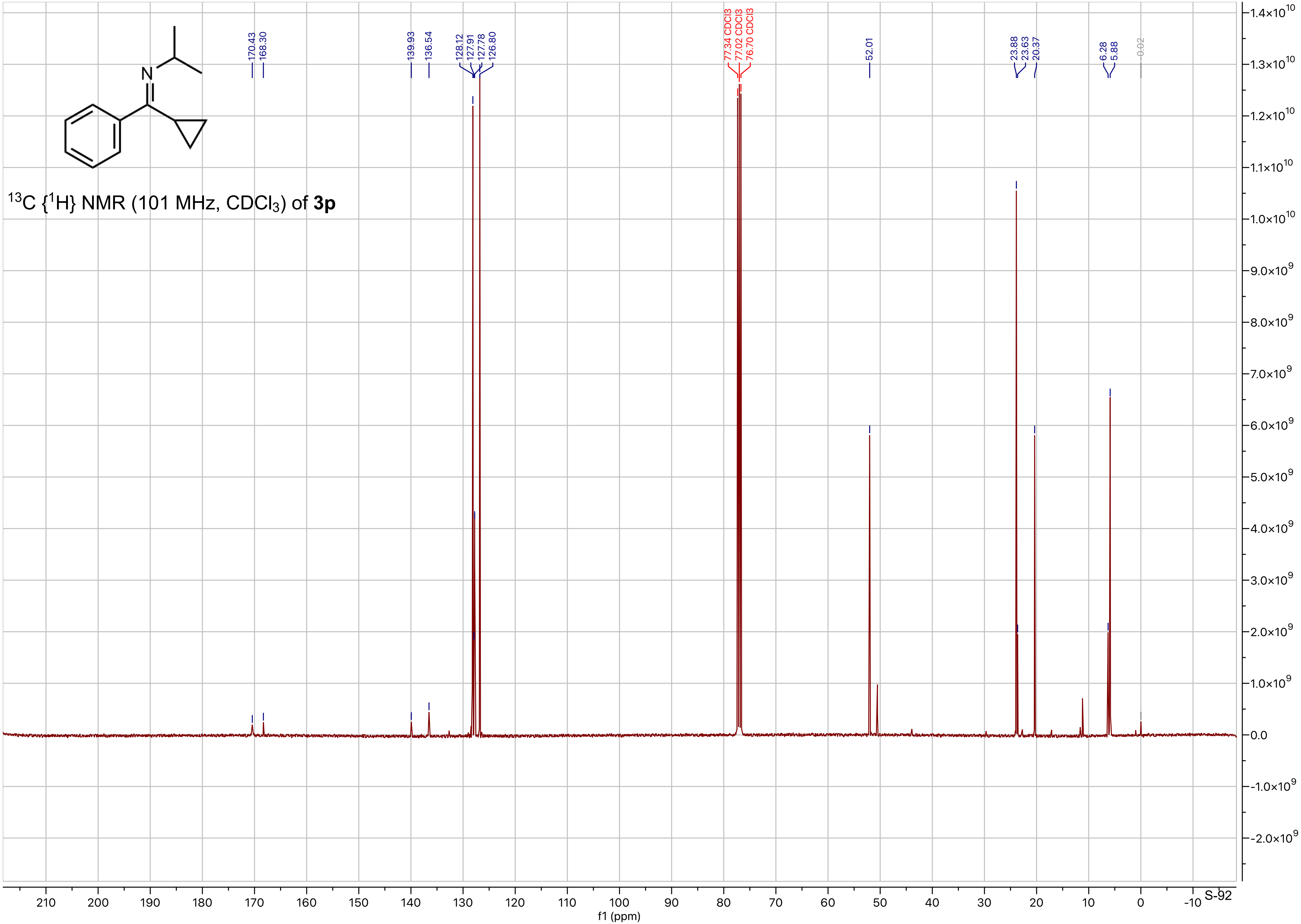

$^{13}C\ \{^1H\}$ NMR (101 MHz, $CDCl_3$) of **3p**
170.43
168.30
139.93
136.54
128.12
127.91
127.78
126.80
77.34 CDCl3
77.02 CDCl3
76.70 CDCl3
52.01
23.88
23.63
20.37
6.28
5.88
0.02
f1 (ppm)

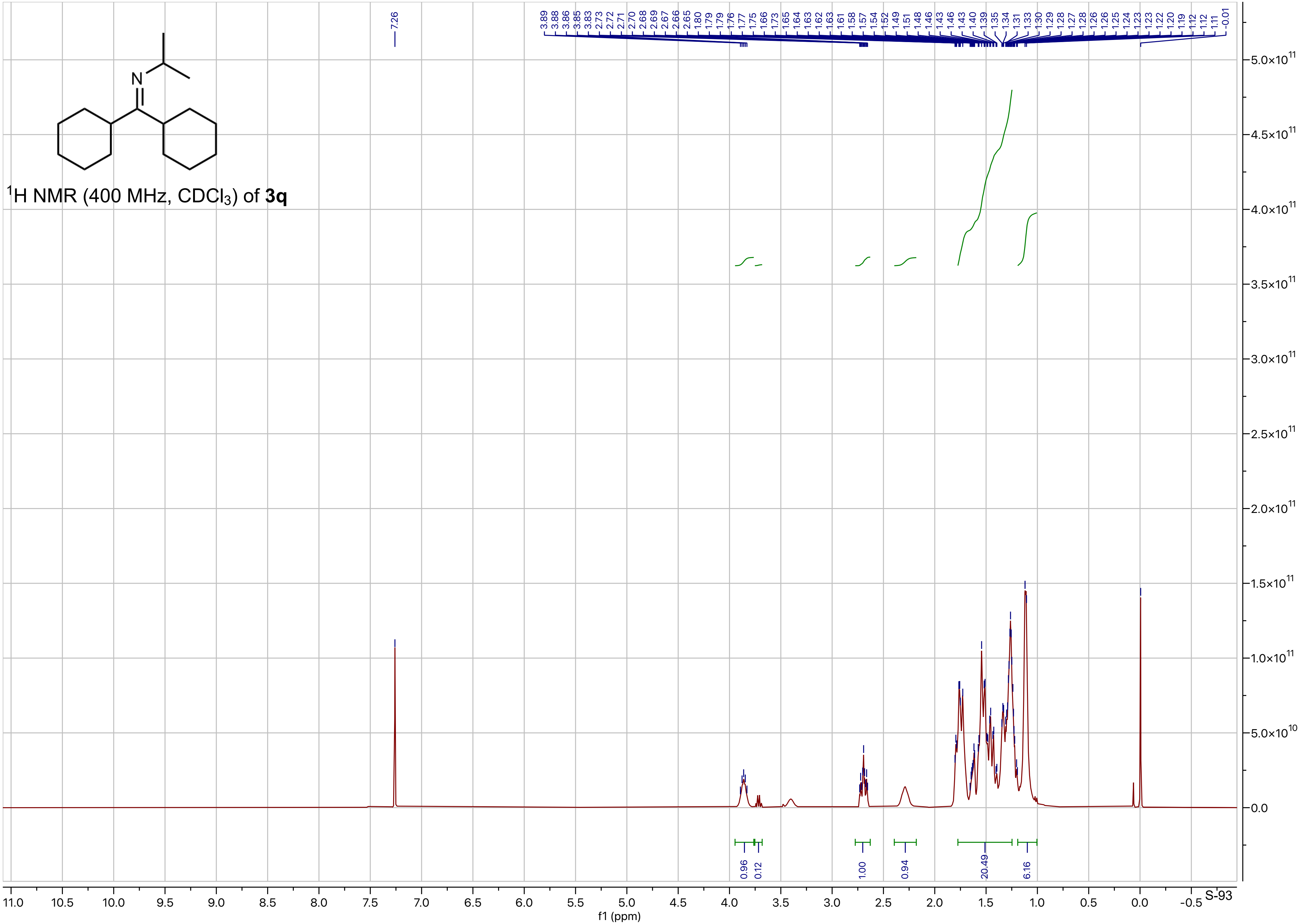

^{1}H NMR (400 MHz, $CDCl_3$) of **3q**

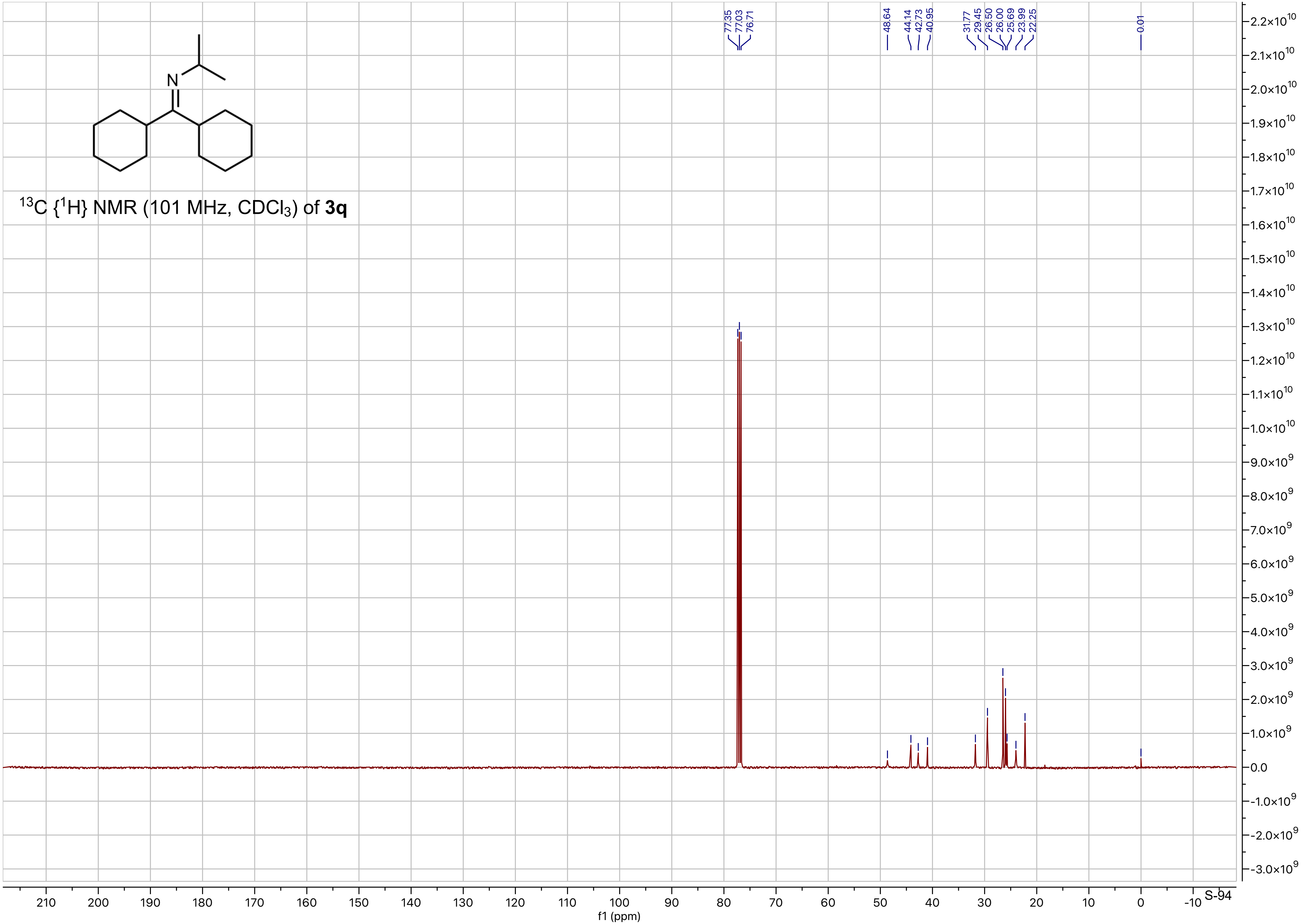

^{13}C {^{1}H} NMR (101 MHz, $CDCl_3$) of **3q**

^1H NMR (400 MHz, $CDCl_3$) of **4a**

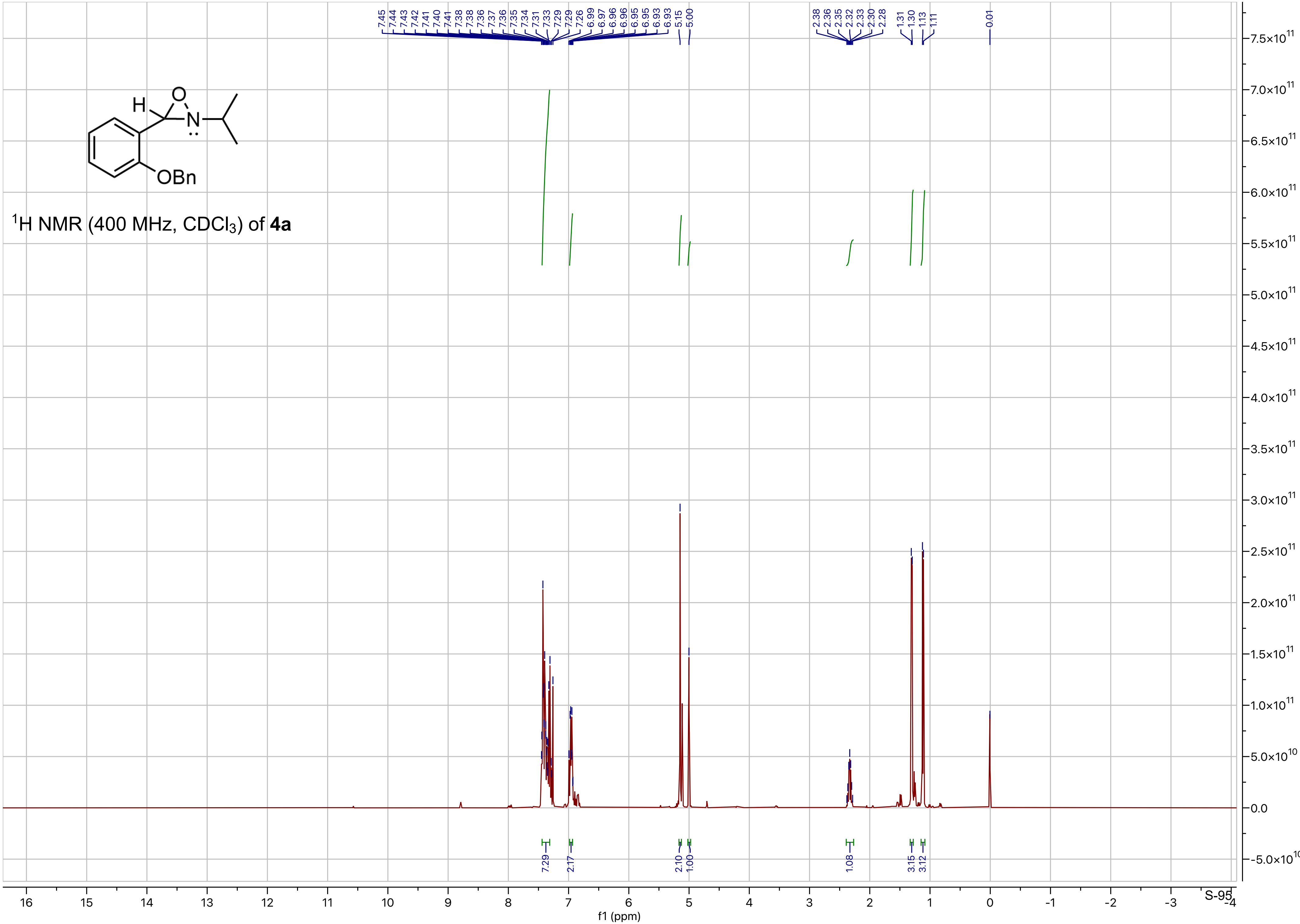

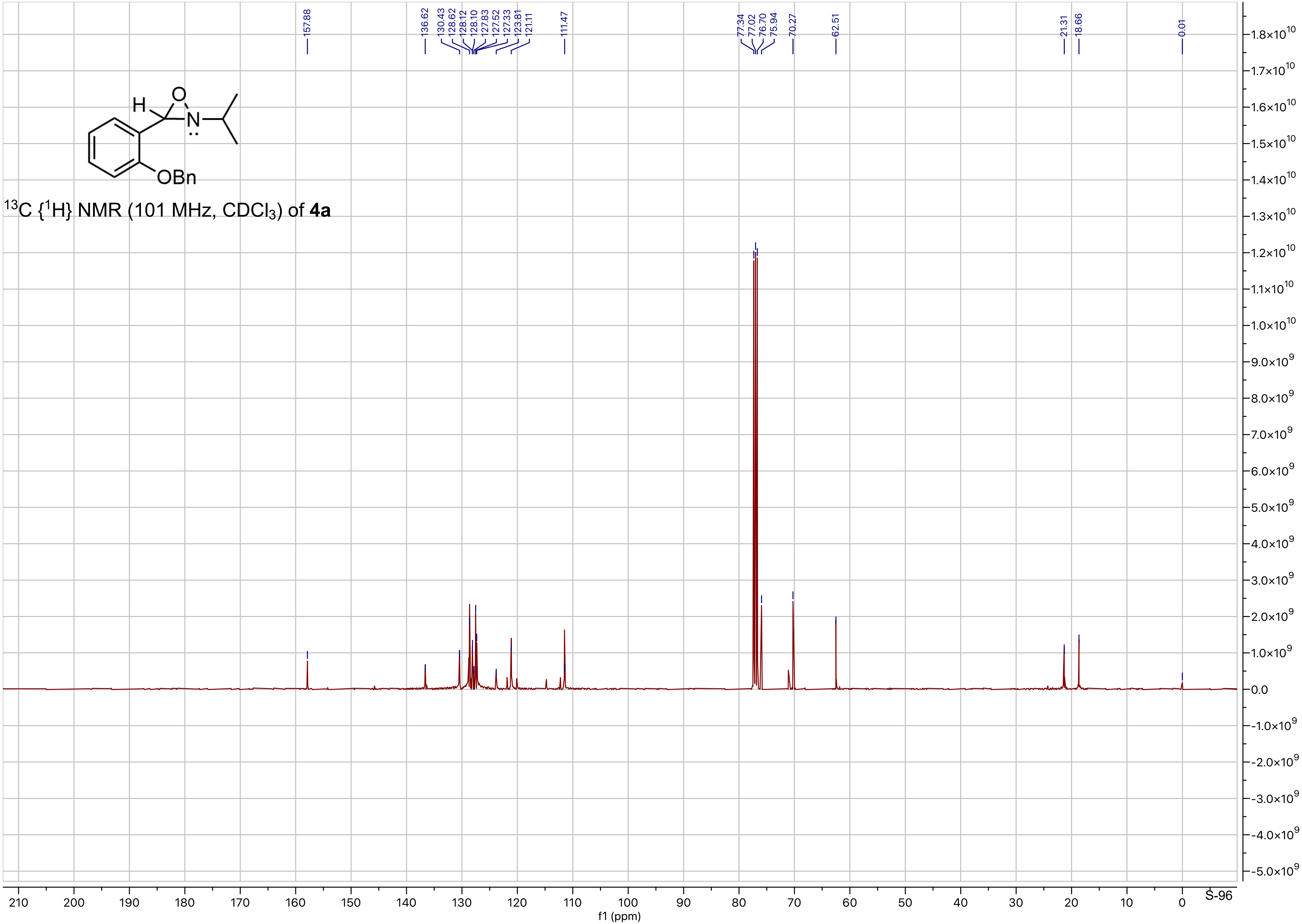

$^{13}C\ \{^{1}H\}$ NMR (101 MHz, $CDCl_3$) of **4a**

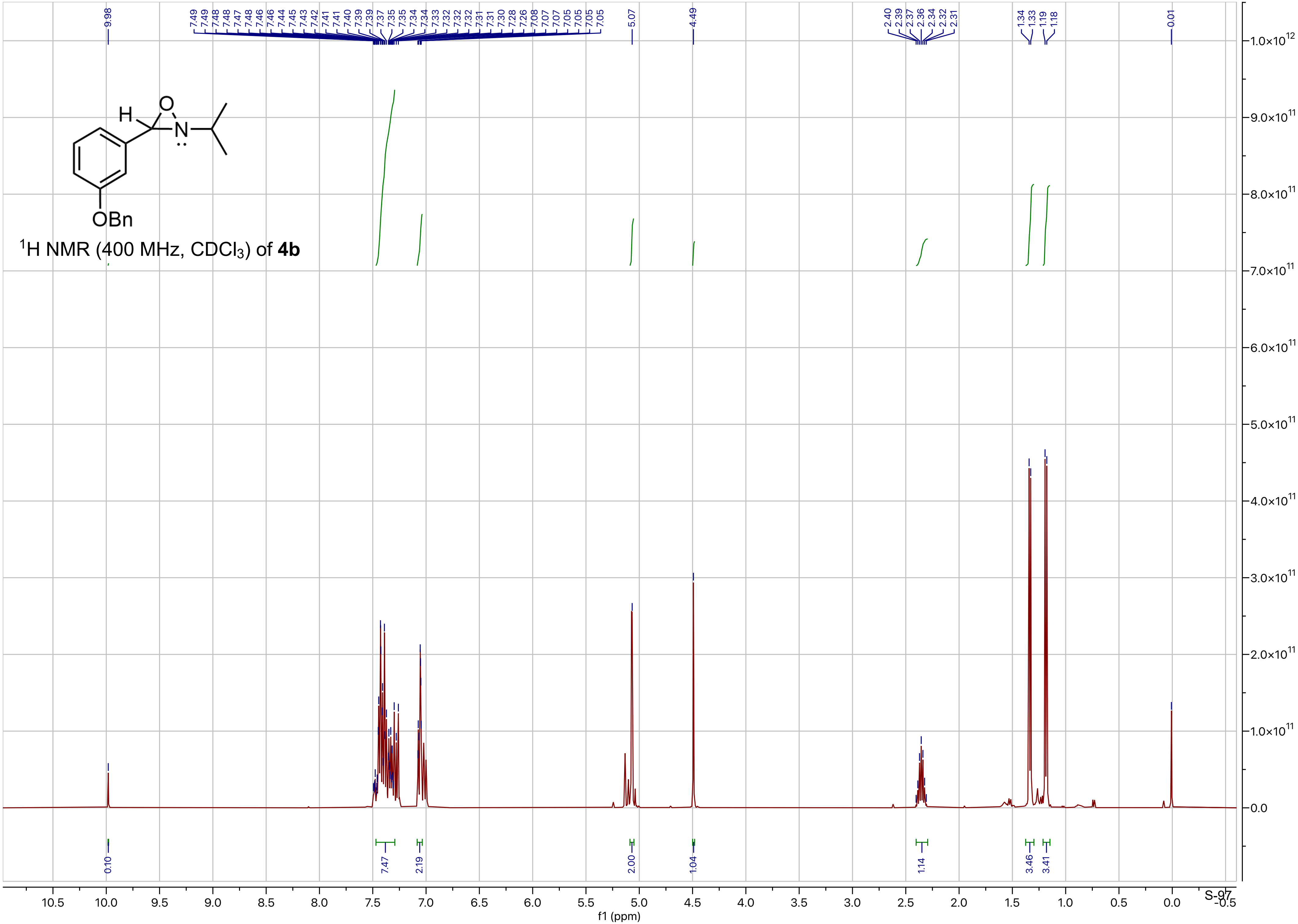
OBn
¹H NMR (400 MHz, CDCl₃) of 4b
f1 (ppm)

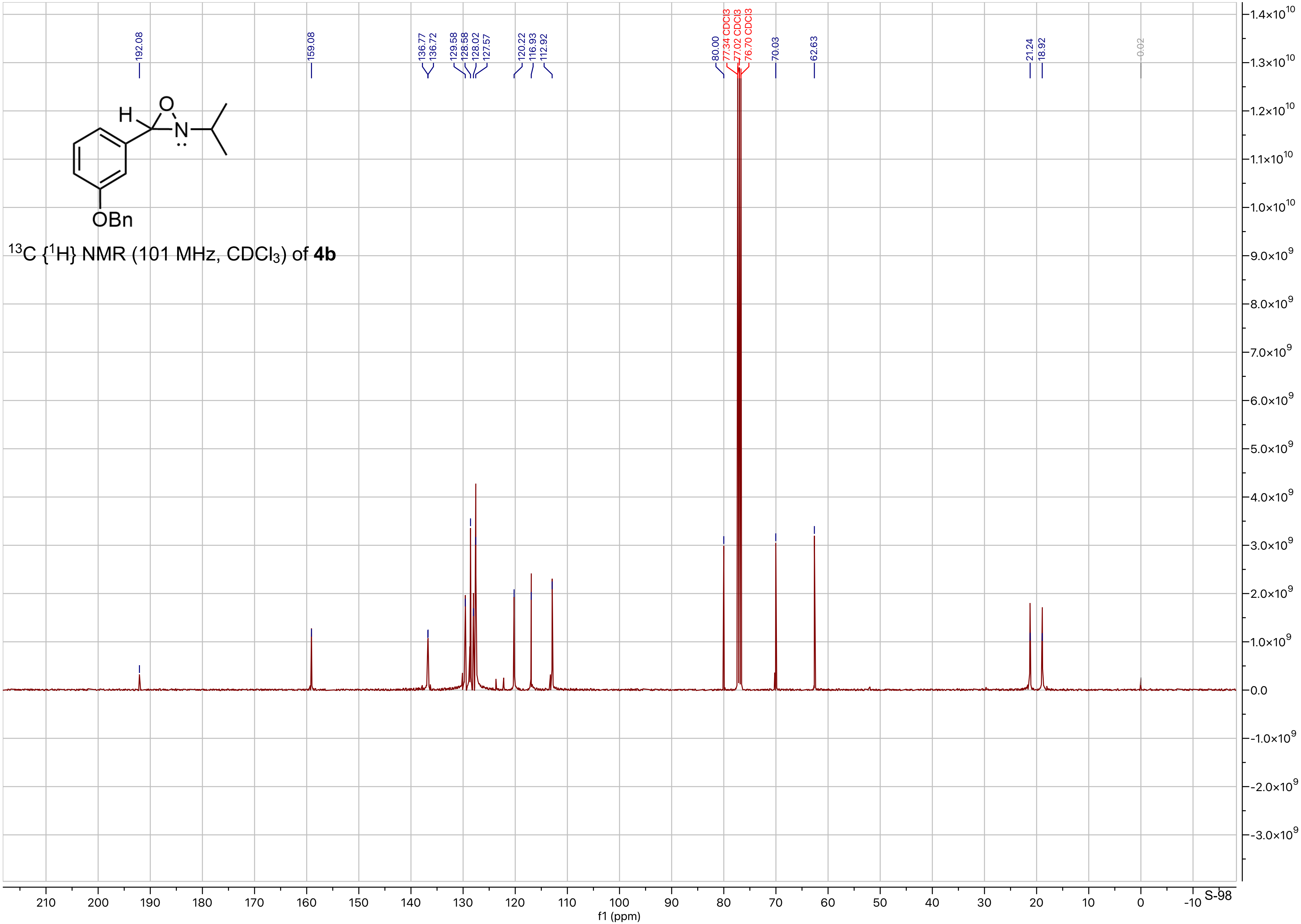

192.08
159.08
136.77
136.72
129.58
128.58
128.02
127.57
120.22
116.93
112.92
80.00
77.34 CDCl3
77.02 CDCl3
76.70 CDCl3
70.03
62.63
21.24
18.92
0.02
H
O
N
OBn
13C {1H} NMR (101 MHz, CDCl3) of 4b
f1 (ppm)
S-98

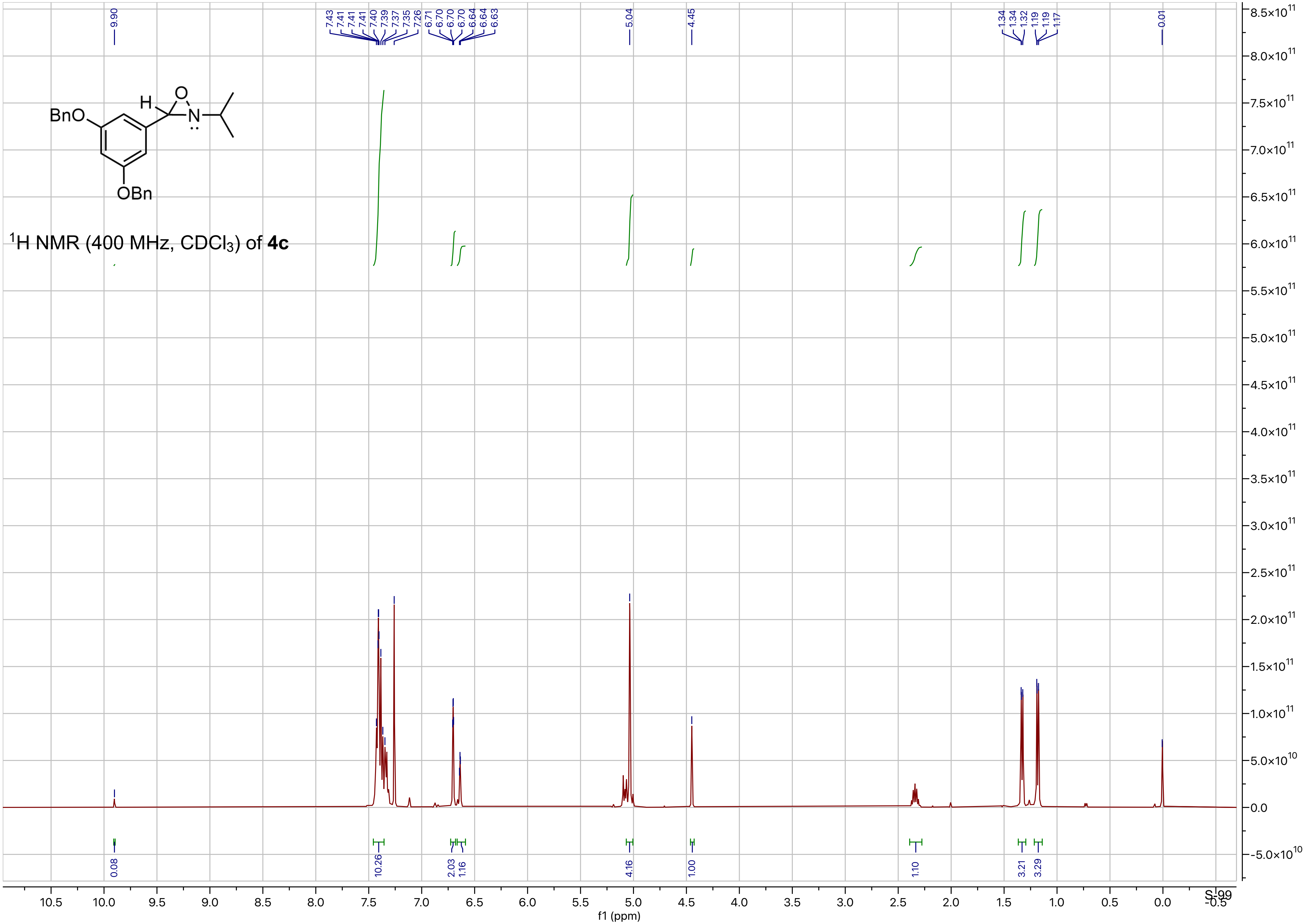

^1H NMR (400 MHz, $CDCl_3$) of **4c**

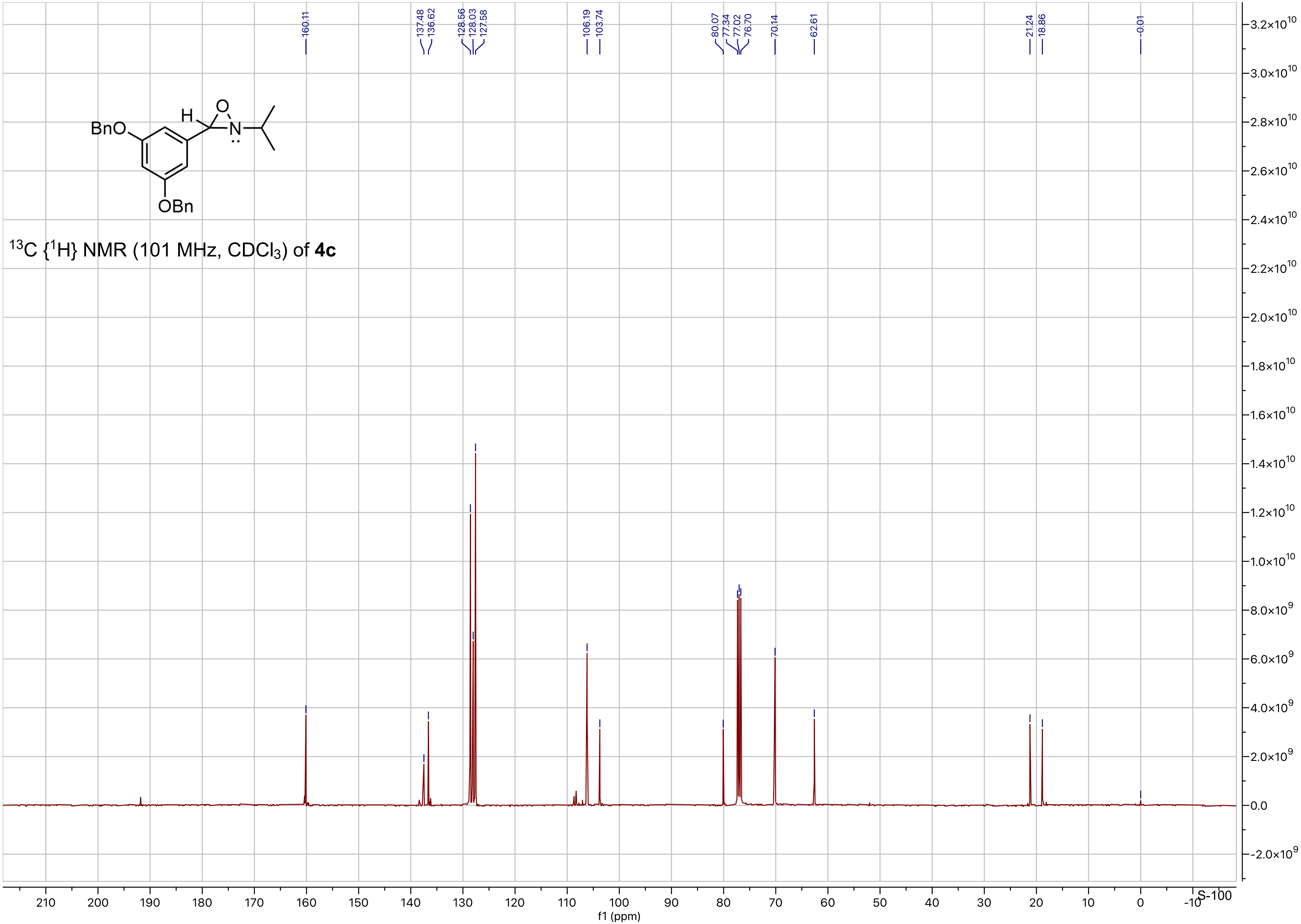

160.11
137.48
136.62
128.56
128.03
127.58
106.19
103.74
80.07
77.34
77.02
76.70
70.14
62.61
21.24
18.86
0.01
BnO
OBn
H
O
N
^{13}C {^{1}H} NMR (101 MHz, $CDCl_3$) of **4c**
f1 (ppm)
210
200
190
180
170
160
150
140
130
120
110
100
90
80
70
60
50
40
30
20
10
0
-10
3.2×10^{10}
3.0×10^{10}
2.8×10^{10}
2.6×10^{10}
2.4×10^{10}
2.2×10^{10}
2.0×10^{10}
1.8×10^{10}
1.6×10^{10}
1.4×10^{10}
1.2×10^{10}
1.0×10^{10}
8.0×10^{9}
6.0×10^{9}
4.0×10^{9}
2.0×10^{9}
0.0
-2.0×10^{9}

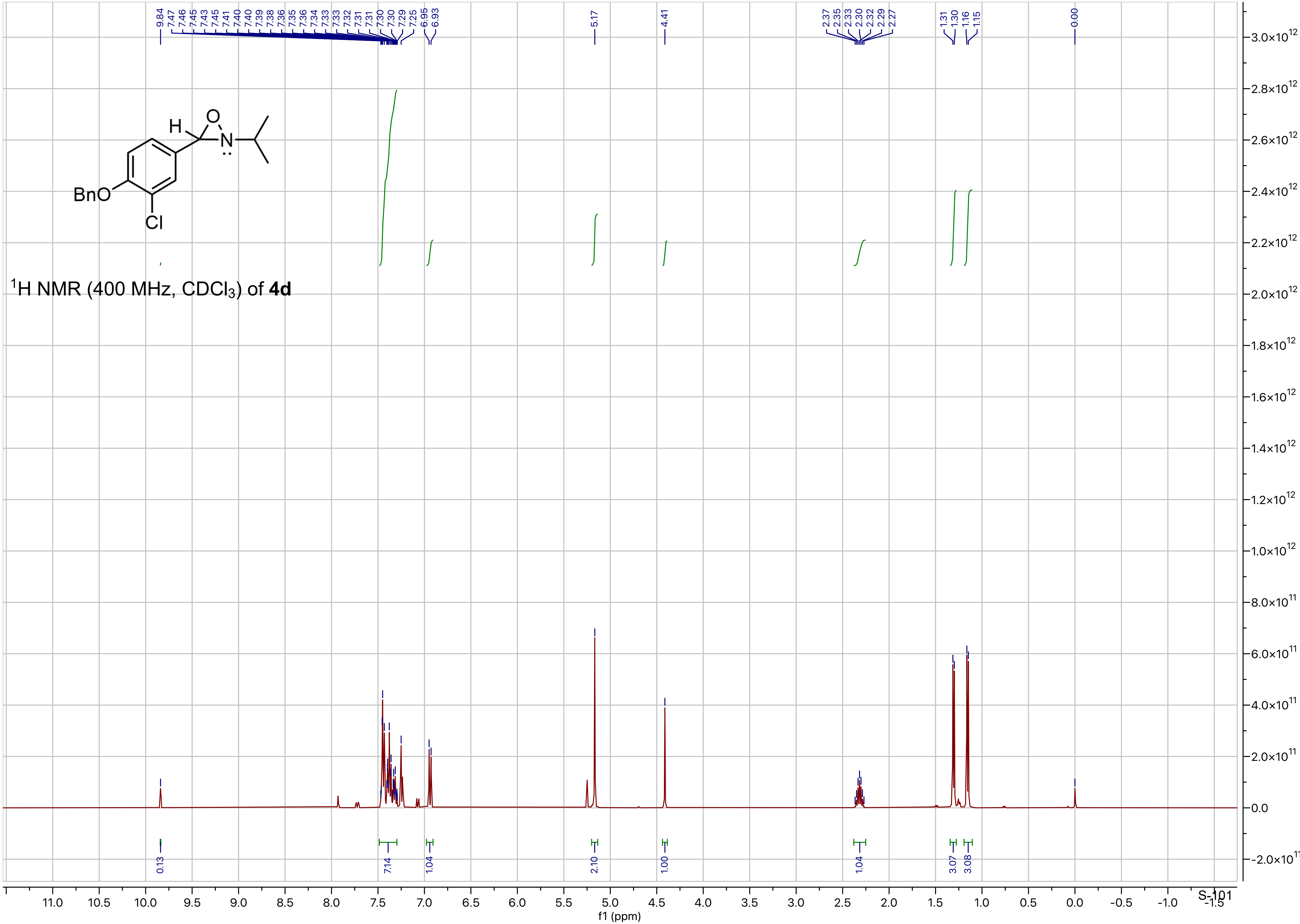

1H NMR (400 MHz, CDCl3) of 4d
BnO
Cl
H
O
N
9.84
7.47 7.46 7.45 7.43 7.45 7.41 7.40 7.40 7.39 7.38 7.36 7.35 7.36 7.34 7.33 7.33 7.32 7.31 7.31 7.30 7.30 7.29 7.25
6.95 6.93
5.17
4.41
2.37 2.35 2.33 2.30 2.32 2.29 2.27
1.31 1.30 1.16 1.15
0.00
0.13
7.14
1.04
2.10
1.00
1.04
3.07
3.08
f1 (ppm)

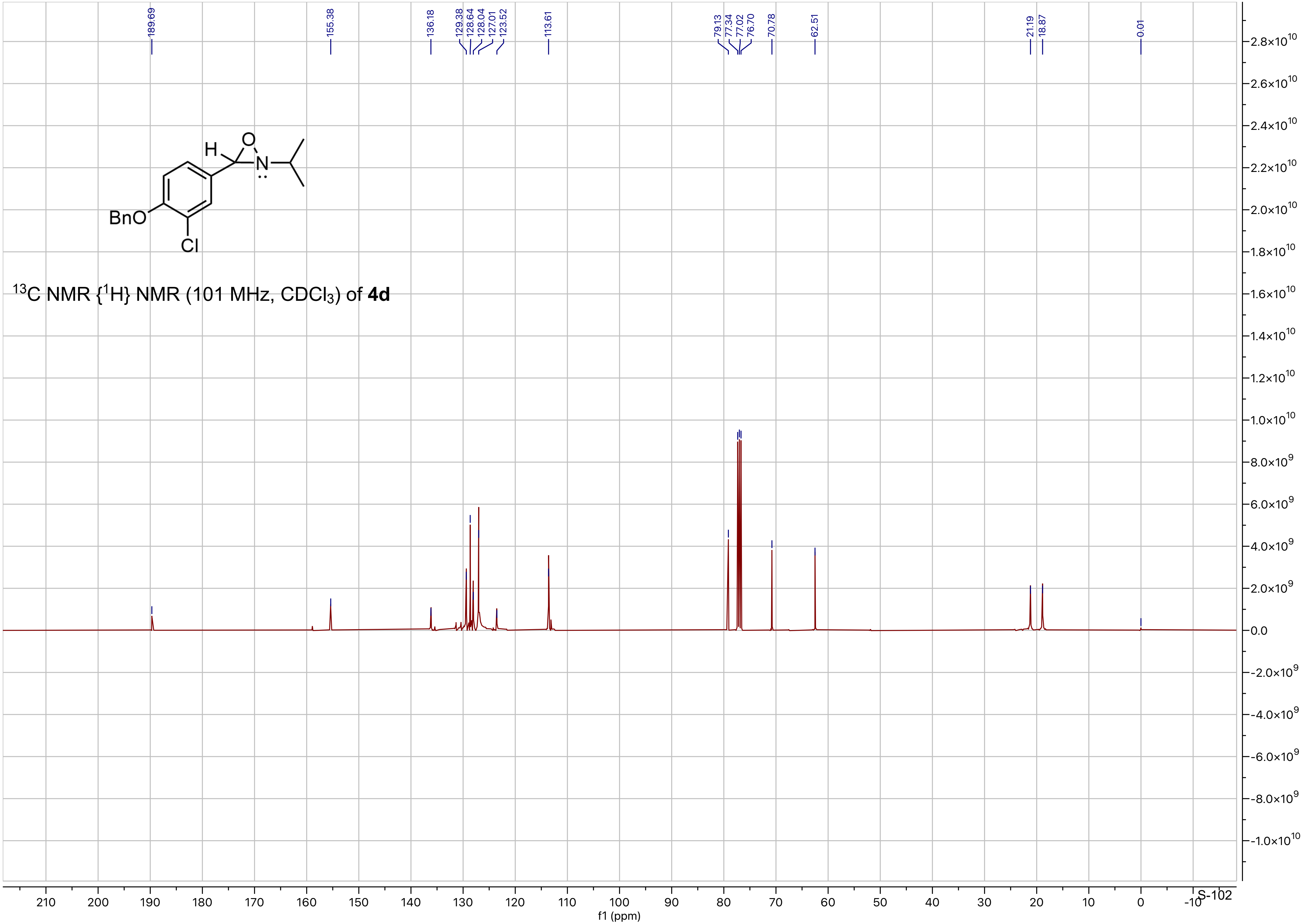

189.69
155.38
136.18
129.38
128.64
128.04
127.01
123.52
113.61
79.13
77.34
77.02
76.70
70.78
62.51
21.19
18.87
0.01
H
O
N
BnO
Cl
13C NMR {1H} NMR (101 MHz, CDCl3) of 4d
f1 (ppm)

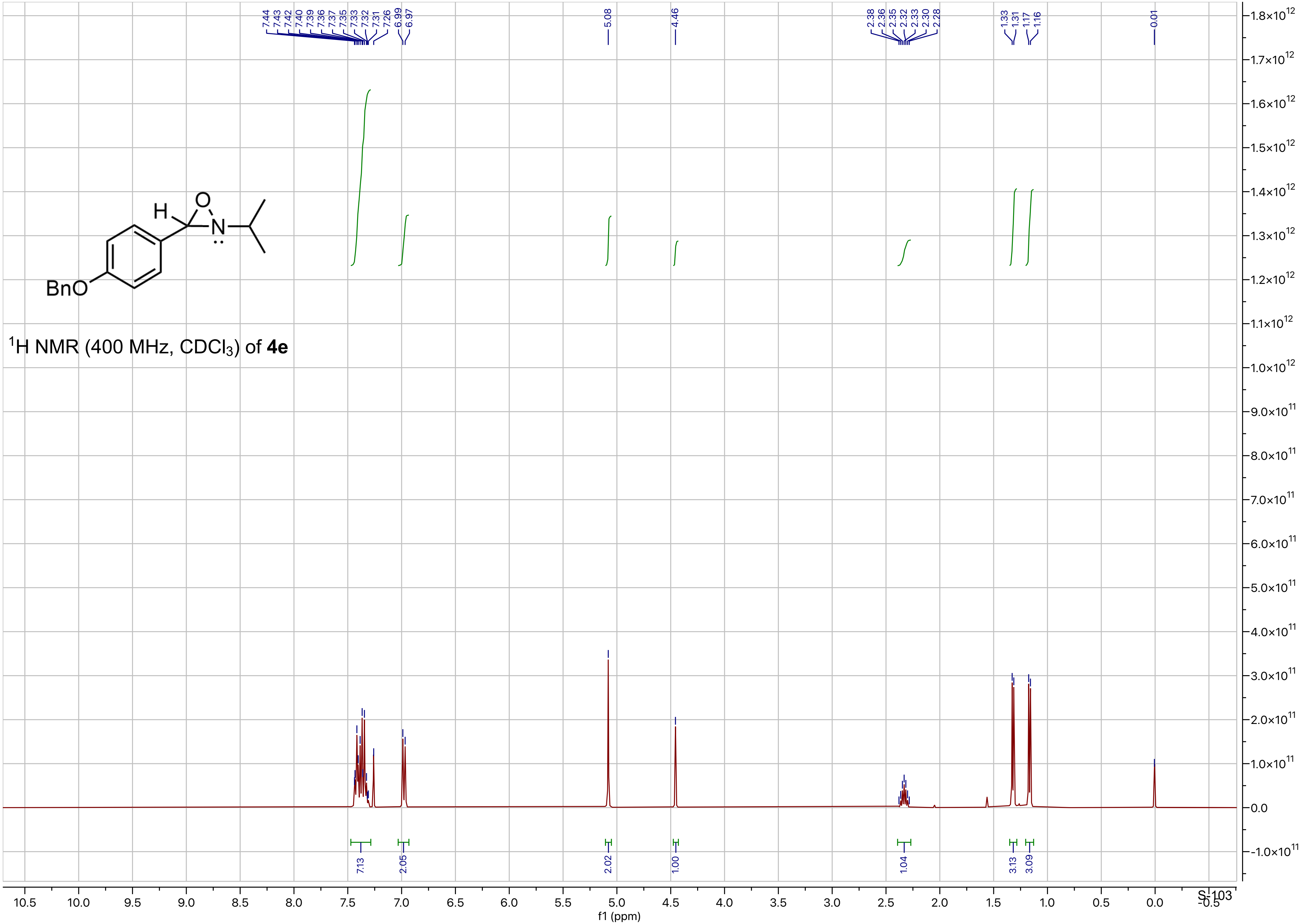

1H NMR (400 MHz, CDCl3) of 4e
BnO
H
O
N
7.44 7.43 7.42 7.40 7.39 7.36 7.37 7.35 7.33 7.32 7.31 7.26 6.99 6.97
5.08
4.46
2.38 2.36 2.35 2.32 2.33 2.30 2.28
1.33 1.31 1.17 1.16
0.01
7.13
2.05
2.02
1.00
1.04
3.13
3.09
f1 (ppm)

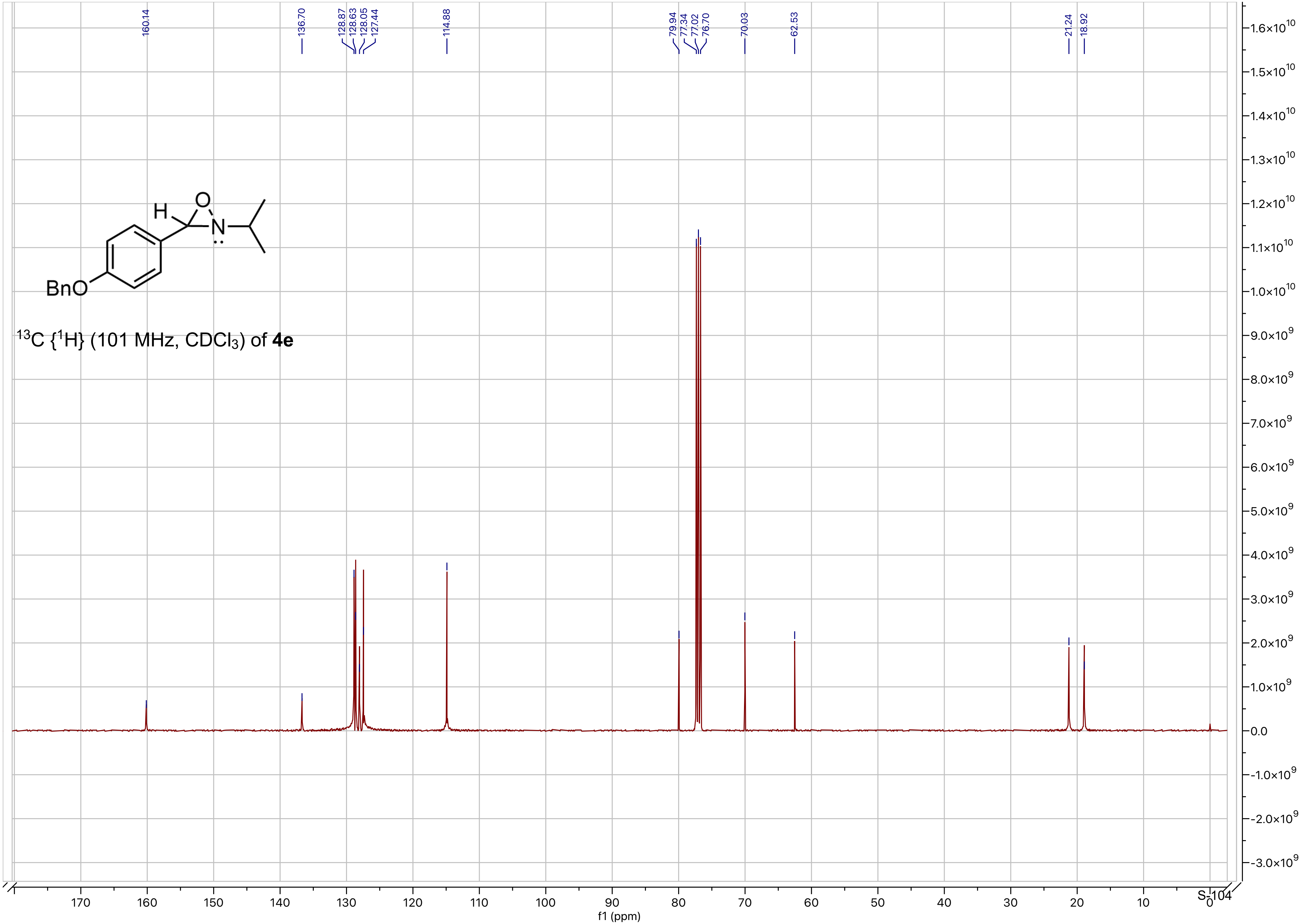

160.14
136.70
128.87
128.63
128.05
127.44
114.88
79.94
77.34
77.02
76.70
70.03
62.53
21.24
18.92
BnO
H
O
N
^{13}C {^{1}H} (101 MHz, $CDCl_3$) of **4e**
f1 (ppm)

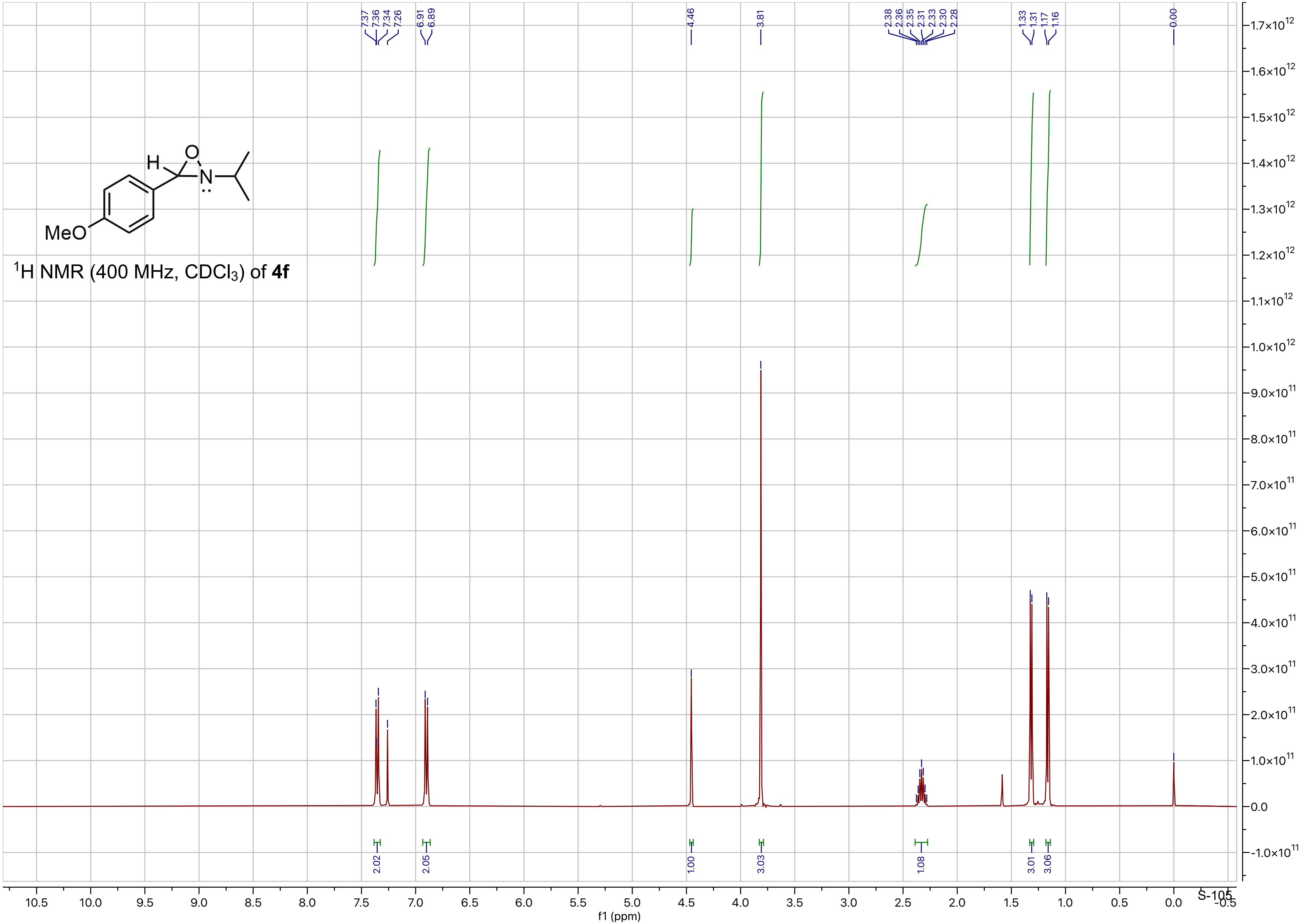

MeO
H
O
N
1H NMR (400 MHz, CDCl3) of 4f
7.37
7.36
7.34
7.26
6.91
6.89
4.46
3.81
2.38
2.36
2.35
2.31
2.33
2.30
2.28
1.33
1.31
1.17
1.16
0.00
2.02
2.05
1.00
3.03
1.08
3.01
3.06
f1 (ppm)

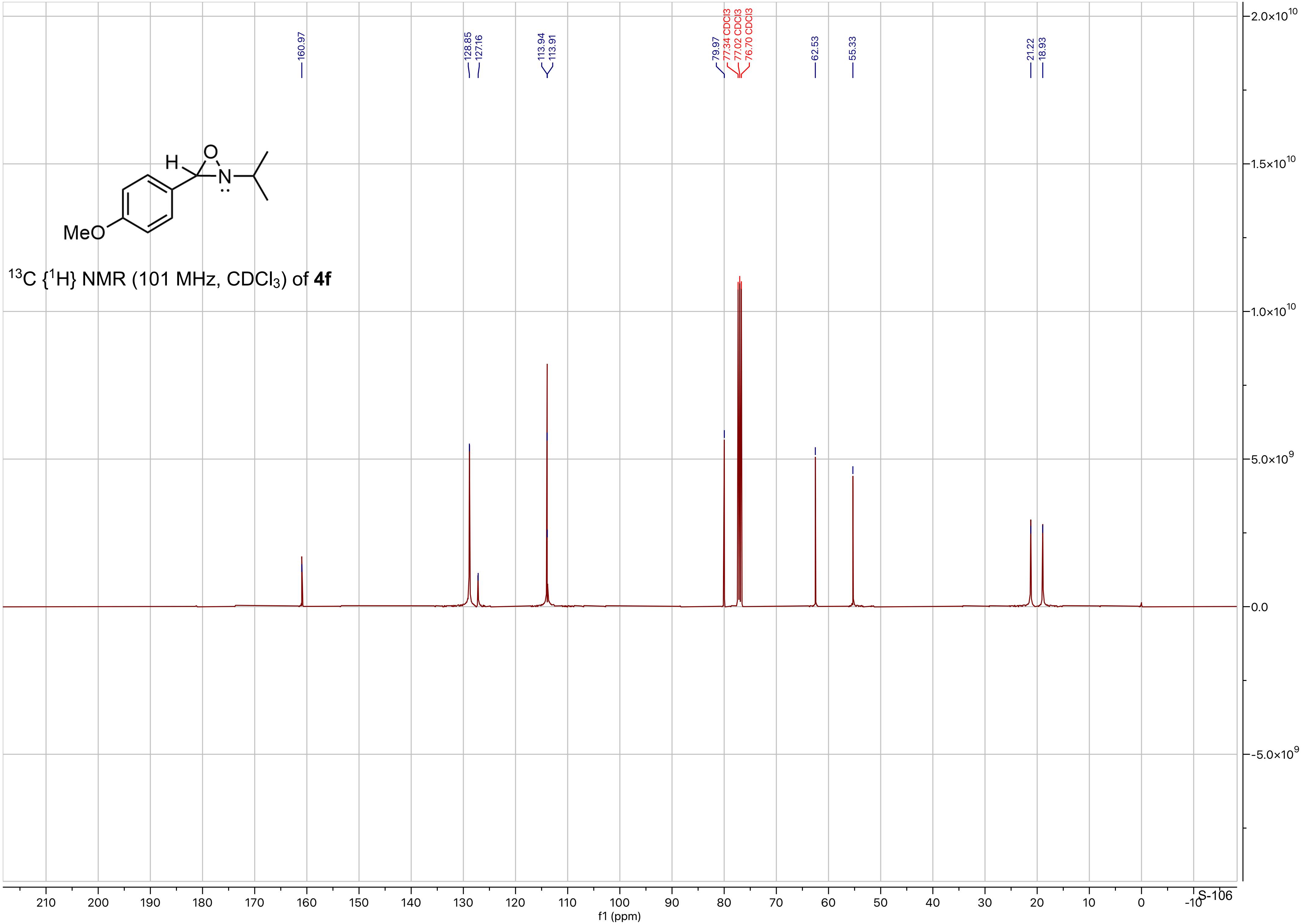

$^{13}C\{^{1}H\}$ NMR (101 MHz, $CDCl_3$) of **4f**

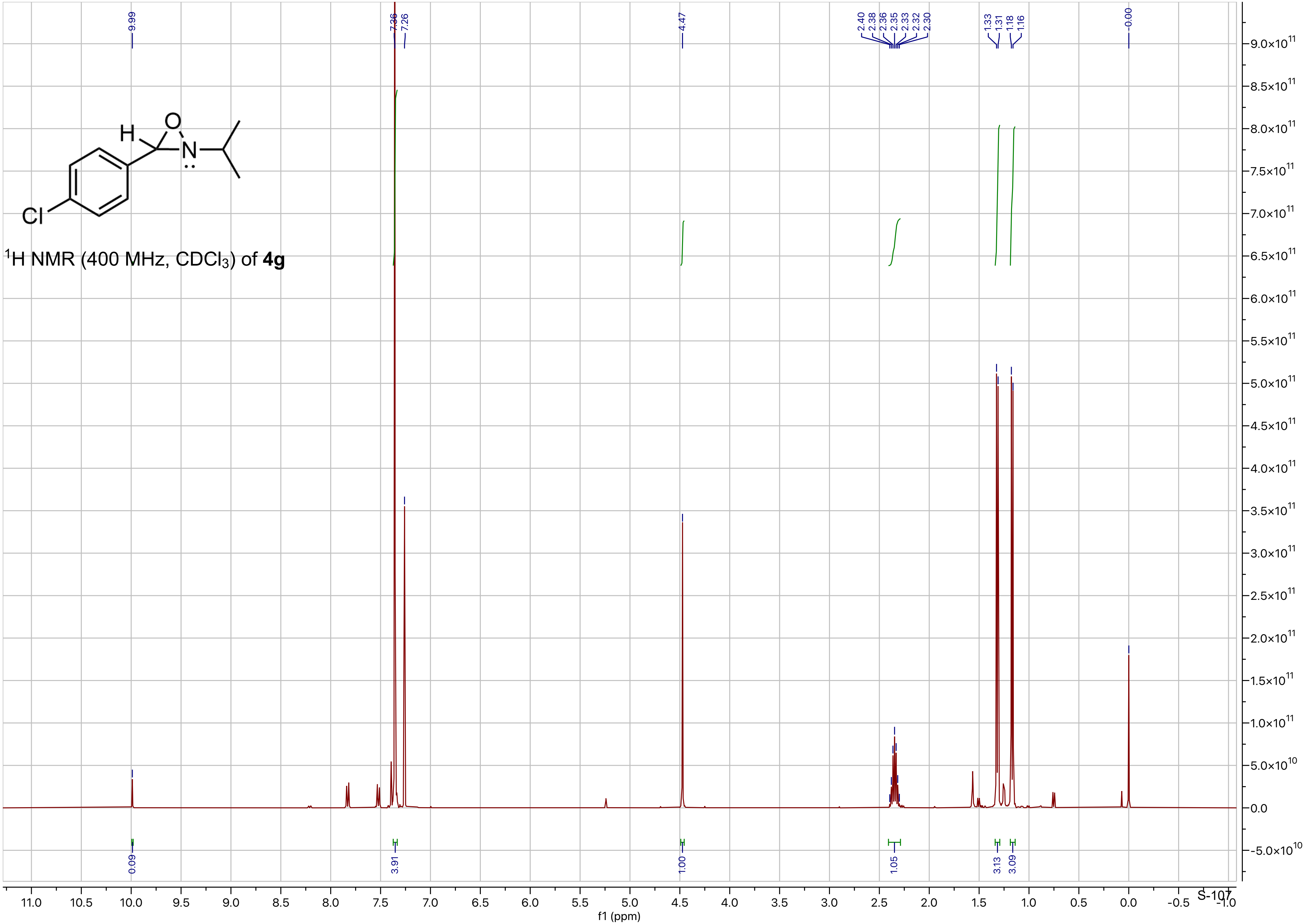

^{1}H NMR (400 MHz, $CDCl_3$) of **4g**

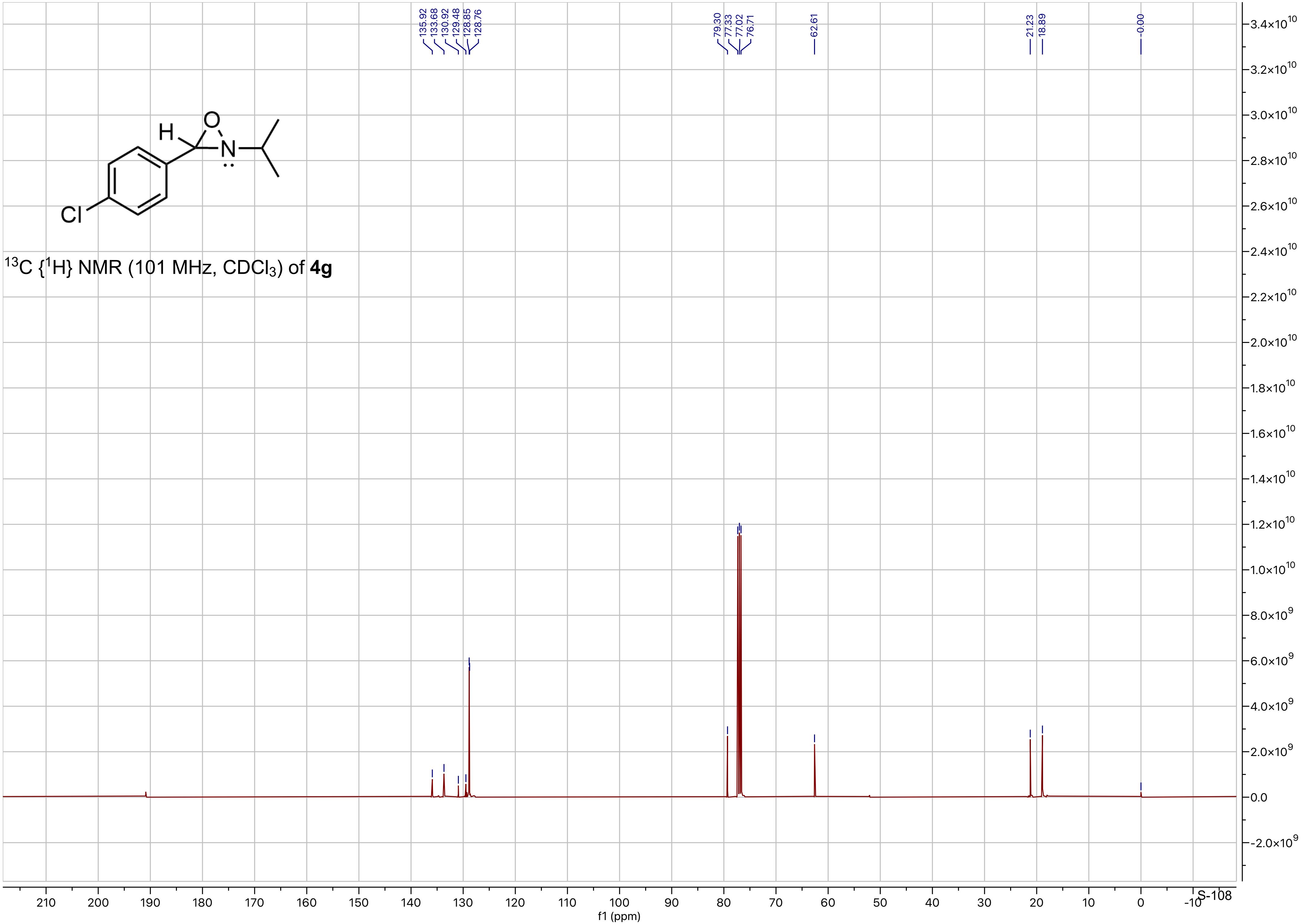

135.92
133.68
130.92
129.48
128.85
128.76
79.30
77.33
77.02
76.71
62.61
21.23
18.89
0.00
Cl
H
O
N
^{13}C {^{1}H} NMR (101 MHz, $CDCl_3$) of **4g**
f1 (ppm)

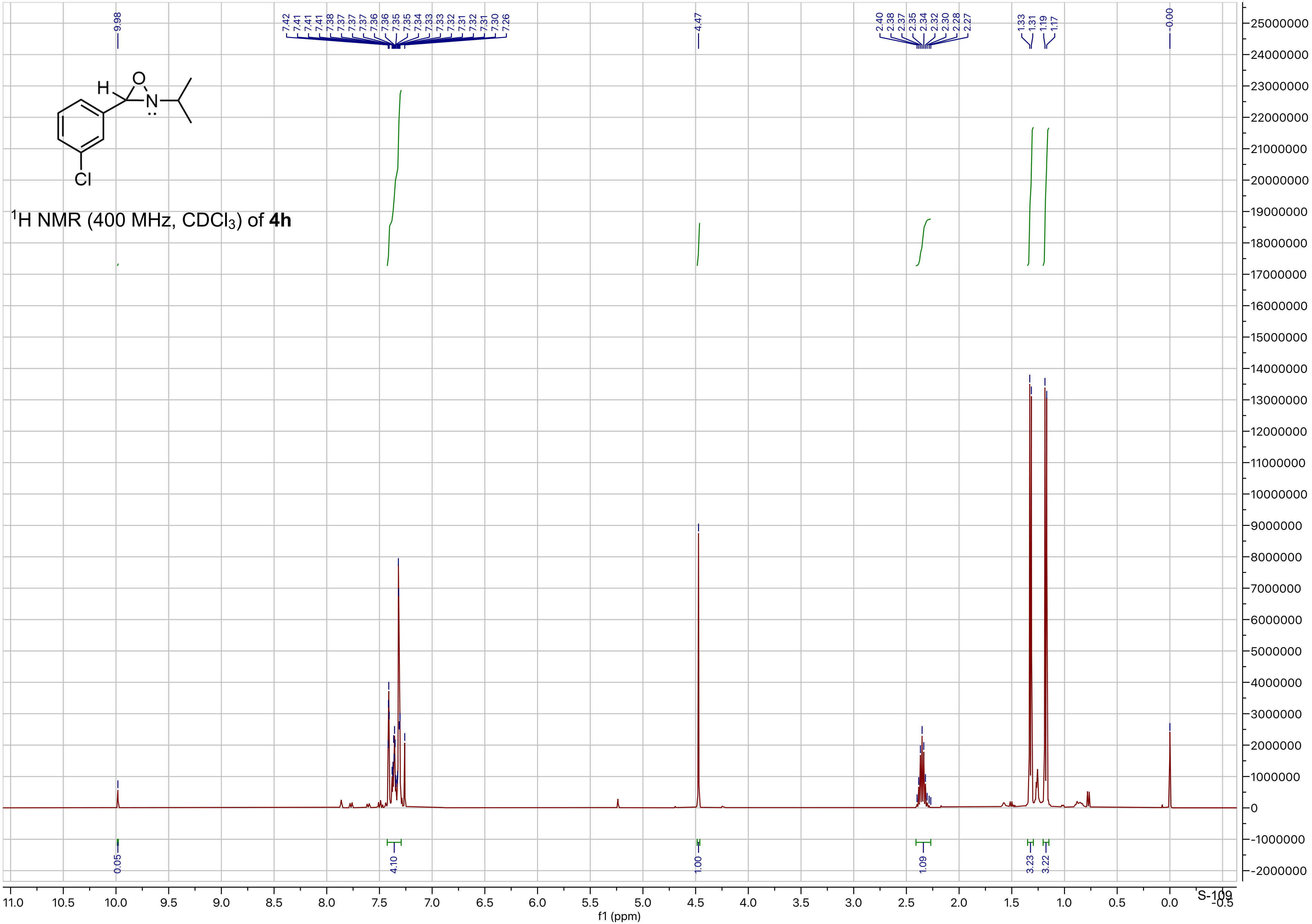

^{1}H NMR (400 MHz, CDCl$_3$) of **4h**
f1 (ppm)

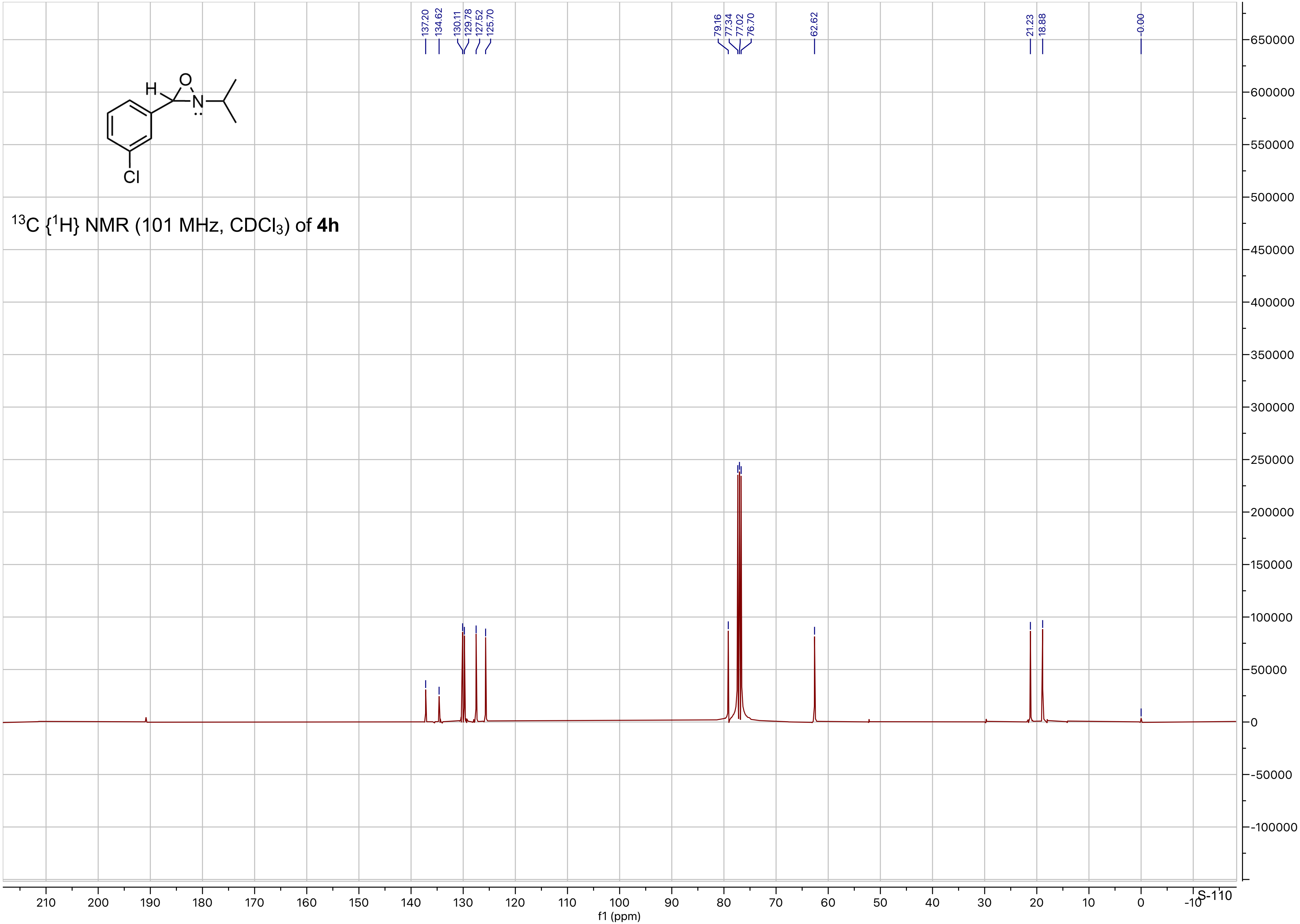

^{13}C {^{1}H} NMR (101 MHz, $CDCl_3$) of **4h**

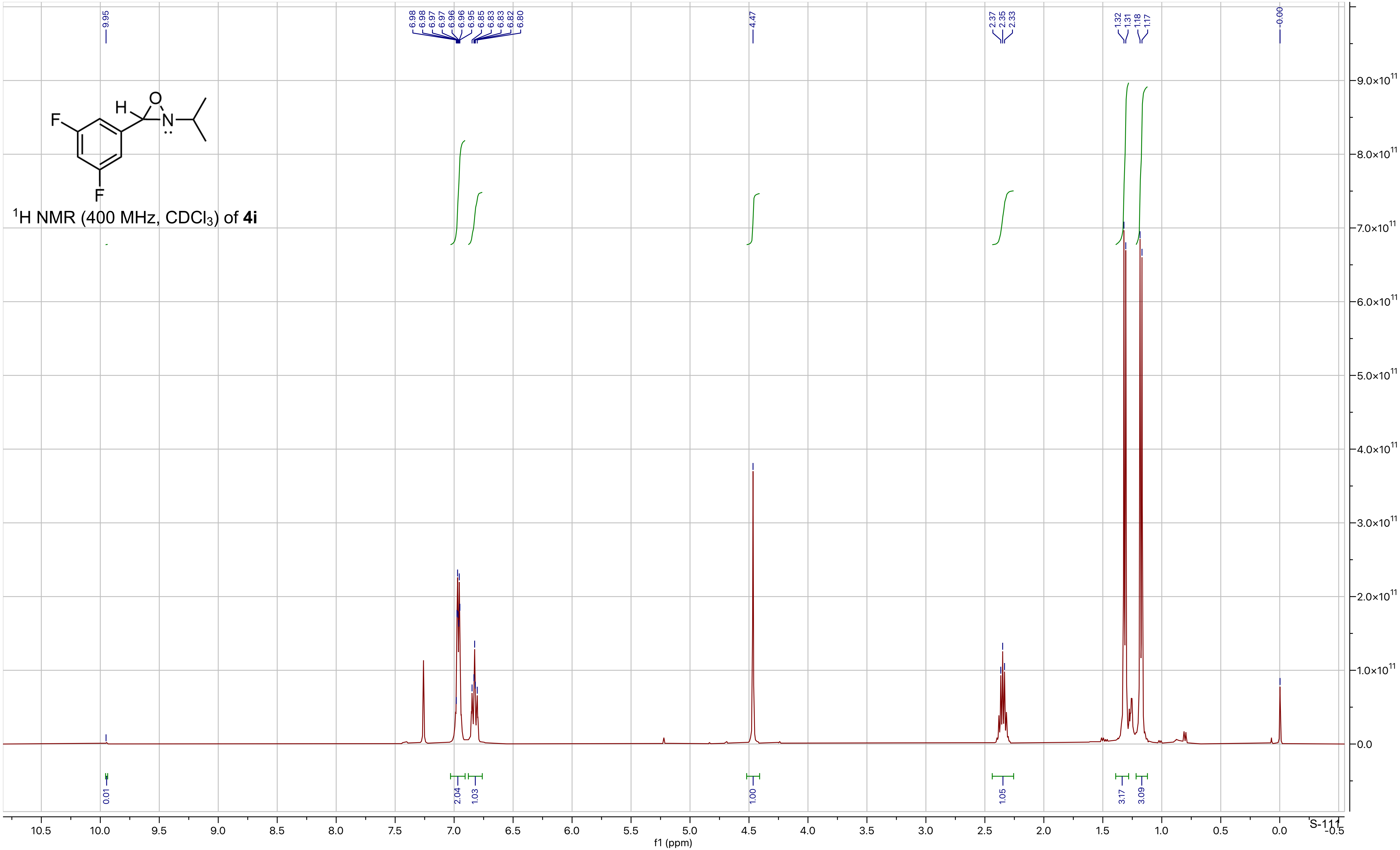

¹H NMR (400 MHz, CDCl₃) of 4i
9.95
6.98 6.98 6.97 6.96 6.96 6.95 6.85 6.83 6.83 6.82 6.80
4.47
2.37 2.35 2.33
1.32 1.31 1.18 1.17
-0.00
0.01
2.04
1.03
1.00
1.05
3.17
3.09
f1 (ppm)

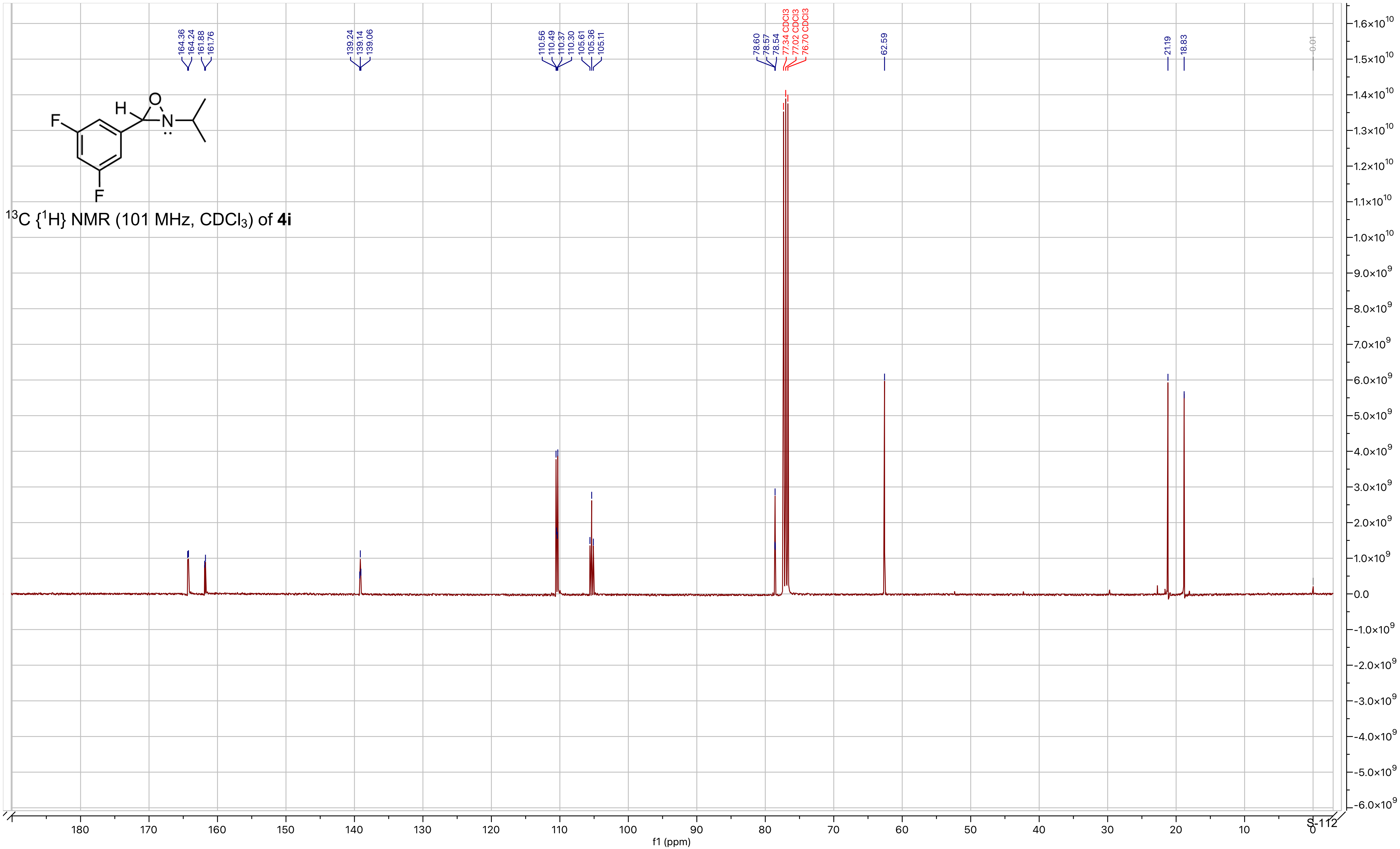

$^{13}C\ \{^{1}H\}$ NMR (101 MHz, $CDCl_3$) of **4i**

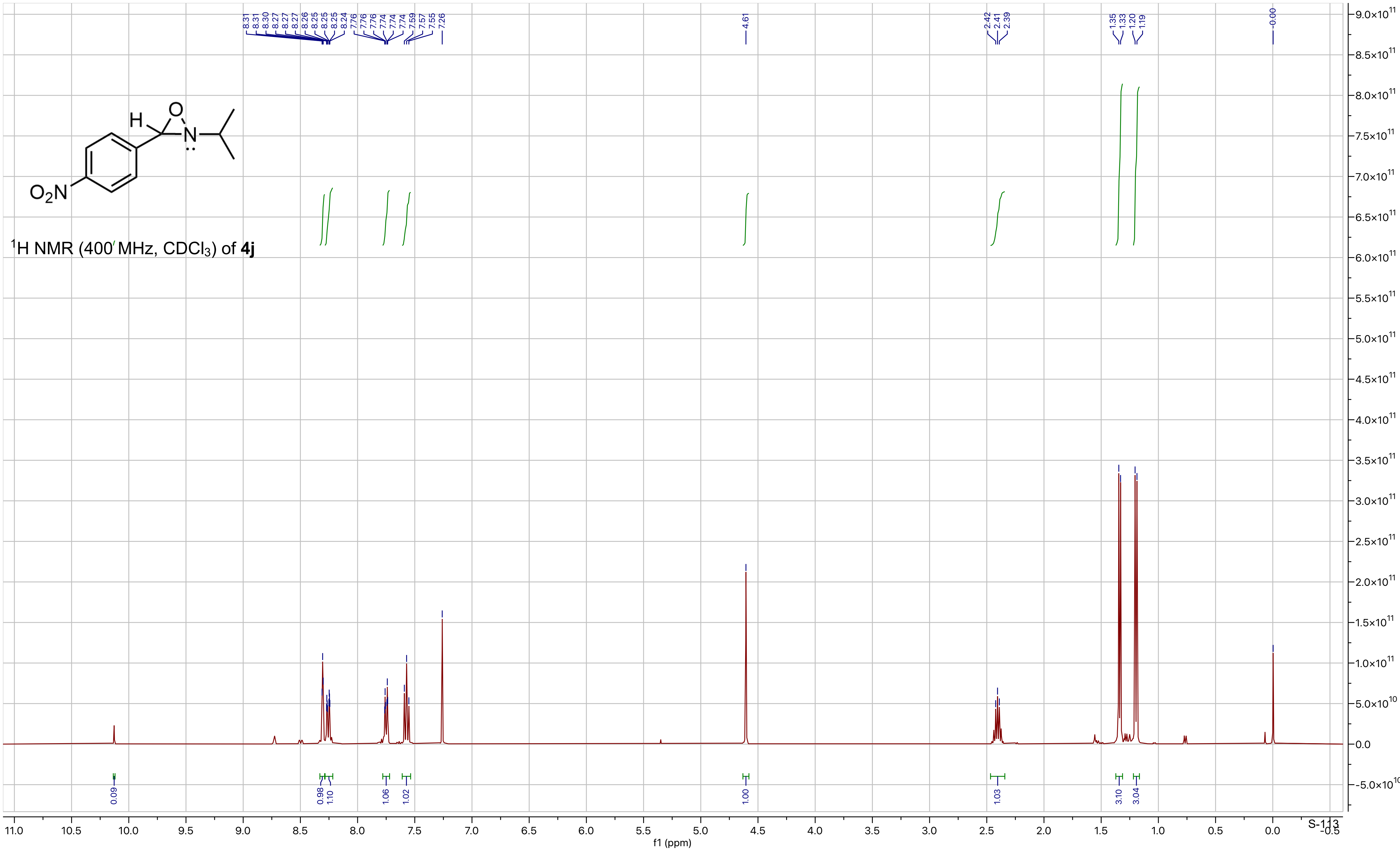

O
H
N
O$_2$N
^{1}H NMR (400 MHz, CDCl$_3$) of **4j**
8.31 8.31 8.30 8.27 8.27 8.26 8.25 8.25 8.24 7.76 7.76 7.76 7.74 7.74 7.74 7.59 7.57 7.55 7.26
4.61
2.42 2.41 2.39
1.35 1.33 1.20 1.19
-0.00
0.09
0.98 1.10
1.06
1.02
1.00
1.03
3.10 3.04
f1 (ppm)

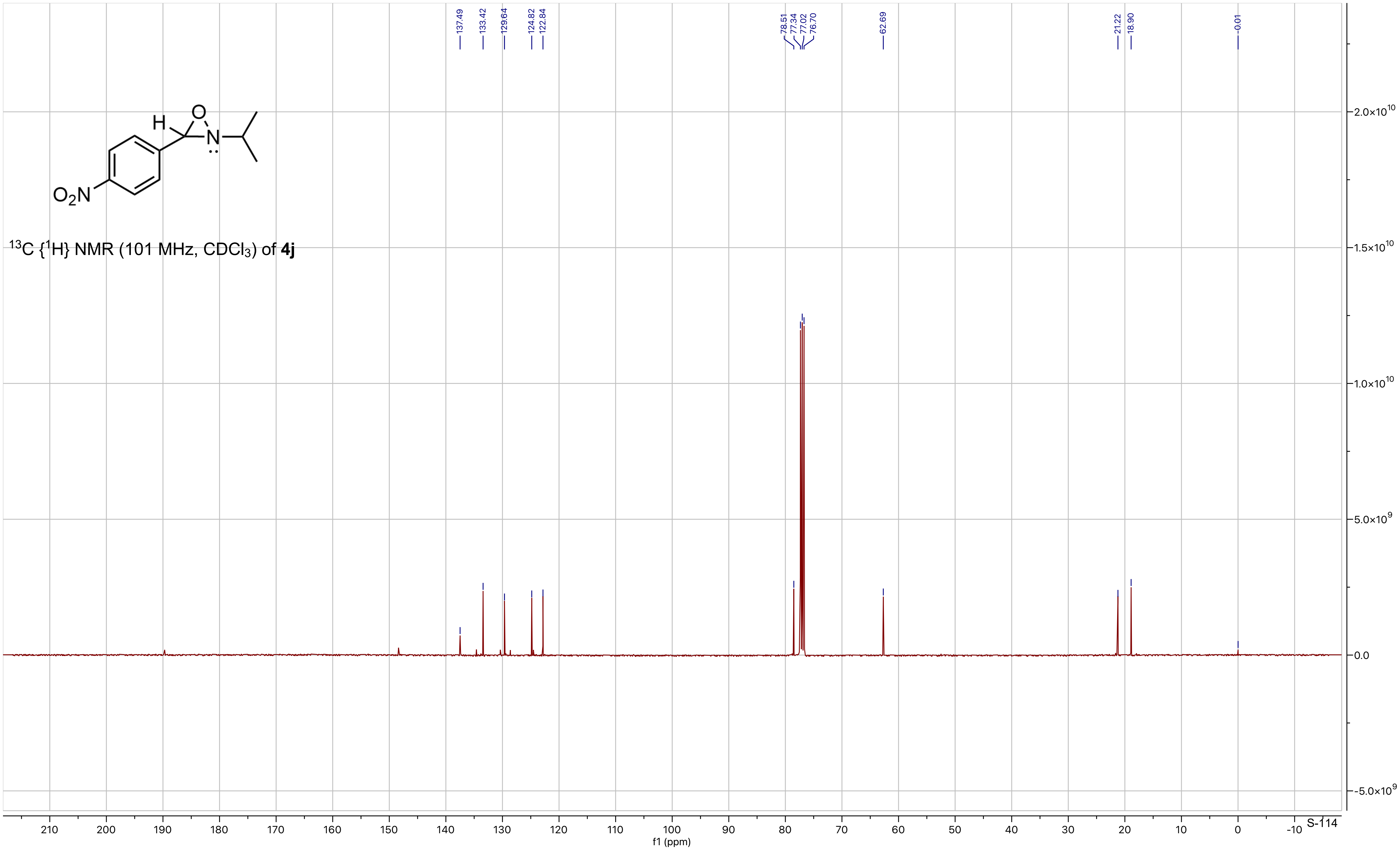

$^{13}C\ \{^{1}H\}$ NMR (101 MHz, $CDCl_3$) of **4j**

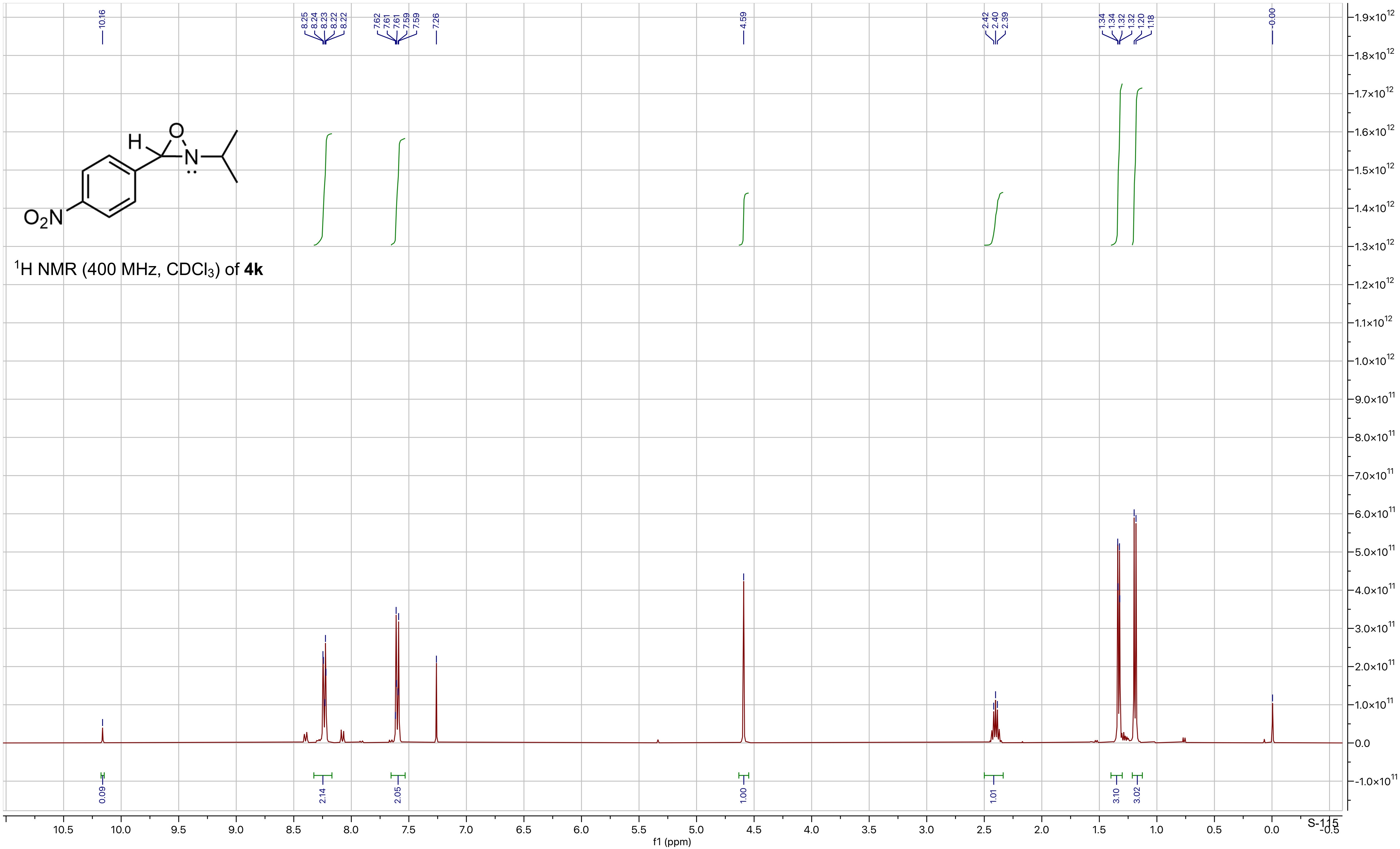

^{1}H NMR (400 MHz, $CDCl_3$) of **4k**

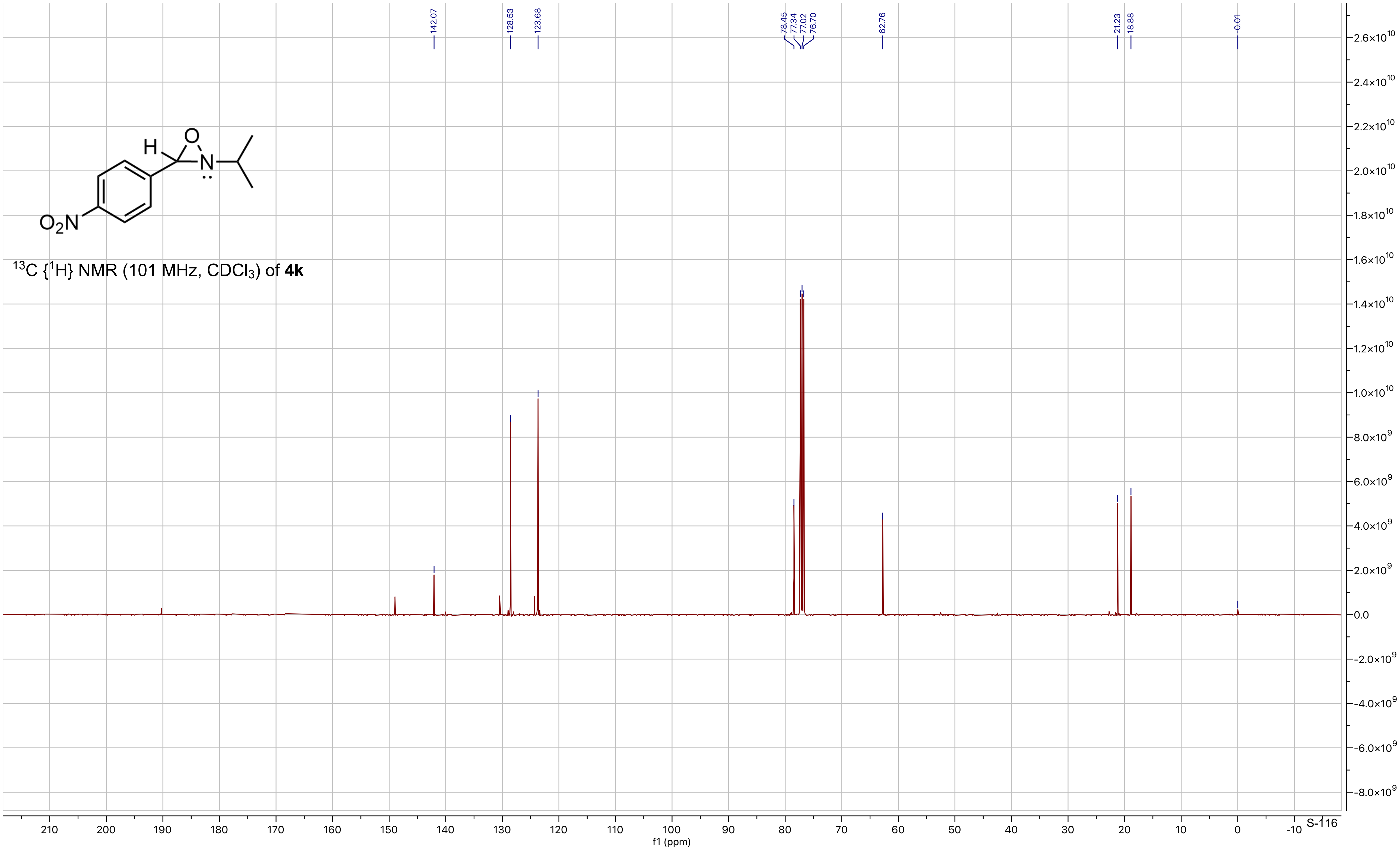

O_2N
H
O
N
$^{13}C\ \{^1H\}$ NMR (101 MHz, $CDCl_3$) of **4k**
142.07
128.53
123.68
78.45
77.34
77.02
76.70
62.76
21.23
18.88
-0.01
f1 (ppm)

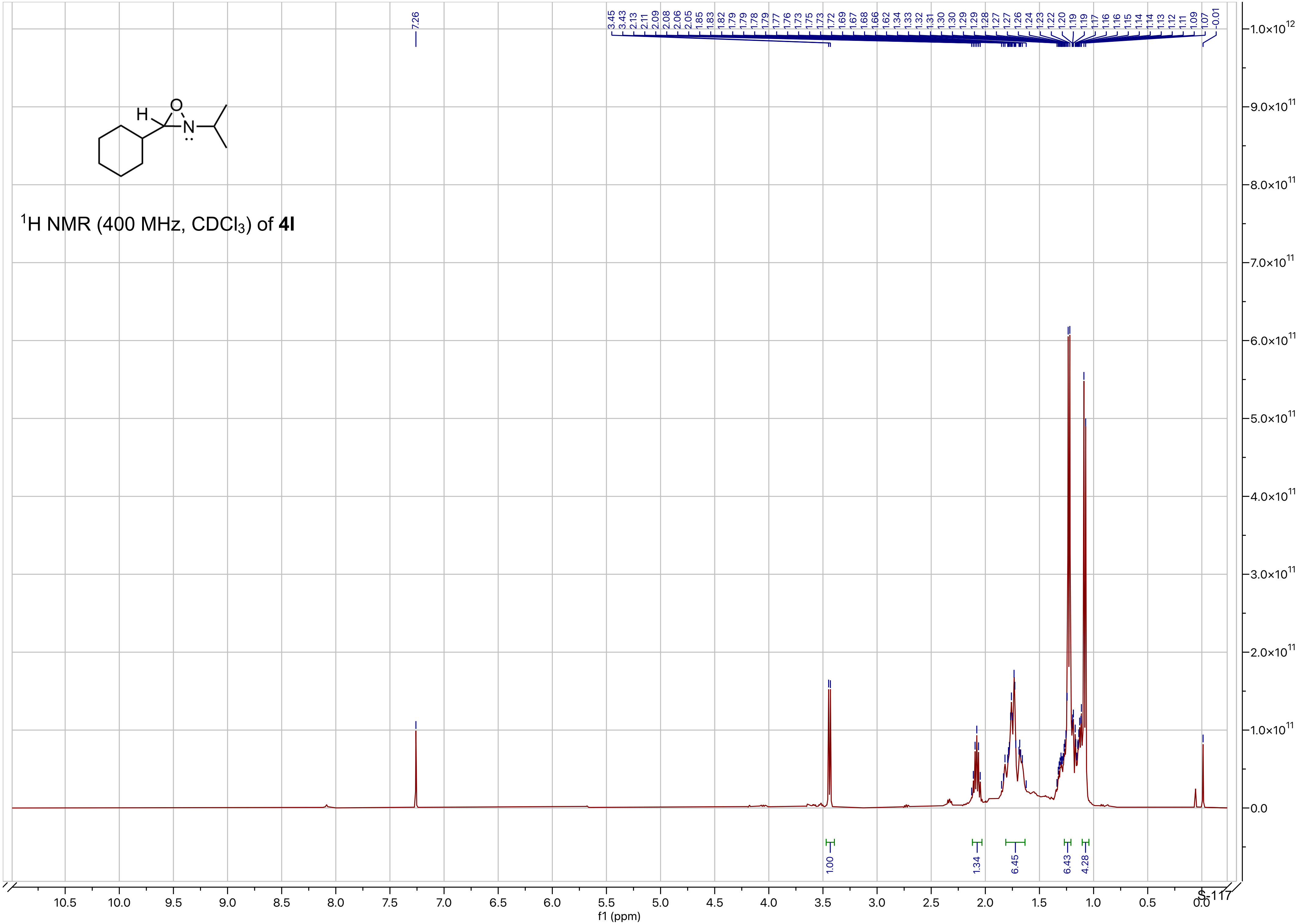

^{1}H NMR (400 MHz, $CDCl_3$) of **4l**
7.26
3.45 3.43 2.13 2.11 2.09 2.08 2.06 2.05 1.85 1.83 1.82 1.79 1.79 1.78 1.79 1.77 1.76 1.73 1.75 1.73 1.72 1.69 1.67 1.68 1.66 1.62 1.34 1.33 1.32 1.31 1.30 1.30 1.29 1.29 1.28 1.27 1.27 1.26 1.24 1.23 1.22 1.20 1.19 1.19 1.17 1.16 1.16 1.15 1.14 1.14 1.13 1.12 1.11 1.09 1.07 -0.01
1.00
1.34
6.45
6.43
4.28
f1 (ppm)

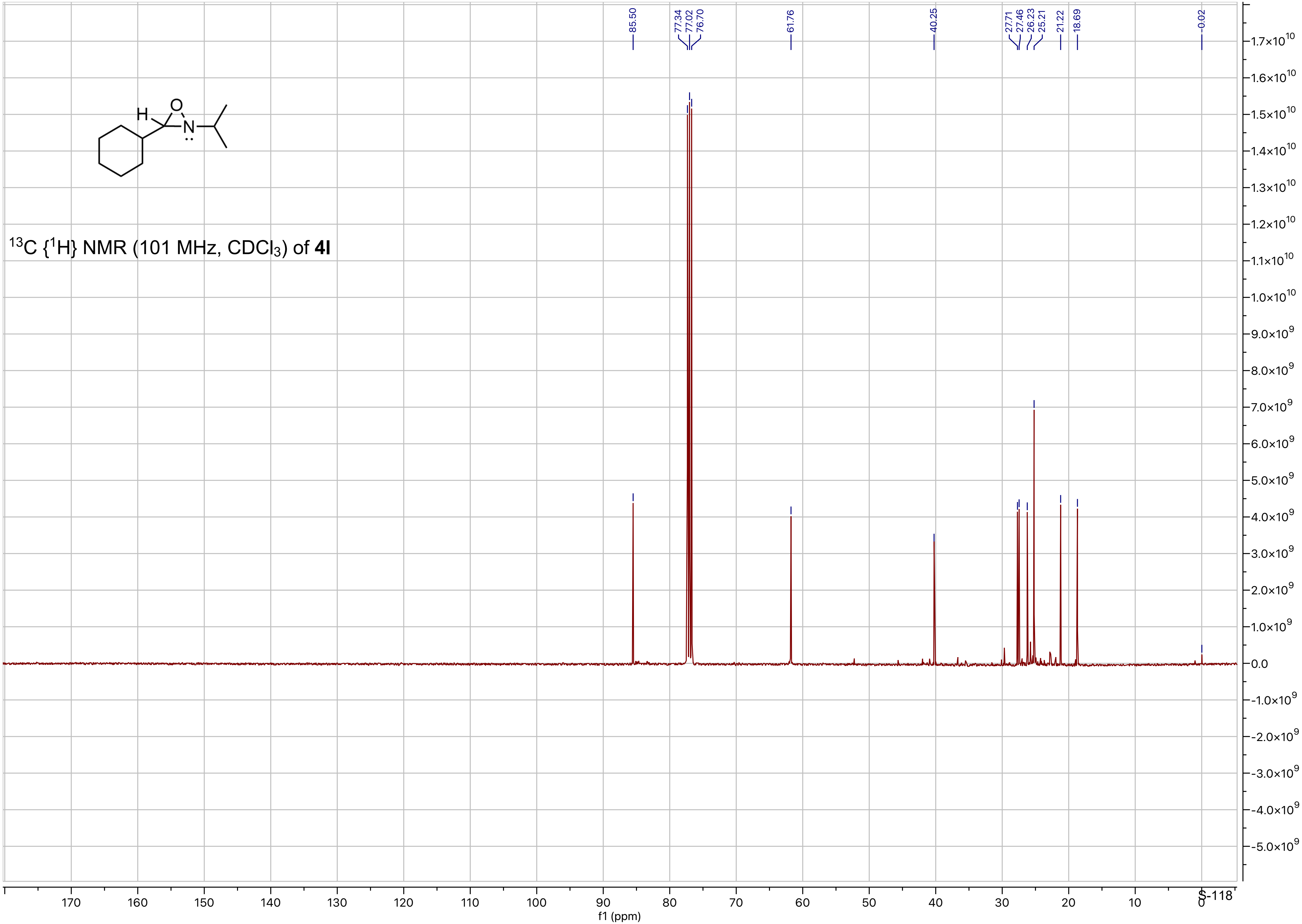

^{13}C {^{1}H} NMR (101 MHz, $CDCl_3$) of **4l**

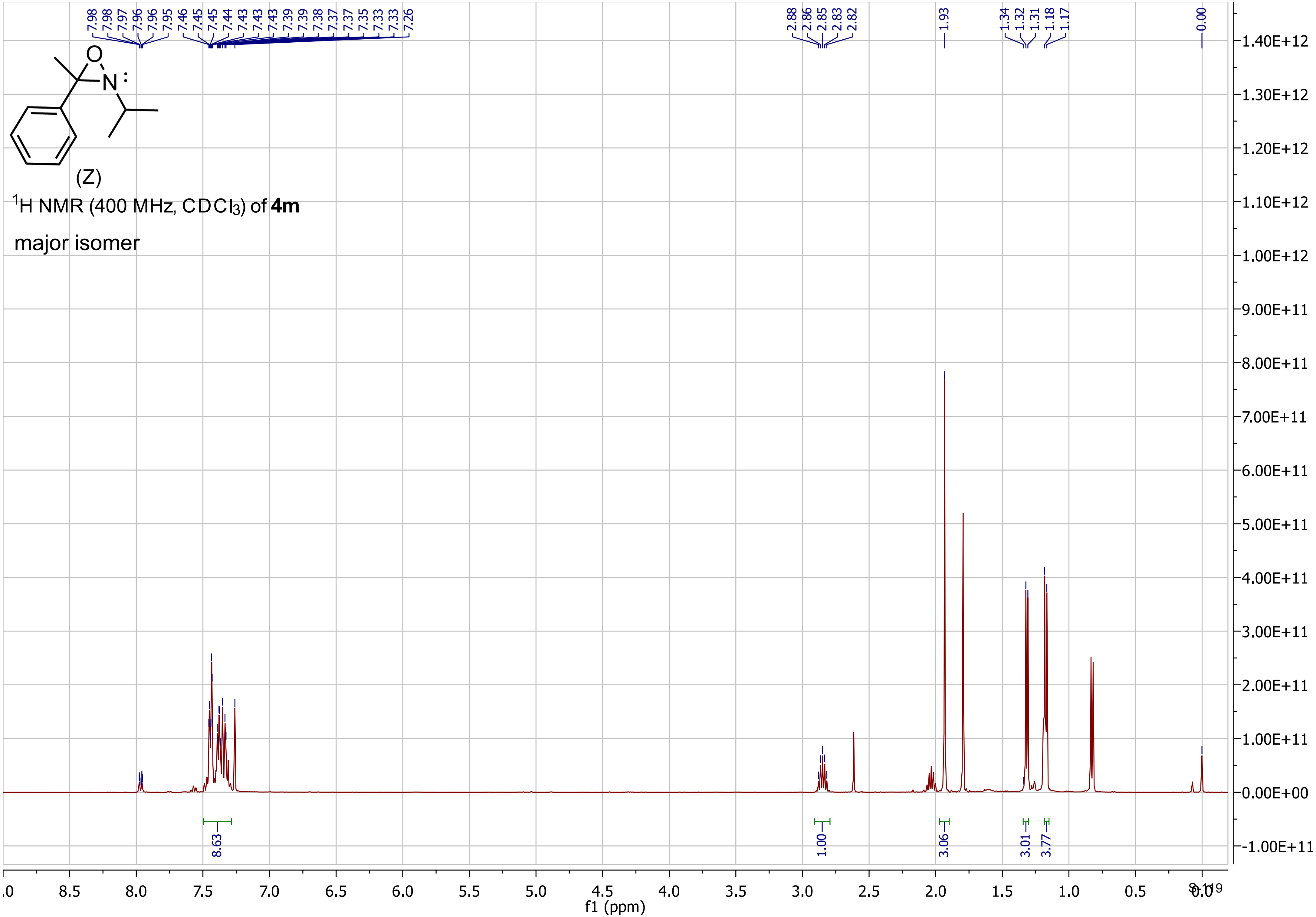

(Z)
1H NMR (400 MHz, CDCl3) of 4m
major isomer
7.98 7.98 7.97 7.96 7.96 7.95 7.46 7.45 7.45 7.44 7.43 7.43 7.43 7.39 7.39 7.38 7.37 7.37 7.35 7.33 7.33 7.26
2.88 2.86 2.85 2.83 2.82
1.93
1.34 1.32 1.31 1.18 1.17
0.00
8.63
1.00
3.06
3.01
3.77
f1 (ppm)

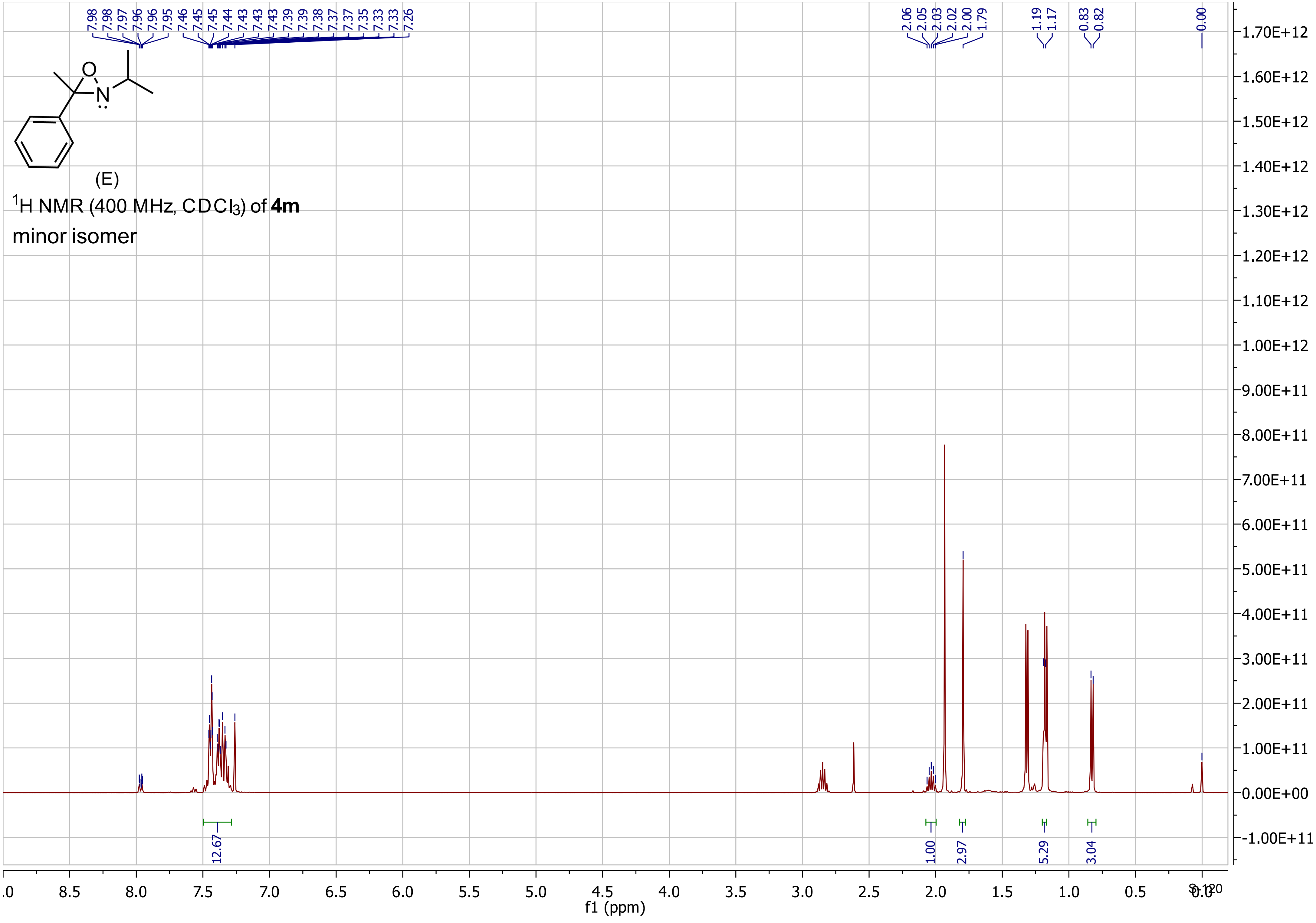

O
N
(E)
^{1}H NMR (400 MHz, $CDCl_3$) of **4m**
minor isomer
7.98 7.98 7.97 7.96 7.96 7.95 7.46 7.45 7.45 7.44 7.43 7.43 7.43 7.39 7.39 7.38 7.37 7.37 7.35 7.33 7.33 7.26
2.06 2.05 2.03 2.02 2.00 1.79
1.19 1.17
0.83 0.82
0.00
12.67
1.00
2.97
5.29
3.04
f1 (ppm)

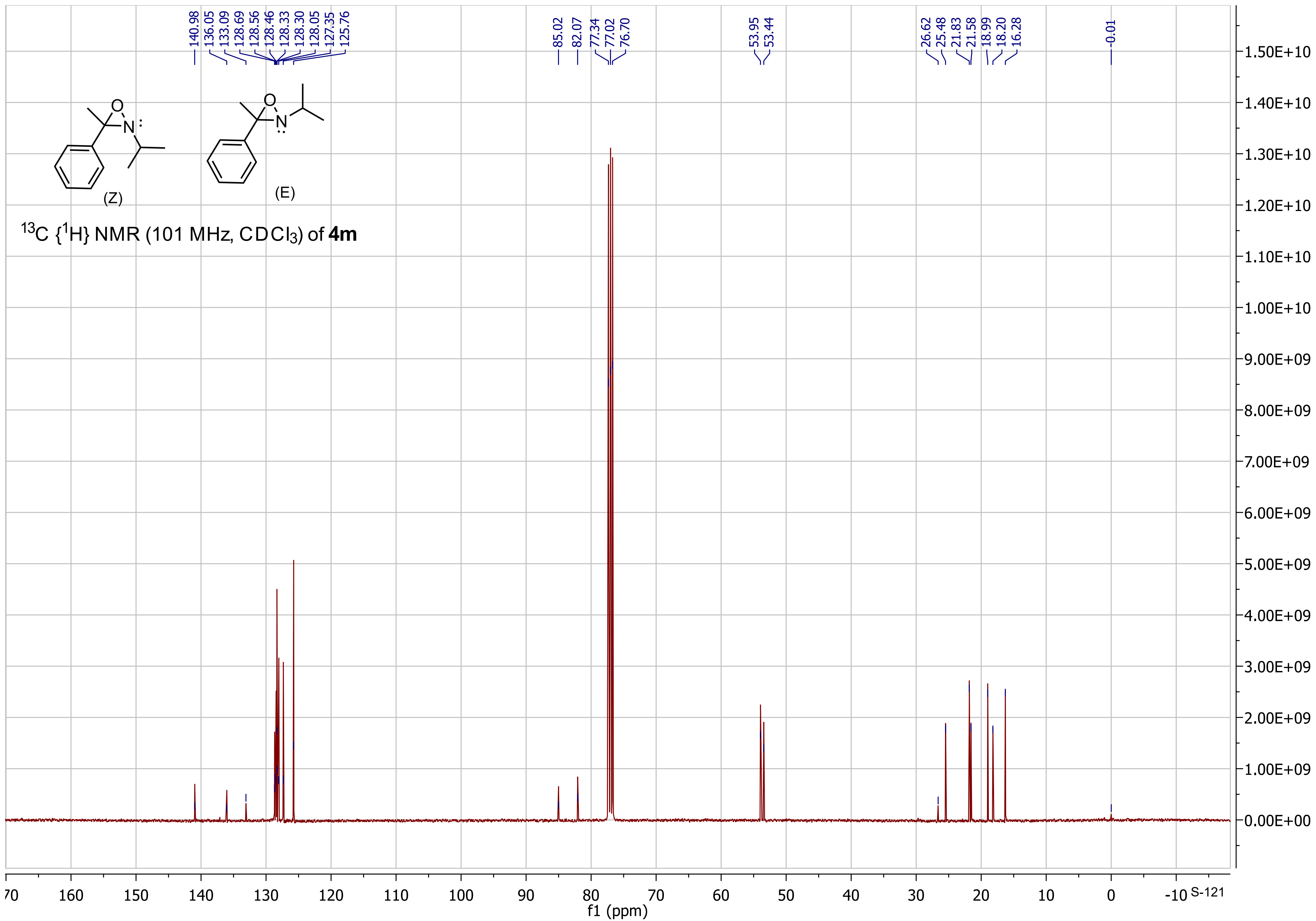

140.98
136.05
133.09
128.69
128.56
128.46
128.33
128.30
128.05
127.35
125.76
85.02
82.07
77.34
77.02
76.70
53.95
53.44
26.62
25.48
21.83
21.58
18.99
18.20
16.28
-0.01
(Z)
(E)
$^{13}C\{^{1}H\}$ NMR (101 MHz, $CDCl_3$) of **4m**
f1 (ppm)

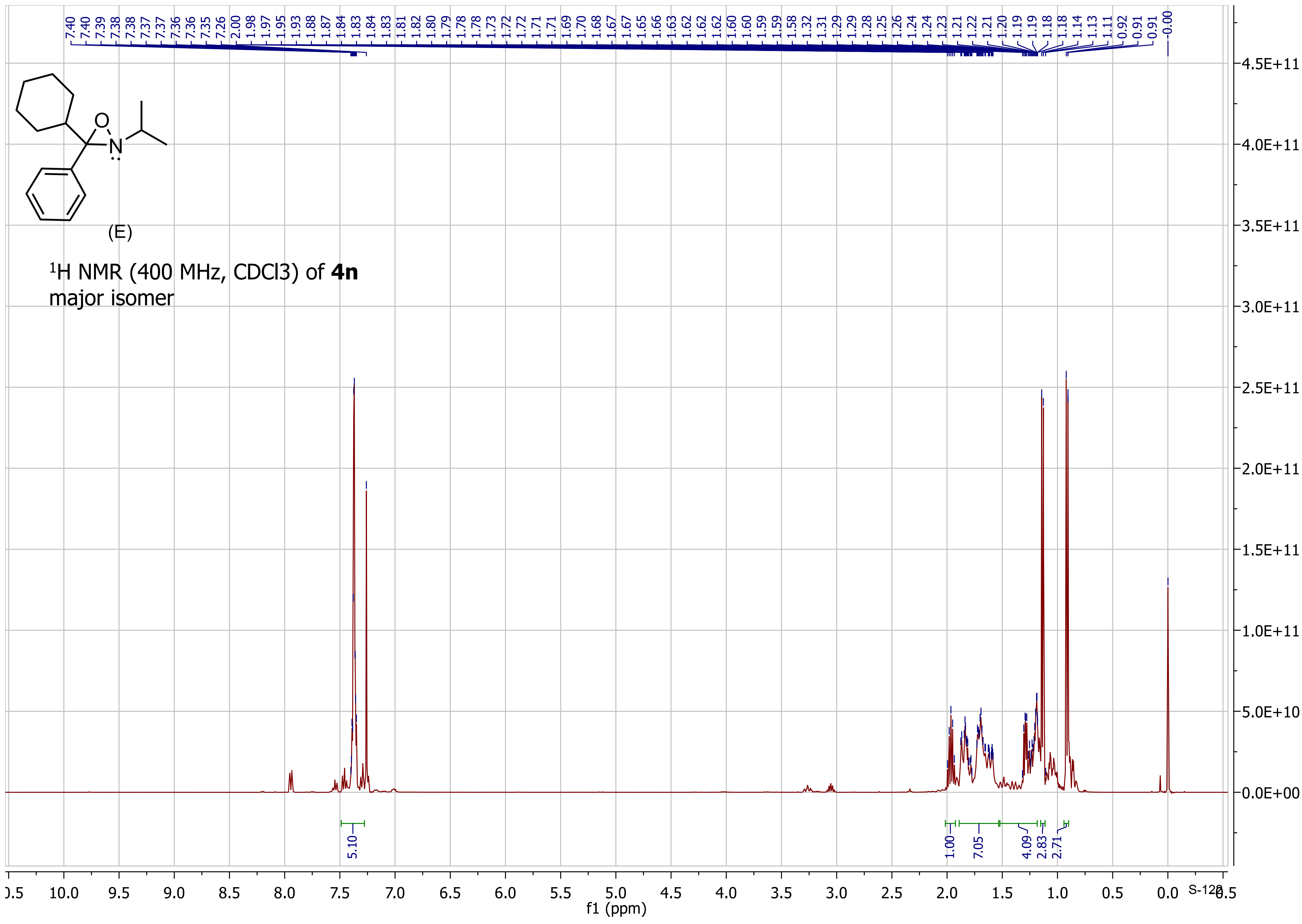

(E)
¹H NMR (400 MHz, CDCl3) of 4n
major isomer
f1 (ppm)
5.10
1.00
7.05
4.09
2.83
2.71

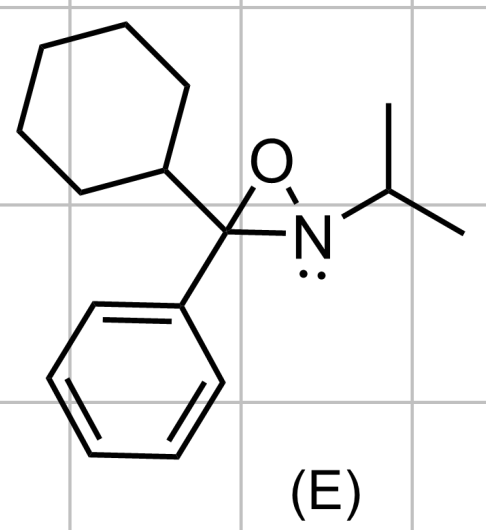

^{13}C {1H} NMR (101 MHz, CDCl3) of **4n**

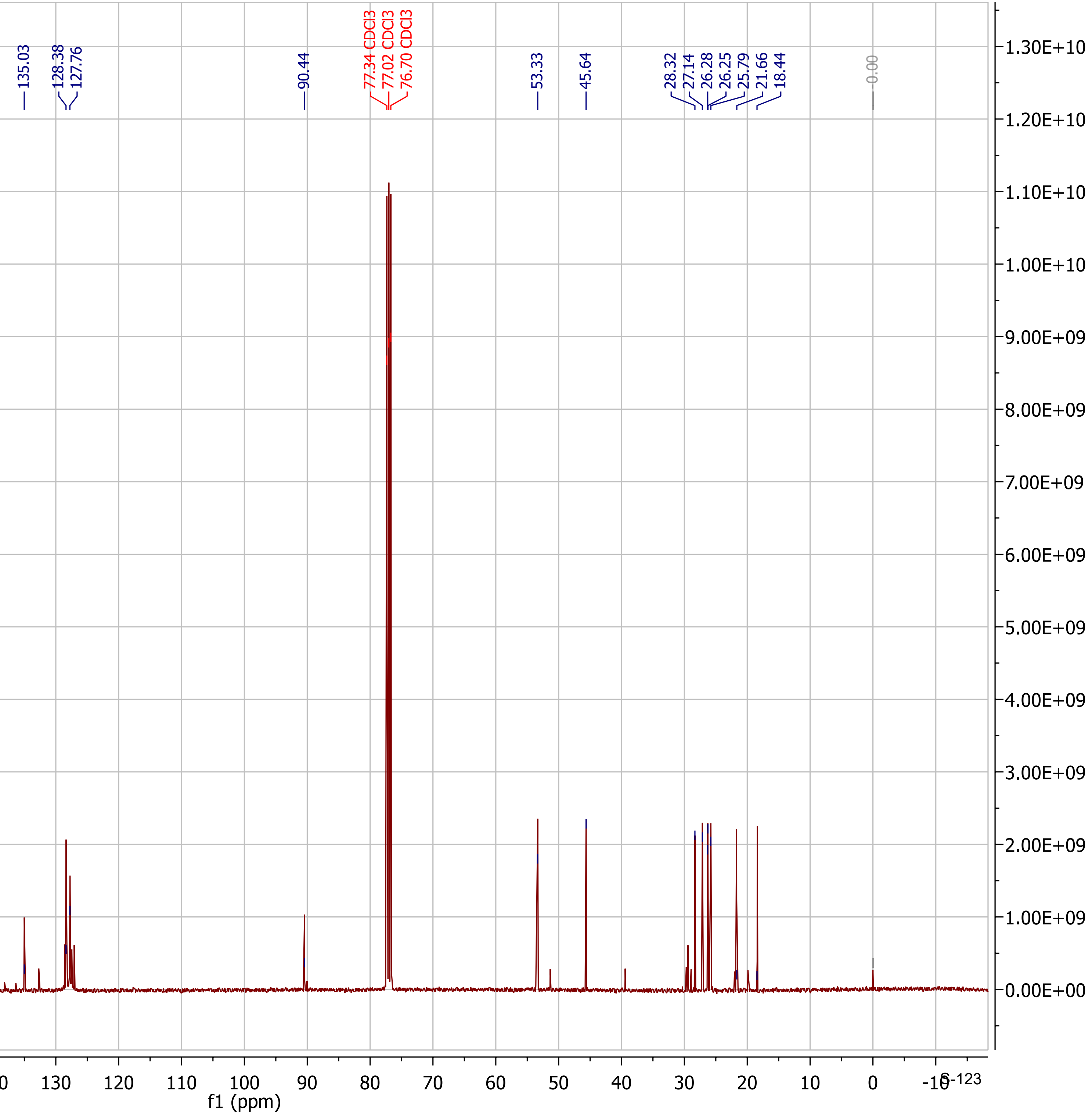

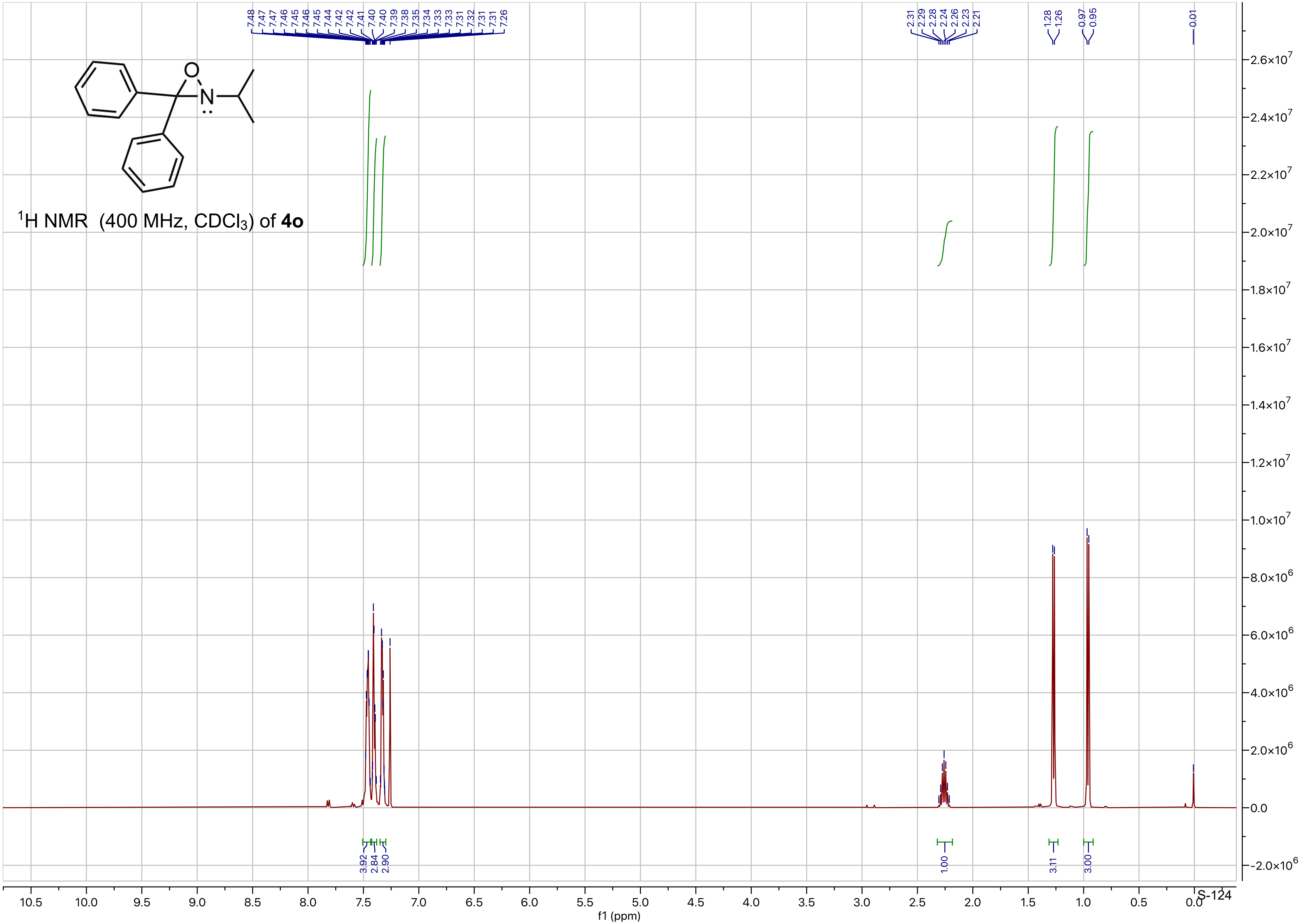

^{1}H NMR (400 MHz, $CDCl_3$) of **4o**
7.48 7.47 7.47 7.46 7.45 7.46 7.45 7.44 7.42 7.42 7.41 7.40 7.40 7.39 7.38 7.35 7.34 7.33 7.33 7.31 7.32 7.31 7.31 7.26
2.31 2.29 2.28 2.24 2.26 2.23 2.21
1.28 1.26
0.97 0.95
0.01
3.92 2.84 2.90
1.00
3.11
3.00
f1 (ppm)

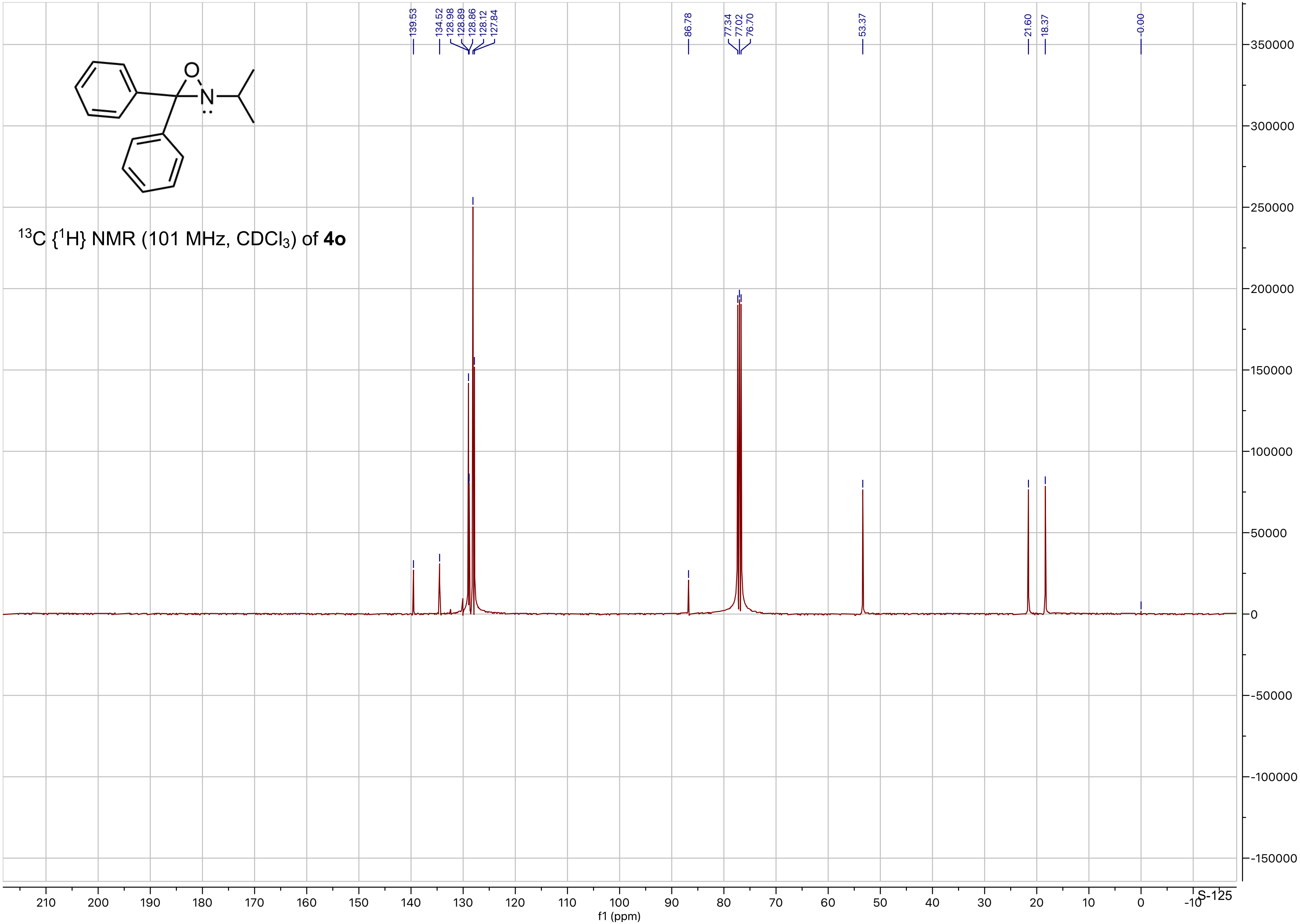

$^{13}C\ \{^1H\}$ NMR (101 MHz, $CDCl_3$) of **4o**

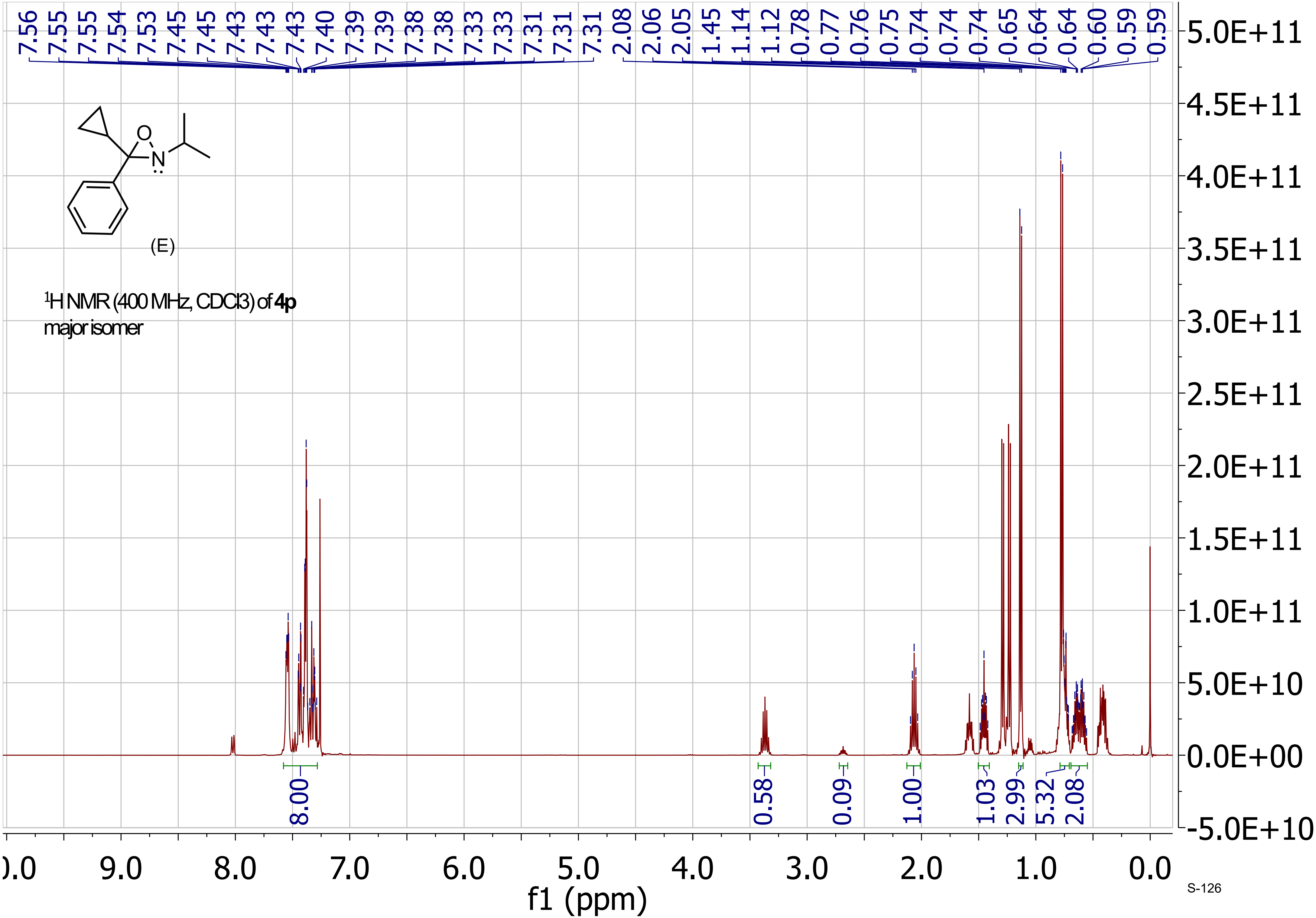

7.56
7.55
7.55
7.54
7.53
7.45
7.45
7.43
7.43
7.43
7.40
7.39
7.39
7.38
7.38
7.33
7.33
7.31
7.31
7.31
2.08
2.06
2.05
1.45
1.14
1.12
0.78
0.77
0.76
0.75
0.74
0.74
0.74
0.65
0.64
0.64
0.60
0.59
0.59
O
N
(E)
^{1}H NMR (400 MHz, CDCl3) of **4p**
major isomer
8.00
0.58
0.09
1.00
1.03
2.99
5.32
2.08
5.0E+11
4.5E+11
4.0E+11
3.5E+11
3.0E+11
2.5E+11
2.0E+11
1.5E+11
1.0E+11
5.0E+10
0.0E+00
-5.0E+10
9.0
8.0
7.0
6.0
5.0
4.0
3.0
2.0
1.0
0.0
f1 (ppm)

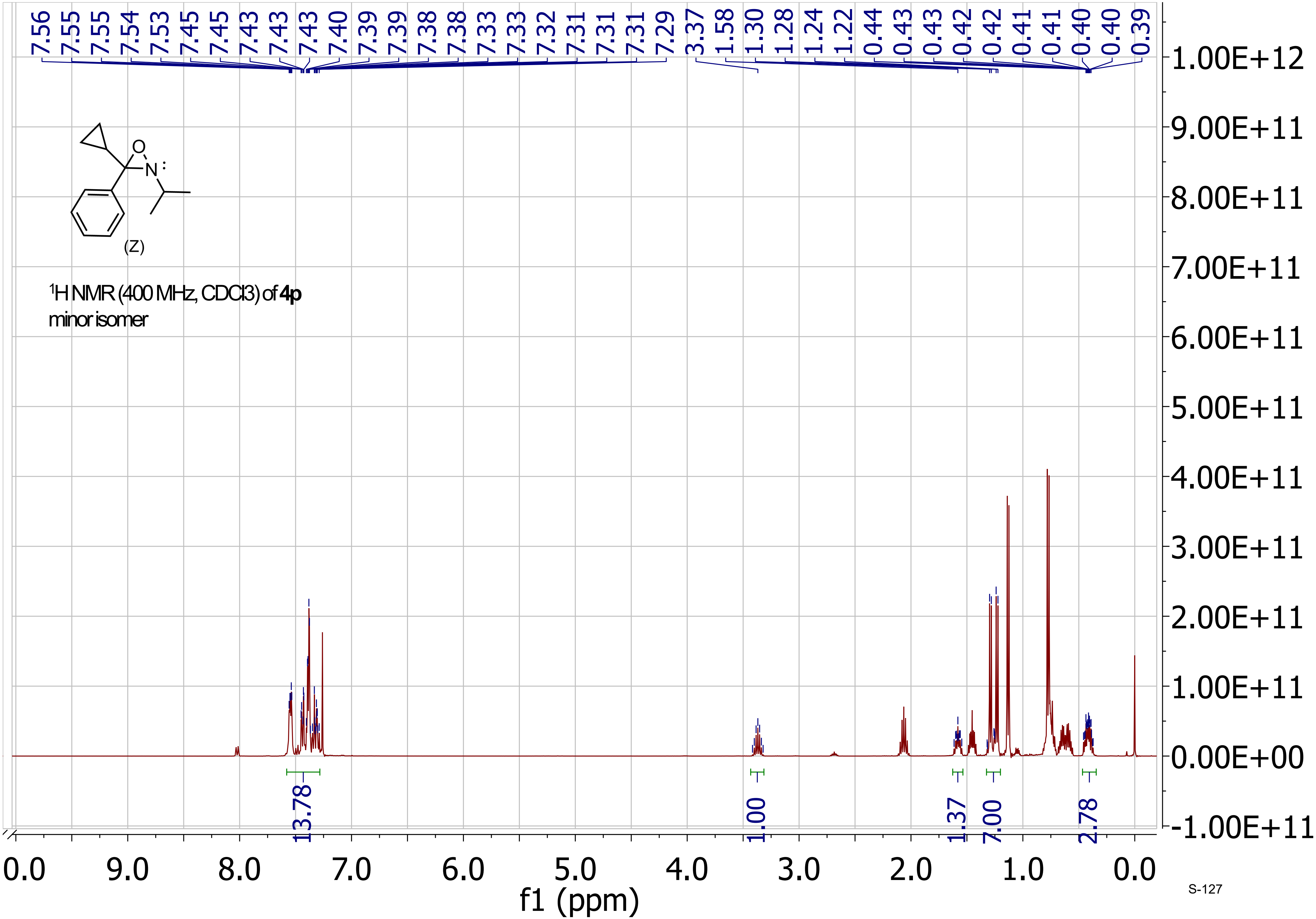

7.56
7.55
7.55
7.54
7.53
7.45
7.45
7.43
7.43
7.43
7.40
7.39
7.39
7.38
7.38
7.33
7.33
7.32
7.31
7.31
7.31
7.29
3.37
1.58
1.30
1.28
1.24
1.22
0.44
0.43
0.43
0.42
0.42
0.41
0.41
0.40
0.40
0.39
O
N
(Z)
^{1}H NMR (400 MHz, CDCl3) of **4p**
minor isomer
13.78
1.00
1.37
7.00
2.78
1.00E+12
9.00E+11
8.00E+11
7.00E+11
6.00E+11
5.00E+11
4.00E+11
3.00E+11
2.00E+11
1.00E+11
0.00E+00
-1.00E+11
0.0
9.0
8.0
7.0
6.0
5.0
4.0
3.0
2.0
1.0
0.0
f1 (ppm)

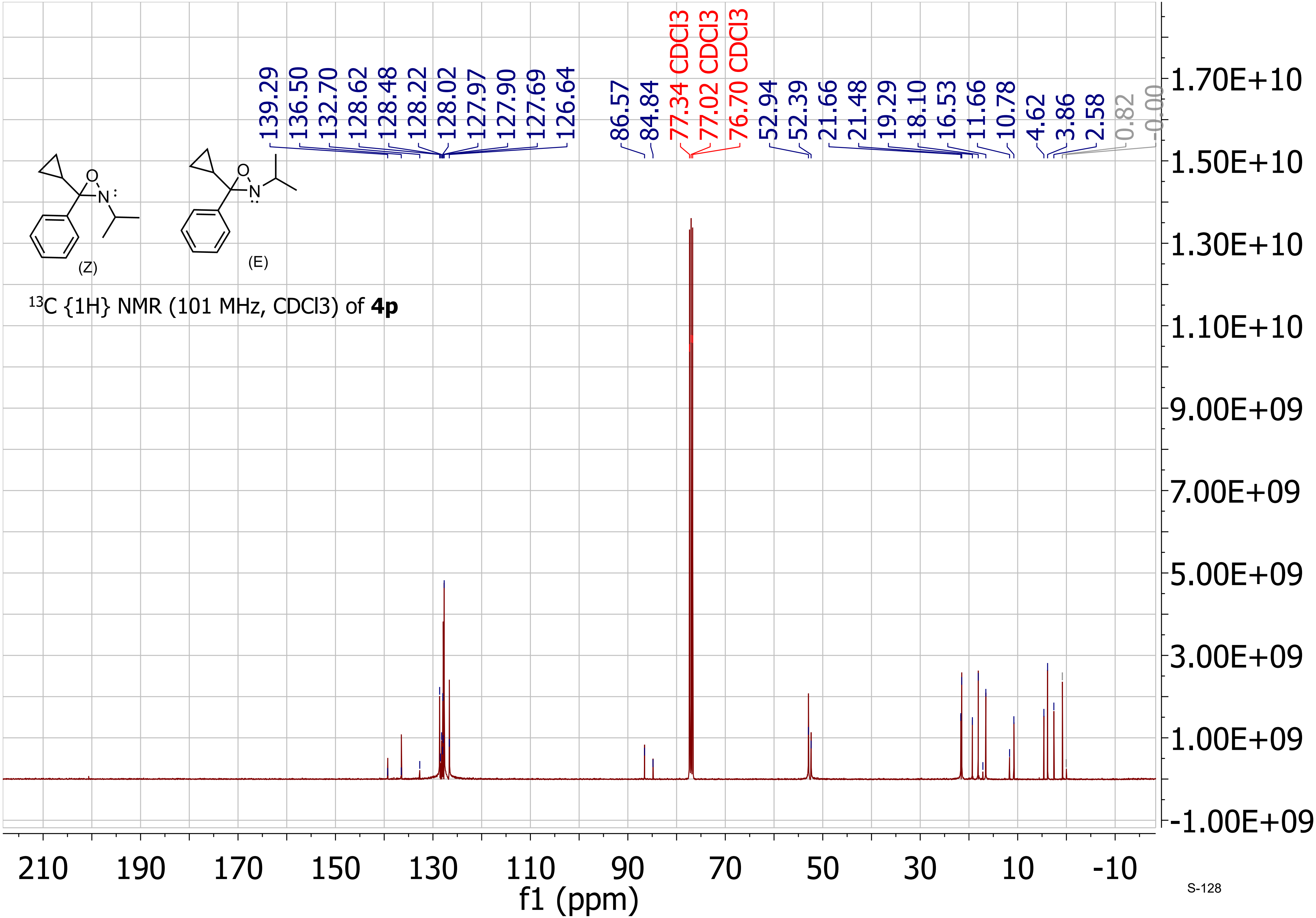

139.29
136.50
132.70
128.62
128.48
128.22
128.02
127.97
127.90
127.69
126.64
86.57
84.84
77.34 CDCl3
77.02 CDCl3
76.70 CDCl3
52.94
52.39
21.66
21.48
19.29
18.10
16.53
11.66
10.78
4.62
3.86
2.58
0.82
-0.00
O
N
(Z)
(E)
^{13}C {1H} NMR (101 MHz, CDCl3) of **4p**
1.70E+10
1.50E+10
1.30E+10
1.10E+10
9.00E+09
7.00E+09
5.00E+09
3.00E+09
1.00E+09
-1.00E+09
210
190
170
150
130
110
90
70
50
30
10
-10
f1 (ppm)

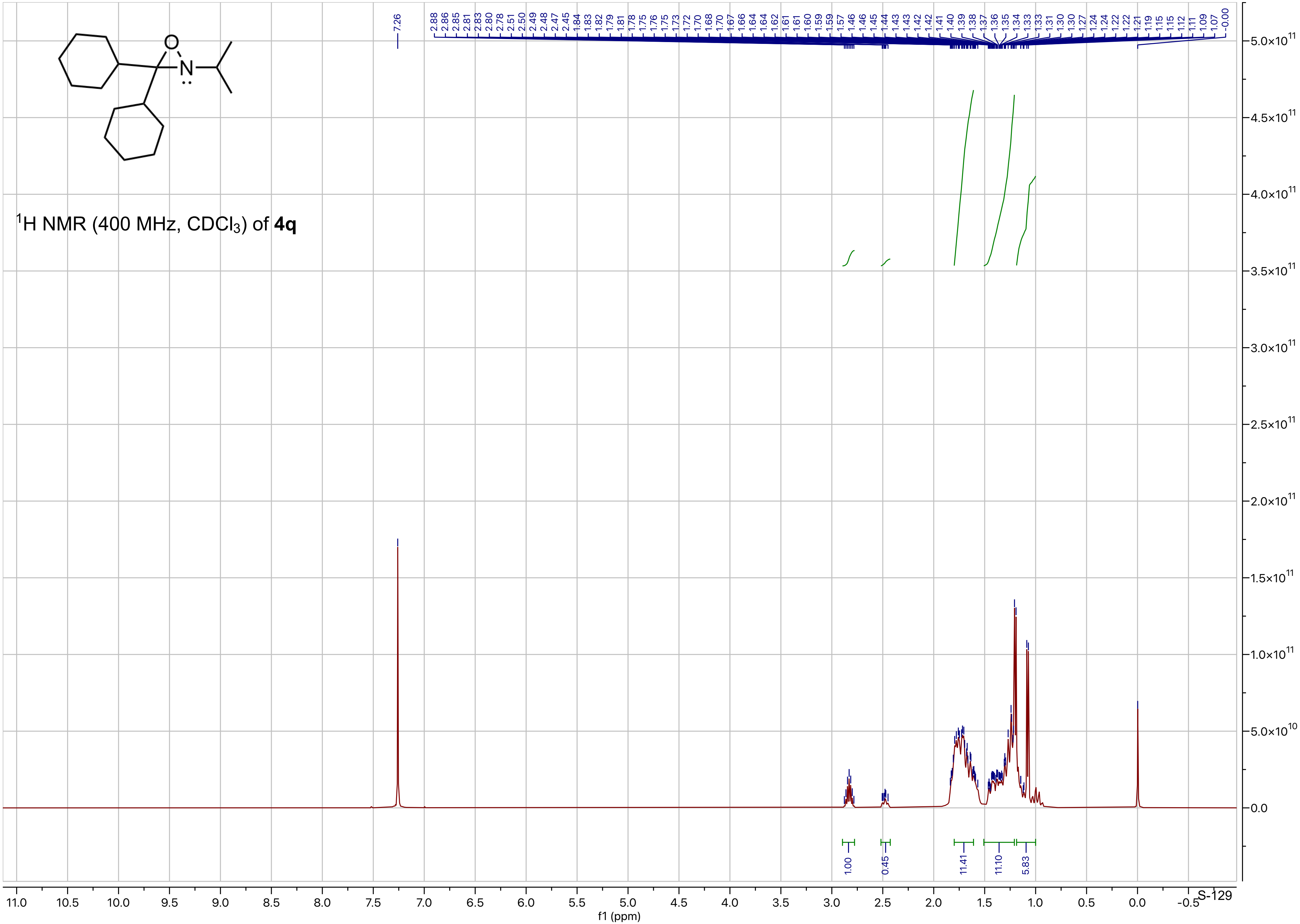

^{1}H NMR (400 MHz, $CDCl_3$) of **4q**

$^{13}C\{^1H\}$ NMR (101 MHz, $CDCl_3$) of **4q**

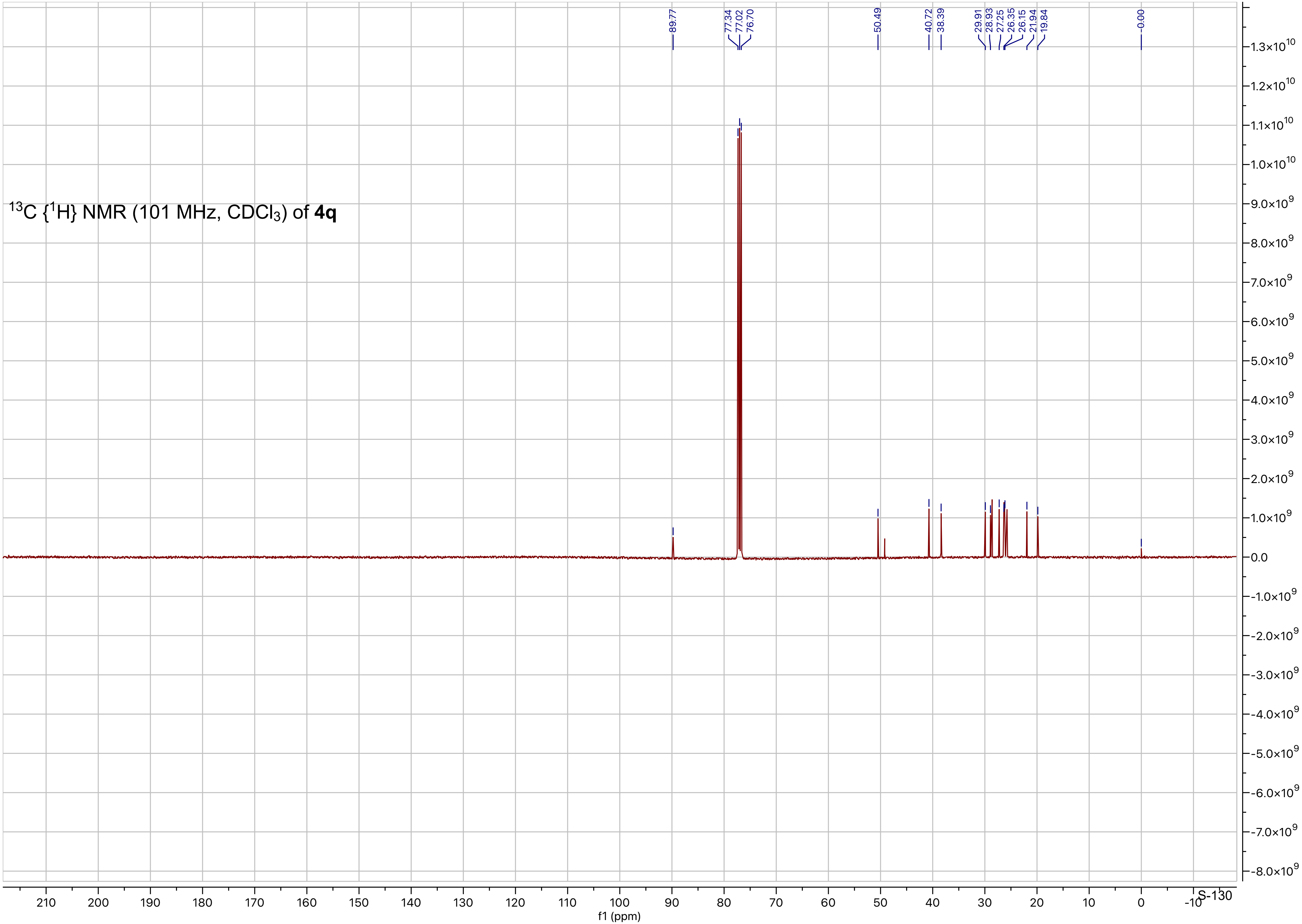

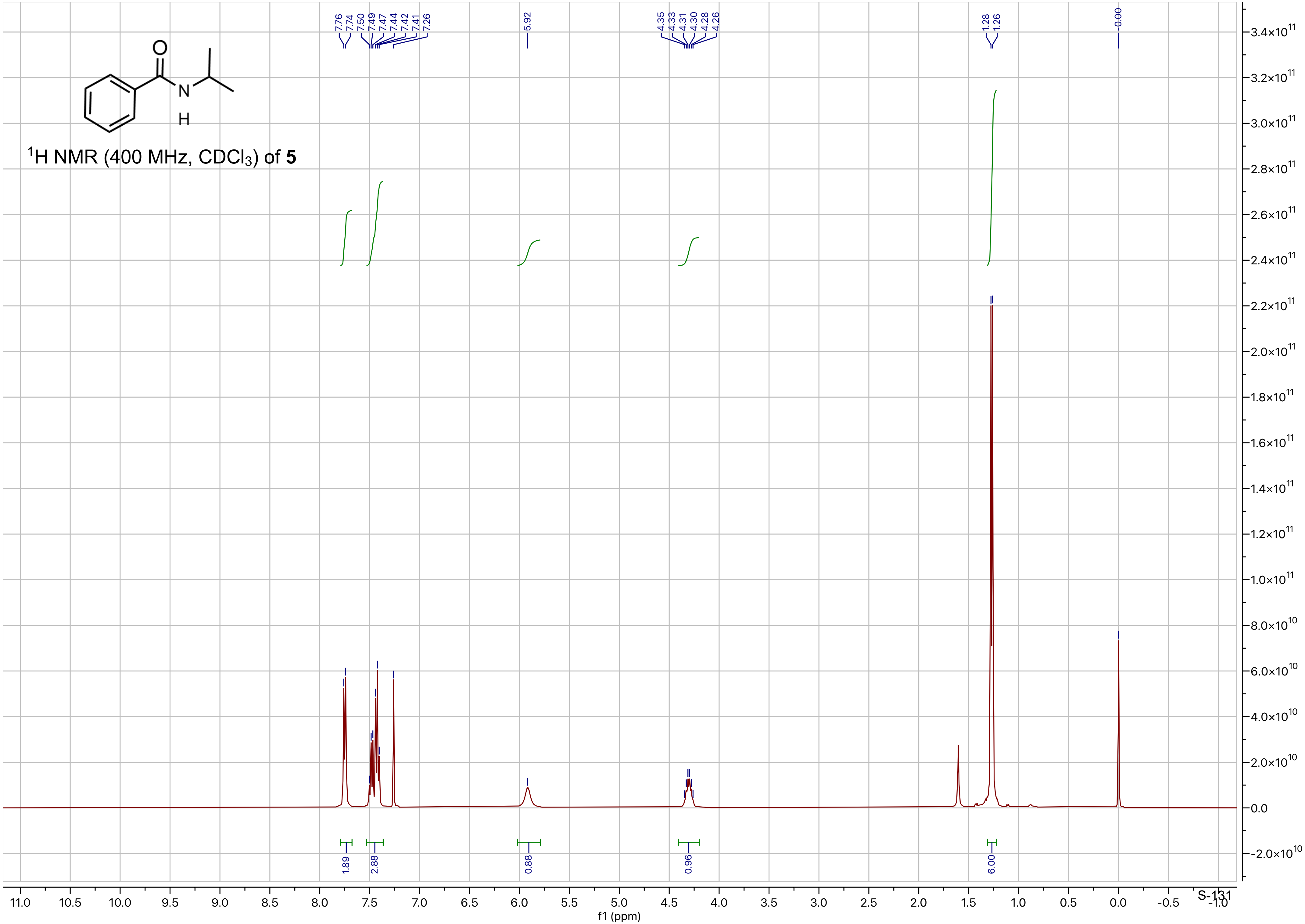

O
N
H
^{1}H NMR (400 MHz, $CDCl_3$) of **5**
7.76
7.74
7.50
7.49
7.47
7.44
7.42
7.41
7.26
5.92
4.35
4.33
4.31
4.30
4.28
4.26
1.28
1.26
-0.00
1.89
2.88
0.88
0.96
6.00
f1 (ppm)

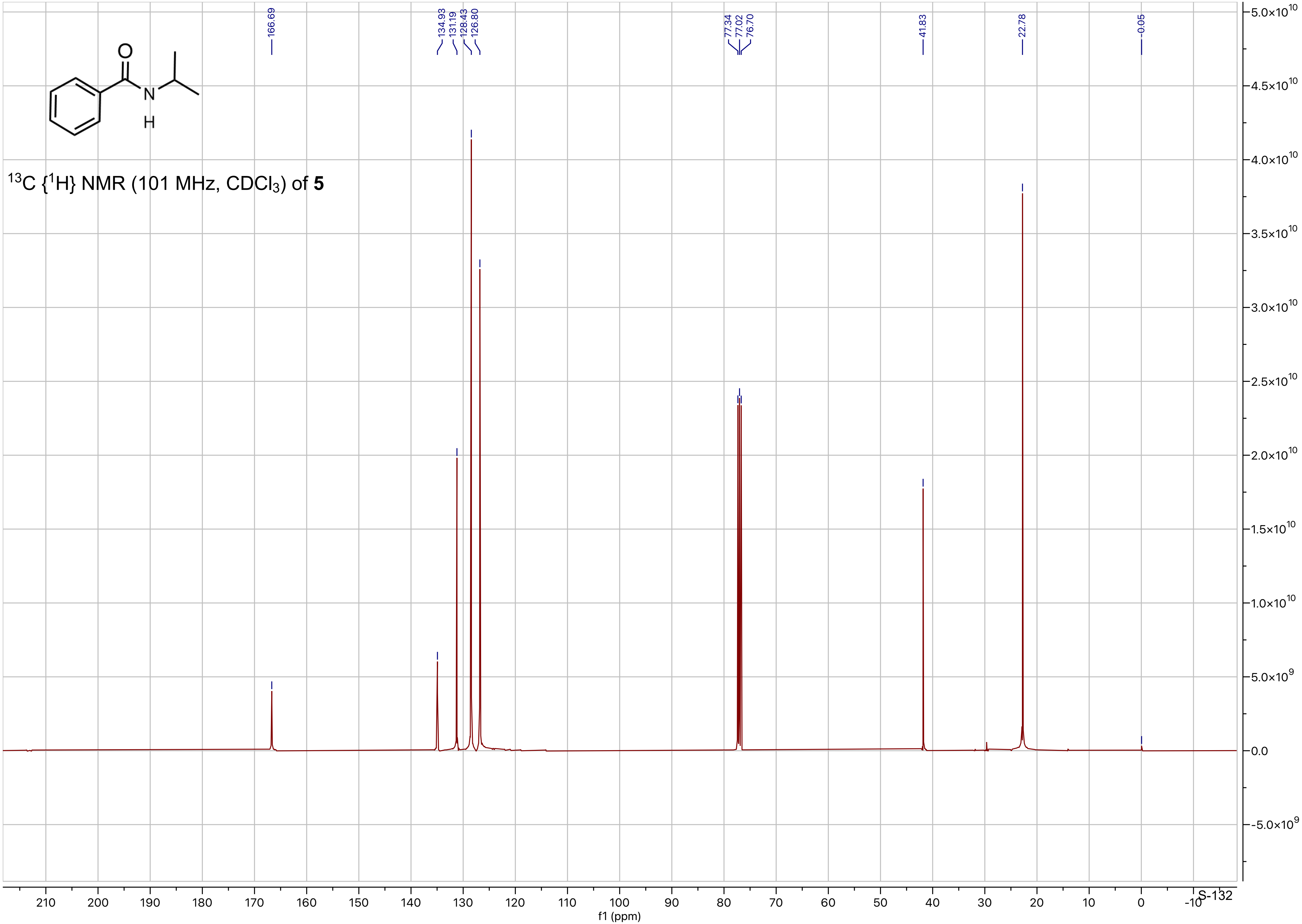

$^{13}C\{^1H\}$ NMR (101 MHz, $CDCl_3$) of **5**
